# Dynamic Allostery in PLCγ1 and Its Modulation by a Cancer Mutation Revealed by MD Simulation and NMR

**DOI:** 10.1016/j.bpj.2018.05.031

**Published:** 2018-07-03

**Authors:** Hans Koss, Tom D. Bunney, Diego Esposito, Marta Martins, Matilda Katan, Paul C. Driscoll

**Affiliations:** 1 Institute of Structural and Molecular Biology, Division of Biosciences, University College London, London, United Kingdom; 2 The Francis Crick Institute, London, United Kingdom

## Abstract

Phosphatidylinositol phospholipase C*γ* (PLC*γ*) is an intracellular membrane-associated second-messenger signaling protein activated by tyrosine kinases such as fibroblast growth factor receptor 1. PLC*γ* contains the regulatory *γ*-specific array (*γ*SA) comprising a tandem Src homology 2 (SH2) pair, an SH3 domain, and a split pleckstrin homology domain. Binding of an activated growth factor receptor to *γ*SA leads to Tyr783 phosphorylation and consequent PLC*γ* activation. Several disease-relevant mutations in *γ*SA have been identified; all lead to elevated phospholipase activity. In this work, we describe an allosteric mechanism that connects the Tyr783 phosphorylation site to the nSH2-cSH2 junction and involves dynamic interactions between the cSH2-SH3 linker and cSH2. Molecular dynamics simulations of the tandem SH2 protein suggest that Tyr783 phosphorylation is communicated to the nSH2-cSH2 junction by modulating cSH2 binding to sections of the cSH2-SH3 linker. NMR chemical shift perturbation analyses for designed tandem SH2 constructs reveal combined fast and slow dynamic processes that can be attributed to allosteric communication involving these regions of the protein, establishing an example in which complex *N*-site exchange can be directly inferred from ^1^H,^15^N-HSQC spectra. Furthermore, in tandem SH2 and *γ*SA constructs, molecular dynamics and NMR results show that the Arg687Trp mutant in PLC*γ*1 (equivalent to the cancer mutation Arg665Trp in PLC*γ*2) perturbs the dynamic allosteric pathway. This combined experimental and computational study reveals a rare example of multistate kinetics involved in a dynamic allosteric process that is modulated in the context of a disease-relevant mutation. The allosteric influences and the weakened binding of the cSH2-SH3 linker to cSH2 should be taken into account in any more holistic investigation of PLC*γ* regulation.

## Introduction

Phosphatidylinositol phospholipases (PLCs) are a family of enzymes that hydrolyze substrate membrane PI(4,5)P_2_ phosphoinositides to yield the important second messengers diacylglycerol and inositol(1,4,5)P_3_ (1, 2). In mammals, the ubiquitously expressed PLC*γ*1 isoform is activated by growth factor receptor tyrosine kinases (RTKs) and the T cell receptor (3, 4). The closely related PLC*γ*2 protein is stimulated by B cell and Fc receptors and mainly found in hematopoietic cells. The expression patterns of PLC*γ*1 and PLC*γ*2 are also reflected by the pathologies linked to various PLC*γ*1 and PLC*γ*2 mutations that activate PLC*γ* catalytic activity. PLC*γ* proteins differ from other PLC subfamily isoforms (PLC *β*, *δ*, *ε*) in that the sequence of the catalytic triose phosphate isomerase barrel domain is interrupted by the regulatory *γ*-specific array (*γ*SA), consisting of a “split” pleckstrin homology (PH) domain, two Src homology 2 (SH2) domains (N-terminal SH2 (nSH2) and C-terminal SH2 (cSH2)), and an SH3 domain. The polypeptide domain arrangement N-spPH–nSH2–cSH2–SH3–C-spPH, in which the N- and C-terminal portions of the split PH domain come together to form a globular domain (5), results in a pseudo-cyclic structure for the *γ*SA.

Activation of fibroblast growth factor receptor 1 (FGFR1) results in phosphorylation of Tyr766 in the unstructured C-terminal tail of the receptor and leads to recruitment of PLC*γ* isoforms (6). Full activation of PLC*γ*1 depends on receptor-dependent phosphorylation of *γ*SA residue Tyr783 and on the presence of a functional cSH2 domain (7, 8, 9). We and others have shown that the cSH2 domain is bound to the catalytic domain to block substrate access to the active site in the resting state (10, 11). Binding of FGFR1 to PLC*γ*1 occurs though the *γ*SA nSH2 domain via both canonical and noncanonical protein-protein interactions (6), though more recently, it has been suggested that recruitment and activation of PLC*γ*1 by a panel of RTKs proceeds via the cSH2 domain alone (12).

Mutations in PLC*γ*s have been detected in a variety of disease conditions through DNA sequence analysis, and these mutations can occur throughout the length of the protein (4). All pathogenic *γ*SA mutations known to date are activating, and all are at sites conserved between PLC*γ*1 and PLC*γ*2 (4). For example, pathogenic *γ*SA mutations are either located in split PH (Tyr495Cys, PLC*γ*2; Ser520Phe, PLC*γ*1; Leu845Phe, PLC*γ*2) or in cSH2 (Arg665Trp, PLC*γ*2; Arg707Gln, PLC*γ*1; Ser707Tyr, PLC*γ*2). Differential expression patterns of PLC*γ*1 and PLC*γ*2 have a great influence on the resulting pathology. For instance, Ser707Tyr (PLC*γ*2), which has been linked to autoimmune disease, was found to disrupt the autoinhibitory cSH2 interface with the enzyme core (10, 13). Arg707Gln (PLC*γ*1) has been linked to secondary angiosarcoma and is suspected to disrupt the cSH2 domain structure and thereby release autoinhibition (14). The cSH2 mutation Arg665Trp (PLC*γ*2) occurs in the context of resistance to the Bruton’s tyrosine kinase inhibitor ibrutinib used in chronic lymphocytic leukemia (15). No structural insight about the consequences of the Arg665Trp (PLC*γ*2)/Arg687Trp (PLC*γ*1) mutation is currently available.

In the PLC*γ* resting state, the extent to which the cSH2 domain is “available” for contact with the enzyme core and upstream agonists is unclear. In this context, the binding status of the cSH2 C-terminus, which is equivalent to the cSH2-SH3 linker and harbors the Tyr771, Tyr775, and Tyr783 phosphorylation sites, plays a particular role; recent studies proposed that Tyr783 phosphorylation by ITK kinase and Tyr771 phosphorylation by FGFR2 kinase would occur because of an enhancement of C-terminus availability (12, 16). Although tandem SH2 crystal structures suggest that the cSH2 C-terminus might indeed be unbound in the nonphosphorylated state (10), a recent NMR study comparing constructs with and without an unmodified C-terminal extension suggested that the C-terminus is bound even in the nonphosphorylated state (16).

Here, we report the results of an investigation of the tandem nSH2-cSH2 segment of PLC*γ*1 and the impact of interactions of the globular cSH2 domain with the C-terminus in both non-phospho- and Tyr783-phospho-states, including within the context of the intact *γ*SA. We present an analysis of the tandem SH2 domain protein by molecular dynamics (MD) simulation and heteronuclear NMR spectroscopy. Unexpectedly, the NMR results reveal a complex signature, the examination of which suggests partial dynamic interaction between the C-terminus and the cSH2 domain. In line with the results yielded from MD simulations, we interpret the NMR data to indicate dynamic allosteric communication between the Tyr783 phosphorylation site and the inter-SH2 domain junction, which operates via the C-terminus and residues preceding the C-terminus. Though examples of dynamic allostery have been presented before, here we introduce a case in which the combination of fast- and slow-exchange phenomena results in complex but interpretable NMR crosspeak patterns. We also characterize the effect of the PLC*γ*1 Arg687Trp mutation—homologous to the cancer therapy resistance Arg665Trp substitution in PLC*γ*2—on the characteristics of the tandem SH2 domain.

## Materials and Methods

Materials and methods used during the course of this study are provided in Supporting Materials and Methods, Section 1.

## Results

**Figure 1:**
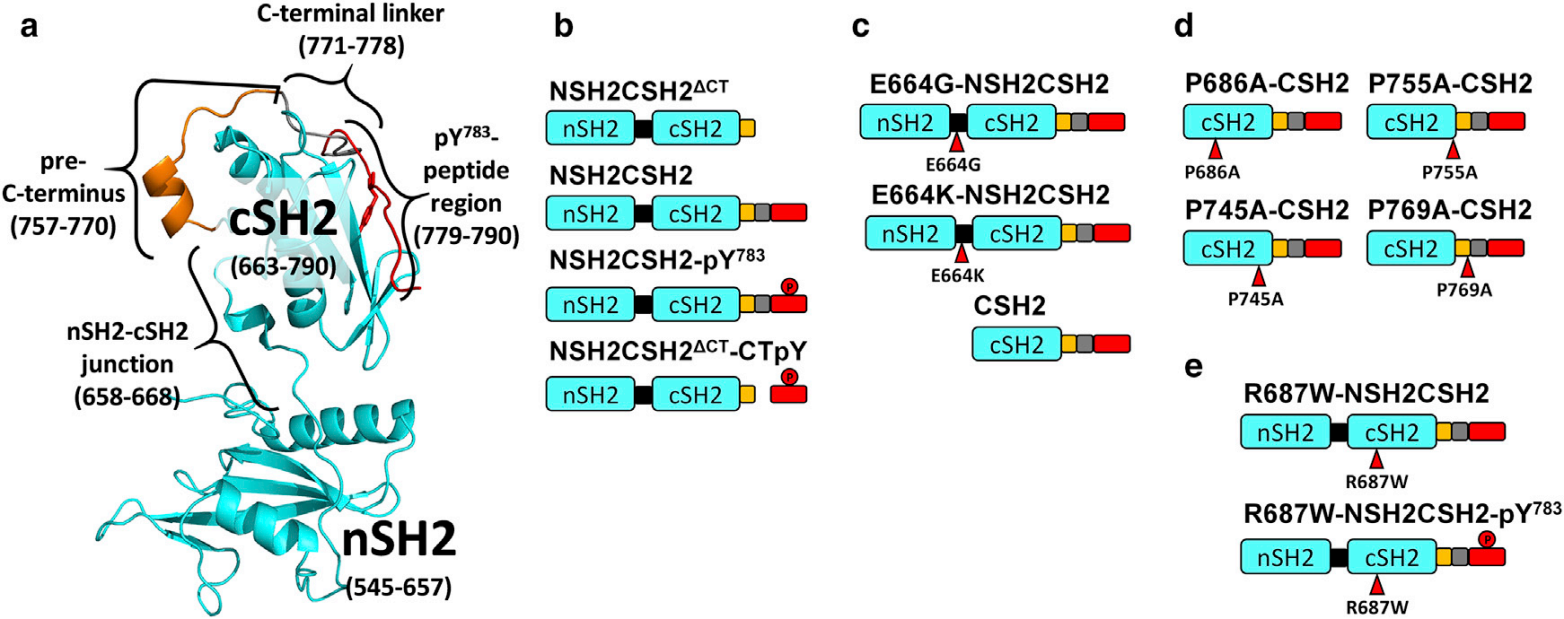
Tandem PLC*γ*1 nSH2-cSH2 constructs and nomenclature. (*a*) Crystal structure (PDB: 4FBN) of the tandem nSH2-cSH2 domains of PLC*γ*1 is shown. Residues 773–782 in the extended C-terminus, which are missing in the electron density, have been modeled for illustration purposes. (*b*–*e*) Schematic illustration shows key protein constructs examined in this study, grouped by function: (*b*) wild-type constructs to assess C-terminal binding, (*c*) derivative constructs to probe the role of the nSH2-cSH2 junction on fast-exchange dynamics and to assess slow exchange, (*d*) constructs with single Pro→Ala substitutions to probe slow exchange, and (*e*) disease-relevant Arg687Trp mutant constructs. The structure shown in (*a*) corresponds to the NSH2CSH2 construct shown in (*b*). The color-coding employed is as follows: cyan, SH2 domains; yellow, pre-C-terminus; gray, C-terminal linker; red, pY783-peptide region. In (*b*) and (*e*), the “P” symbol indicates phosphorylation of Tyr783. NSH2CSH2^*Δ*CT^-CTpY corresponds to the 1:1 complex between NSH2CSH2^*Δ*CT^ and a synthetic phosphopeptide (CTpY) corresponding to residues 779–790 in the NSH2CSH2 protein.

Our principal aim was to probe the impact of Tyr783 phosphorylation on the structural and dynamical properties of both the PLC*γ*1 nSH2 and cSH2 domains. The following will discuss in detail the structure and dynamics of different PLC*γ*1 tandem SH2 proteins (Fig. 1). According to the canonical description of SH2 domains, the ordered part of the PLC*γ*1 nSH2 domain comprises residues 545–662 and the cSH2 domain residues 663–756. The junction between the two SH2 domains (“nSH2-cSH2 junction”) is here defined as the residue region 658–668. The region of the polypeptide that encompasses residues to the C-terminus of the cSH2 domain can be described as comprising three segments: the “pre-C-terminus” (residues 757–770), the “C-terminal linker” (residues 771–778), and the “Tyr783-peptide region” (residues 779–790). Together, the C-terminal linker and the Tyr783 peptide region constitute the “C-terminus”; the combination of all three segments is referred to here as the “extended C-terminus.”

Three crystal structures (Protein Data Bank (PDB): 3GQI, 4FBN, 4EY0) are available for the tandem nSH2-cSH2 protein. The structures reported by Bunney et al. comprise a “long” unmodified construct (residues 545–790, which include the nSH2, cSH2 and C-terminal regions) and a similar construct that was phosphorylated on the Tyr783 side chain (PDB: 4FBN and 4EY0, respectively) (10). The three-dimensional (3D) structure of 4FBN is illustrated in Fig. 1 *a*. In the structures, electron density for residues 773–782 (4FBN) and 774–781 (4EY0), mainly corresponding to the C-terminal linker region, is missing, suggesting that this segment is dynamic in nature. In 4EY0, electron density corresponding to the phosphorylated Tyr783 peptide region is observed in the typical SH2 peptide-binding groove, indicating binding in *cis*. In the case of 4FBN, similar but much weaker density is observed, suggesting that at least partial binding can occur in the absence of Tyr783 phosphorylation. We sought to explore the effect of Tyr783 phosphorylation on the PLC*γ*1 tandem SH2 domain protein in solution using MD and heteronuclear NMR spectroscopy.

### MD simulations to probe structural changes in the extended C-terminus and the nSH2-cSH2 junction upon Tyr783 phosphorylation

MD simulations were used to generate hypotheses about the structural consequences of Tyr783 phosphorylation and to guide subsequent experiments. We performed six atomistic 100 ns MD simulations of NSH2CSH2 and four atomistic 100 ns simulations of NSH2CSH2-pY^783^ using starting models derived from the crystal structures with PDB: 4FBN and 4EY0, respectively (corresponding to constructs for NMR, vide infra).

We evaluated convergence for local motions in the cSH2 domain by comparing contact and eigenvalue ranges for different MD trajectories using identical eigenvector (principal component) sets from a principal component analysis (PCA). The trajectories were not of sufficient duration to satisfactorily predict the full range of protein motion, interdomain flexibility, and orientation that might be present in solution; special approaches that enhance sampling of the conformational space would be required to accurately address questions regarding interdomain properties of the tandem SH2 constructs. Nevertheless, we identified differences within the cSH2 domain between NSH2CSH2 and NSH2CSH2-pY^783^ trajectories.

**Figure 2:**
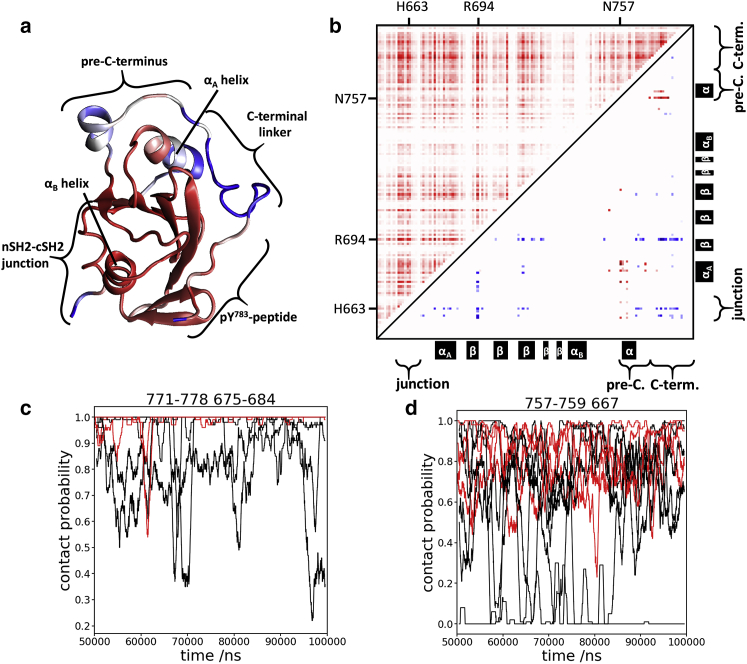
Molecular dynamics simulations predict dynamic differences between NSH2CSH2 and NSH2CSH2-pY^783^ for residues in the pre-C-terminus and the C-terminal linker. (*a*) Root mean-square fluctuation (RMSF) of the second eigenvector of the NSH2CSH2 PCA contains information about differences in backbone motions of the cSH2 domain between NSH2CSH2 and NSH2CSH2-pY^783^ (see also Fig. S1). The data suggest that the (pre-)-C-terminus could be relevant for an allosteric connection between Tyr783 and the nSH2-cSH2 junction. RMSF data were projected on a simulation model using color scaling: red (no fluctuation)–white–blue (high fluctuation). The particularly high RMSF for parts of the pre-C-terminus and the C-terminus is evident. (*b*) Mutual information between side-chain dihedral torsion angles for NSH2CSH2 and NSH2CSH2-pY^783^ trajectories is shown. The results for the cSH2 domain are shown in this panel (for the entire construct, see Fig. S2*b*). Upper left half: residue-wise sum of mutual information between side-chain dihedral angles in NSH2CSH2 is shown; results were averaged but not filtered. Lower right half: mutual information difference between NSH2CSH2 and NSH2CSH2-pY^783^ is shown filtered (*t*-test). Red indicates an increase in mutual information in the phosphorylated protein (marked as described in the main text); blue indicates a decrease in mutual information. Particularly interesting residues as well as secondary structure elements are indicated on the plot abscissae. (*c* and *d*) Contact probabilities are shown between C-terminal linker and *α*_A_-helix (*c*) or between pre-C-terminal residues 757–759 and nSH2-cSH2 junction residue 667, shown for different MD trajectories. Black, NSH2CSH2 (*n* = 6 trajectories); red, NSH2CSH2-pY^783^ (*n* = 4). Any number of contacts (<2.5 Å) at a given time point is counted as a single contact event, yielding a contact probability for a 1 ns sliding average time bin.

PCA in Cartesian space for backbone atoms of the cSH2 domain and the nSH2-cSH2 junction was performed to provide a quantitative analysis of motion represented in the trajectories (Fig. 2 *a*; Fig. S1). Eigenvectors were identified using merged trajectories for each set of simulations. The trajectories for the nonphosphorylated protein converged, as judged by overlap of the range of trajectory projections on the most significant eigenvectors. Analysis of the projections of each trajectory on the PCA eigenvectors revealed a difference between NSH2CSH2-pY^783^ and NSH2CSH2 trajectories in NSH2CSH2 eigenvector 2 (Fig. S2 *b*). Specifically, a high root mean-square fluctuation (RMSF) of the C-terminal linker and the pre-C-terminus in this eigenvector (Fig. 2 *a*) suggests that the dynamics of this region differs between NSH2CSH2 and NSH2CSH2-pY^783^.

To further explore this finding, we performed contact analyses of the trajectories. We specifically focused on contacts between the extended C-terminus and the nSH2-cSH2 junction on one hand and other regions of the protein on the other hand (Fig. 2 *a*). For these protein regions, we use the secondary structure nomenclature that has been widely employed in previous descriptions of SH2 domains (17). Of particular note to this investigation, the *α*_A_-helix (Arg675-Arg684) forms a rather exposed part of the cSH2 domain; the shorter *α*_B_-helix (Asp634-Gln641) is close in space to the nSH2-cSH2 junction (Glu650-Trp668). The results of the contact analysis are shown in Fig. 2, *c* and *d* and Document S1. Supporting Materials and Methods, Supporting Results, Figs. S1–S15, and Data S1 and S2, Figure360: An Author Presentation of Fig. 7 *a* for the extended C-terminus, and in Document S1. Supporting Materials and Methods, Supporting Results, Figs. S1–S15, and Data S1 and S2, Figure360: An Author Presentation of Fig. 7 *a* for the *α*B-helix/nSH2-cSH2 junction. Here, we focus on the most significant differences in contact probability between the NSH2CSH2 and NSH2CSH2-pY^783^ trajectories related to the pre-C-terminus and the C-terminus. As expected, the contact between pTyr783 and the cSH2 domain was essentially permanent in the NSH2CSH2-pY^783^ trajectories but less stable in the NSH2CSH2 trajectories (Fig. S1 *b*). The contact between the following interface pairs was more frequent in the phosphorylated constructs: (p)Tyr783 and cSH2 domain core (670–750), C-terminal linker and cSH2 domain *α*_A_-helix, pre-C-terminal region Asn757-Lys759 and cSH2 domain core, and pre-C-terminal region Asn757-Lys759 and nSH2-cSH2 junction residue Glu667 (Figs. 2 and S2 *a*). Thus, the MD simulations suggest that Tyr783 phosphorylation modulates the interactions between the pre-C-terminus and the nSH2-cSH2 junction and leads to stronger interactions of the extended C-terminus with the cSH2 domain.

We used the tool MutInf (18) to predict allosteric pathways from the MD trajectories by analyzing mutual information in the distribution of side-chain dihedral angles (Fig. 2 *b*). Particularly strong mutual information was found in the pre-C-terminus, the C terminus, and cSH2 core, which are likely to be in direct contact with these elements. Importantly, significant differences in mutual information were detected between NSH2CSH2 and NSH2CSH2-pY^783^. The mutual information between pre-C-terminus residues Asn757 and Glu759 on one hand and residues in the 1) C-terminal linker and 2) cSH2 domain—specifically the *α*_A_-helix—on the other hand is higher in the NSH2CSH2-pY^783^ simulations (Fig. 2 *b*). Conversely, the mutual information between various residues in the C-terminus, cSH2 residue Arg694, and junction residues Thr660 and His663 is lower for the NSH2CSH2 trajectories. There was also a lower level of mutual information between Arg694 and the nSH2-cSH2 junction on one hand and on the other hand several nSH2 residues (Fig. S2 *b*), only some of which are spatially close to the nSH2-cSH2 junction (Asn547-Arg567), whereas others are more distant (Asp615, Gly617, Glu589, Arg645-Glu649). Our data suggest an allosteric connection between the Tyr783 phosphorylation site and the nSH2-cSH2 junction (and to a minor extent, the nSH2 domain), which primarily involves sections of the extended C-terminus and the cSH2 domain residues with which they interact. Together, the MD PCA, contact, and MutInf analysis results suggest the potential presence of dynamic interplay between the extended C-terminus and the cSH2 domain.

### Constructs for NMR spectroscopy

For NMR experiments, we prepared a variety of tandem-SH2 proteins; key constructs are illustrated schematically in Fig. 1 *b*. Similar to the proteins in the crystal structures, we prepared unmodified (non-phospho) “NSH2CSH2” (residues 545–790) and Tyr783-phosphorylated “NSH2CSH2-pY^783^” (residues 545–790, pTyr783). We also prepared a construct comprising solely the cSH2 domain and the appended C-terminal region (“CSH2”; residues 663–790), a shortened form of the tandem lacking the C-terminal residues 545–770 denoted “NSH2CSH2^*Δ*CT^,” and a complex of NSH2CSH2^*Δ*CT^ with a pTyr783 phosphopeptide (residues 779–790) denoted “NSH2CSH2^*Δ*CT^-CTpY”; for the last sample, the CTpY peptide was added to 1.2 equivalents (CTpY binding to cSH2 has been described in a previous study (10)). From titration experiments, we confirmed that CTpY peptide binds sufficiently strongly (*K*_D_ < 15 *μ*M) to the cSH2 domain to be saturating under the NMR measurement conditions (Fig. S6; Supporting Materials and Methods, Section 2.7). In addition, we prepared single residue Gly and Lys mutants at junction residue Glu664 of NSH2CSH2 (“E664G-NSH2CSH2” and “E664K-NSH2CSH2”), both nonphospho- and phospho-preparations of a mutant Arg687Trp construct (“R687W-NSH2CSH2” and “R687W-NSH2CSH2-pY^783^”), and single residue Ala mutants of CSH2 at Pro686, Pro745, Pro755, and Pro769.

### Backbone crosspeak assignments and chemical shift perturbation analyses

^1^H,^15^N-heteronuclear single quantum coherence HSQC spectra of the tandem nSH2-cSH2 proteins constructs were generally well dispersed (Fig. S3). ^1^H-, ^15^N-, ^13^C_*α*_-, ^13^CO, and ^13^C_*β*_ crosspeak assignments were obtained for NSH2CSH2-pY^783^ and NSH2CSH2 using standard 3D NMR experiments. ^1^H- and ^15^N-backbone crosspeak assignments for all other constructs were transferred by inspection of ^15^N-HSQC experiments. For NSH2CSH2^*Δ*CT^ and R687W-NSH2CSH2-pY^783^, 3D HNCA data sets were recorded to support the assignments. The assignment is most complete for NSH2CSH2-pY^783^ (87%) and least complete for NSH2CSH2^*Δ*CT^ (53%). Some crosspeaks for the cSH2 domain, in the nSH2-cSH2 junction, and in the extended C-terminus could not be located, suggesting intermediate exchange broadening. Multiple crosspeaks were observed for several residues in mostly, but not exclusively, CSH2, E664G-NSH2CSH2, and E664K-NSH2CSH2, indicating the presence of slow exchange. Spectra of nonphospho constructs generally displayed weaker intensity than their phospho counterparts. Although pronounced line broadening in the cSH2 domain is often a result of the aforementioned exchange phenomena, the generally lower signal/noise observed for all residues (also in the nSH2 domain) is likely attributable to a propensity to weak self-association. We have characterized this behavior with ^15^N nuclear relaxation and small angle x-ray scattering data obtained for NSH2CSH2 and NSH2CSH2-pY^783^ (Fig. S4; Supporting Materials and Methods, Section 2.1). Notably, the crosspeak positions and the extent of peak doubling were concentration independent. On the other hand, the presence of weak self-association confounded detailed linewidth analysis and relaxation dispersion experiments; hence, our NMR analyses are predominantly based on chemical shift patterns. In most of the following description, only the major (most intense) crosspeak is considered when peak doubling is present.

### The C-terminus is partially bound in NSH2CSH2 constructs

Interactions between an SH2 domain and a cognate phosphopeptide ligand typically exhibit slow-exchange characteristics, which is consistent with relatively high affinity binding and a slow off-rate (19). On this basis, the spectrum of NSH2CSH2^*Δ*CT^ was expected to display different chemical shifts compared to either NSH2CSH2-pY^783^ or NSH2CSH2^*Δ*CT^-CTpY arising from the occupation of the cSH2 binding site by the C-terminal pTyr783 peptide in the latter cases. In line with this expectation, the NMR spectra of NSH2CSH2^*Δ*CT^ and NSH2CSH2-pY^783^ appear quite different (Fig. S5). For the purposes of describing our observations, it is useful to declare the following: trivially, for NSH2CSH2^*Δ*CT^, no interaction of the pTyr783 region with the cSH2 domain is present, and the protein is in an unbound or “open” state. On the other hand, the characteristics of the NSH2CSH2-pY^783^ protein, with at least pTyr783 of the C-terminal tail occupying the cSH2 binding site, can be regarded as representing a bound or “closed” state. Importantly, the largest chemical shift perturbations (CSPs) between the “open” and “closed” forms were not confined to the putative binding contact zones in the cSH2 domain and pTyr783 region but were also observed for sites in the pre-C-terminus (distant from the pY783 binding site), the C-terminal linker, and throughout the cSH2 domain.

**Figure 3:**
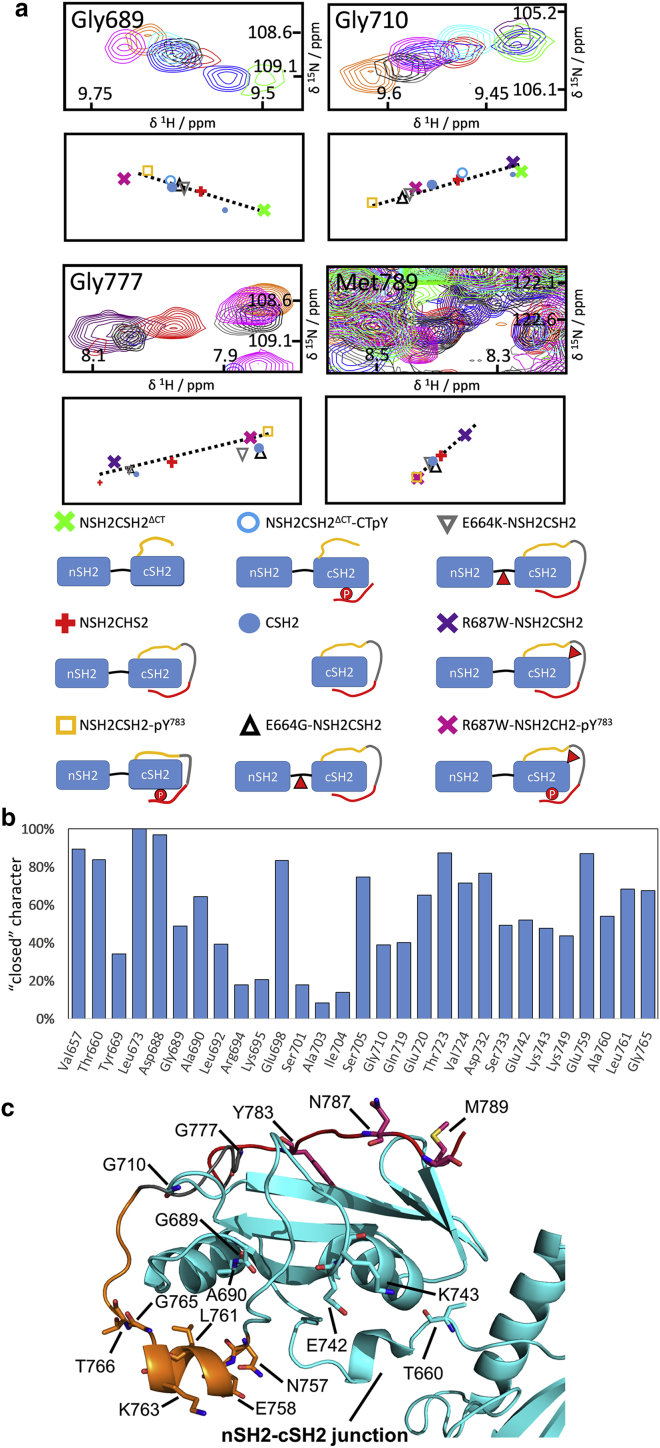
NMR spectra demonstrate that the C-terminus is partially bound in NSH2CSH2 constructs. (*a*) Superposition of the ^1^H,^15^N-HSQC NMR spectra of the tandem nSH2-cSH2 constructs is shown. Each pair of panels is focused on a particular residue and shows the raw NMR spectra (*upper panel*) with schematic illustrations of the crosspeak centroids (*lower panel*). The contour colors and centroid symbols are plotted according to the protein construct, as indicated in the key below, which uses cartoon representations wherein the NSH2-CSH2 junction, pre-C-terminus, C-terminal linker, and Tyr783 region are shown in black, yellow, gray, and red, respectively; phosphorylation of Tyr783 is indicated by a red circle with a white “P,” and sites of single residue substitutions are indicated by a red arrowhead. In each case, the crosspeaks are located on a vector connecting positions corresponding to fast-exchanging “open” (O) and “closed” (C) states (see main text). (*b*) Plot of the degree of “closed” character in the NSH2CSH2 construct is shown, estimated from the position of the corresponding crosspeaks on the O-C vector. (*c*) Model of the cSH2 protein (based on 4FBN) shows the location of residues whose crosspeak chemical shifts demonstrate O ⇌ C fast-exchange.

Superposition of the NMR spectra for all of the other constructs that we have examined shows that the chemical shifts for conserved residues do not always coincide with those of either NSH2CSH2^*Δ*CT^ (“open” state) or NSH2CSH2-pY^783^ (“closed” state). The crosspeaks for a given residue lie on or close to the vector that joins the corresponding crosspeaks for the “open” and “closed” states. Importantly, this behavior was detected over a whole series of different constructs, several of which were not prepared with this specific phenomenon in mind but for other purposes, and so, this was an adventitious observation. For example, residues Gly777 and Gly789 from the C-terminus and residues Gly689, Ala690, and Gly710, which are located on the cSH2 protein surface, exhibit this behavior (Fig. 3 *a*). This pattern suggests an underlying fast-exchange phenomenon; importantly, the population of “closed” and “open” states for any given construct and residue can be directly inferred by comparing the corresponding peak position to those for the reference “open” and “closed” states, similar to the procedure employed in chemical shift projection analysis (CHESPA) (20). Given their respective locations in the 3D structure, these two sets of residues could take part in mutual interactions that would accompany entry of the extreme C-terminal pTyr783 region into the cSH2 binding site. The MD simulations suggest high mobility and multiple transient contacts between the C-terminus and the cSH2 domain. Therefore, the fast-exchange phenomenon suggested by the pattern of chemical shifts can be attributed to a dynamic interaction between the C-terminus and the cSH2 domain; the local conformation of the NSH2CSH2 protein shuttles between at least two states, rationalized by partial binding of the C-terminal region to the cSH2 domain despite the absence of Tyr783 phosphorylation. This interaction is consistent with the occupation by the nonphosphorylated Tyr783 region of the cSH2 binding site in the 4FBN crystal structure of the NSH2CSH2 protein. The fast-exchange NMR characteristic and the weak electron density in the crystal structure suggest that this interaction is substantially weaker than in the case of NSH2CSH2-pY^783^ but, compared to an untethered nonphospho-Tyr783 peptide, might be promoted by the enhanced effective concentration provided by the tethering pre-C-terminus and C-terminal linker.

Multiple NSH2CSH2 crosspeak patterns evidencing fast exchange were identified; the corresponding locations are projected onto a 3D structural representation in Fig. 3 *c*, whereas peak patterns are displayed in Figs. 3 *a*, S7, S11, and S12. Residue-wise examination of the crosspeak positions reveals that the “open” and “closed” state population distribution differs from residue to residue (Fig. 3 *b*), indicating that the dynamic situation in NSH2CSH2 cannot be properly described by a single two-state fast-exchange model applicable to the whole protein construct. Rather, the pattern suggests a heterogeneous dynamic interaction between the cSH2 domain and different parts of the C-terminal tail. For this reason, it is not possible to determine a single *K*_D_ or *k*_ex_ parameter to describe globally the equilibrium between “open” and “closed” forms.

### C-terminal linker-dependent dynamic allosteric communication of Tyr783 phosphorylation to the nSH2-cSH2 junction

**Figure 4:**
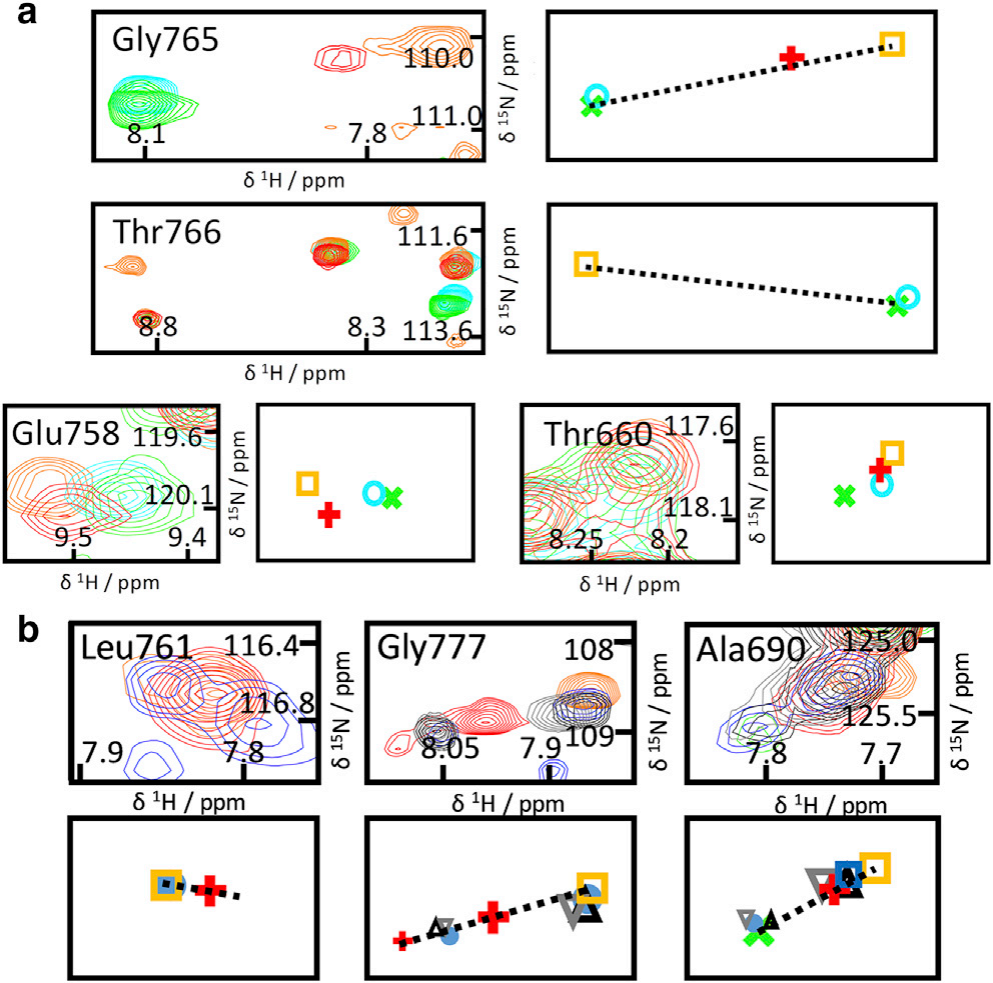
NMR evidence for a C-terminal-linker-dependent allosteric pathway connecting the Tyr783 phosphorylation site with the nSH2-cSH2 junction. (*a* and *b*) Superposition of ^1^H,^15^N-HSQC spectra is shown next to supporting schematic representations of crosspeak centroids ((*a*) 600 MHz, (*b*) 700 MHz, using the same color scheme as in Fig. 3; further examples are shown in Fig. S7). (*a*) For many extended C-terminus residues, only the crosspeaks for NSH2CSH2^*Δ*CT^ and NSH2CSH2^*Δ*CT^-CTpY coincide, indicating a C-terminal-linker-dependent effect. Thr660 (nSH2-cSH2 junction) is affected by Tyr783 phosphorylation in part via the bridging C-terminal linker. (*b*) The crosspeaks for the residues shown are shifted toward the “closed” state when the nSH2 domain is absent (CSH2) or when the nSH2-cSH2 junction is mutated, consistent with allosteric communication via the extended C-terminus.

Similar to the situation with NSH2CSH2, a subset of cSH2 residues of NSH2CSH2^Δ^^CT^-CTpY display evidence of heterogeneous “open” ⇌ “closed” dynamics, corresponding to a partially bound pre-C-terminus (Fig. 3 *a*; Fig. S7). However, the crosspeaks for these residues are directly coincident only for the NMR spectra of NSH2CSH2^Δ^^CT^ and NSH2CSH2^Δ^^CT^-CTpY. This pattern indicates that upon Tyr783 phosphorylation, CSP for the pre-C-terminus (Glu758, Ala760, Leu761, Gly765, Thr766) toward the “closed” state is observed only when the C-terminal linker (residues 771–778) is present (Fig. 4 *a*); the interaction between the cSH2 domain and the pre-C-terminus is dependent upon an intact tether between the pre-C-terminus and the C-terminal tail.

**Figure 5:**
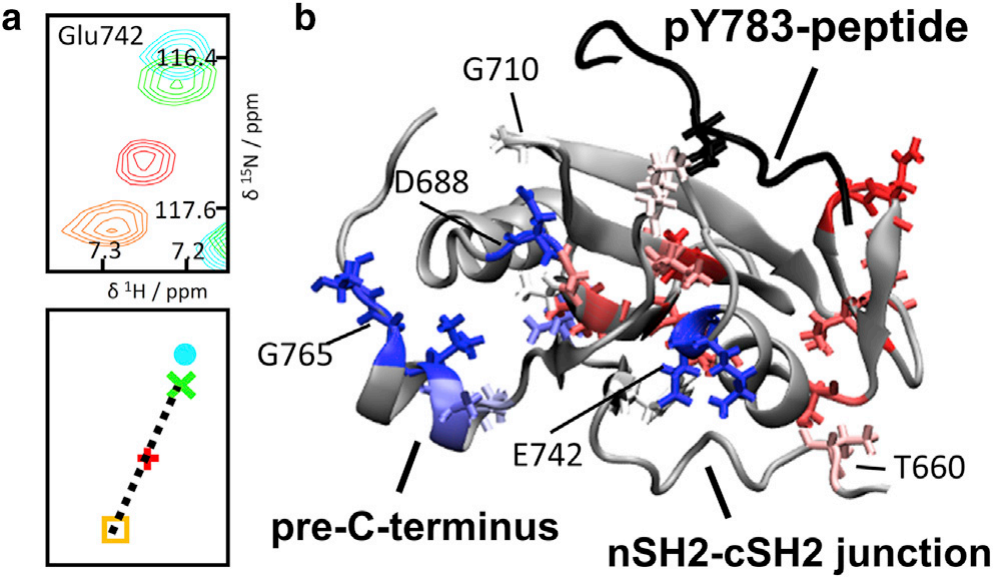
C-terminal-linker-dependent and C-terminal-linker-independent contributions to allosteric communication. (*a*) Superposition of ^1^H,^15^N-HSQC NMR spectra and corresponding schematic representation of the crosspeak centroids for residue Glu742 are shown. The color scheme is as depicted in Fig. 3. The crosspeaks for NSH2CSH2-pY^783^ and NSH2CSH2^*Δ*CT^-CTpY are shifted in opposite directions along the “fast-exchange vector,” exposing a differential impact of allosteric influences. (*b*) Depiction of the cSH2 domain illustrates how the Tyr783 status is communicated via two distinct allosteric pathways (the C-terminal linker is not shown; pY783-peptide is in *black*). The communication type is graded by color: C-terminal-linker-independent allosteric pathway in red—via white—to C-terminal-linker-dependent communication in blue. Only residues with data are displayed in stick model form. The communication type was determined by the projection of the NSH2CSH2^*Δ*CT^-CTpY peak on the vector connecting the NSH2CSH2-pY^783^ and NSH2CSH2^*Δ*CT^ crosspeaks.

The peak position for nSH2-cSH2 junction residue Thr660 is perturbed by phosphorylation of Tyr783 both for NSH2CSH2^*Δ*CT^-CTpY and to a greater degree for NSH2CSH2-pY^783^ (with respect to NSH2CSH2^*Δ*CT^; Fig. 4 *a*). As the separation between Thr660 and the C-terminus is substantial (the distance between Thr660 and Tyr783 C*α* atoms is ∼23.9 Å), the shift pattern suggests that Tyr783 phosphorylation is communicated to Thr660 in the nSH2-cSH2 junction, at least in part, via the tethering C-terminal linker. In line with this observation, substantial chemical shift differences between NSH2CSH2-pY^783^ and NSH2CSH2^*Δ*CT^-CTpY were observed throughout the cSH2 domain and the pre-C-terminus (Document S1. Supporting Materials and Methods, Supporting Results, Figs. S1–S15, and Data S1 and S2, Figure360: An Author Presentation of Fig. 7 *c*; Fig. 5 *b*). One can rationalize these chemical shift patterns on the basis of a dynamic allosteric connection between the Tyr783 binding site and the nSH2-cSH2 junction.

To confirm the dynamic allosteric connection between the nSH2-cSH2 junction and the Tyr783-binding site, we assessed mutants that modulate the fast-exchange equilibria state populations for multiple residues in a manner consistent with long-range dynamic allostery. The nSH2-cSH2 junction has the potential to provide a tight linkage between the two SH2 domains. The NMR spectra of the isolated cSH2 domain (CSH2, a construct that is equivalent to deletion of the nSH2 domain from the NSH2CSH2 protein) and nSH2-cSH2-junction mutants E664G-NSH2CSH2 and E664K-NSH2CSH2 provide an opportunity to trace which cSH2 residues are influenced by the presence of the nSH2 domain and/or an intact nSH2-cSH2 junction. Relative to the wild-type tandem domain constructs, these proteins can be characterized as “junction-disrupted.” Moreover, along with the wild-type (WT) proteins, the spectra of these mutants are helpful in dissecting the influence of the C-terminus on the nSH2-cSH2 junction. The crosspeaks of residues Leu761, Lys763, Gly765, Glu776, Gly777, and Asn787 in the extended C-terminus for CSH2 as well as for E664G-NSH2CSH2 and E664K-NSH2CSH2 are often not coincident with those for NSH2CSH2 but instead are located on the vectors connecting those for NSH2CSH2 and NSH2CSH2-pY^783^ (Fig. 4 *b*; Document S1. Supporting Materials and Methods, Supporting Results, Figs. S1–S15, and Data S1 and S2, Figure360: An Author Presentation of Fig. 7 *a*), suggesting that the pre-C-terminus and the C-terminus populate the “closed” form more than in NSH2CSH2. Mutations within the nSH2-cSH2 junction, which in effect communicates the presence of the nSH2 domain, or removal of the nSH2 domain results in a stronger association of the pre-C-terminus and C-terminal linker with the cSH2 domain. As well as these C-terminal residues, Ala690 in the globular part of the cSH2 domain and in spatial proximity of the C-terminus displays a crosspeak in NSH2CSH2 at a position corresponding to dynamic fast exchange between “open” and “closed” states (Fig. 4 *b*), whereas the equivalent crosspeak for the CSH2 construct is shifted toward position in the “closed” NSH2CSH2-pY^783^. This difference suggests that the attached nSH2 domain influences not only the extended C-terminus but also the cSH2 residues with which the C-terminus interacts. MD simulations and NMR reveal an unstable contact between Asn757-Glu759 and the nSH2-cSH2 junction in NSH2CSH2-pY^783^, which is further loosened in NSH2CSH2 by virtue of contact between the nSH2-cSH2 junction and the pre-C-terminus (details in Supporting Materials and Methods, Section 2.2).

Overall, the picture is that the extended C-terminus and the nSH2 domain represent countervailing influences on the chemical shifts of cSH2 crosspeaks, consistent with two-way dynamic allosteric communication between the pY783-binding site and the nSH2-cSH2 junction. In energetic terms, the effects must be weak because the “open”-“closed” population balances for different residues vary only within a small range, but this is sufficient to lead to distinct influences on the chemical shifts that are directionally coherent (i.e., consistently toward either the “open” or “closed” states).

### Relevance of the dynamic C-terminal-linker-dependent pathway for allosteric communication

The foregoing experimental observations support the concept that binding of the Tyr783 region to the cSH2 domain influences the nSH2-cSH2 junction in a C-terminal-linker-dependent manner, in line with expectations from the MD simulations described above. Therefore, crosspeak positions that differ for particular residues in NSH2CSH2^*Δ*CT^ and NSH2CSH2^*Δ*CT^-CTpY must indicate a C-terminal-linker-“independent” impact of Tyr783 phosphorylation. As outlined in Supporting Materials and Methods, Section 2.6, and Fig. S7, we identified residues that are remote from the pY783 peptide binding site but influenced by pY783 binding even in the absence of the C-terminal linker, constituting a C-terminal-linker-“independent” pathway. Mapping the impact on both C-terminal-linker-dependent and C-terminal-linker-“independent” pathways on the cSH2 structure (Fig. 5 *b*) suggests that the former pathway is mostly dominant, other than in the pY783 peptide binding site.

Structural and dynamic interactions between the cSH2 *α*_B_-helix and the nSH2-cSH2 junction that were predicted in the MD simulations are supported by the NMR data, as discussed in Supporting Materials and Methods, Section 2.2, and Fig. S8. In this context, Glu742 emerges as a particularly notable residue. In the crystal structure, this residue is located next to the cSH2 domain *α*_B_-helix, adjacent to the nSH2-cSH2 junction (Document S1. Supporting Materials and Methods, Supporting Results, Figs. S1–S15, and Data S1 and S2, Figure360: An Author Presentation of Fig. 7 *c*). Glu742 shows a fast-exchange type peak pattern that indicates that it is part of the dynamic C-terminal-linker-dependent allosteric pathway. In addition, we found evidence that pY783 peptide binding to NSHCSH2^*Δ*CT^ also modulates the dynamic equilibrium at this residue. Specifically, C-terminal-linker-dependent and -independent Tyr783 binding have opposing effects on the Glu742 chemical shift (Fig. 5 *a*). A more detailed chemical shift and MD analysis results suggest that the nearby nSH2-cSH2 junction structure is modulated in a different manner in each case (more information can be found in Supporting Materials and Methods, Section 2.5). The relevance of the dynamic C-terminal linker-dependent allosteric pathway in the framework of overall allostery is corroborated by the MD simulations, the dominant influence on sites that are spatially distant from Tyr783, and the specific impact of this pathway on the dynamics of the cSH2 domain and the C-terminus. Importantly, the disease-relevant Arg687Trp substitution has a specific impact on the C-terminal-linker-dependent allosteric pathway, as discussed below.

### Arg687Trp substitution modulates the dynamic allosteric pathway

The single site activating mutation Arg665Trp in PLC*γ*2 is known to occur in the context of ibrutinib resistance in chronic lymphocytic leukemia (4, 5, 15). Although this was a phenomenon was associated with influence on Rac-induced activation of the enzyme (21), we have found that when the Arg665Trp mutant of PLC*γ*2, and separately the equivalent Arg687Trp mutant of PLC*γ*1, is expressed in COS-7 cells, stimulation with epidermal growth factor (EGF) leads to significant loss of autoinhibition of phospholipase activity (Fig. S14). Although the basal level of activity (i.e., without EGF) is only moderately increased compared to WT, both mutants exhibited a higher level of activity under EGF receptor stimulation, suggesting that the mutants are more readily activated than the WT equivalents. For experimental studies, we have found it difficult to obtain ^15^N-labeled PLC*γ*2 constructs for NMR studies. However, the PLC*γ*1 Arg687Trp mutant is tractable.

As a prelude to NMR investigations of the mutant, we performed three MD simulations each 100 ns of R687W-NSHCSH2 and R687W-NSH2CSH2-pY^783^ for comparison with the respective WT trajectories. In the 3D structure, Arg687 is located on the loop connecting cSH2 helix *α*_A_ and the first strand of the central *β*-sheet in close proximity to the pre-C-terminus. The simulations suggest that the Arg687Trp substitution reduces the allosteric communication described above for the WT (Figs. S1, S9, and S10; Supporting Materials and Methods, Section 2.8). This disruption appears to be linked to a change of the structure or dynamics of the C-terminus.

**Figure 6:**
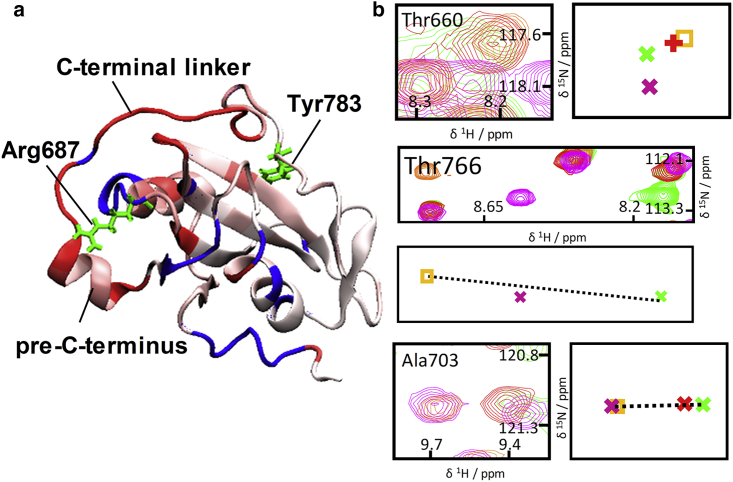
NMR data indicating a specific effect of the Arg687Trp mutation on the C-terminal-linker-dependent pathway. (*a*) Depiction of the cSH2 domain is colored to illustrate the degree of chemical shift perturbation for R687W-NSH2CSH2-pY^783^ relative to NSH2CSH2-pY^783^ mutants: white (no perturbation) to red (strong perturbation); blue, insufficient data. (*b*) Superposition of ^1^H,^15^N-HSQC spectra recorded at 600 MHz and corresponding schematic representations of crosspeak centroids are shown (*color scheme* as in Fig. 3). The Arg687Trp mutation leads to a perturbation for junction residue Thr660. The crosspeaks for residues such as Ala703 that are associated with the C-terminal-linker-independent allosteric pathway are barely shifted. Larger CSPs are observed for residues exemplified by Thr766 that are associated with the C-terminal-linker-dependent pathway. Additional examples are shown in Fig. S11.

**Figure 7:**
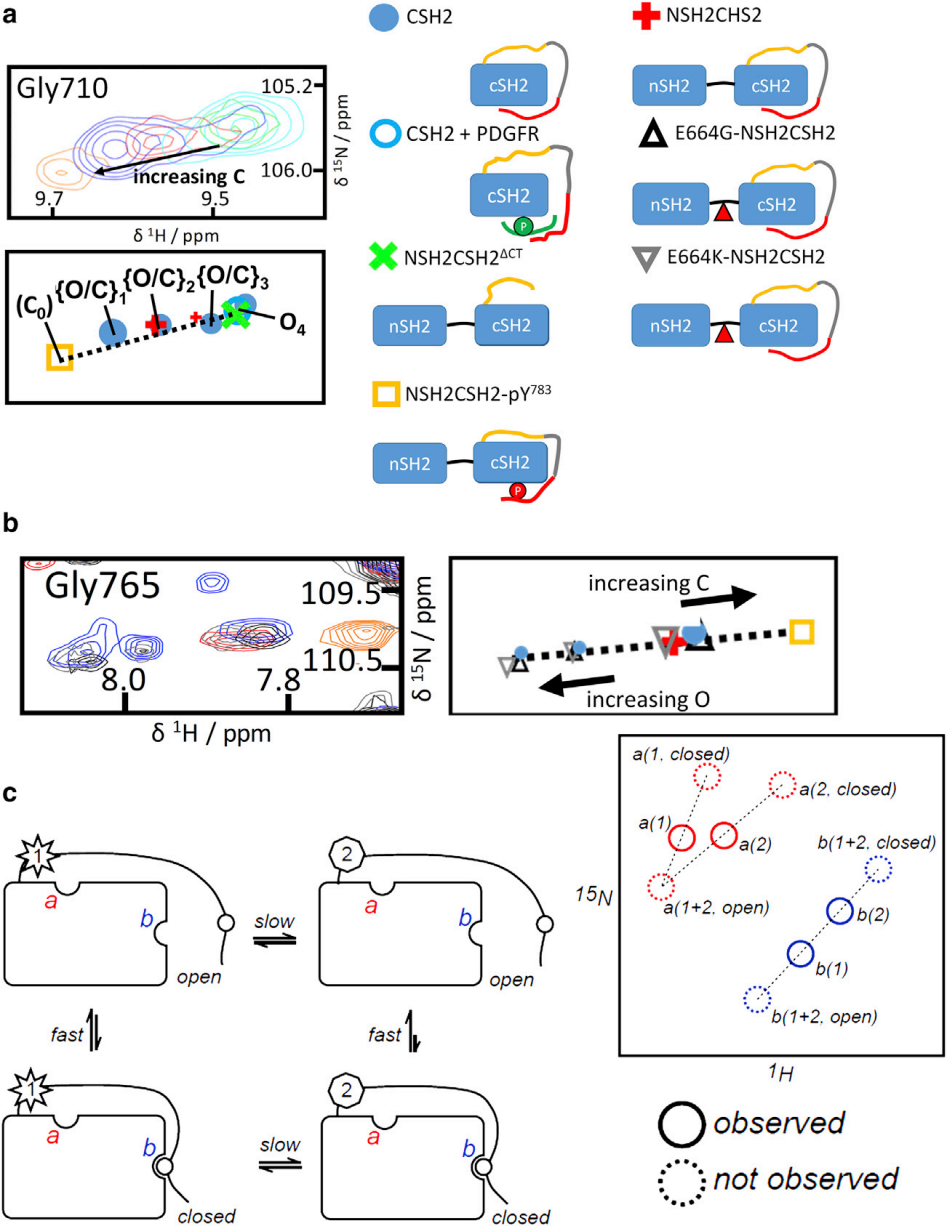
For a Figure360 author presentation of Fig. 7 *c*, see the figure legend at https://doi.org/10.1016/j.bpj.2018.05.031#mmc2. Evidence for multistate fast and slow exchange in CSH2 and NSH2CSH2 constructs. (*a*) CSH2 crosspeaks observed for residue Gly710 coincide with the peak positions of some other constructs (the *color* scheme is similar to that employed in Fig. 3, extended to include the complex of CSH2 with the platelet-derived growth factor receptor (PDGFR) phosphopeptide). The crosspeak label C_0_ indicates the NSH2CSH2-pY^783^ bound reference state that is not observed in CSH2. Four CSH2 states in slow exchange are identified: {O/C}_1_, {O/C}_2_, {O/C}_3_, and O (see main text). These states can also be observed over the course of a titration of CSH2 with PDGFR phosphopeptide (titration and further examples are shown in Fig. S15). The titration endpoint (CSH2 fully bound to the PDGFR phosphopeptide) is shown here. (*b*) Superposition of ^1^H,^15^N-HSQC spectra (700 MHz) indicates slow exchange for CSH2 and junction mutant constructs, exemplified by residue Gly765; color scheme as in (*a*). (*c*) Consideration of the effects of fast exchange in combination with “local” versus “remote” slow exchange on crosspeak patterns; see Figure360 for an extended animated version of this scheme. Top: in the scheme, the origin of slow exchange between states “1” and “2” (*left* and *right*, respectively) is illustrated conceptually by star and heptagon symbols. Consider now additional fast exchange (between *top* and *bottom* structures), for example corresponding to the association of an otherwise flexible appendage with the protein core. The rate of fast exchange depends on whether the molecule is in state “1” or “2.” We can now probe two sites *a* and *b* at which the combined exchange phenomena might be observed; the corresponding schematic spectra for both sites are shown on the right of the bottom panel. For site *a*, the origin of the slow exchange is “local”; for site *b*, it is “remote.” Note that in this study, the position of the fully “closed” position is known from experiments with NSH2CSH2-pY^783^. In the “local” slow case *a*, we would not expect crosspeak(s) to sit on a single vector between “open” and “closed” state coordinates. The observations described in this article correspond to the scenario depicted for site *b*, i.e., the crosspeak patterns that we have analyzed arise from residues apparently “remote” from the source of slow exchange. Figure360: An Author Presentation of Fig. 7

We prepared Arg687Trp tandem SH2 constructs to experimentally probe the impact on structure, dynamics, and the dynamic allostery. The pre-C-terminus Gly765 crosspeak for the Arg687Trp mutant construct is located well away from the fast-exchange “open”-“closed” vector, indicating CSPs dominated by the local effect of the side-chain substitution over other influences. The overall pattern suggests that Arg687 could be in direct contact with residues in the C-terminus. However, CSPs between NSH2CSH2-pY^783^ and R687W-NSH2CSH2-pY^783^ constructs are not confined to the immediate locale of residue Arg687 (Fig. 6 *a*). The Arg687Trp change likely impacts the nSH2-cSH2 junction itself, as suggested by the large CSPs for Thr660 (Fig. 6 *b*). Significant CSPs were observed for several residues in the putative contact regions between the extended C-terminus and the cSH2 domain, for example Ala690, Gly710, Gly758, Ala770, Leu761, Gly772, Ala773, and Thr766, as well as for the *α*_B_-helix residue Glu742 (Fig. 6 *b*; Document S1. Supporting Materials and Methods, Supporting Results, Figs. S1–S15, and Data S1 and S2, Figure360: An Author Presentation of Fig. 7 *c*). Data for R687W-NSH2CSH2 indicate a similar trend (Fig. S11 *b*). The data reveal that the pre-C-terminus and C-terminal linker populate the “closed” state to a lesser degree in Arg687Trp constructs than in the WT, suggesting that the Arg687Trp substitution modulates the C-terminal-linker-dependent allosteric pathway.

It is noteworthy that there are no major CSPs for residues belonging exclusively or even mostly to the C-terminal-linker-independent pathway upon nSH2 domain removal or upon Arg687Trp mutation; namely, Leu692, Lys695, Ala703, Ile704, Glu720, Val724, Gly727, Asn728, and Ser733 have the same or very similar chemical shifts in the mutant (Fig. S11 *d*). The Arg687Trp mutation selectively perturbs the dynamic C-terminal-linker-dependent pathway and exerts its functional influence either by weakening the association of the C-terminus with the cSH2 domain or by impact upon the nSH2-cSH2 junction.

### Structural origin and dynamic complexity of the C-terminal-linker-dependent pathway

Returning to the WT proteins, in addition to the fast-exchange phenomena described above, peak doubling or broadening was also observed in the spectra. Residues for which peak doubling, and even tripling or more, was evident are located in the extended C-terminus or in regions of the cSH2 domain predicted to be in close proximity, for example Gly689, Ala690, Gly710, Gly727, Gly765, Thr766, and Gly777 (Figs. 3 *a*, 4 *b*, and 7; Figs. S12 and S15). Notably, the peak doubling was most readily discerned in the spectrum of CSH2, presumably because of the overall lower number of residues (∼crosspeaks) and narrower linewidths on account of the smaller molecular mass. However, additional minor populations in NSH2CSH2 were revealed upon mutation of nSH2-cSH2 junction residue Glu664. In multiple instances, the resulting peak patterns resemble those found for CSH2 (Figs. 7 *b* and S7 *a*), consistent with the notion that the junction plays a role in relaying the presence of the nSH2 domain to the tandem construct C-terminus.

The peak doubling pattern was observed consistently over several independently prepared samples and therefore cannot be attributed to chemical heterogeneity and must arise because of slow exchange between different states. As a result, the relative intensity of the crosspeaks is defined by the respective state populations. In the cases in which (only) two CSH2 crosspeaks are observed, one peak is frequently located at the position of, or very close to, the corresponding peak for NSH2CSH2^*Δ*CT^, whereas the other sits on the vector connecting the crosspeaks for NSH2CSH2^*Δ*CT^ and NSH2CSH2-pY^783^. The observed crosspeak pattern is consistent with the concept that, in addition to the major population already discussed, the second population of molecules is also undergoing the “open” ⇌ “closed” (fast) exchange discussed above for NSH2CSH2. However, these additional slowly exchanging states of the C-terminus and pre-C-terminus differentially influence the apparent (local) affinity of a given residue for the cSH2 domain. For the “simpler” case of CSH2 peak-doubling, we can envisage the following: in one slow-exchanging state (state “1”), the effective “open” → “closed” on-rate is *k*_on,1_, and the “closed” → “open” off-rate is *k*_off,1_; in the second slow-exchanging state “2,” at least one of the effective on-rates and/or off-rates *k*_on,2_, *k*_off,2_ is different. Whereas the rate for the “1” ⇌ “2” interconversion is slow, in each of states “1” and “2” (with populations p_1_ and p_2_), the exchange between “open” and “closed” is fast on the NMR timescale. For residue *i*, we will thus observe two effective binding constants, namely *K*^i^_1_ = *p*^i^_1,closed_/*p*^i^_1,open_ = *k*^i^_1,on_/*k*^i^_1,off_ and *K*^i^_2_ = *p*^i^_2,closed_/*p*^i^_2,open_ = *k*^i^_2,on_/*k*^i^_2,off_. In the absence of multiple slow-exchanging states, a single crosspeak would sit at a position along the vector joining the chemical shift coordinates for the “open” and “closed” forms. When the “1” ⇌ “2” exchange is slow, two crosspeaks would be expected, with intensities defined by the state populations *p*^i^_1_ and *p*^i^_2_. However, when both of the states X=“1,” “2” are each also in “open”_X_ ⇌ “closed”_X_ fast exchange, the crosspeaks are again positioned along the “open”-“closed” vector. The whole scenario can be encapsulated in the schema:

$${\left\{O\overset{*}{⇌}C\right\}}_{1}\overset{†}{⇌}{\left\{O\overset{*}{⇌}C\right\}}_{2},$$

where O and C denote “open” and “closed,” brackets encapsulate the states “1” and “2,” and ^∗^ and † indicate fast and slow exchange, respectively.

Thus, there are two populations of molecules, each demonstrating “open” ⇌ “closed” equilibria but spectroscopically separated by a “remote” slow-exchange phenomenon. “Remote” here is meant in the sense that the difference in molecular properties that gives rise to slow exchange does not influence the chemical shifts underlying the concomitant fast-exchange process but can influence the populations for the fast exchange in each of the two (slowly exchanging) states “1” and “2.” The general scenario can be conveniently depicted in cartoon form (Fig. 7 *c*, animated Figure360). Importantly, the pattern of crosspeaks is distinctly different from that expected for a crosspeak that originates from a location close to the site of slow exchange.

Our model for the dynamic origin of the detected crosspeak patterns is bolstered by our observations of crosspeak doubling in a titration of a platelet-derived growth factor receptor *β* (PDGFR*β*)-derived phosphopeptide with CSH2 (Fig. 7 *a*). As outlined in Supporting Materials and Methods, Section 2.9, and Fig. S15, we observe more than two slowly exchanging states over the course of the titration, which can be understood by invoking an extension of the combined fast/slow-exchange scheme:

$${\left\{O\overset{*}{⇌}C\right\}}_{1}\overset{†}{⇌}{\left\{O\overset{*}{⇌}C\right\}}_{2}\overset{†}{⇌}{\left\{O\overset{*}{⇌}C\right\}}_{3}\overset{†}{⇌}O:P,$$

where O:P indicates the protein:peptide complex. Importantly, we observe that over the course of the titration, the populations of the slow-exchanging “1,” “2,” and “3” states change because of the mass action of the peptide, which effectively captures the “open” state of the protein to form the O:P protein-peptide complex. When the peptide is in molar excess, essentially all of the protein is in the O:P state (see Supporting Materials and Methods, Section 2.9). Our interpretation of peak patterns as the result of a combination of slow and fast-exchange phenomena also rationalizes the peak tripling observed for residue Thr766 in the CSH2 construct (Fig. S12 *b*; Supporting Materials and Methods, Section 2.3).

Slow exchange due to purely conformational events is relatively rare in protein NMR. Given that the most common source of such slow events is the *cis* ⇌ *trans* isomerism of X-Pro peptide bonds (22, 23), often in disordered regions of polypeptides, we compared CSPs for WT constructs with variants in which specific proline residues, including two that reside in the C-terminal linker, were substituted with alanine (Document S1. Supporting Materials and Methods, Supporting Results, Figs. S1–S15, and Data S1 and S2, Figure360: An Author Presentation of Fig. 7 *a*; Supporting Materials and Methods, Section 2.4). Although none of the mutations completely quenched the presence of slow exchange, overall our observations strongly suggest that *cis*-/*trans*-peptide bond isomerization in the extended C-terminus leads to the complexity of the spectra of the cSH2-domain-containing proteins.

### Tyr783 phosphorylation and the Arg687Trp mutation in the context of the *γ*-specific array

**Figure 8:**
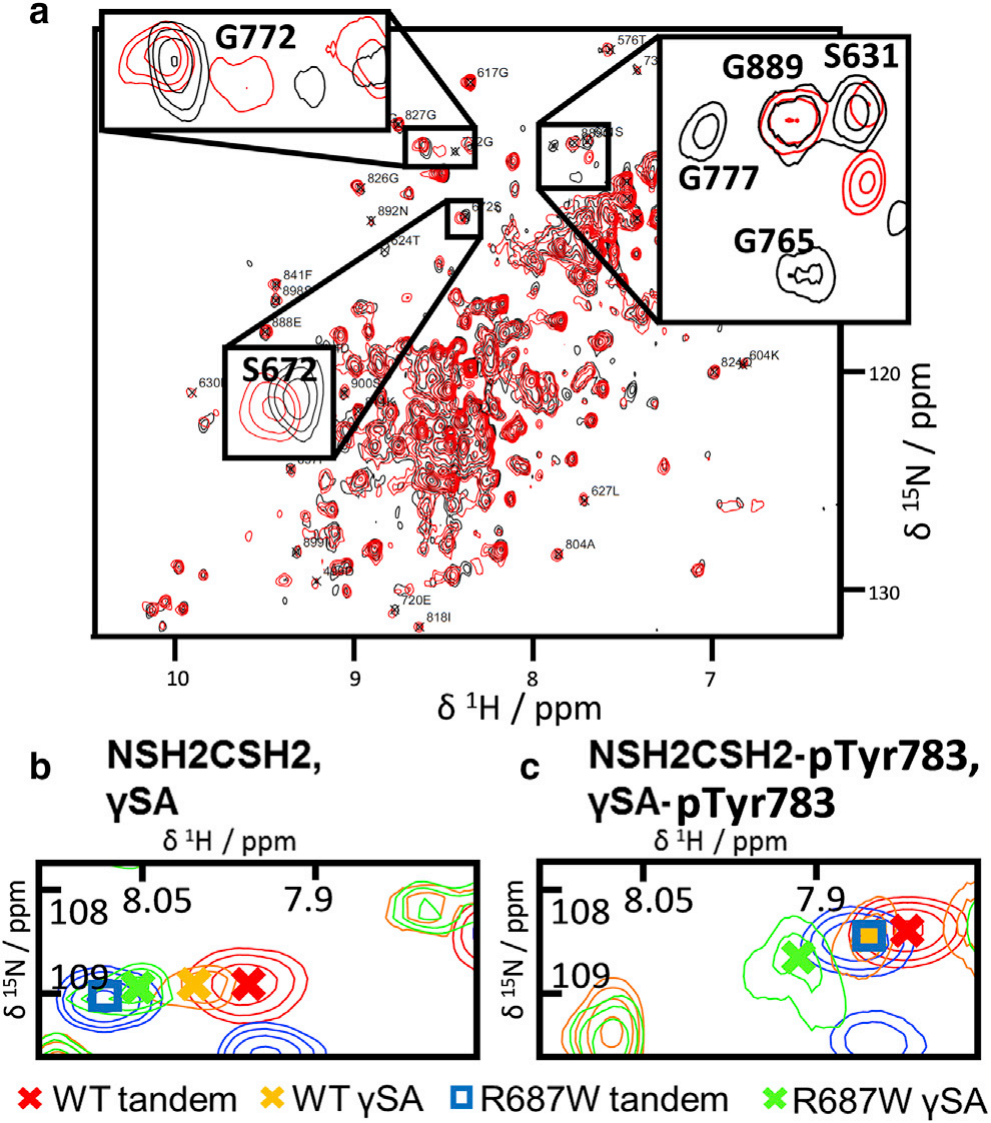
The NMR observations made in context of tandem- nSH2-cSH2 constructs are reflected in ^1^H,^15^N-NMR spectra for the *γ*-specific array (*γ*SA). (*a*) ^1^H,^15^N-SOFAST-HMQC spectra of *γ*SA (*red*) and *γ*SA-pY783 (*black*) are shown. Spectra were recorded at 950 MHz at 25°C. Further backbone crosspeak assignments for nonphosphorylated *γ*SA can be found elsewhere (10). (*b* and *c*) ^1^H,^15^N-SOFAST-HMQC crosspeaks of residue Gly777 in nonphosphorylated (*b*) and phosphorylated (*c*) tandem- or *γ*SA constructs are shown. Symbols depict the crosspeak centroids (*color* key depicted below). The “closed” state is less populated in nonphosphorylated and R687W mutant constructs than in the corresponding phosphorylated and WT constructs. Spectra were recorded at 34°C (*b*) or 25°C (*a* and *c*) and at 700 MHz (*b*) or 950 MHz (*a* and *c*).

To test whether the observations made for tandem SH2 proteins also apply in the context of the larger *γ*SA protein, both nonphospho- and phospho-constructs for WT and mutant versions of the *γ*SA were prepared. Many backbone crosspeaks in two-dimensional ^1^H,^15^N-HSQC- or HMQC-type spectra were assigned (Fig. 8; Fig. S13), mostly through superposition with NMR spectra for various subconstructs (Supporting Materials and Methods, Section 1.4).

For *γ*SA, the well-resolved crosspeak for C-terminal-linker residue Gly777, which has a relatively narrow line shape, is particularly suited for analysis (Fig. 8). The nonphospho-, and separately the phospho-, protein crosspeaks almost coincide for WT and Arg687Trp tandem SH2 and *γ*SA proteins but, because of their relative positions, indicate weaker association of the cSH2-SH3 linker (which corresponds to the C-terminus in the context of NSH2CSH2) in *γ*SA compared to the respective tandem nSH2-cSH2 proteins. However, the relative (phospho- vs. nonphospho-, WT versus mutant) CSPs within each construct group (*γ*SA and tandem SH2) are essentially identical, indicating that the underlying source of the shift difference is present in both the tandem and *γ*SA contexts. Further, CSP analysis for residues Ser672, Gly765, Gly772, and Gly777 (Fig. 8; Fig. S13) confirms that the “closed” population differences between nonphospho- and phospho-constructs and between WT and Arg687Trp constructs apply in the *γ*SA context.

## Discussion

We and others have demonstrated that phosphorylation of Tyr783 in the cSH2-SH3 linker of PLC*γ*1 has a critical regulatory role in the mechanism of activation of the enzyme (10). Here, MD simulations of PLC*γ*1 tandem SH2 constructs were analyzed to interrogate the potential impact of phosphorylation on NSH2CSH2 structure and dynamics. PCA suggested significant differences between correlated motions in NSH2CSH2 and NSH2CSH2-pY^783^. Specifically, differences were detected between the one-dimensional projections of NSH2CSH2 and NSH2CSH2-pY^783^-tandem trajectories on the eigenvector describing motions of the C-terminus and the pre-C-terminus. Contact probabilities for NSH2CSH2 and NSH2CSH2-pY^783^ differ for the following pairs: extended C-terminus/cSH2, extended C-terminus/nSH2-cSH2 junction, and nSH2-cSH2 junction/cSH2 *α*_B_-helix. Side-chain mutual information analysis, which can be used to detect correlations between allosteric sites (18), suggests the presence of an allosteric connection between the C-terminus and the nSH2-cSH2 junction. These observations spurred the experimental analysis of the set of tandem SH2 protein constructs by heteronuclear NMR, with the aim to validate the predictions.

Naïvely, Tyr783 phosphorylation would be expected to lead to binding of the Tyr783 peptide region to the phosphopeptide binding site on the cSH2 domain. In contrast, NMR data show that Tyr783 phosphorylation leads to changes in structure and dynamics in the cSH2 domain that are more extensive. Importantly, a substantial proportion of these changes depend on the presence of the C-terminal linker. The chemical shift patterns observed for many residues in different constructs can be rationalized by invoking fast averaging between “open” and “closed” states. Significantly, it is not possible to rationalize the fast-exchange kinetics in terms of a single binding equilibrium; rather, the data suggest that different parts of the C-terminus are more strongly associated with the surface of the cSH2 domain than others.

A previous study showed that the presence of the C-terminus modulates the chemical shifts of multiple cSH2 domain crosspeaks; therefore, the C-terminus was thought to be bound to the cSH2 domain even in the absence of Tyr783 phosphorylation (16). However, our observations obtained for a range of WT and mutant phospho- and nonphospho-constructs indicate that the C-terminus is only partially bound in the latter case. That the binding is not complete is relevant in the context of the supposed association of the cSH2 domain with the PLC*γ*1 enzyme core, leading to autoinhibition in the nonphospho-state (10) because a fully bound C-terminus would impede the molecular interaction of the cSH2 domain with the core. Moreover, it has been suggested that interactions of the cSH2 domain with two FGFR kinase domains in the supramolecular complex arising from FGF ligand-receptor association are part of the activation mechanism for PLC*γ*1 (12). Whether or not these interactions are involved in PLC*γ*1 activation, each requires that the cSH2 domain exhibit at least transient exposure of the requisite interaction surface. Because the interactions are expected to take place at the cSH2 pTyr-binding site, there would be a requirement that the cSH2-SH3 linker can be readily displaced from the binding site, which is in agreement with our findings for transient association in both tandem SH2 and *γ*SA proteins.

Tyr783 phosphorylation leads to CSPs for nSH2-cSH2 junction residue Thr660, which is at >20 Å distance, strongly suggesting an allosteric effect. A direct allosteric communication via the C-terminal linker can be postulated when including the “pre-C-terminus” region that is predicted by both crystallographic analysis and MD simulations to come into contact with the nSH2-cSH2 interdomain junction. The chemical shift patterns for many cSH2 residues indicate that Tyr783 phosphorylation affects the relative populations rather than the structures of the pre-existing “open” and “closed” states. Importantly, the fast-exchange “open” ⇌ “closed” equilibrium is modulated in various mutant constructs; for each construct, we observed a global tendency to shift residues toward either the “open” or “closed” state. A substantial part of the change triggered by Tyr783 phosphorylation can therefore be described as dynamic allostery (24, 25, 26).

The dynamic allosteric connection is attributed to the C-terminus and the pre-C-terminus of the tandem domain constructs; in particular, the nature of the allostery depends on the presence of the C-terminal linker. The additional observation that the populations of the local “open” and “closed” states differ for various C-terminal residues implies that the overall number of conformations is large. The high number of degrees of freedom suggests that entropic considerations could be particularly relevant in the allosteric communication of Tyr783 phosphorylation as well as in potential interactions of a binding partner with the C-terminus or the cSH2 domain (26). These intermolecular interactions could be controlled by dynamic interactions of the C-terminus with the cSH2 domain, a situation that would be an example of a “fuzzy” intramolecular complex (27).

Identification of the dynamic, C-terminal-linker-dependent allosteric effect of Tyr783 phosphorylation should improve the understanding of the effect of mutations in the cSH2 domain. We have studied the dynamic impact of the activating, disease-relevant Arg687Trp mutation by MD simulations and NMR. We find that within the context of the tandem nSH2-cSH2 and *γ*SA proteins, the Arg687Trp change shifts the extended C-terminus toward the “open” state and modulates the dynamic C-terminal-linker-dependent allosteric pathway. Distinct hypotheses emerge for how these dynamic and structural changes lead to the activating effect of the Arg687Trp substitution. Perturbation of the nSH2-cSH2 junction structure or stability could lead to an alteration in the relative orientation of or flexibility between the nSH2 and cSH2 domains; alternatively, the weakened interaction between the cSH2 domain and the extended C-terminus, which corresponds to the tether between the cSH2 with SH3 domains in the *γ*SA, might be key to the mutant behavior. The consequences of Arg687Trp could include changes in interactions of the *γ*SA with RTKs, with the enzyme core, or, in the case of Arg665Trp in PLC*γ*2, with Rac2.

**Figure 9:**
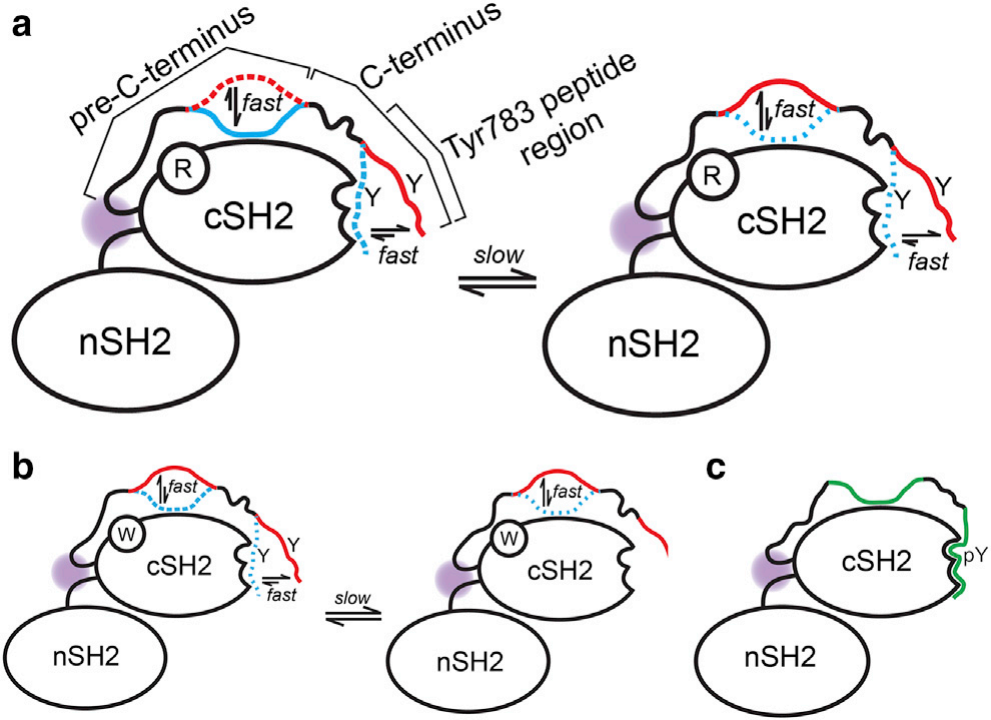
Scheme illustrating the interpretation of the combination of slow- and fast-exchange phenomena present in various tandem SH2 constructs. (*a*) NSH2CSH2, (*b*) R687W-NSH2CSH2, and (*c*) NSH2CSH2-pY^783^ are shown. The regional variation of kinetic equilibria throughout the extended C-terminus is indicated. Red and blue colors refer to parts of the extended C-terminus in fast-exchanging “open” and “closed” states, respectively. The relative populations of the different chain segments in the extended C-terminus are symbolized by colored solid (high), dotted (medium), or sparsely dotted (low) chain segments. (*a*) For NSH2CSH2, the pattern of crosspeaks suggests underlying fast-exchange processes attributable to fleeting contacts of the cSH2 domain with different segments of the extended C-terminus; additional peak doubling indicates slow exchange between states, potentially associated with *cis-/trans-*peptide bond isomerization. (*b*) For R687W-NSH2CSH2, a similar mixture of slow and fast exchange pertains, though the affinity of the cSH2 domain for the extended C-terminus appears weaker. (*c*) For NSH2CSH2-pY^783^, the strong interaction between the cSH2 pTyr783 binding site and the extreme pY783 peptide region at the C-terminus cooperates with the binding of other regions of the C-terminus and the pre-C-terminus (all depicted in *green color*) to the “body” of the cSH2 domain. In each case, long-range effects involving the interactions of the extended C-terminus were evident in the NMR characteristics of crosspeaks for residues located at the nSH2-cSH2 junction, indicated by purple shading.

Herein, we have presented a system in which dynamic allosteric communication is directly observable by monitoring ^1^H,^15^N-HSQC peak shifts, representing population shifts between fast-exchanging locally bound and unbound states. Our approach is related to the chemical shift projection analysis procedure (28) to reveal allosteric networks, which is distinct from chemical shift covariance analysis in that we focus mostly on population shifts of fast-exchanging residues, which helps avoid false positives (20). Our work also reveals the presence of a complex set of slow-exchange phenomena that combines with fast exchange and thus leads to unusual NMR crosspeak patterns, with two or more crosspeaks representing two fast-exchanging but differentially populated states for a single residue. In particular, we rationalize the spectral characteristics in terms of “remote” slow-exchange equilibria that determine the population of “locally” fast-exchanging “open” ⇌ “closed” populations (Fig. 7). The system is conceptually similar to the population shuffling of rotamer conformations that was recently described for protein G and ubiquitin (29), though it remains to be investigated whether the underlying mechanistic processes in PLC*γ* are mutually independent. A schematic that aims to capture the different exchange processes at play in this system is presented in Fig. 9. We emphasize that the apparent complexity of the tandem nSH2-cSH2 system described herein emerged only by examination of a relatively large number of protein variants. It is possible that other unrelated systems could also encompass such multitimescale dynamic allostery that may go undetected in the absence of appropriate variant constructs. We note that the PLC*γ*1 system described here should lend itself to quantitative exploration of the *N*-state kinetics that underlies dynamic allosteric communication using state-of-the-art methods in NMR relaxation experiments over a wide range of timescales (30, 31).

## Conclusions

This work has highlighted a hitherto unappreciated and complex pattern of allosteric influences within the receptor adaptor domains of PLC*γ*1 that are likely relevant for experiments aimed at elucidating its regulation in both space and time at any greater length scale (e.g., with full-length proteins in a whole cell context). MD simulations suggested structural connectivity between occupation of the canonical cSH2 phosphopeptide binding site and the nSH2-cSH2 interdomain junction. As part of the experimental validation of the key MD predictions, we uncovered a surprisingly complex set of NMR characteristics for the tandem SH2 domain protein. The MD simulations also provided an invaluable context within which to effectively design and assess tandem SH2 domain variants to decipher the emergent NMR characteristics of allostery in the cSH2 domain. We have shown that the C-terminus is partially bound in the nonphosphorylated state and that presence of the nSH2 domain changes the dynamic properties of the cSH2 domain. The comparison of multiple protein variants revealed evidence for allosteric connectivity that importantly involves the polypeptide tether between the globular part of the cSH2 domain and the Tyr783 phosphorylation site. The allosteric connection is dynamic in nature and involves the presence of multiple combined fast- and slow-exchange processes. We have demonstrated that the disease-relevant mutation Arg687Trp weakens the dynamic allosteric connection (Fig. 9), provoking specific hypotheses about the mechanistic basis for the effect of this substitution; it remains to be determined which particular section of the dynamic allosteric arc spanning the long extended C-terminus and the nSH2-cSH2 junction is functionally relevant in context of holo-PLC*γ*1. Importantly, the modulated binding of the extended C-terminus in Arg687Trp mutants was also detected in the context of the larger *γ*SA fragment of PLC*γ*1, indicating that the impact of the dynamic modulation extends beyond tandem SH2 constructs and should be taken into account in any examination of the upstream regulation of PLC*γ* proteins by RTKs and small G-proteins.

## Author Contributions

H.K., M.K., and P.C.D. designed the research. H.K. performed and analyzed the MD simulations. H.K. and T.D.B. performed the mutagenesis experiments. H.K. and T.D.B. expressed and purified proteins. H.K. and D.E. performed the NMR experiments. H.K. analyzed the NMR data. M.M. conducted the enzyme activity measurements. H.K. and P.C.D. wrote the article.

## Supporting Citations

References (32, 33, 34, 35, 36, 37, 38, 39, 40, 41, 42, 43, 44, 45, 46, 47, 48, 49, 50, 51, 52) appear in the Supporting Material.

## Supporting Material

Document S1. Supporting Materials and Methods, Supporting Results, Figs. S1–S15, and Data S1 and S2

Document S2. Article plus Supporting Material

## References

[1] Bunney T.D., Katan M. (2011). PLC regulation: emerging pictures for molecular mechanisms. Trends Biochem. Sci.

[2] Suh P.G., Park J.I., Ryu S.H. (2008). Multiple roles of phosphoinositide-specific phospholipase C isozymes. BMB Rep.

[3] Rebecchi M.J., Pentyala S.N. (2000). Structure, function, and control of phosphoinositide-specific phospholipase C. Physiol. Rev.

[4] Koss H., Bunney T.D., Katan M. (2014). Dysfunction of phospholipase Cγ in immune disorders and cancer. Trends Biochem. Sci.

[5] Walliser C., Retlich M., Bunney T.D. (2008). rac regulates its effector phospholipase Cgamma2 through interaction with a split pleckstrin homology domain. J. Biol. Chem.

[6] Bae J.H., Lew E.D., Schlessinger J. (2009). The selectivity of receptor tyrosine kinase signaling is controlled by a secondary SH2 domain binding site. Cell.

[7] Poulin B., Sekiya F., Rhee S.G. (2005). Intramolecular interaction between phosphorylated tyrosine-783 and the C-terminal Src homology 2 domain activates phospholipase C-gamma1. Proc. Natl. Acad. Sci. USA.

[8] Gresset A., Hicks S.N., Sondek J. (2010). Mechanism of phosphorylation-induced activation of phospholipase C-gamma isozymes. J. Biol. Chem.

[9] DeBell K., Graham L., Rellahan B. (2007). Intramolecular regulation of phospholipase C-gamma1 by its C-terminal Src homology 2 domain. Mol. Cell. Biol.

[10] Bunney T.D., Esposito D., Katan M. (2012). Structural and functional integration of the PLCγ interaction domains critical for regulatory mechanisms and signaling deregulation. Structure.

[11] Hajicek N., Charpentier T.H., Sondek J. (2013). Autoinhibition and phosphorylation-induced activation of phospholipase C-γ isozymes. Biochemistry.

[12] Huang Z., Marsiglia W.M., Mohammadi M. (2016). Two FGF receptor kinase molecules act in concert to recruit and transphosphorylate phospholipase Cγ. Mol. Cell.

[13] Zhou Q., Lee G.S., Aksentijevich I. (2012). A hypermorphic missense mutation in PLCG2, encoding phospholipase Cγ2, causes a dominantly inherited autoinflammatory disease with immunodeficiency. Am. J. Hum. Genet.

[14] Behjati S., Tarpey P.S., Campbell P.J. (2014). Recurrent PTPRB and PLCG1 mutations in angiosarcoma. Nat. Genet.

[15] Woyach J.A., Furman R.R., Byrd J.C. (2014). Resistance mechanisms for the Bruton’s tyrosine kinase inhibitor ibrutinib. N. Engl. J. Med.

[16] Devkota S., Joseph R.E., Andreotti A.H. (2015). Scaffold protein SLP-76 primes PLCγ1 for activation by ITK-mediated phosphorylation. J. Mol. Biol.

[17] Eck M.J., Pluskey S., Shoelson S.E. (1996). Spatial constraints on the recognition of phosphoproteins by the tandem SH2 domains of the phosphatase SH-PTP2. Nature.

[18] McClendon C.L., Friedland G., Jacobson M.P. (2009). Quantifying correlations between allosteric sites in thermodynamic ensembles. J. Chem. Theory Comput.

[19] Siegal G., Davis B., Driscoll P.C. (1998). Solution structure of the C-terminal SH2 domain of the p85 alpha regulatory subunit of phosphoinositide 3-kinase. J. Mol. Biol.

[20] Boulton S., Akimoto M., Melacini G. (2014). A tool set to map allosteric networks through the NMR chemical shift covariance analysis. Sci. Rep.

[21] Walliser C., Hermkes E., Gierschik P. (2016). The phospholipase Cγ2 mutants R665W and L845F identified in ibrutinib-resistant chronic lymphocytic leukemia patients are hypersensitive to the Rho GTPase Rac2 protein. J. Biol. Chem.

[22] Alderson T.R., Lee J.H., Bax A. (2018). Propensity for cis-proline formation in unfolded proteins. ChemBioChem.

[23] Wüthrich K. (1976). NMR in Biological Research: Peptides and Proteins.

[24] Tzeng S.R., Kalodimos C.G. (2011). Protein dynamics and allostery: an NMR view. Curr. Opin. Struct. Biol.

[25] Cooper A., Dryden D.T. (1984). Allostery without conformational change. A plausible model. Eur. Biophys. J.

[26] Wand A.J. (2001). Dynamic activation of protein function: a view emerging from NMR spectroscopy. Nat. Struct. Biol.

[27] Fuxreiter M., Tompa P. (2012). Fuzzy complexes: a more stochastic view of protein function. Adv. Exp. Med. Biol.

[28] Selvaratnam R., VanSchouwen B., Melacini G. (2012). The projection analysis of NMR chemical shifts reveals extended EPAC autoinhibition determinants. Biophys. J.

[29] Smith C.A., Ban D., Lee D. (2015). Population shuffling of protein conformations. Angew. Chem. Int. Ed. Engl..

[30] Palmer A.G., Kroenke C.D., Loria J.P. (2001). Nuclear magnetic resonance methods for quantifying microsecond-to-millisecond motions in biological macromolecules. Methods Enzymol.

[31] Koss H., Rance M., Palmer A.G. (2017). General expressions for R_1ρ_ relaxation for N-site chemical exchange and the special case of linear chains. J. Magn. Reson.

[32] Murray V., Huang Y., Li Q. (2012). A novel bacterial expression method with optimized parameters for very high yield production of triple-labeled proteins. Methods Mol. Biol.

[33] Hess B., Kutzner C., Lindahl E. (2008). GROMACS 4: algorithms for highly efficient, load-balanced, and scalable molecular simulation. J. Chem. Theory Comput.

[34] Humphrey W., Dalke A., Schulten K. (1996). VMD: visual molecular dynamics. J. Mol. Graph.

[35] Schwieters C.D., Kuszewski J.J., Clore G.M. (2003). The Xplor-NIH NMR molecular structure determination package. J. Magn. Reson.

[36] Lindorff-Larsen K., Piana S., Shaw D.E. (2010). Improved side-chain torsion potentials for the Amber ff99SB protein force field. Proteins.

[37] Homeyer N., Horn A.H., Sticht H. (2006). AMBER force-field parameters for phosphorylated amino acids in different protonation states: phosphoserine, phosphothreonine, phosphotyrosine, and phosphohistidine. J. Mol. Model.

[38] Mobley D.L., Chodera J.D., Dill K.A. (2006). On the use of orientational restraints and symmetry corrections in alchemical free energy calculations. J. Chem. Phys.

[39] Schulte-Herbrüggen T., Sorensen O.W. (2000). Clean TROSY: compensation for relaxation-induced artifacts. J. Magn. Reson.

[40] Eletsky A., Kienhöfer A., Pervushin K. (2001). TROSY NMR with partially deuterated proteins. J. Biomol. NMR.

[41] Hyberts S.G., Arthanari H., Wagner G. (2012). Applications of non-uniform sampling and processing. Top. Curr. Chem.

[42] Kazimierczuk K., Orekhov V.Y. (2011). Accelerated NMR spectroscopy by using compressed sensing. Angew. Chem. Int. Ed. Engl.

[43] Kay L.E., Torchia D.A., Bax A. (1989). Backbone dynamics of proteins as studied by 15N inverse detected heteronuclear NMR spectroscopy: application to staphylococcal nuclease. Biochemistry.

[44] Delaglio F., Grzesiek S., Bax A. (1995). NMRPipe: a multidimensional spectral processing system based on UNIX pipes. J. Biomol. NMR.

[45] Orekhov V.Y., Jaravine V.A. (2011). Analysis of non-uniformly sampled spectra with multi-dimensional decomposition. Prog. Nucl. Magn. Reson. Spectrosc.

[46] Vranken W.F., Boucher W., Laue E.D. (2005). The CCPN data model for NMR spectroscopy: development of a software pipeline. Proteins.

[47] Williamson M.P. (2013). Using chemical shift perturbation to characterise ligand binding. Prog. Nucl. Magn. Reson. Spectrosc.

[48] Everett K.L., Bunney T.D., Katan M. (2009). Characterization of phospholipase C gamma enzymes with gain-of-function mutations. J. Biol. Chem.

[49] Svergun D.I., Koch M.H. (2002). Advances in structure analysis using small-angle scattering in solution. Curr. Opin. Struct. Biol.

[50] Bernadó P., Mylonas E., Svergun D.I. (2007). Structural characterization of flexible proteins using small-angle X-ray scattering. J. Am. Chem. Soc.

[51] Rossi P., Swapna G.V., Montelione G.T. (2010). A microscale protein NMR sample screening pipeline. J. Biomol. NMR.

[52] Pascal S.M., Singer A.U., Forman-Kay J.D. (1994). Nuclear magnetic resonance structure of an SH2 domain of phospholipase C-gamma 1 complexed with a high affinity binding peptide. Cell.

