# Supplementary material for: Dynamic Allostery in PLCγ1 and Its Modulation by a Cancer Mutation Revealed by MD Simulation and NMR
Source: Biophys J. 2018 Jul 3;115(1):31–45. doi: 10.1016/j.bpj.2018.05.031 (PMC6035297; doi:10.1016/j.bpj.2018.05.031)
Supplement: Document S1. Supporting Materials and Methods, Supporting Results, Figs. S1–S15, and Data S1 and S2 [file mmc1.pdf]

Biophysical Journal, Volume *115*

Supplemental Information

Dynamic Allostery in PLCγ1 and Its Modulation by a Cancer Mutation Revealed by MD Simulation and NMR

Hans Koss, Tom D. Bunney, Diego Esposito, Marta Martins, Matilda Katan, and Paul C. Driscoll

S1. Materials and Methods

S1.1 Cloning, mutagenesis, protein expression and purification

PLCγ1 constructs used in this work are all human and generally based on the NCBI sequence NP_002651.2. All PLCγ1 constructs were expressed from pOPINS (Oxford Protein Production Facility) plasmids using *E. coli* strain C41 (DE3) (Lucigen). For a definition of construct boundaries, see first paragraph in the results section and *Fig. 1b*. Protein constructs include an N-terminal 6xHis-SUMO tag. For improved comparability with NSH2CSH2-pY^{783}, NSH2CSH2 "WT" constructs carry Tyr771,775Phe mutations, which do not change any relevant peak positions in ^{1}H,^{15}N-HSQC spectra. The plasmids for NSH2CSH2 (545-790), NSH2CSH2$^{\Delta CT}$ (545-770), γSA WT (488-933), γSA Tyr771Phe (488-933) and all cSH2 constructs (663-790) were part of the lab inventory and created in context of a previous study (Bunney et al 2012).

Mutagenesis reactions were performed using the Thermo Scientific Pfu DNA Polymerase kit to create constructs with Arg687Trp or/and Tyr771,775Phe mutations. Per 25 µl reaction, 62.5 ng of both forward and reverse oligonucleotides were added; Tyr771,775Phe mutants were created in a one-step mutagenesis reaction.

Oligonucleotides for mutagenesis reactions were ordered from Invitrogen. Forward sequences are:
R687W (PLCγ1): CTAATGCGCGTCCCTTGGGATGGGGCCTTCCTGG
R665W (PLCγ2): GATGAGGATTCCCTGGGACGGGGCCTTCC
E664G (PLCγ1): CAGACCAACGCCCACGGGAGCAAAGAGTGG
E664K (PLCγ1): CAGACCAACGCCCACAAGAGCAAAGAGTGG
Y771,775F (PLCγ1): GACTTCGGGGCCCTGTTTGAGGGACGCAACCC).

For sequencing of PLCγ constructs, a T7 forward primer, a set of internal forward primers and a SUMO-tag primer were used. For the construction of other plasmids refer to Bunney et al. (10).

The following procedure was used to produce ^{15}N or ^{13}C,^{15}N-labelled protein. Liquid cultures were generally shaken at 200 rpm and baffled flasks were used for expression. Expression yields were improved by taking into account suggestions from Murray et al. (32). The pOPINS vector harboring the construct was transformed into E. coli strain C41 (DE3). Eight colonies were picked and incubated to 37 °C for 9 hours in a 2 l flask with 500 ml 2xYT/Kana, then pelleted and resuspended in a 2l flask with 500 ml minimal medium (90 mM Na_2HPO_4, 22 mM KH_2PO_4, 8.5 mM NaCl, 7.5 mM ^{15}N-$(NH_4)_2SO_4$ 56 mM D-Glucose, 0.5 x Trace Metal Mix, 50 µg/ml Kanamycin, 20 mg/l Thiamin, 20 mg/l Biotin, 2 mM $MgCl_2$, pH 7.4). Glucose concentration was set to 11 mM for ^{13}C-labelled proteins (substituting regular glucose with D-Glucose-$^{13}C_6$). The flask was kept at 37 °C for one hour and then cooled to 20 °C for one hour. Protein expression was induced with 100 µM IPTG and expressed at 20 °C for 10 hours. Bacteria were pelleted and kept frozen at -80 °C. For expression in 100% D_2O, the

culture was pre-grown in a small volume of 100% D_2O prior to expression; expression time was 20 hours.

Frozen pellets from a 500 ml – 2000 ml culture were used in the purification procedure; larger cultures up to 6 l were split accordingly, and pooled after His tag cleavage. All steps were performed at 4 °C. 15 ml (1000 ml culture: 30 ml) lysis buffer (25 mM TrisCl, pH 8.0, 250 mM NaCl, 40 mM imidazole, 10 mM benzamidine, 1 mM $MgCl_2$, 10 μM $CaCl_2$, lysozyme) was added to frozen pellets in large-diameter centrifugation vessels (500 ml or 1000 ml culture). The vessels were placed on a shaker (200 rpm) for 30 minutes to 1 hour. 3.5 ml (1000 ml culture: 7.5 ml) 10% (v/v) Triton X-100 (needs about one hour to dissolve) and 75 μl (1000 ml culture: 150 μl) Dnase I (bovine pancrease) from glycerated solution were added. The vessels were shaken for another hour and then centrifuged for one hour (Beckmann Coulter Avanti K-20XP, JS-25.50 rotor, 12000 rpm). The supernatant was used for further purification using the AKTA Explorer or Purifier system (GE Healthcare). A HisTrap (GE Healthcare, individual columns for distinct proteins) column and His buffers A (25 mM TrisCl, pH 8.0, 500 mM NaCl, 40 mM imidazole, 1 mM TCEP) and B (25 mM TrisCl, pH=8.0, 500 mM NaCl, 500 mM imidazole, 1 mM TCEP) were used for the first purification step (10 column volumes of His buffer A, then 5 column volumes linear gradient to His buffer B). The His-SUMO tag of collected sample was then cleaved by adding 100 μl of 5 mg/ml Ulp1 protease and dialyzing (MW 10 kDa tubing) against His Chelating Buffer (25 mM TrisCl, pH=8.0, 250 mM NaCl, 20 mM imidazole, 1 mM TCEP) overnight (at least 14 hours). For the second purification step a HisTrap Chelating column (GE Healthcare, the same column for all proteins) was used (same conditions as for the first purification step, but use filtered Chelating buffer instead of His buffer A). The collected sample was dialyzed (MW 10 kDa tubing) against Low Salt buffer for at least 4 hours (25 mM TrisCl, pH 8.0, 1 mM TCEP). A HiTrap Q column (GE Healthcare) and Q buffers A (25 mM TrisCl, pH=8.0, 20 mM NaCl, 1 mM TCEP) and B (25 mM TrisCl, pH 8.0, 1M NaCl, 1 mM TCEP) were used for the third purification step (first Q buffer A, then linear gradient to 50% Q buffer B over 25 column volumes). Elution from a Superdex 75 26/60 column was performed prior to the final spin concentration step. In a protocol variation without gel filtration, the buffer was exchanged by spin-concentrating (Vivascience; tandem SH2: 10 kDa filters; γSA: 30 kDa filters) and diluting thrice with 20 ml NMR buffer (25 mM Na_2HPO_4/NaH_2PO_4, pH=6.5, 50 mM NaCl, 5 mM DTT, 1 mM EDTA; in the first two runs concentrated 500 μl; the last run to 200-300 μl, if possible). The sample was snap-frozen and stored at -80 °C.

E664G-NSH2CSH2, E664K-NSH2CSH2 and cSH2 mutant constructs were expressed according to the similar, previously published procedure (10).

S1.2 Tyr783 Phosphorylation of proteins

FGFR1 kinase (464-775) with mutations only leaving a single, functionally relevant phosphorylation site was expressed and purified according to the procedure published previously (10). ^{15}N- or ^{13}C,^{15}N-labelled NSH2CH2 or γSA (as always with Tyr771Phe and Tyr775Phe mutations) was used for phosphorylation. 200 µl Strepactin Macroprep beads (IBA) were washed three times with phosphorylation buffer A (25 mM TrisCl, 150 mM NaCl, 1 mM TCEP, pH = 8.0) and incubated for 5 minutes with 0.4 mg FGFR1 kinase. The identical volume phosphorylation buffer B (25 mM TrisCl, 150 mM NaCl, 1 mM TCEP, 20 mM ATP, 50 mM MgCl2, pH 8.0) was added to obtain an ATP concentration of 10 mM; the solution was kept at room temperature for 10 minutes. 10 mg tandem SH2 was then added. The sample was left for 48 hours in the cold (4 °C). After centrifugation at low speed to precipitate the beads, the supernatant was applied to a gel filtration procedure equivalent to the procedure described in the purification protocol (see above).

S1.3 Molecular dynamics simulations

All molecular dynamics simulations were performed in Gromacs v. 4.5.4 (33) and resulting trajectories processed either with the same package or with VMD (34).

NSH2CSH2 (PDB ID: 4FBN) and NSH2CSH2-pY^{783} (PDB: 4EY0) crystal structures were taken as starting structures after modelling missing residues and performing an energy minimization with Xplor-NIH v. 2.3.8 (35); the Arg687Trp-mutated constructs were also generated with Xplor-NIH v. 2.3.8.

The AMBER ff99SB-ILDN force field (36) was used for all simulations. A phosphotyrosine residue topology was not available in GROMACS, therefore an AMBER parameter set for phosphotyrosine residues (37) was integrated into GROMACS using a conversion script (38). The phosphotyrosine residue side chain was assumed to be in a nonprotonated state.

The protein was set to be in a dodecahedral box; the box dimensions were 2 nm plus the maximum diameter of the protein. TIP3P was chosen as a water model, and the NaCl concentration was set to 150 mM. The following runs were performed for preparation: 1) L-BFGS energy minimization, timestep 0.001 ps, 5 ps duration, protein atom positions restrained; 2) L-BFGS energy minimization, timestep 0.001 ps, 10 ps duration, protein atom positions not restrained; 3) equilibration run 1 - leap frog integrator, timestep: 0.0005 ps, simulation duration: 100 ps, protein atom positions restrained. Coulomb interactions: Particle-Mesh Ewald; short range: 0.95 nm. Van-der-Waals-type: cut-off (0.95 nm). NPT ensemble; velocity-rescale thermostat: t_τ = 0.1 ps, reference temperature = 300 K; Berendsen barostat, isotropic, separate protein / non-protein coupling, t_P = 1.5 ps, compressibility = 4.5 10^{-5} bar^{-1}, reference pressure = 1.0 bar; 4) equilibration run 2: equal to equilibration run 1, but no protein atom position restrains, timestep: 0.002 ps; 5) equilibration run 3: leap frog integrator,

timestep: 0.002 ps, simulation duration: 100 ps, protein atom positions not restrained. Coulomb interactions: Particle-Mesh Ewald; short range: 1.0 nm. Van-der-Waals-type: cut-off (1.0 nm). NVT ensemble; Berendsen thermostat: t_τ = 1.0 ps, reference temperature = 298 K; 6) The production run was performed with the same parameters as the last equilibration run, but longer (up to 100 ns). Our local cluster was used (per calculation 2x8 cores, with LAM-MPI, efficiency: ~ 8.5 ns simulation / (day * 16 cores * ~ 50,000 atoms).

Contact events between two regions (any number of residues) were identified using the GROMACS g_mindist function (cutoff 2.5 Å), a bash script for this is provided in *Suppl. Inf. 3.1*. At a given frame, exactly one contact event involving two regions can occur: Any number of contacts (< 2.5 Å) at a given time point is counted as a single contact event. The contact events are added and normalized to the maximum number of contact events to give a contact probability, using a sliding average window of 1 ns.

For principle component analysis, the GROMACS 4.5.4 g_anaeig tool was used. In order to determine whether a certain eigenvector of one structure (obtained from the merged trajectory) can be used to describe the motions in a given trajectory of the same or another structure, the eigenvalue range of this given trajectory, projected on the eigenvector in question, can be estimated. The reference eigenvalue range is defined by the projection of the original merged trajectory used to generate the eigenvector in question. The projection operation was performed for any trajectory on any of the eigenvectors extracted from the three to six merged trajectories of the four different structures (three NSH2CSH2, four NSH2CSH2-pY^{783}, three R687W-NSH2CSH2-pY^{783}, three R687W-NSH2CSH2). Average values and standard deviations for the eigenvalue minimum and maximum for each structure projected on each eigenvector were determined using all non-merged trajectories. In order to compare 1D projections of trajectory sets on eigenvectors, a custom-made script (provided in *Suppl. Inf. 3.2*) was used.

Mutual information analysis: Six (NSH2CSH2) or three (NSH2CSH2-pY^{783}, R687W-NSH2CSH2-pY^{783}, R687W-NSH2CSH2) 100 ns trajectories were used for analysis. From each of these trajectories, six 10 ns bins starting at 40 ns were extracted, yielding 36 (NSH2CSH2) or 18 (others) 10 ns bins. The tool Mutinf (18) typically uses a combination of several 10 ns blocks for analysis. We performed 10 Mutinf runs using 5 randomly selected blocks (options –n 5 and –o 5), generating resampled datasets. The required side chain angle files were obtained from the trajectories using the Gromacs tool g_chi, with the following options: -maxchi 6 -phi -psi -all. The Mutinf scripts were compiled and run on a dedicated, custom-modified Linux Ubuntu machine, following the provided instructions. Residue Y783/pY783 was excluded from analysis. Mutinf running options were: -g gcc -a "yes" -o 5 -w 30 -o 0 -n 5. The mutual information sums for each residue, without Wilcoxon test, were collected to then have 10 mutual information data points for each residue. The

results are shown unfiltered in this paper. To establish the relevance of a difference in mutual information between two constructs (for example NSH2CSH2 vs. NSH2CSH2-pY^{783}), a two-sided t-test (unequal population variances, Welch's t-test) was performed, using the SEM obtained from 10 data points, obtained from Mutinf runs which were based on resampled data. Residue-wise mutual information differences between two constructs are only shown if this test returns that they are not equal with $p > 0.95$. The python data processing and visualization script, starting from Mutinf results, is provided in *Suppl. Inf. 3.3*.

S1.4 Nuclear magnetic resonance spectroscopy: Backbone resonance assignment and $^{1}H,^{15}N$-heteronuclear correlation experiments

Bruker Avance III (600, 700, 800 and 950 MHz) NMR spectrometers equipped with cryogenically cooled triple resonance probes with a z-axis pulse field gradient coil were used for all NMR experiments. Spectra were recorded at 25 °C in NMR buffer (25 mM Na_2HPO_4/NaH_2PO_4, pH 6.5, 50 mM NaCl, 5 mM DTT, 1 mM EDTA and 9% D_2O), unless stated otherwise. Shigemi NMR tubes were used to maximize protein concentration for most experiments, because sample availability rather than solubility is a limiting factor. Protein concentrations were usually 100 - 250 μM; results in this paper are concentration-independent.

A standard set of 3D backbone resonance assignment spectra (HNCA, HNCOCA, HNCACB, CACBCONH, HNCO and HNCACO) using Bruker library pulse sequences (with Watergate water suppression) were recorded for $^{15}N,^{13}C$-labelled NSH2CSH2 and NSH2CSH2-pY^{783} samples; HNCA spectra were also recorded for NSH2CSH2$^{\Delta CT}$ and R687W-NSH2CSH2-pY^{783}. The respective $^{1}H,^{15}N$-TROSY spectra using echo-antiecho and gradient water suppression were recorded for $^{2}H,^{15}N,^{13}C$-labelled NSH2CSH2 (39, 40). Non-uniform sampling (NUS) was used to obtain a high-resolution HNCA spectrum for $^{2}H,^{15}N,^{13}C$-nonphospho- and $^{15}N,^{13}C$-NSH2CH2-pY^{783} (41, 42). The NUS fraction 17%, and the transverse relaxation time estimate for NUS was set to 8 ms.

$^{1}H,^{15}N$-HSQC-type experiments for γSA constructs displayed many more cross peaks when being recorded at 34 °C rather than at 25 °C. γSA-pY783 constructs were stable at 25 °C only for a relatively limited duration. Superposition of the recorded and assigned $^{1}H,^{15}N$-HSQC NMR spectra for the isolated SH3, spPH, and NSH2CSH2 and NSH2CSH2-pY^{783} proteins (for spPH assignments see (5)), in combination with a 3D HNCA spectrum recorded for a Tyr771Phe-γSA construct allowed for transfer of some resonances. In some cases, the assignments were confirmed using a 3D HNCA spectrum recorded for a Tyr771Phe-γSA construct; only this particular γSA construct was available at a sufficiently high concentration and stability to record a 3D spectrum.

Bruker pulse sequences hsqcfpf3gpphwg (version 12/01/11) or sfhmqcf3gpph (version 13/02/28) were used to record $^{1}H,^{15}N$-HSQC spectra and $^{1}H,^{15}N$-SOFAST-HMQC spectra, respectively. For

NSH2CSH2$^{\Delta CT}$-CTpY samples, the pTyr783-peptide NPGFpYVEANPMP (PLCγ 779-790) was added in excess prior to performing the experiment (10). The PDGFRβ peptide TSNQEpYLDLSM was obtained from Cambridge Peptides Ltd; for more information, see Bunney et al. (10).
Backbone resonance assignments were submitted to the BioMagResBank, accession number 27496.

S1.5 NMR data processing and analysis of fast exchange

NMR raw data were preprocessed with NMRPipe and NMRDraw (44). Non-uniform sampling data were analyzed using the iterative re-weighted least squares reconstruction algorithm (IRLS) (42) implemented in the MddNMR 2.1 (45) suite. NMR spectra were analyzed with CcpNmr Analysis (46). Chemical shift perturbation was calculated from ^{1}H and ^{15}N chemical shift differences by adjusting the ^{15}N shift differences using 0.14 as a scaling factor for ^{15}N shift changes (47).
For analysis of relaxation data, CcpNmr Analysis was used to pick cross peaks and fit peak heights to exponential decays to get the relaxation time constants T_1 and T_2. CcpNmr returns a fitting error that was used as the error for R_1 and R_2. The error was obtained from the fitting procedure.

S1.6 Cell culture, transfection, and fractionation.

COS-7 and HEK293 cells were maintained at 37°C in a humidified atmosphere of 95% air and 5% CO2 in Dulbecco's modified Eagle's medium (DMEM) (Invitrogen) supplemented with 10% (v/v) fetal bovine serum (Invitrogen) and 2.5 mM glutamine. Prior to transfection, cells were seeded into 6-well plates at a density of 2.5×10^{5} cells/well and grown for 16 h in 2 ml/well of the same medium. For transfection of COS-7, 1.0 µg of PLCγ DNA was mixed with 1 µl PlusReagent and 7 µl Lipofectamine (Invitrogen) and the mixture added to the cells in 0.8 ml DMEM without serum. The cells were incubated for 3.5 h at 37°C, 5% CO_2 before the transfection mixture was removed and replaced with DMEM containing serum.

S1.7 Analysis of inositol phosphate formation in intact COS-7 cells.

This analysis was performed essentially as described previously (5, 48). Briefly, 24 h posttransfection, cells were labeled with 1.5 µCi/ml myo-[2-^{3}H]inositol. After a further 24 h, the cells were incubated in 1.2 ml inositol-free DMEM, without serum, containing 20 mM LiCl with or without stimulation with 100 ng/ml epidermal growth factor (EGF; Calbiochem) for 1 h. The cells were lysed by the addition of 1.2 ml 4.5% perchloric acid, and supernatants and pellets were separated. Inositol phosphates were collected using AG1-X8 200-400 columns (Bio-Rad). The levels of inositol phosphates were quantified by liquid scintillation counting using Ultima-Flo scintillation fluid (PerkinElmer). The PLC activity analyzed only by this standard measurement is given as "PLC activity

(cpm).” Data shown are the means ± SDs of triplicate samples and are representative of three or more independent experiments.

S1.8 Relaxation experiments

Relaxation data (^{15}N-R_1, ^{15}N-R_2) for NSH2CSH2 and NSH2CSH2-pY^{783} were collected at 600 MHz similar to a procedure described elsewhere (43) and based on Bruker pulse sequences hsqct2etf3gpsi3d.2 (version 04/01/05) and hsqct1etf3gpsi3d.2 (04/01/05). Recovery delays for ^{15}N-R_2 relaxation experiments were set to 8, 16, 24, 40, 56, 72, 96 and 120 ms or 8, 16 and 40 ms (for the NSH2CSH2 sample at 30 μM). Recovery delays for ^{15}N-R_1 relaxation experiments were set to 10, 100, 200, 300, 500, 700, 800 and 1200 ms. The delay time recording schedule was randomized with data recorded an in interleaved manner.

S1.9 Peptide titration experiments for K_D determination

Peptide titration experiments were performed at constant volume by titrating peptide into protein solution and recording either ^{15}N-HSQC or ^{15}N-SOFAST experiments. Due to peak broadening, overlap or complex exchange phenomena, not many peaks were eligible for quantitative analysis. The peptide binding occurs in the slow exchange regime (with minimal broadening in some cases), which is why peak volumes rather than positions were used for quantiative analysis.

The following pairs of titration pairs were examined to determine K_Ds: CSH2 (-770) (conc. 432 μM) and pY783 peptide (peptide/protein ratio 0 – 2.0; residues analyzed: Glu679, GlyG689, Ser701, Glu720, Ser739); NSH2CSH2 (conc. 200 μM) and pY783 peptide (peptide/protein ratio 0 – 1.25; residues analyzed: His670, Ala703, Cys715, Glu742); CSH2 (-790) (conc. 117 μM) and PDGFR peptide (peptide/protein ratio 0 – 1.2; residues analyzed: Glu679, Gly689, Gly710, Cys715, Gly727, Glu742).

The dissociation constant for binding of a single ligand to a protein is defined as

$$K_D = \frac{([L_0]-[PL])([P_0]-[PL])}{[PL]} = \frac{([L_0]-f[P_0])([P_0]-f[P_0])}{f[P_0]},$$

with $[P_0]$: total concentration of protein; $[L_0]$: total concentration of ligand; [PL]: protein-ligand complex concentration; $f=[PL]/[P_0]$.

This can be rewritten

$$K_D[P_0]f = [L_0][P_0] - f[L_0][P_0] + f^2[P_0]^2 - f[P_0]^2$$

$$f = \left(\frac{[P_0]+[L_0]+K_D}{2[P_0]}\right) + \sqrt{\left(\frac{[P_0]+[L_0]+K_D}{2[P_0]}\right)^2 - \frac{[L_0]}{[P_0]}}$$

Sufficiently separated peaks (see above which residues) in the titration spectra for the bound and the unbound form were selected for fitting. The fraction f, representing the ratio of complex to total protein concentration, were obtained from total peak volumes. For $f < 0.5$, f was obtained from the intensity of the peaks corresponding to the unbound state. A simultaneous fit to the above equation was performed with a custom Python (2.7) script using the Numpy 1.11.3, Matplotlib 2.0.0 and the Scipy. 0.18.1 libraries (the iPython notebook is provided in *Suppl. Inf. 3.4*). For sampling statistics, 6-8 subsets of data (2-3 data points per peptide concentration) were used for the fitting procedure (jackknife resampling).

S1.10 Small Angle X-Ray Scattering (SAXS)

Small angle X-ray scattering (SAXS) data were recorded at the Deutsche Elektronen-Synchrotron (DESY) facility using synchrotron radiation from the DORIS III electron-positron storage ring. SAXS data have been recorded for NSH2CHS2 and NSH2CSH2-pY783. Note that the construct used for NSH2CSH2 analysis did not contain the Tyr771,775Phe mutations as in other parts in the paper (however, 2D NMR spectra for these two minimally different constructs are almost identical). Both constructs were measured in SAXS buffer (25 mM Tris-Cl, 150 mM NaCl, 10% (v/v) glycerol, 5 mM TCEP, pH 8.0) at the following concentrations: NSHCSH2 - 5.83, 4.02, 2.24 mg/ml; NSH2CHS2-pY^{783}: 5.82, 4.05, 2.16 mg/ml. Scattering data were recorded with a Pilatus detector and an EMBL X33 camera.

The initial SAXS data analysis for calculating the radius of gyration and maximum diameter was performed with PRIMUS (49). Most processing steps were performed using a variety of tools of the ATSAS program package (49, 50), including basic SAXS data processing. Fitting of an ensemble of structures (rigid bodies with flexible linkers) to a SAXS data was performed using the tool EOM (ensemble optimization method).

S2. Supporting Results

S2.1 Relaxation experiments and SAXS experiments showing aggregation

The ^{15}N R_2/R_1 ratios for NSH2CSH2-pY^{783} and NSH2CSH2 (both ≈28.5 kDa) were measured residue-wise. The expected average R_2/R_1 ratio for a spherical protein of 28.5 kDa is estimated as a value around 23.1 (at 600 MHz, estimated from standards measured by Rossi et al.) (51). The measured

average ^{15}N R_2/R_1 ratio at 600 MHz for NSH2CSH2-pY^{783} is 26 at 150 μM, which is close to the value expected for this protein. The average ^{15}N R_2/R_1 ratio at 600 MHz for NSH2CSH2 is 34. Comparing ^{15}N R_2 values of NSH2CSH2 with those of NSH2CSH2-pY^{783} also reveals that NSH2CSH2 has rather high ^{15}N R_2 values, as it is expected for high ^{15}N R_2/R_1 ratios. Dilution of NSH2CSH2 (from 150 μM to 30 μM) returns ^{15}N R_2 values that are much smaller than those recorded for NSH2CSH2 at a higher concentration (150 μM). The reason for the large ^{15}N R_2/R_1 ratios in undiluted samples is probably a propensity of NSH2CH2 to aggregate. Importantly, this aggregation did not lead to any change in chemical shifts.

Small angle X-ray scattering (SAXS) experiments were performed in order to test for aggregation propensity of NSH2CHS2 and NSH2CSH2-pY^{783}. The shape of the SAXS curves, general parameters and distance distribution function match broadly to what would be expected for these two-domain proteins (*Fig. S4*). However, the gyration radius and volume is higher for NSH2CSH2. The large distances for NSH2CSH2 which are found in the distance distribution cannot be explained by the presence of a flexible nSH2-cSH2 junction and a flexible C-terminus alone; we tested this by generating structural ensembles using the Ensemble Optimization Method (50) in which rigid-body structures (individual SH2 domains) are connected by flexible linkers. In addition, we find a concentration dependency of R_g (*Fig. S4a*), revealing that the SAXS data are affected by partial aggregation of this construct.

S2.2 Crystal structure and chemical shift patterns revealing the potential role of interactions between Asn757 and the nSH2-cSH2 junction

According to the crystal structures, the side chain NH_2 group of residue Asn757 at the beginning of the pre-C-terminus could form a hydrogen bond to the backbone carbonyl oxygen of nSH2-cSH2 junction residue Glu667 (distances for Asn-Nδ - Glu-O: 4FBN – 3.4 Å; 4EY0 – 3.9 Å; 3GQI – 3.9 Å), which would offer a potential allosteric connection from Y783 to the nSH2-cSH2 junction that depends on the presence of the C-terminal linker (*Fig. S8b*).

MD simulations predicted an unstable contact between Asn757-Glu759 and the nSH2-cSH2 junction in NSH2CSH2-pY^{783}. The major cross peak for Asn757 in the CSH2 spectrum is located on an *extension* of the vector connecting the corresponding cross peak positions in the spectra of NSH2CSH2 and NSH2CSH2-pY^{783} (*Fig. S7b*), i.e. not *between* these two positions. How might this pattern be rationalized within the context of the exchange phenomenon invoked above? One means is as follows. For NSH2CSH2-pY^{783} it might be expected that for most residues $p^i(\text{closed}) \sim 1$. Within the model of chemical exchange described above the Asn757 peak position for CSH2 suggests $p^{N757}{}_{\text{closed}} > 1$, which is clearly not reasonable. Rather, the cross peak position in NSH2CSH2-pY^{783} may reflect balance

between 'closed' and (a - naively - unexpected non-zero population of) 'open' states specifically at Asn757 (different to the situation at Tyr783), and that p^{N757}_{closed}(NSH2CSH2-pY^{783}) < p^{N757}_{closed}(CSH2). In this scenario the presence of the nSH2 domain in the NSH2CSH2-pY^{783} protein has the effect of 'loosening' the interaction between the cSH2 domain and the pre-C-terminus, if we assume that this is the structural equivalent of a "more open" conformation. This scenario is equivalent to inferring that even in the case of NSH2CSH2-pY^{783} the pre-C-terminus is not fully bound; this has been predicted by MD simulations (*Fig. 2d*). It can be inferred that residue Asn757 in NSH2CSH2-pY^{783} is not always in the 'closed' state, possibly due to the nSH2 domain weakening the interaction between the cSH2 domain and the pre-C-terminus via contacts between the nSH2-cSH2 junction and the pre-C-terminus.

Taking the likely structural contact between the nSH2-cSH2 junction and the Asn757 domain into account, it appears likely that the nSH2 exercises the loosening influence on the pre-C-terminus / cSH2 interaction via the nSH2-cSH2 junction.

S2.3 Combined slow and fast exchange kinetics can explain peak tripling for residue Thr766

Residue Thr766 in the pre-C-terminus provides another example of a residue exhibiting both slow and fast exchange (*Fig. S12b)*. In this case, three cross peaks are detected, one of which is at the same position as that detected for NSH2CSH2$^{\Delta CT}$. Of the other two, one appears significantly broadened. It is a challenge to unambiguously decipher the underlying mechanism that gives rise to this pattern. However, the following argument can be posited. The first cross peak can be thought of a corresponding to a state O_3 wherein the C-terminal tail is locally 'open'. The second cross peak represents a state which is exchanging rapidly between 'closed' and 'open' forms $\{C \rightleftharpoons O\}_2$ with an equilibrium partitioning given by $K^i_2 = p^i_{2,C}/p^i_{2,O}$. The third cross peak represents another state which exchanges $\{C \rightleftharpoons O\}_1$ at a different rate and is partitioned according to $K^i_1 = p^i_{1,C}/p^i_{1,O}$. The *local* structures O_1, O_2 and O_3 and likewise the *local* structures C_1 and C_2 can reasonably assumed to be identical because the cross peaks for O_3, $\{O/C\}_2$ and $\{O/C\}_1$ are all located on the same vector connecting the cross peaks for $O_1/O_2/O_3$ and C_1/C_2. However, what gives rise to the presence of multiple cross peaks is a slow conformational process that is *remote* but nevertheless 'sensed' at Thr766 by virtue of a shift in the extent of 'closed' character at that position in the different states. A general kinetic scheme for this exchange situation would read $\{O \rightleftharpoons^{*} C\}_1 \rightleftharpoons^{\dagger} \{O \rightleftharpoons^{*} C\}_2 \rightleftharpoons^{\dagger} O_3$, where * and † denote fast and slow exchange equilibria, respectively. Sample schemes illustrating possible more specific underlying kinetic schemes are illustrated in *Fig. S12d*.

S2.4 Proline *cis-trans* peptide bond isomerization could be the source of slow exchange

One can speculate that in the context of the NSH2CSH2 protein, the source(s) of slow processes could be *cis-trans* peptide bond isomerization in the C-terminal region at Glu768-Pro769, Asn779-Pro780, Asn787-Pro7888 and/or Met789-Pro790. In line with wide experience of unstructured peptides, such isomerization would be on a slow timescale, and is highly likely in the relatively unconstrained Tyr783-unbound state of the C-terminal region. It is entirely plausible that the *cis-/trans-* state of one or more of these peptide bonds could influence the effective affinity of the Tyr783 region for the cSH2 domain, and thereby give rise to the observed NMR characteristics. We have performed initial experiments comparing the WT CSH2, Pro686Ala, Pro745Ala, Pro755Ala and Pro769Ala mutant constructs (Fig. S*12a*). Chemical shift analysis suggests that these mutations can dramatically shift the exchange behavior of multiple residues. For several residues, the Pro686Ala and Pro769Ala substitutions tilt the dynamic equilibrium towards the 'open' state; note that Pro686 and Pro759 are spatially close to each other, and that Pro686 neighbors the site of the disease-relevant residue Arg687. Comparing the cross peak patterns for WT and Pro745Ala proteins suggests that the 'open' population(s) might often be lower in the latter case. Overall these observations are strongly suggestive that *cis-trans* peptide bond isomerization in the extended C-terminus is contributing to the complexity in the spectra of the cSH2 domain-containing proteins. Moreover, that complexity serves to reinforce the conclusion that the various residues within the C-terminal linker are in dynamic binding equilibria with the cSH2 domain surface.

S2.5 Structural interactions between the cSH2 α_B helix and the nSH2-cSH2 junction

The structural and dynamic interactions between the cSH2 α_B helix (including surrounding residues) and the nSH2-cSH2 junction are of interest because of the impact of allosteric communication triggered by Tyr783 phosphorylation. In order to predict the effect of Tyr783 phosphorylation on the nSH2-cSH2 junction, the potential for interaction between the junction and nearby residues, specifically those in cSH2 helix α_B, were identified from MD simulation trajectories (*Fig. S8a*). Transient interactions between nSH2-cSH2 junction residue Trp668 and α_B residue Ile738, as well as between junction residues Thr660-His663 and α_B residue Ser733, are much more pronounced in the NSH2CSH2 trajectories than for NSH2CSH2-pY^{783}, suggesting that Tyr783 phosphorylation leads to a reduction of some contacts between the nSH2-cSH2 junction and the α_B helix; other contact analysis focusing on the α_B helix (with surrounding residues, Ser732-Glu742) and on nSH2-cSH2 junction

sections (Thr660-His663 or Glu664-Lys666) show a slightly stronger interaction in the phosphorylated state.

In agreement with the MD simulation, Ile738 chemical shifts are sensitive to structural changes in the nSH2-cSH2 junction (*Fig. S8d*). Similar perturbations were observed for neighboring residue Leu737 (*Fig. S8d*). A particularly large perturbation in the CSH2 spectrum indicates the potential for a direct contact between Ile738 and the nSH2-cSH2 junction that is disrupted upon removal of the nSH2 domain (*Fig. S8b*).

Chemical shift perturbation experiments also reveal that a large impact of Tyr783 phosphorylation on the cross peak position is evident for residue Glu742; a plot of the peak position for various constructs indicates that they can be interpreted within a framework of fast exchange (*Fig 5a*). Inspection of the cross peaks for NSH2CSH2, NSH2CSH2$^{\Delta CT}$, NSH2CSH2-pY^{783}, E664G-NSH2CSH2, E664K-NSH2CSH2 and CSH2 reveals an equivalence with the peak patterns observed in context of the dynamic allosteric C-linker dependent pathway. However, comparison of NSH2CSH2-pY^{783} and NSH2CSH2$^{\Delta CT}$-CTpY peak positions relative to NSH2CSH2$^{\Delta CT}$ indicate that the presence and absence of the C-terminal linker have opposing effects for Glu742 in the two phospho-constructs. First, it is indicated that even without C-terminal linker presence, pY^{783} phosphorylation can be communicated to this residue. Secondly, the assumption that NSH2CSH2$^{\Delta CT}$ is wholly ‘open’ ($p_C = 0$) does not hold because the NSH2CSH2$^{\Delta CT}$-CTpY cross peak is not located between the corresponding NSH2CSH2$^{\Delta CT}$ and NSH2CSH2-pY^{783} peaks but rather on an extension of a vector pointing from NSH2CSH2-pY^{783} to NSH2CSH2$^{\Delta CT}$. Thus, it is not certain to what extent the two states underlying the Glu742 peak positions can be identified as strictly ‘open’ or ‘closed’. C-terminal linker-dependent and -*in*dependent binding have an opposing effect on the Glu742 chemical shift, suggesting that the nearby nSH2-cSH2 junction structure might be modulated in a different manner in each case.

S2.6 C-terminal linker-*in*dependent allostery

In agreement with the location of the putative pY783 peptide binding site, which has been described elsewhere (10, 11), large CSPs are observed between NSH2CSH2$^{\Delta CT}$-CTpY and NSH2CSH2$^{\Delta CT}$ for multiple β-sheet residues: Peak positions for β-sheet residues Arg694, Lys695, Ala703, Ile704, Ser705 and Glu720 are essentially identical for NSH2CSH2-pY^{783} and NSH2CSH2$^{\Delta CT}$-CTpY (*Fig. S7d*), indicating that these residues are mostly affected by C-terminal linker-*in*dependent effects of pY783 peptide binding. The nSH2-cSH2 junction and α_B helix (with surrounding residues) are not part of the putative pY783 peptide binding site. However, cross peak position differences between NSH2CSH2$^{\Delta CT}$ and NSH2CSH2$^{\Delta CT}$-CTpY observed for junction residue Thr660 and residue Ala732

(close to the α_B helix, *Fig 4a*, *Fig. S8c*) indeed reveal a significant C-terminal linker-*in*dependent allosteric connection to the nSH2-cSH2 junction.

S2.7 Peptide titration experiments for K_D determination

For the peptide titration method and data processing see *Suppl. Mat. S1.9*, All peptide titrations reveal strong binding of the peptides to the cSH2 domain. The K_D for PDGFRβ peptide is estimated to be ≤ 1.1 μM. Due to peak broadening, overlap and exchange phenomena, a precise estimation of the K_D of binding pY^{783} peptide to the cSH2 domain is challenging. The K_D for the system NSH2CSH2 / pY^{783} is 3.1±1.1 μM and for $CSH2^{\Delta CT}$ / pY^{783} 14.6±1.1 μM. Titration plots, with all data points and overall fit, are shown in *Fig. S4*. At much higher pY^{783} peptide/protein ratio (> 1.5), secondary peptide binding to the nSH2 domain occurs. From these results, and from qualitative inspection of $NSH2CSH2^{\Delta CT}/pY^{783}$ peptide (1.2 equivalents) spectra, we can confirm that in all NMR experiments of this paper, PDGFR and pY^{783} peptide is fully bound to cSH2 and in slow exchange.

S2.8 Molecular dynamics simulations of Arg687Trp-mutant constructs

Three 100 ns simulations for each R687W-NSH2CSH2 and R687W-NSH2CSH2-pY783 were performed for comparison with WT constructs. The predictive value of these simulations is limited as they were not started from crystal structure, but rather from Arg687Trp *in silico* mutated constructs.

Principal component analysis was extended to Arg687Trp constructs. The projections of each trajectory on the PCA eigenvectors revealed a difference between R687W-NSH2CSH2 and NSH2CSH2 trajectories in NSH2CSH2 eigenvector 2 (*Fig. S1a*); the same eigenvector has been found to be relevant to describe differences between NSH2CSH2 and NSH2CSH2-pY783. In addition, R687W-NSH2-CSH2 eigenvector 3 and R687W-NSH2CSH2-pY783 eigenvector 2 reveal differences between Arg687Trp and WT construct (*Fig. S1c-d*). A high root-mean-square fluctuation (RMSF) of the C-terminal linker and the pre-C-terminus in these eigenvectors *(Fig. S10b-c)* suggests that the dynamics of these regions differs between Arg687Trp and WT.

Comparison of mutual information analysis results for R687W-NSH2CHS2 trajectories with NSH2CSH2 trajectory results (*Fig. S10a*) reveal that the Arg687Trp mutation leads to a general reduction of mutual information between C-terminal residues and a great number of cSH2 residues, including the nSH2-cSH2 junction, and some residues in the nSH2 domain that are spatially close to the nSH2-cSH2 junction. These data suggest that the Arg687Trp disrupts the allosteric network which we have found in the WT construct.

The results of contact probability analysis for R687W-NSH2CSH2 and NSH2CSH2 molecular dynamics trajectories are ambiguous for contacts between pre-C-terminal and C-terminal residues and the cSH2 domain: contact events between C-terminal linker and the α_A helix are more likely in R687W-NSH2CSH2, while many other contacts are less probable (*Fig. S9a*); however, additional simulation material for R687W-NSH2CSH2would be required to yield more precise predictions. Comparing R687W-NSH2CH2 with R687W-NSH2CSH2-pY^{783} suggests that allosteric communication might be disrupted in the mutant: most contact probability changes for contacts with the nSH2-cSH2 junction (related to Tyr783 phosphorylation) that were observed in the respective WT constructs could be observed in the presence of the Arg687Trp substitution (*Fig. S9c*), with the exception of a higher probability of contacts between (a) Tyr783 and cSH2 and (b) Thr660-His663 and Asp732-Glu742 (α_B helix and surrounding residues; *Fig. S9d*). For additional information about contacts between the α_B helix and the nSH2-cSH2 junction, see also *Suppl. Inf. 2.5*.

In summary, MD simulations suggest that the Arg687Trp mutation reduces allosteric communication across the cSH2 domain. This disruption of allostery appears to be linked in a change of the structure or dynamics of the C-terminus.

S2.9 PDGFRβ peptide titration to explore combined, pre-formed slow/fast exchange equulibria in CSH2

The PDGFRβ peptide was employed previously in context of a study of PLCγ1 and is known to bind to CSH2 (10, 52). We found strong binding ($K_D \leq 1.1$ μM) in a titration experiment (*Suppl. Inf. 2.7, Fig. S6*). In the titration, the PDGFRβ peptide shifts the slow exchanging populations of residues sensitive to the attached C-terminal linker, towards the 'most open' state exemplified by Gly710 in *Fig. S15a* and Gly689, Gly727, Glu742, Gly765 and Gly777 in *Fig. S15c*). Remarkably, in this experiment more than two slowly exchanging states are evident. The Gly710 site in the cSH2 domain αA-β1 loop is remote from the PDGFRβ peptide binding site. Specifically, for this residue we find that the population of a partially 'closed' state $\{O \rightleftharpoons C\}_1$ falls upon addition of the phosphopeptide (*Fig. 15b*). As more peptide is added, the cross peak intensity is then successively distributed between a number of resonances that are located on the vector connecting $\{O/C\}_2$, $\{O/C\}_3$ and the complex between the cSH2 domain and the PDGFRβ peptide, here denoted O:P. Namely, the observed titration behaviour can be understood by invoking an extension of the combined fast/slow exchange scheme:

$(\{O \rightleftharpoons^{*} C\}_1 \rightleftharpoons^{\dagger} \{O \rightleftharpoons^{*} C\}_2 \rightleftharpoons^{\dagger} \{O \rightleftharpoons^{*} C\}_3 \rightleftharpoons^{\dagger} O{:}P$

Close inspection of the CSH2 peak pattern suggests that the set of $\{O/C\}_i$ states pre-exist in WT CSH2; the population shifts occur due to peptide association at the (remote) binding site, leading to local

changes of the dynamic equilibrium between states where the C-terminal linker is associated, or not, with the surface of the cSH2 domain.

S3 Bash and Python Scripts

S3.1 MD data processing - contact event detection in Gromacs trajectories using bash scripts and g_mindist

main bash script for contact analysis

```
#!/bin/bash
for x in $(echo $1) #need $1.gro and $1.xtc in this folder.
do
printf "" > "$x"summat.dat
printf "" > "$x"freqmat.dat
y=$x
for z in $(echo "650-668 670-750 675-684 650-659 660-668 660-663 664-666 667 668 680-690 687");
do
printf "$x"'\n'"$y"'\n'"$z"'\nq\n757-790\n757-770\n771-778\n779-790\n757-759\n760-766\n767-770\n783\n732-
742\n733\n738\nq\n' | ./mindistcalc.sh
sleep 0.01
echo "header" > "$1"res.csv; cat "$1"_n.csv | awk '{printf "xx "}{for (i=1; i<=NF/2; i++){printf "%i ",
$(2*i)}}{printf "\n"}' >> "$1"res.csv
cp "$x"res.csv ./"$x"_"$z"res.csv
echo $(cat "$x"res.csv | sed "2,$ ! d" | awk '{for (i=2; i<=NF; i++) {if ($i>0) printf "%s ", 1; else printf "%s ",
0} {printf "\n"}}' | awk 'BEGIN{for (i=1; i<=NF; i++) {x[i]=0;z=0}}{z+=1;for (i=1; i<=NF; i++) {x[i]+=$i}}END{for
(i=1;i<=NF;i++){printf "%i ", 1000000*x[i]/(z)}}') >> "$x"summat.dat
echo $(cat "$x"res.csv | sed "2,$ ! d" | awk '{for (i=2; i<=NF; i++) {if ($i>0) printf "%s ", 1; else printf "%s ",
0} {printf "\n"}}' | awk 'BEGIN{for (i=1; i<=NF; i++) {x[i]=0; y[i]=$i; z[i]=0}}{z+=1;for (i=1; i<=NF; i++) {if
(NR==1) {y[i]=$i}else {if (y[i]==0 && $i ==1) {x[i]+=1};if (y[i]==1 && $i ==0) {x[i]+=1};y[i]=$i}}}END{for
(i=1;i<=NF;i++){printf "%i ", 1000000*x[i]/(2*z)}}') >> "$x"freqmat.dat
sleep 0.01
done
done
```

bash script - mindistcalc.sh, called by the main bash script for contact analysis

```
#!/bin/bash
echo -e "Please type name of trajectory to process (no xtc at the end)"
read trjfile
echo -e "Please type name of mass file to take into account (no gro at the end)"
read massfile
declare -a index1list
declare -a index2list
count=0
while [ "$index1" != "q" ]
do
echo -e "Please put (mext) index1 variable (format i.e. 545 or 545-559), press q when finished"
read index1
index1list[(($count))]="$index1"
count=$(( $count + 1 ))
done
count2=0
while [ "$index2" != "q" ]
do
echo -e "Please put (mext) index2 variable, press q when finished"
read index2
index2list[(($count2))]="$index2"
count2=$(( $count2 + 1 ))
done
count3=0
firstline="time "
space=" "
while [ $count3 != $(($(($count)) - 1)) ]
do
count4=0
while [ $count4 != $(($(($count2)) - 1)) ]
do
rm tempndx.ndx
printf "r${index1list[$(($count3))]}\nr${index2list[$(($count4))]}\n\nq'" | make_ndx -f "$massfile".gro -o
tempndx.ndx
printf "10\n11\n" | g_mindist -f "$trjfile".xtc -group -s "$massfile".gro -od tempd.xvg -or tempr.xvg -on tempn.xvg
-xvg none -n tempndx.ndx -d 0.25
echo "$firstline""$space"r${index1list[$(($count3))]}_r${index2list[$(($count4))]}
firstline="$firstline""$space"r${index1list[$(($count3))]}_r${index2list[$(($count4))]}
        if [ $count3 == 0 ] && [ $count4 == 0 ]
        then
```

```
        mv tempr.xvg "$massfile"_r.csv
        mv tempd.xvg "$massfile"_d.csv
        mv tempn.xvg "$massfile"_n.csv
        else
        paste -d \  "$massfile"_r.csv tempr.xvg > "$massfile"t_r.csv
        paste -d \  "$massfile"_d.csv tempd.xvg > "$massfile"t_d.csv
        paste -d \  "$massfile"_n.csv tempn.xvg > "$massfile"t_n.csv
         rm temp*
        mv "$massfile"t_r.csv "$massfile"_r.csv
        mv "$massfile"t_d.csv "$massfile"_d.csv
        mv "$massfile"t_n.csv "$massfile"_n.csv
        fi
count4=$(($count4 + 1))
done
count3=$(($count3 + 1))
done
rm temp*
rm \#*
echo $firstline
echo $firstline > header.txt
```

S3.2 MD data processing – principal component analysis with post-processing (bash script)

```
#!/bin/bash
datasets='nph ph 687nph 687ph' #list of trajectory sets. Needs to have relevant .gro and .xtc files in the ../(dataset)/
path.
stres=658 #residue from which the PCA starts. set to 545 for the entire tandem construct.

###### preparation and fusion of trajectories. #######
mkdir fusetraj
cd fusetraj
for a in $datasets
do
n=0
#in this section, it is defined how trajectories will be merged for PCA. For example, in this example, the 'nph'
construct
#includes 6 trajectories of 50000 us length (because only 50-100 ns will be used).
if [[ $a == 'nph' ]]
then
trajst='10\n50010\n100010\n150010\n200010\n250010\n'
trajlst='tx_1.xtc tx_2.xtc tx_3.xtc tx_4.xtc tx_5.xtc tx_6.xtc'
elif [[ $a == 'ph' ]]
then
trajst='10\n50010\n100010\n150010\n'
trajlst='tx_1.xtc tx_2.xtc tx_3.xtc tx_4.xtc'
else
trajst='10\n50010\n100010\n'
trajlst='tx_1.xtc tx_2.xtc tx_3.xtc'
fi

##this aligns trajectories, ensures that they are whole, extracts backbone atoms of the desired segment, and fuses
trajectories.
for b in $(ls ../../"$a"/*.gro | sed "s/.gro//g")
do
echo $b
n=$((n+1))
cp "$b".gro ./"$a"_"$n".gro
printf '4&r'"$stres""-790\nq\n" | make_ndx -f "$a"_"$n".gro -o "$a"_"$n".ndx
printf "10\n" | trjconv -n "$a"_"$n".ndx -s "$a"_"$n".gro -f "$a"_"$n".gro -o "$a"_"$n"_cSH2.gro
printf "r670-750\nq\n" | make_ndx -f "$a"_"$n"_cSH2.gro -o "$a"_"$n"_cSH2.ndx
printf "1\n0\n" | trjconv -s "$a"_"$n"_cSH2.gro -f "$a"_"$n"_cSH2.gro -o "$a"_"$n"_cSH2b.gro -box 200 200 200 -center
echo $b
printf "10\n" | trjconv -n "$a"_"$n".ndx -s "$a"_"$n"_cSH2.gro -f $b.xtc -b 50010 -o ta_"$n".xtc

printf "10\n1\n" | trjconv -s "$a"_"$n"_cSH2.gro -f ta_"$n".xtc -fit progressive -o tb_"$n".xtc -n "$a"_"$n"_cSH2.ndx
printf "1\n" | trjconv -s "$a"_"$n"_cSH2.gro -f tb_"$n".xtc -o tc_"$n".xtc -b 50010 -e 100000 -n "$a"_"$n"_cSH2.ndx
printf "1\n0\n" | trjconv -s "$a"_"$n"_cSH2.gro -f tc_"$n".xtc -o tx_"$n".xtc -box 200 200 200 -center
done

printf $trajst | trjcat -f $trajlst -o tx_comb.xtc -cat -settime
printf "1\n" | trjconv -s "$a"_"$n"_cSH2b.gro -f tx_comb.xtc -pbc nojump -o ty_comb.xtc
printf "1\n1\n" | trjconv -s "$a"_"$n"_cSH2b.gro -f ty_comb.xtc -fit progressive -o "$a"_cSH2.xtc
cp "$a"_"$n"_cSH2b.gro ./"$a"_cSH2.gro
echo 'done'
done
cd ..


############ This is the actual principal component analysis, based on the fused trajectories generated
beforehand.#####
mkdir pca_docsh2
cd pca_docsh2

for x in $datasets
do
mkdir $x
cp ../fusetraj/"$x"_cSH2.xtc ./$x/"$x"_cSH2.xtc
cp ../fusetraj/"$x"_cSH2.gro ./$x/"$x"_cSH2.gro
done
cwd=$(pwd)
```

```
for x in $datasets
do
cd $x
printf "1\n1\n" | g_covar -s "$x"_cSH2.gro -f "$x"_cSH2.xtc -av aver_"$x".pdb -o eigenval_"$x".xvg -l covar_"$x".log
-v eigenvec_"$x".trr
cd $cwd
done

cwd=$(pwd)

############## All trejectories are now projected on all eigenvectors, in two rounds:
#first, projections on "self" (for example nph on nph), then all others.
for initrun in $(seq 0 1 1)
do
for z in $datasets
do
if [[ $z == 'nph' ]]
then
maxnum=25001
elif [[ $z == 'ph' ]]
then
maxnum=15001
else
maxnum=10000
fi
echo 'maxnum' $maxnum
if [[ $initrun -eq 0 ]]
then
mkdir pca_actual_$z
fi
cd pca_actual_$z
cp ../$z/"$z"_cSH2.xtc jointtrj_$z.xtc
for x in $datasets;
do
if [[ ($initrun -eq 0 && $z == $x) || ($initrun -eq 1 && $z != $x) ]]
then
echo 'select ' $initrun $z $x
printf "" > proj_analysis_"$z"_"$x".txt
cp ../$x/aver_"$x".pdb aver1_$x.pdb
cp ../$x/eigenval_"$x".xvg eigenval_$x.xvg
cp ../$x/eigenvec_"$x".trr eigenvec_$x.trr
cp ../$x/covar_"$x".log covar_$x.log


for y in $(seq 1 1 10)
do
#do the projections on the joint trajectories
printf "1\n1\n" | g_anaeig -s aver1_"$x".pdb -f jointtrj_"$z".xtc -eig eigenval_"$x".xvg -v eigenvec_"$x".trr -extr
extreme_"$x"_$y.pdb -first $y -last $y
printf  "0\n0\n"  |  g_anaeig  -s  aver1_$x.pdb  -f  jointtrj_$z.xtc  -eig  eigenval_$x.xvg  -v  eigenvec_$x.trr  -proj
proj_"$x"_$y.xvg -split -first $y -last $y

#the results are split to obtain a result for each trajectory. For each trajectory, minimum, maximum and average
projection value are determined. For these three results, an average and standard deviation is calculated and saved
in a results table.
cp proj_"$x"_$y.xvg tempdata.tmp
printf "" > dataout.tmp
grep -v @ tempdata.tmp | grep -v \& | tac > dataout.tmp
for   q   in   $(seq   1   5000   $maxnum);   do   p=$(($q+5000));   sed   "$q"','"$p""   !   d"   dataout.tmp   |   awk
'BEGIN{a=0;b=100000000;c=0;d=0}{a+=1;d+=$2;if($2<b){b=$2};if($2>c){c=$2}}END{printf "%.4f %4f %4f\n", b, c, d/a}';
done > test.log
cp test.log test_"$x"_"$z".log
echo $z $x $y $(awk -v e=$(awk 'BEGIN{a=0;b=0;c=0;d=0}{a+=1;b+=$1}END{printf "%.6f\n", b/a}' test.log) -v f=$(awk
'BEGIN{a=0;b=0;c=0;d=0}{a+=1;b+=$2}END{printf        "%.6f\n",        b/a}'        test.log)        -v        g=$(awk
'BEGIN{a=0;b=0;c=0;d=0}{a+=1;b+=$3}END{printf   "%.6f\n",   b/a}'   test.log)   'BEGIN{a=0;b=0;c=0;d0}{a+=1;b+=($1-
e)**2;c+=($2-f)**2;d+=($3-g)**2}END{printf "%.5f %.5f %.5f %.5f %.5f %.5f\n", e, sqrt(b/a),f,sqrt(c/a),g,sqrt(d/a)}'
test.log) >> proj_analysis_"$z"_"$x".txt

done

fi
done

cd $cwd
done
done

############# collection of data and output preparation ########
cp -rp ../cSH2full ./
mkdir colldat
cd colldat
for t in $datasets
do
s='../cSH2full/'"$t"'_cSH2_x'
r='../'"$t"'/'"$t"
printf "r""$stres""-790\nq\n" | make_ndx -f "$s".gro -o "$r"_CSH2x.ndx
printf "10\n" | trjconv -f "$s".gro -s "$s".gro -n "$r"_CSH2x.ndx -o "$r"_CSH2x.gro
printf "1\n" | trjconv -f "$r"_CSH2x.gro -s "$r"_CSH2x.gro -o "$r"_CSH2x.pdb

for v in $(seq 1 1 10);
do
```

```
## the extreme structures for each eigenvector have been calculated. For better illustration, the RMSD (revealing the regions which are most different between the extreme structures) is
#determined. A structure with sidechains (for illustrative purposes) is used to write the RMSD into the B factor column.
printf "1\n" | g_rmsf -s "$r"_cSH2.gro -f ../pca_actual_$t/extreme_"$t"_"$v".pdb -oq ../$t/temp.pdb -res; sed "s/ 1.00/  1.00 /g" ../$t/temp.pdb > "$r"_cSH2_ex_"$v".pdb
for a in $(seq $stres 1 790);
do
grep ^"ATOM" "$r"_CSH2x.pdb | grep '   '"$a"'   ' | sed 's/1.00  0.00/1.00  '"$(grep ^"ATOM" "$r"_cSH2_ex_"$v".pdb | grep '   '"$a"'   ' | sed "1 ! d" | awk '{printf "%.2f\n", $10}')"'/g'
done > "$t"_ex_"$v".pdb
done
done
cwd=$(pwd)
for initrun in $(seq 0 1 1)
do
for y in $datasets
do
mkdir $y
for x in $datasets
do
if [[ ($initrun -eq 0 && $y == $x) || ($initrun -eq 1 && $x != $y) ]]
then
cp ../pca_actual_"$x"/proj_analysis_"$x"_"$y".txt ./$y/

cd ./$y
c=$y
d=$x
## This routine filters the projection analysis results to reveal where projections are "significantly" different. Significance not in the statistical sense.
if [[ $x != $y ]]
then
a=proj_analysis_"$c"_"$c".txt; b=proj_analysis_"$d"_"$c".txt; for x in $(seq 1 1 $(cat $a | wc | awk '{print $1}')); do h=$(sed "$x ! d" $a | awk '{print $4}'); i=$(sed "$x ! d" $a | awk '{print $5}'); j=$(sed "$x ! d" $a | awk '{print $6}'); k=$(sed "$x ! d" $a | awk '{print $7}'); l=$(sed "$x ! d" $a | awk '{print $8}'); m=$(sed "$x ! d" $a | awk '{print $9}'); cat $b | sed "$x ! d" | awk -v h=$h -v i=$i -v j=$j -v k=$k -v l=$l -v m=$m '{n=0; o=0; p=0; q=0; r=0; s=0; if ($4+$5<h-i){n=$4; o=$5}; if ($4-$5>h+i){n=$4; o=$5}; if ($6+$7<j-k){p=$6; q=$7}; if ($6-$7>j+k){p=$6; q=$7}; if ($8+$9<l-m){r=$8; s=$9}; if ($8-$9>l+m){r=$8; s=$9}; printf "%i %.5f %.5f %.5f %.5f %.5f %.5f\n", $3, n, o,p,q,r,s}'; done > TRAJ_"$d"_on_EVset_"$c"_RES"$d"; b=proj_analysis_"$c"_"$c".txt; a=proj_analysis_"$d"_"$c".txt; for x in $(seq 1 1 $(cat $a | wc | awk '{print $1}')); do h=$(sed "$x ! d" $a | awk '{print $4}'); i=$(sed "$x ! d" $a | awk '{print $5}'); j=$(sed "$x ! d" $a | awk '{print $6}'); k=$(sed "$x ! d" $a | awk '{print $7}'); l=$(sed "$x ! d" $a | awk '{print $8}'); m=$(sed "$x ! d" $a | awk '{print $9}'); cat $b | sed "$x ! d" | awk -v h=$h -v i=$i -v j=$j -v k=$k -v l=$l -v m=$m '{n=0; o=0; p=0; q=0; r=0; s=0; if ($4+$5<h-i){n=$4; o=$5}; if ($4-$5>h+i){n=$4; o=$5}; if ($6+$7<j-k){p=$6; q=$7}; if ($6-$7>j+k){p=$6; q=$7}; if ($8+$9<l-m){r=$8; s=$9}; if ($8-$9>l+m){r=$8; s=$9}; printf "%i %.5f %.5f %.5f %.5f %.5f %.5f\n", $3, n, o,p,q,r,s}'; done > TRAJ_"$d"_on_EVset_"$c"_RES"$c"
fi
rm \#*
rm temp*
rm \#*
cd $cwd
rm \#*
fi
done
cp ./"$y"_ex* ./$y/
done
done
```

S3.3 MD data processing – MutInf post-processing (Python 2.7 script)

```
import csv
import numpy as np
import matplotlib as mpl
import matplotlib.pyplot as plt
import os
from IPython.core.interactiveshell import InteractiveShell
from IPython.core.display import display,HTML
InteractiveShell.ast_node_interactivity = "all"
from scipy import stats
from os import listdir

def getdatalist(path2):
    global fnames,path
    path="/media/hanskoss/data/PLCg1_PhD/NIMR_evacuate/research3/hkoss/md_tandem/new_reanalysis/mutinf/proc/"+path2
    fnames=os.listdir( path )

def prepselection(selection,mina,maxa,minb,maxb):
    for a in selection:
        global abc
        global datlist
        global listx
        with open(path+'/'+fnames[a],'rb') as csvfile:
            datax = csv.reader(csvfile, delimiter=' ')
            i=0
            val = []
            for row in datax:
                val.append(row)
                i+=1
        valnp=np.array(val)
        abc=np.shape(valnp)[0]
        listx=[]
        header=valnp[0][0:abc-1]
        for x in np.arange(1,abc,1):
            listx.append(valnp[x][1:abc])
        datlist=np.array(listx).astype('float64')
        if abc - 1 == maxresxx-minresxx+2:
            newdat=np.zeros([np.shape(datlist)[0]-1,np.shape(datlist)[1]-1])
            delres=783
            newdat[0:delres-minresxx,0:delres-minresxx]=datlist[0:delres-minresxx,0:delres-minresxx]
            newdat[delres-minresxx:maxresxx-minresxx+1,delres-minresxx:maxresxx-minresxx+1]=datlist[delres+1-
minresxx:maxresxx-minresxx+2,delres+1-minresxx:maxresxx-minresxx+2]
            newdat[0:delres-minresxx,delres-minresxx:maxresxx-minresxx+1]=datlist[0:delres-minresxx,delres+1-
minresxx:maxresxx-minresxx+2]
            newdat[delres-minresxx:maxresxx-minresxx+1,0:delres-minresxx]=datlist[delres+1-minresxx:maxresxx-
minresxx+2,0:delres-minresxx]
            datlist=newdat
            abc=np.shape(valnp)[0]-1
        mpl.rcParams['figure.figsize'] = (10,10)

def filterdiag(t,omitdiag):
        u=np.zeros(np.shape(t))
        for x in np.arange(0,np.shape(t)[0]):
            for y in np.arange(np.shape(t)[0]):
                if not x-omitdiag < y or not y < x+omitdiag:
                    u[x,y]=t[x,y]
        return u

def filterhalfdiag(t,whichhalf): #1 upper half
        u=np.zeros(np.shape(t))
        for x in np.arange(0,np.shape(t)[0]):
            for y in np.arange(0,np.shape(t)[0]):
                if whichhalf == 1:
                    if y<=x:
                        u[x,y]=t[x,y]
                else:
                    if y>=x:
                        u[x,y]=t[x,y]
        return u

def plotstuff(mina,maxa,minb,maxb,filenam):
    if abc == maxresxx-minresxx+2:
        datlist0=filterdiag(datlist,5)[::-1]
        b3=mina-minresxx
        b4=maxa-mina+1+b3
        b2=maxresxx-(minb-minresxx)-minresxx+1
        b1=(maxresxx-maxb)+minresxx-minresxx
        a=datlist0[b1:b2,b3:b4]
        totmax=np.max(np.abs(a))
        print "maximum abs value of this plot is " + str(totmax)
        plt.rcParams['figure.facecolor']='white'
        totmax=5.12 #this sets a fixed max/min value for the plot.Comment out when needed.
        plt.imshow(a,   cmap='seismic',   clim=(-totmax,totmax),interpolation='none',extent=[mina-0.5,maxa+0.5,minb-
0.5,maxb+0.5])
        #seismicbwr
        plt.colorbar()
        plt.savefig('/home/hanskoss/mutinfres/'+filenam)
        plt.show()
```

```
def getregionsums(whatever,r1,r2,r3,r4,filtersize):
    xq=filterdiag(whatever,filtersize)[r1-minresxx:r2-minresxx+1,r3-minresxx:r4-minresxx+1]
    #print xq
    x=np.arange(r1,r2+1,1)
    y=np.sum(xq,1)
    return [list(x),list(y)]

def plotsome(whatever,r1,r2,r3,r4,filtersize):

    [x,y]=getregionsums(whatever,r1,r2,r3,r4,filtersize)
    plt.plot(x,y)
    plt.show()
    stuff=[x,y]
    match=np.array(stuff)[0,list(np.argsort(stuff,1)[1])]*(np.array(stuff)[1,list(np.argsort(stuff,1)[1])]>0)
    matchlist=[]
    for x in match[match > 0]: matchlist.append(int(x))
    print np.array(matchlist)
    print np.sort(matchlist)

def preparedata(setname,mina,maxa,minb,maxb,maxcol):
    getdatalist(setname)
    alldata=[]
    for x in np.arange(1,maxcol,1):
        prepselection([x],mina,maxa,minb,maxb)
        alldata.append(datlist)
    return [np.average(alldata,axis=0), np.std(alldata,axis=0)]

def ttestdo(datasetaver1,datasetstd1,statn1,datasetaver2,datasetstd2,statn2,cutoffstat):

[statval,pval]=stats.ttest_ind_from_stats(datasetaver1,datasetstd1*np.sqrt(statn1),statn1,datasetaver2,datasetstd2*
np.sqrt(statn2),statn2,equal_var=False)
    return np.where(~np.isnan(pval),pval,1)<cutoffstat
print 'finished loading definitions'
print 'loading data and calculating...'
minresxx=545; maxresxx=789
mina=minresxx; maxa=maxresxx; minb=minresxx; maxb=maxresxx
[phdat,phdats]=preparedata("phrndcoll",mina,maxa,minb,maxb,10)
[nphdat,nphdats]=preparedata("nphrndcoll",mina,maxa,minb,maxb,10)
[nph687dat,nph687dats]=preparedata("nph687rndcoll",mina,maxa,minb,maxb,10)
[ph687dat,ph687dats]=preparedata("ph687rndcoll",mina,maxa,minb,maxb,10)
nphupdownstat=ttestdo(nphdat,nphdats,10,phdat,phdats,10,0.05)
nph687updownstat=ttestdo(nphdat,nphdats,10,nph687dat,nph687dats,10,0.05)
ph687updownstat=ttestdo(phdat,phdats,10,ph687dat,ph687dats,10,0.05)
datlist=filterhalfdiag(nphdat,1)+filterhalfdiag((phdat-nphdat)*nphupdownstat,0)

print 'producing figures...'
plotstuff(mina,maxa,minb,maxb,'nph_nphud_t.png')
datlist=filterhalfdiag((ph687dat-phdat)*ph687updownstat,0)+filterhalfdiag((nph687dat-nphdat)*nph687updownstat,1)
plotstuff(mina,maxa,minb,maxb,'ph687nphud_687phud_t.png')
datlist=filterhalfdiag(ph687dat,0)+filterhalfdiag(nph687dat,1)
plotstuff(mina,maxa,minb,maxb,'ph687nph_687ph_t.png')
datlist=filterhalfdiag(phdat,0)+filterhalfdiag(nphdat,1)
plotstuff(mina,maxa,minb,maxb,'nph_ph_t.png')
datlist=filterhalfdiag(nphdat,1)+filterhalfdiag((phdat-nphdat)*nphupdownstat,0)
plotstuff(mina,maxa,minb,maxb,'nph_nphud_c.png')
datlist=filterhalfdiag((ph687dat-phdat)*ph687updownstat,0)+filterhalfdiag((nph687dat-nphdat)*nph687updownstat,1)
plotstuff(mina,maxa,minb,maxb,'ph687nphud_687phud_c.png')
datlist=filterhalfdiag(ph687dat,0)+filterhalfdiag(nph687dat,1)
plotstuff(mina,maxa,minb,maxb,'ph687nph_687ph_c.png')
datlist=filterhalfdiag(phdat,0)+filterhalfdiag(nphdat,1)
plotstuff(mina,maxa,minb,maxb,'nph_ph_c.png')
print 'finished'
```

S3.4 K_D determination – NMR data post-processing (Python 2.7 script)

```
import csv
import numpy as np
from IPython.core.interactiveshell import InteractiveShell
InteractiveShell.ast_node_interactivity = "all"
from IPython.core.display import display,HTML
import matplotlib as mpl
import matplotlib.pyplot as plt
import os
from os import listdir
from scipy import optimize

#p1 path. can contain files, no folders, which are in alphabetical order matching the concentrations; for example,
#use 0.0.csv, 0.2.csv instead of 0.csv, 0.2.csv. First and last file have to have peaks in identical order.
#other files can't contain extra peaks.
#
fitfunc = lambda p, xb: ((cp+xb*cp+p[0])/(2*cp))-np.sqrt(((cp+xb*cp+p[0])/(2*cp))**2-xb)
conccutoff=0.5  ## cutoff (peptide concentration) at which the fraction of
p0 = [0.07] ## kD guess
selectset=1
if selectset == 1:
```

```
    p1="/media/hanskoss/nethadat/nmrtrans/nmrrec/tombunney/cSH2_special/hPLCg1_cSH2-pY_150710/"
    conclist=[0.0,0.2,0.4,1.0,1.6,1.8,2.0]
    cp=0.432 #1 #432 #protein concentration
    jacknlistset=[[0,1,2],[1,3,4],[1,2,4],[0,3,4],[1,2,3],[0,2,3]] #jackknife sets / sets selected for fitting
    errfunc = lambda p, x1, y1, x2, y2,x3,y3: np.r_[fitfunc(p,x1)-y1,fitfunc(p,x2)-y2,fitfunc(p,x3)-y3]
    finalplot=[0,1,2,3,4]
    titleset='cSH2 (-770) and pY783'
    filetitle='/home/hanskoss/peptidetrans/cSH2_770_pY783.png'
elif selectset == 2:
    p1="/media/hanskoss/nethadat/nmrtrans/nmrrec/tombunney/tandem_special/hPLCg1_nSH2cSH2_pY_111110/"
    conclist=[0.0,0.2,0.4,0.7,1.0,1.25]
    cp=0.2
    jacknlistset=[[0,1],[1,2],[0,3],[1,3],[2,3],[0,2]]
    errfunc = lambda p, x1, y1, x2, y2: np.r_[fitfunc(p,x1)-y1,fitfunc(p,x2)-y2]
    finalplot=[0,1,2,3]
    titleset='tandem-SH2 (-790) and pY783'
    filetitle='/home/hanskoss/peptidetrans/tandem_790_pY783.png'
elif selectset ==3:
    cp=0.117
    p1="/media/hanskoss/nethadat/nmrtrans/nmrrec/tombunney/cSH2_special/hPLCg1_cSH2_PDGFR_030112/"
    conclist=[0.0,0.2,0.4,0.6,0.8,1.0,1.2]
    jacknlistset=[[1,2,4],[2,3,4],[0,4,5],[0,2,4],[0,3,5],[2,4,5],[1,3,5],[0,1,3]]
    errfunc = lambda p, x1, y1, x2, y2,x3,y3: np.r_[fitfunc(p,x1)-y1,fitfunc(p,x2)-y2,fitfunc(p,x3)-y3]
    finalplot=[0,1,2,3,4,5]
    titleset='cSH2 (-790) and PDGFR'
    filetitle='/home/hanskoss/peptidetrans/cSH2_790_PDGFR.png'


maxdat=np.shape(conclist)[0]-1 #some useful abbreviation
flatten = lambda l: [item for sublist in l for item in sublist] #flattens lists

#reads and lists path contents, could be improved (folder structure and file naming has to be perfect)
def getdatalist(path2):
    global fnames,path
    path=p1+path2
    fnames=os.listdir( path )

#open CSV file and read in data
def prepselection(selection):
    for a in selection:
        colselect=[0,2,3,6,7,8]
        global abc
        global datlist
        global listx
        with open(path+'/'+fnames[a],'rb') as csvfile:
            datax = csv.reader(csvfile, delimiter=' ')
            i=0
            val = []
            for row in datax:
                val.append(row)
                i+=1
        valnp=np.array(val)
        np.shape(np.array(valnp))
        abc=np.shape(valnp)[0]
        listx=[]
        header=valnp[0]
        for x in np.arange(1,abc,1):
            listx.append(valnp[x][colselect])
        datlist=np.array(listx).astype('float64')
        return datlist, header[colselect], fnames[a]

#expands certain elements to a list of repeating elements for shape and accessibility reasons
def expfillist(num):
    Posfnx=[]; statex=[]
    for a in np.arange(0,np.shape(datcoll[num]['Position_F1'])[0]):
        Posfnx.append(datcoll[num]['file'])
        statex.append(num)
    return np.array(Posfnx), np.array(statex)

#useful to find all elements equal to a in list lst
def find(lst, a):
    return [i for i, x in enumerate(lst) if x==a]

#start here
getdatalist('data')

#after reding in data, create dictionary with peak list and other information.
datcoll=[]
for x in np.arange(np.shape(fnames)[0]):
    datcoll.append({})
    datcoll[x]['data']=prepselection([x])[0]
    datcoll[x]['header']=prepselection([x])[1]
    datcoll[x]['file']=prepselection([x])[2]
    datcoll[x]['conc']=conclist[x]
    for y in np.arange(np.shape(datcoll[x]['header'])[0]):
        datcoll[x][datcoll[x]['header'][y]]=datcoll[x]['data'][:,y]

#Creation of a reference peak dictionary to easily access information for each reference peak.
#reference peaks are peaks at the lowest and the highest concentration (have to be alphabetically
#at the beginning and the end of the folder.)
PosF1ref=datcoll[0]['Position_F1']
```

```
Posfn=expfillist(0)[0]; Posstat=expfillist(0)[1]
PosF1ref=np.append(PosF1ref,datcoll[maxdat]['Position_F1'])
PosF2ref=datcoll[0]['Position_F2']
PosF2ref=np.append(PosF2ref,datcoll[maxdat]['Position_F2'])
Posvol=datcoll[0]['Volume']
Posvol=np.append(Posvol,datcoll[maxdat]['Volume'])
Posfn=np.append(Posfn,expfillist(maxdat)[0])
Posstat=np.append(Posstat,expfillist(maxdat)[1])
Posres=datcoll[0]['Number']
Posres=np.append(Posres,datcoll[maxdat]['Number'])
Posref={}
Posref['shifts']=np.transpose(np.array([PosF1ref,PosF2ref]))
Posref['files']=Posfn;Posref['residue']=Posres;Posref['state']=Posstat;Posref['volume']=Posvol

# This goes through all peak lists and finds the reference peak which is closest to the peak in question.
for a in np.arange(np.shape(datcoll)[0]):
    datcoll[a]['refpeak']=[]
    for b in np.arange(np.shape(datcoll[a]['Position_F1'])[0]):
        datcoll[a]['refpeak'].append(np.argmin((datcoll[a]['Position_F1'][b]-
Posref['shifts'][:,0])**2+(datcoll[a]['Position_F2'][b]-Posref['shifts'][:,1])**2))

# This routine goes through the reference peak lists and scans all other peak lists for matching peaks (the
# matches have been determined in the previous routine. It fills a "matched" dictionary for each reference peak
# so that all relevant peaks for a certain concentration, residue and state are collected.
Posref['matched']=[]
for a in np.arange(np.shape(Posref['volume'])[0]):
    Posref['matched'].append([])
    Posref['matched'][a]={}
    matchingx=[]; volx=[]; shiftx=[]; filex=[];concx=[]
    for b in np.arange(np.shape(datcoll)[0]):
        for c in np.arange(np.shape(datcoll[b]['Position_F1'])[0]):
            if a == datcoll[b]['refpeak'][c]:
                matchingx.append([b,c])
                shiftx.append([datcoll[b]['Position_F1'][c],datcoll[b]['Position_F2'][c]])
                volx.append(datcoll[b]['Volume'][c])
                filex.append(datcoll[b]['file'])
                concx.append(datcoll[b]['conc'])
    Posref['matched'][a]['shifts']=shiftx
    Posref['matched'][a]['volume']=volx
    Posref['matched'][a]['position']=matchingx
    Posref['matched'][a]['file']=filex
    Posref['matched'][a]['conc']=concx

# The following routine checks whether there are peak volume data for a given residue at a given
# concentration for the bound and the unbound state. The fraction of the bound state for each residue and
# concentration is then determined. This can in principle be modified to merge information from the bound
# and the unbound peaks.
collectx=[];collecty=[]
for a in list(np.unique(Posref['residue'])):
    x=[];y=[]
    for b in conclist:
        state0pos=list(set(find(Posref['residue'], int(a))).intersection(find(Posref['state'], 0)))[0]
        state1pos=list(set(find(Posref['residue'], int(a))).intersection(find(Posref['state'], maxdat)))[0]
        vol0=Posref['volume'][state0pos]
        vol3=Posref['volume'][state1pos]
        try:
            st0pos2=find(Posref['matched'][state0pos]['conc'],b)[0]
            vol1=Posref['matched'][state0pos]['volume'][st0pos2]
            if b < conccutoff:
                x.append(b);
                y.append(1-vol1/vol0)
        except:
            pass
        try:
            st1pos2=find(Posref['matched'][state1pos]['conc'],b)[0]
            vol2=Posref['matched'][state1pos]['volume'][st1pos2]
            if b >= conccutoff:
                x.append(b);
                y.append(vol2/vol3)
        except:
            pass
    collectx.append(np.array(x));collecty.append(np.array(y))

#jackknife fits and plots
plt.rcParams.update({'font.size': 14})
p1collect=[]
for jacknlist in jacknlistset:
    guru3=[]
    for x in jacknlist:
        guru3.append(collectx[x]); guru3.append(collecty[x])
    guru4=tuple(guru3)
    p1,success = optimize.leastsq(errfunc, p0, args=guru4) #args=(Tx, tX, Ty, tY)
    p1collect.append(p1)
    num_points = 100
    x_dat = np.linspace(0, 2, num_points)
    y_dat = fitfunc(p1,x_dat)
    plt.clf()
    colorlist=['k','r','g','b','cyan','m','y']
    cnt=0
    for bx in jacknlist: #np.arange(np.shape(np.unique(Posref['residue']))[0]):
        a=plt.figure(2)
```

```
a=plt.plot(list(collectx[bx]),list(collecty[bx]),marker='x',linestyle='None',markersize=10,c=colorlist[cnt])
        a=plt.xlabel('peptide/protein ratio')
        a=plt.ylabel('bound fraction')
        cnt+=1
    a=plt.plot(x_dat,y_dat,marker='None',linestyle='-',color='k')
    a=plt.show()
print 'kD is ' + str(1000*np.average(p1collect))+' uM +- ' + str(1000*np.std(p1collect))

cnt=0
for bx in finalplot: #np.arange(np.shape(np.unique(Posref['residue']))[0]):
    a=plt.figure(2)
    a=plt.plot(list(collectx[bx]),list(collecty[bx]),marker='x',linestyle='None',markersize=10,c=colorlist[cnt])
    a=plt.xlabel('peptide/protein ratio')
    a=plt.ylabel('bound fraction')
    cnt+=1
num_points = 100
x_dat = np.linspace(0, 2, num_points)
y_dat = fitfunc([np.average(p1collect)],x_dat)
a=plt.title(titleset)
a=plt.plot(x_dat,y_dat,marker='None',linestyle='-',color='k')
a=plt.savefig(filetitle)
a=plt.show()
```

Supporting Figures

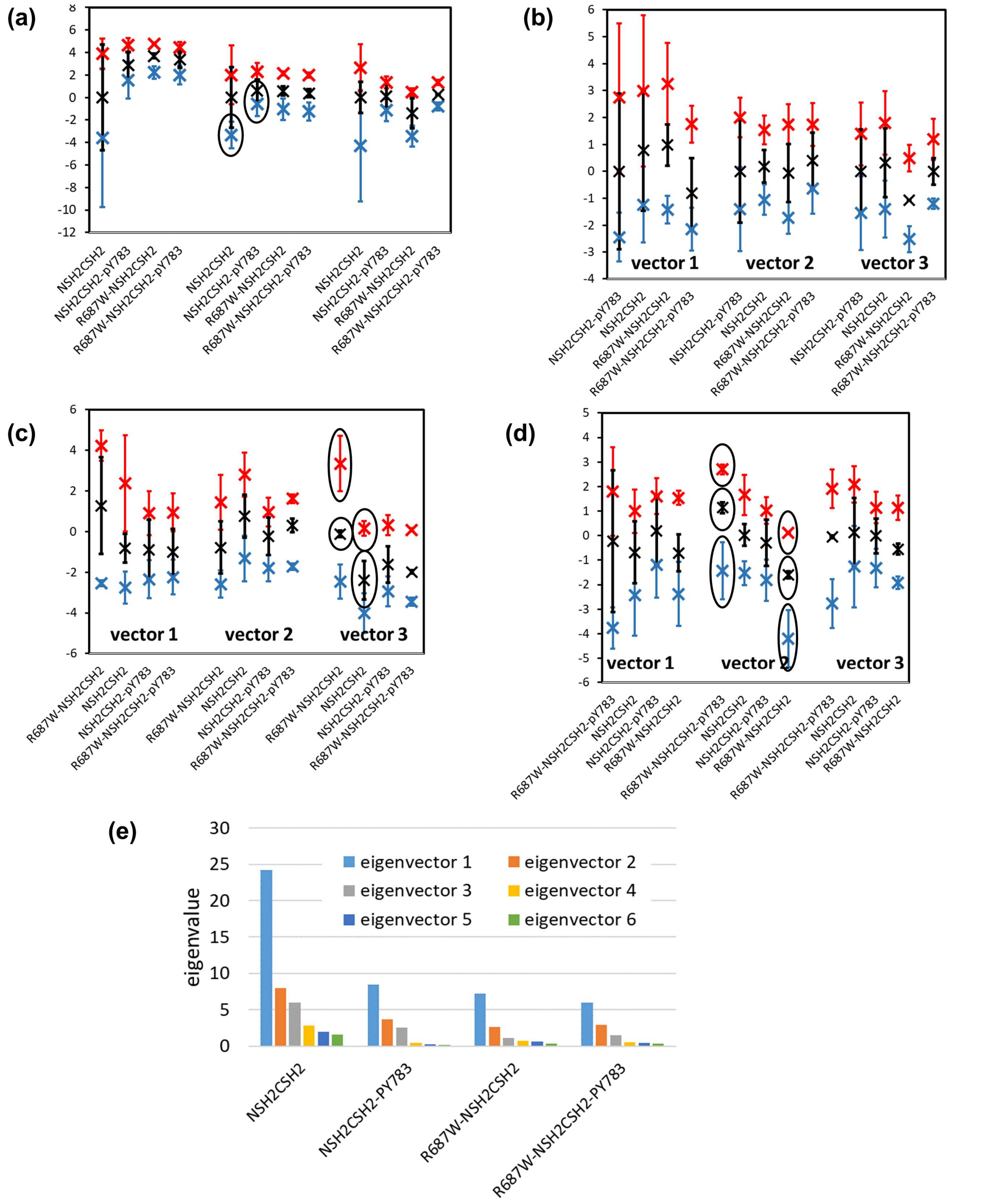

Figure S1. Differences between NSH2CSH2, NSH2CSH2-pY^{783}, R687W-NSH2CSH2 and R687W-NSH2CSH2-pY^{783} molecular dynamics trajectories revealed by principal component analysis.

a-d: Projections of trajectories on PCA eigenvectors reveal differences between trajectories, represented by the eigenvectors. Eigenvectors were generated from pooled trajectories (50-100 ns) for each of the four constructs: (a) NSH2CSH2, (b) NSH2CSH2-pY^{783}, (c) R687W-NSH2CSH2 and (d) R687W-NSH2CSH2-pY^{783}. The projection of any trajectory on any eigenvector covers a certain range

(which varies for each trajectory). Rather than this range, the upper and lower limit of this range as well as the average, along with the errors for these limits, are shown for some of the projections. The upper and lower range limits and the average were calculated by projecting the different individual trajectories of a construct on the eigenvector in question (giving average and standard deviation for both upper and lower limit).

Example for the PCA of NSH2CSH2 - eigenvector 2 (panel a): The 1D projection of the NSH2CSH2-pY^{783} trajectories on this eigenvector is different from the NSH2CSH2 projection - the lower limit of the projection range (circled) does not overlap with the projection of NSH2CSH2. This analysis reveals that NSH2CSH2 and NSH2CSH2-pY^{783} can be expected to differ for eigenvector 2 of the NSH2CSH2 PCA. Statistical significance is not implied, but based on this analysis the (mostly C-terminal and pre-C-terminal) motion represented by this eigenvector (*Fig. 2a*) is realized to different extents between these two constructs. Other relevant projections to describe differences between WT and ARG687TRP constructs are marked in panels (c) and (d).

(e) The magnitude of the first six eigenvalues obtained from the PCA for each of the four MD trajectories. Example: the first three eigenvalues correspond to the three eigenvectors on which various trajectories are projected in panel (a). NSH2CSH2 eigenvector 2 obtained from pooled NSH2CSH2 data describes a motion which differs between NSH2CSH2 and NSH2CSH2-pY^{783}. The eigenvalue corresponding to this eigenvector appears relatively large.

(a)

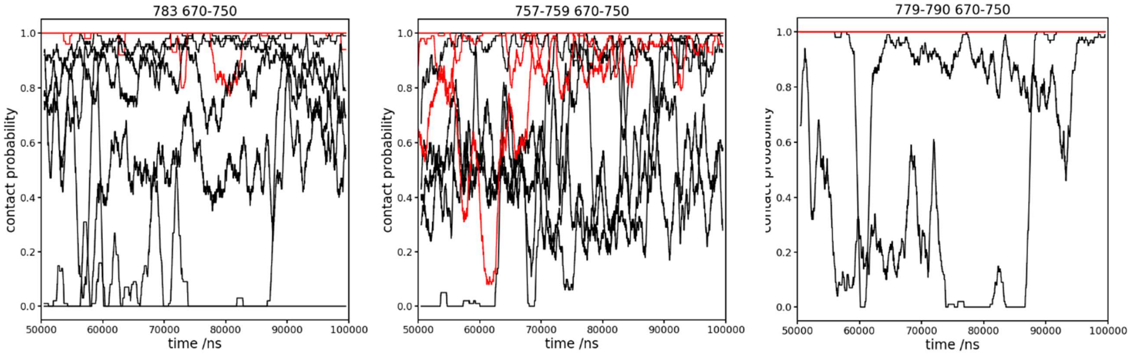

(b)

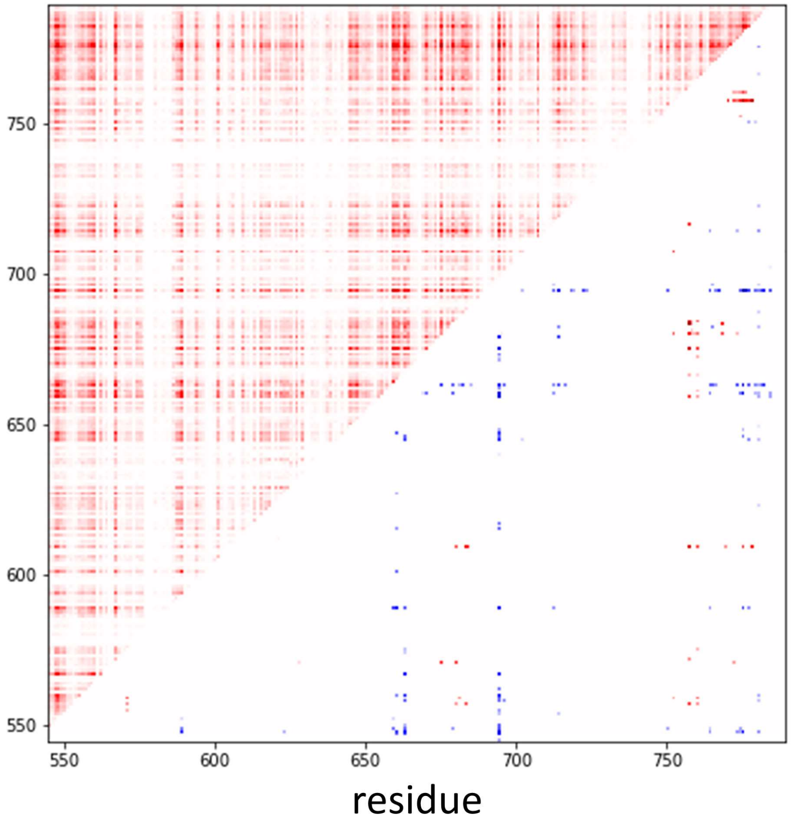

Figure S2. Molecular dynamics simulations predict dynamic differences between NSH2CSH2 and NSH2CSH2-pY^{783} for residues in the pre-C-terminus and the C-terminal linker, supplemental contact analysis and mutual information data. (a) Probability of contact between C-terminal linker residues and the cSH2 domain, shown for different MD trajectories. Color key (numbers of available trajectories in brackets): black – NSH2CSH2 (n = 6 trajectories); red – NSH2CSH2-pY^{783} (n = 4). Any number of contacts (< 2.5 Å) at a given time point is counted as a single contact event, yielding a contact probability for any 1 ns sliding average time bin. (b) Mutual information between side chain dihedrals of NSH2CSH2 and NSH2CSH2-pY783 trajectories. See *Fig. 2b* for details and labels. The matrix is shown here for the entire construct, including the nSH2 domain.

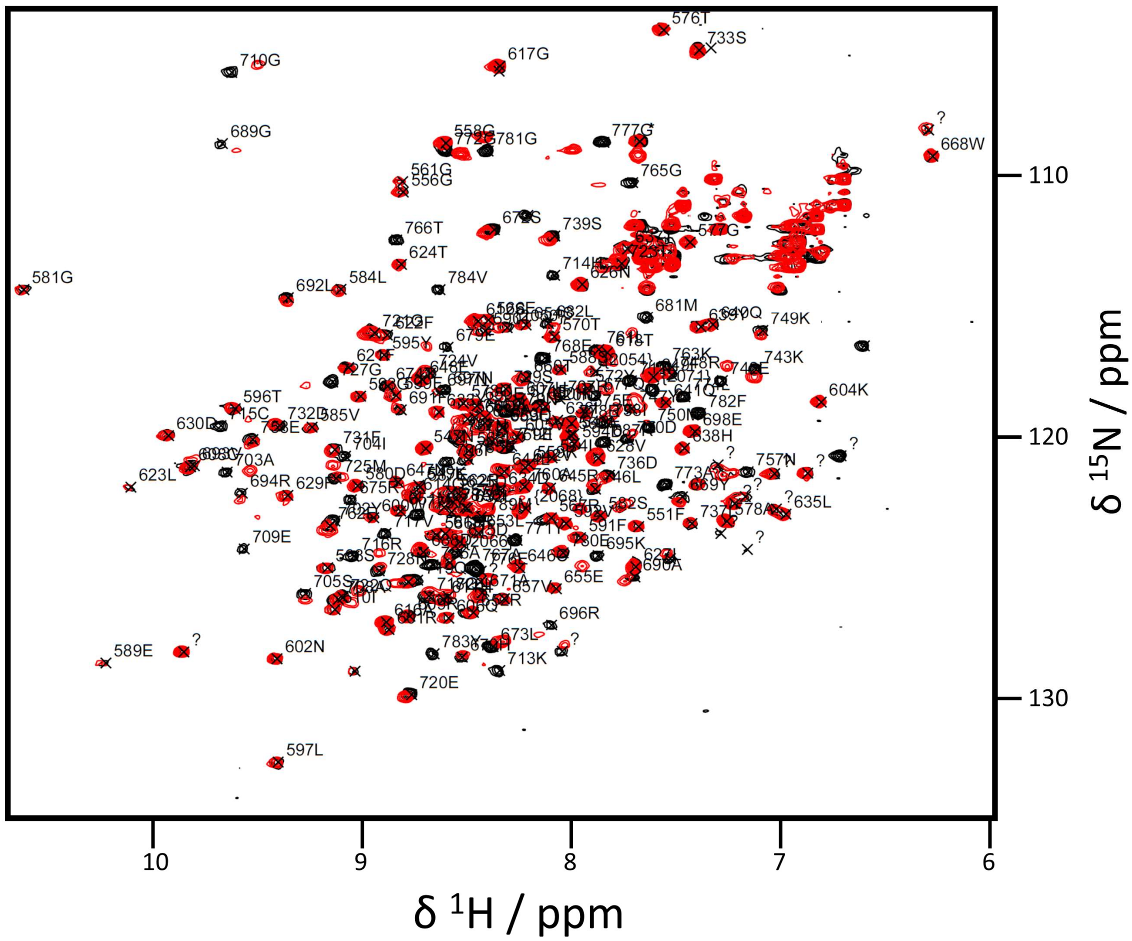

Figure S3: ^{1}H,^{15}N-HSQC spectra of NSH2CSH2 (red) and NSH2CSH2-pY^{783} (black). Spectra were recorded at 700 MHz. For more extensive backbone assignment information refer to the relevant BMRB depositions (BMRB entry 27496).

(a)

construct	MW / kDa	conc / mg ml^{-1}	deleted points	R_g / nm	I_0	D_{max} / nm	Vol_{excl} / nm^3	back-calc. MW / kDa
NSH2CSH2-pY^{783}	28.5	5.8	32	2.43±0.20	21.83±0.05	8	38	21
	28.5	4.1	28	2.38±0.10	21.18±0.05	8	39	21
	28.5	2.2	34	2.27±0.07	20.03±0.08	7	40	20
NSH2CSH2	28.5	5.8	26	2.76±0.29	26.57±0.06	9	42	26
	28.5	4.0	31	2.62±0.18	24.43±0.07	9	40	24
	28.5	2.2	28	2.48±0.29	22.05±0.09	8	39	22

(b)

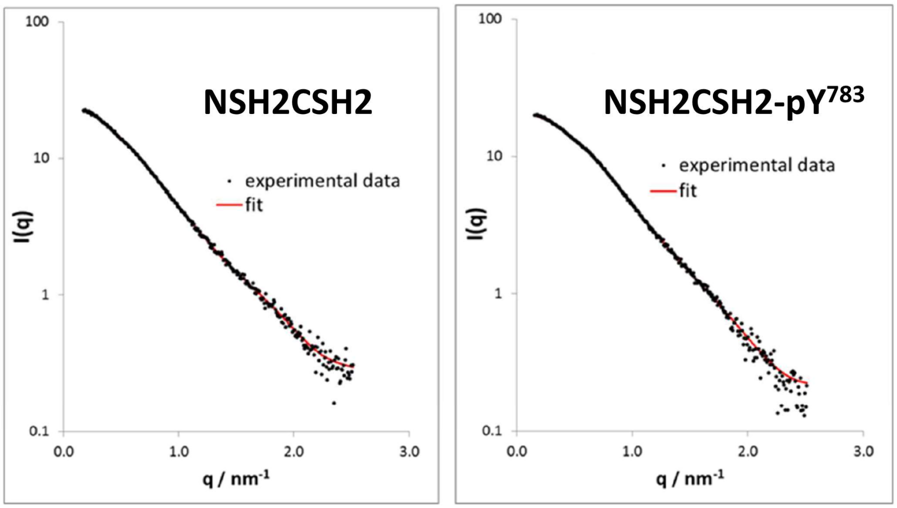

(c)

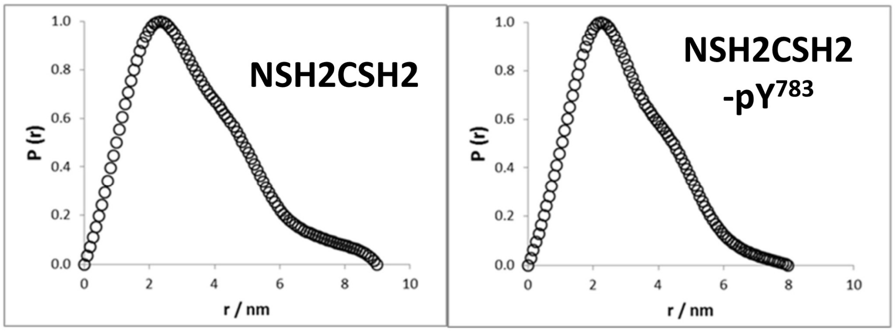

Figure S4: SAXS data recorded for NSH2CSH2 and NSH2CSH2-pY^{783} reveal an aggregation propensity for NSH2CSH2. (a) General parameters extracted from the SAXS data. The molecular weight was back-calculated from I_0 for the construct and I_0 for a reference sample of bovine serum albumin. The gyration radius and volume of NSH2CSH2-pY^{783} is generally lower and more stable upon concentration reduction than the respective values for NSH2CSH2. This might indicate a weak propensity to aggregate for NSH2CSH2. (b) Experimental data and reciprocal fits of the distance distribution functions. (c) Distance distribution functions. The shapes of these curves match to a two-domain protein of the given size. However, the presence of some larger distances, especially for NSH2CSH2, suggests that some aggregation is present.

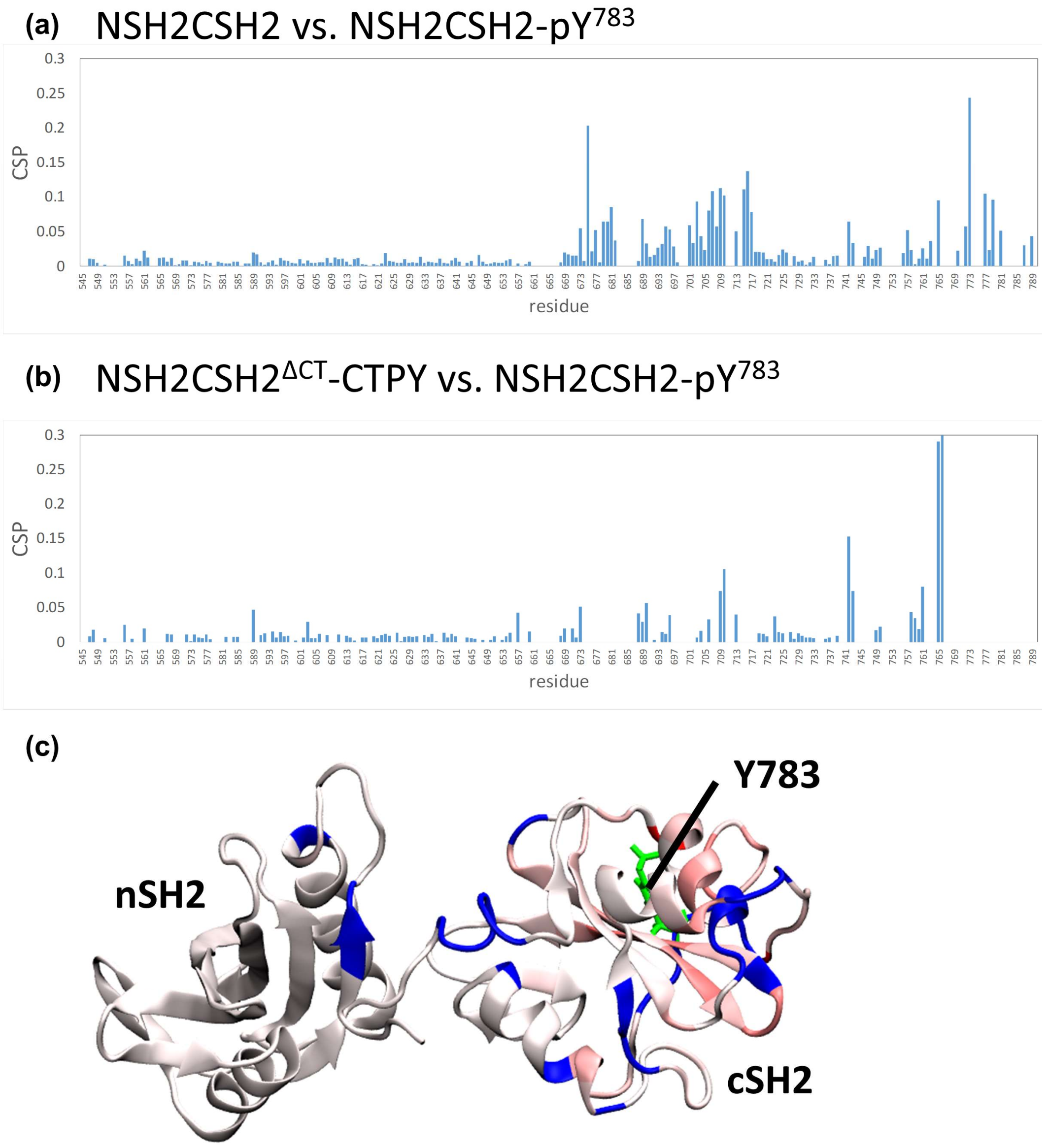

Figure S5: Chemical shift perturbation (CSP) analysis for tandem SH2 constructs in ^{1}H,^{15}N-HSQC spectra (recorded at 700 MHz). (a) and (c): CSPs between NSH2CSH2 and NSH2CSH2-pY^{783} contructs. (b): CSPs between NSH2CSH2$^{\Delta CT}$-CTpY and NSH2CSH2-pY^{783} constructs. (c): Illustration of the CSPs between NSH2CSH2 and NSH2CSH2-pY^{783}, projected on the structural model based on the tandem domain crystal structure 4FBN. Color scheme: white (small CSP) to red (large CSP); blue: no data. The atoms of Tyr783 are highlighted in green.

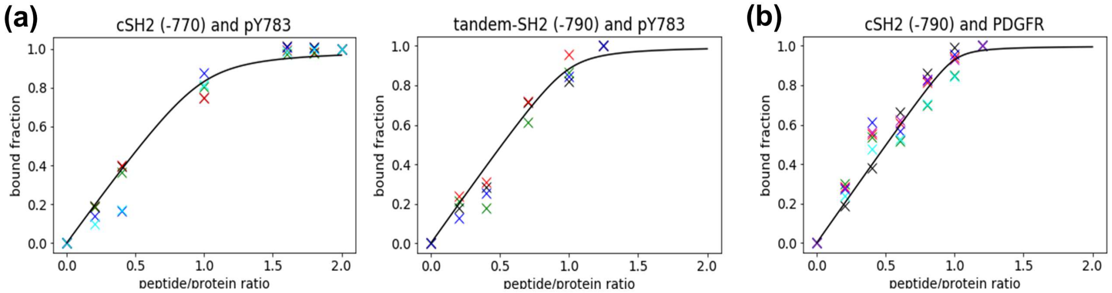

Figure S6: Peptide / protein titrations for K_D determination. The black line was plotted based on the average K_D determined from several jackknife-sampled fits. Experimental data were obtained from the peak volume ratios (bound fraction) for different residues, each plotted here in a different color. For each jackknife-sampled fit, data sets from 2-3 residues were taken for each K_D determination.

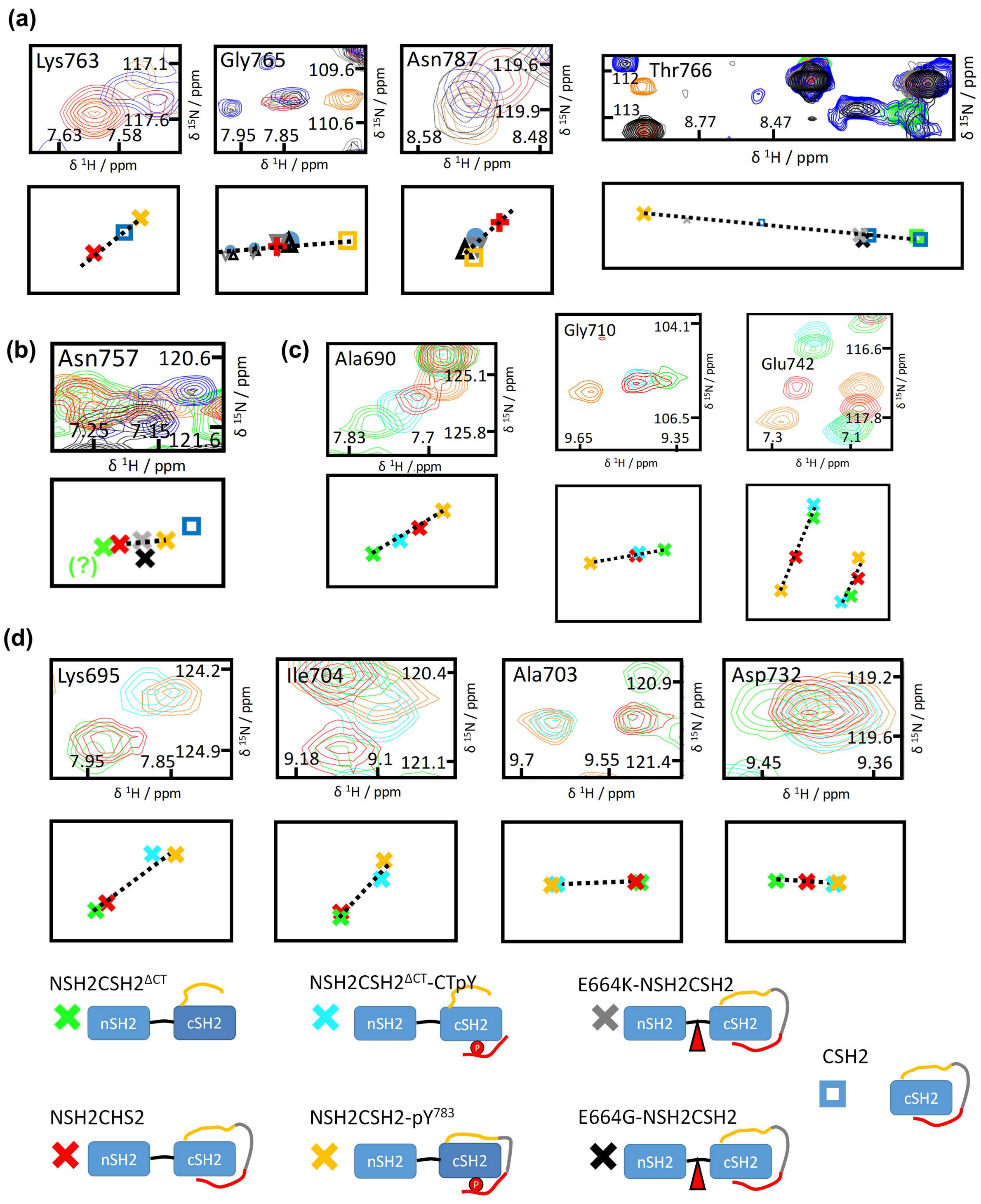

Figure S7: Allosteric pathways connecting the Tyr783 phosphorylation site with the nSH2-cSH2 junction; see also Fig. 4. ^{1}H,^{15}N-HSQC spectra are shown next to supporting symbolic representations of the spectra (panels a and b - 700 MHz; panels c and d - 600 MHz; legend at bottom of figure).

(a) The peaks are shifted towards the 'closed' state when the nSH2 domain is absent (CSH2) or when nSH2-cSH2 junction mutations are present, confirming dynamic allosteric communication via the extended C-terminus (see also *Fig. 4b*).

(b) The cross peak for Asn757 in the CSH2 spectrum is located on an *extension* of the vector connecting the corresponding cross peak positions in the spectra of NSH2CSH2 and NSH2CSH2-pY^{783}. It can be inferred that residue Asn757 in NSH2CSH2-pY^{783} is not fully 'closed' in CSH2. The exact position of the corresponding NSH2CSH2$^{\Delta CT}$ cross peak is uncertain.
(c) For some residues, the cross peaks for NSH2CSH2-pY^{783} and NSH2CSH2$^{\Delta CT}$-CTpY do not coincide, indicating a C-terminal linker-dependent effect (see also *Fig. 4a*).
(d) For some residues, especially those located in the β-sheet, the peaks for NSH2CSH2-pY^{783} and NSH2CSH2$^{\Delta CT}$-CTpY coincide, indicating a C-terminal linker-*in*dependent effect.

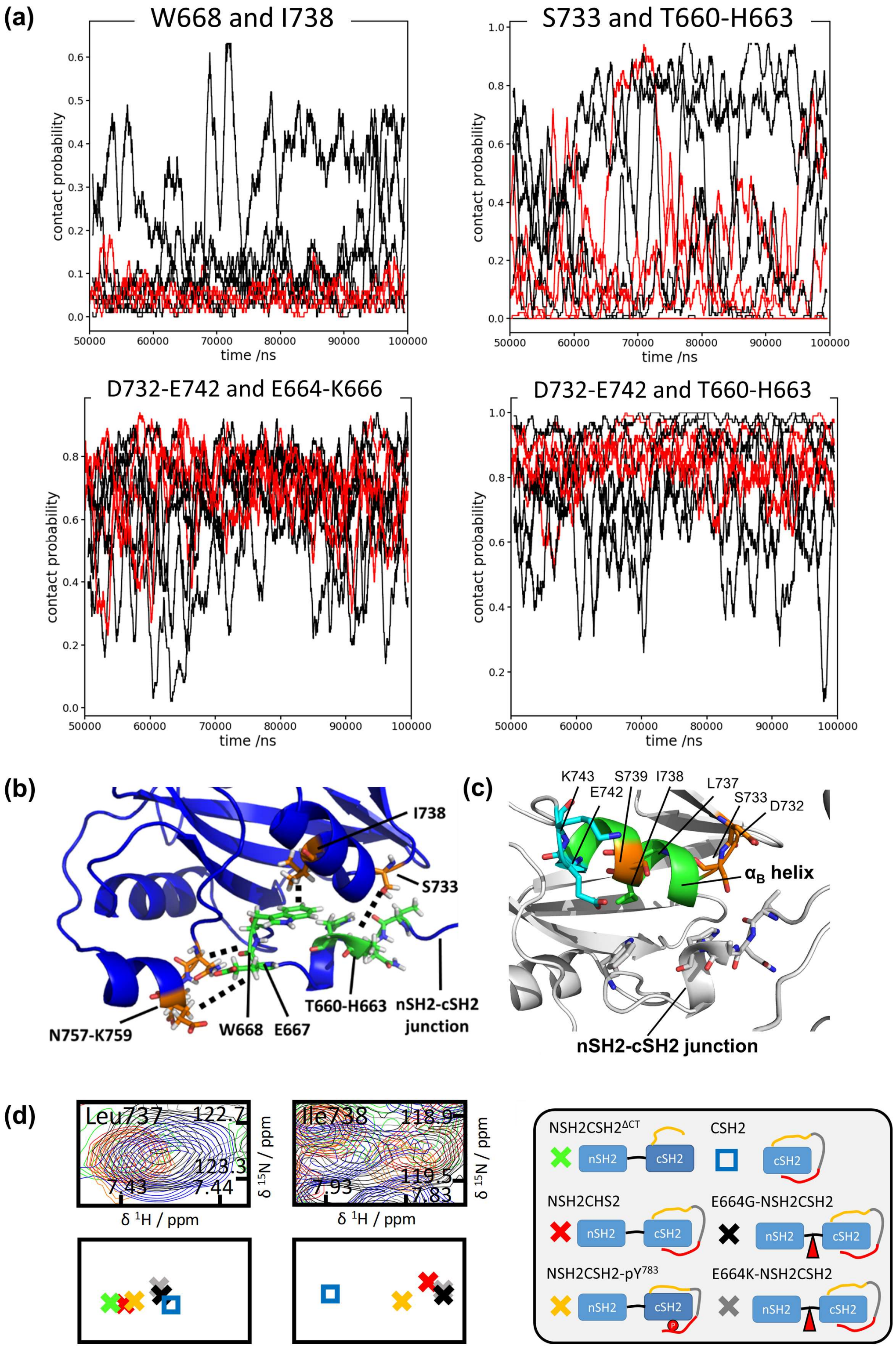

Figure S8: Contacts between the nSH2-cSH2 junction and the α_B helix and the extended C-terminus. (a) Contact probabilities between α_B helix and nSH2-cSH2 junction, shown for different MD trajectories. Color key (numbers of available trajectories in brackets): black, NSH2CSH2 (6); red, NSH2CSH2-pY^{783} (4). Any number of contacts (< 2.5 Å) at a given time point is counted as a single contact event, yielding the contact probability over a 1 ns sliding average time bin.

(b) This cSH2 model (based on the 4FBN structure) shows residues and contacts that were found to be relevant for the interaction between the nSH2-cSH2 junction with the pre-C-terminus and the α_B helix.
(c) Depiction of part of the cSH2 domain structure illustrating the location of α_B residues discussed in the main text: cyan, residues whose chemical shifts are perturbed mostly by the C-terminal linker-dependent allosteric pathway; orange, residues whose peaks are perturbed mostly by the C-terminal linker-independent pathway.
(d) Impact of the presence or modification of the nSH2-cSH2 junction on α_B helix residues Leu737 and Ile738. $^{1}H,^{15}N$-HSQC spectra are shown next to supporting symbolic representations of the spectra (700 MHz, color key shown on the right).

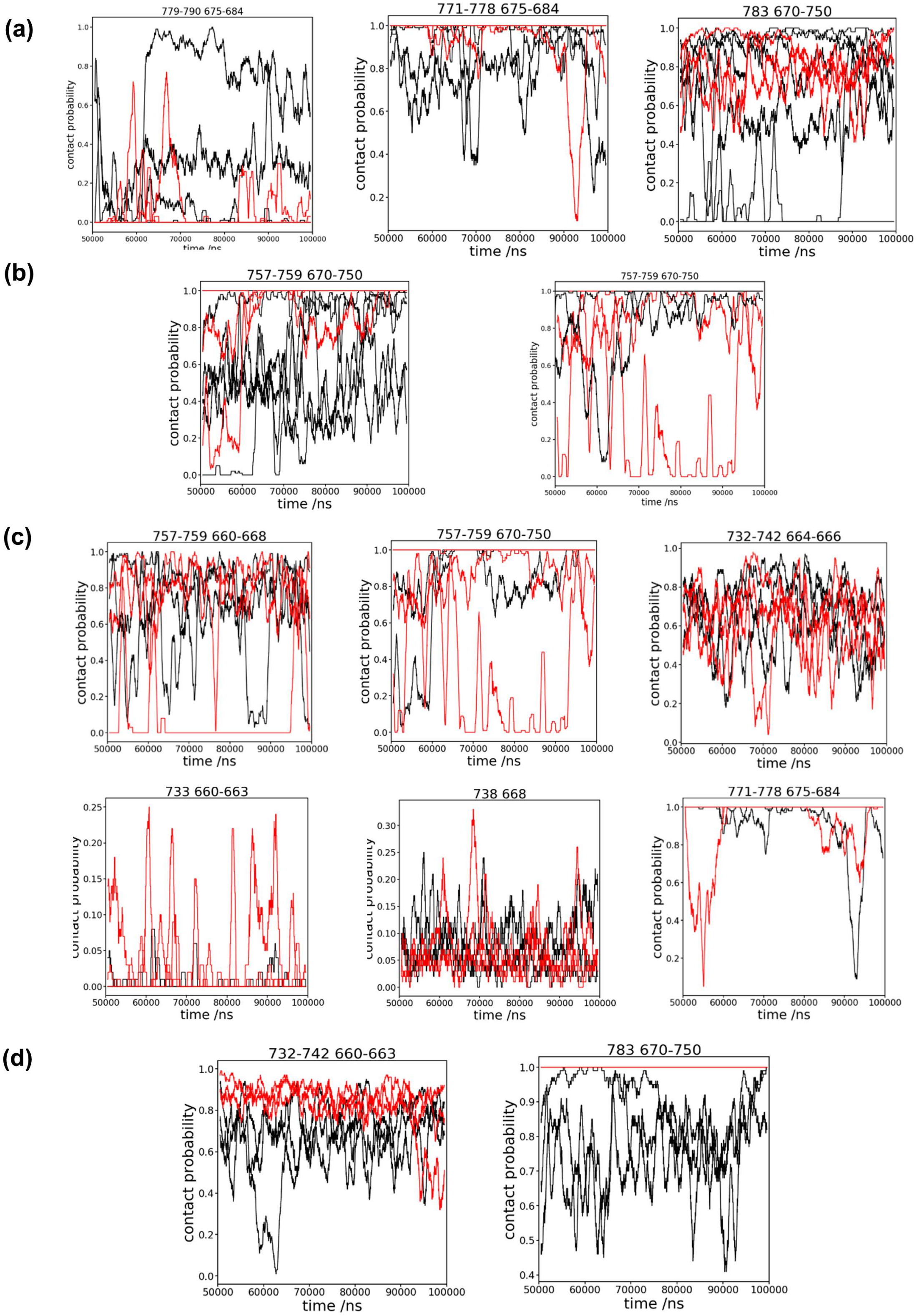

Figure S9: MD contact analyses for Arg687Trp mutant constructs. Contact probabilities are shown for different MD trajectories. The number of region pairs which have clearly different contact probabilities between nonphosphorylated and phosphorylated Arg687Trp states is much lower than for the WT. Any number of contacts (< 2.5 Å) at a given time point is counted as a single contact event, yielding a contact probability over a 1 ns sliding average time bin. Labels at the top of the figure

indicate the specific contact regions that were evaluated. (a) Comparison of NSH2CSH2 (black) with R687W-NSH2CSH2 (red) constructs, contacts between C-terminal regions and cSH2 domain regions. (b) Contacts between pre-C-terminus and cSH2 domain. left: Comparison of NSH2CSH2 (black) with R687W-NSH2CHS2 (red); right: comparison of NSH2CHS2-pY^{783} (black) with R687W-NSH2CSH2-pY^{783} (red). (c) Comparison of R687W-NSH2CSH2 (black) with R687W-NSH2CSH2-pY^{783} (red): differencesdo not appear to be significant based on inspection of the trajectories; much clearer differences were shown for the respective WT contact pairs as shown in other figures. (d) Comparison of R687W-NSH2CSH2 (black) with R687W-NSH2CSH2-pY^{783} (red); differences appear to be significant based on inspection of the trajectories.

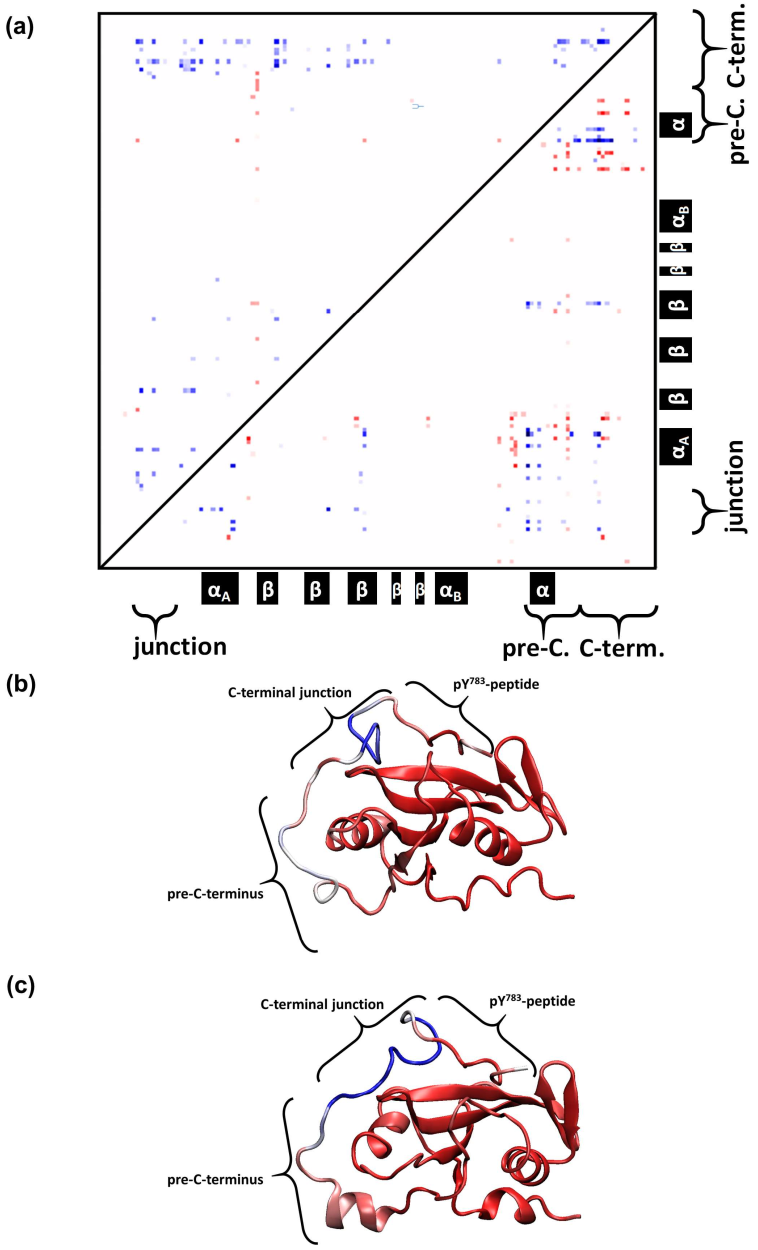

Figure S10: Molecular dynamics simulations predict dynamic differences between WT and Arg687Trp constructs for residues in the pre-C-terminus and the C-terminal linker. (a) Mutual side chain dihedral information, differences between WT and Arg687Trp mutants. The filtered results (t-test) for the cSH2 domain are shown in this panel. Upper left half: mutual information difference between NSH2CSH2 and R687W-NSH2CSH2. Lower right half: mutual information difference between NSH2CSH2-pY^{783} and R687W-NSH2CSH2-pY^{783}. Red indicates an increase in mutual information in the mutated construct; blue, decrease in mutual information. (b-c) RMS fluctuation of Arg687Trp eigenvectors: (b) R687W-NSH2CSH2-pY^{783} eigenvector 2; (c) R687W-NSH2CSH2 eigenvector 3 containing information about differences in backbone motions in the cSH2 domain between Arg687Trp and WT proteins (see also *Fig. 2b* and *Fig. S1*).

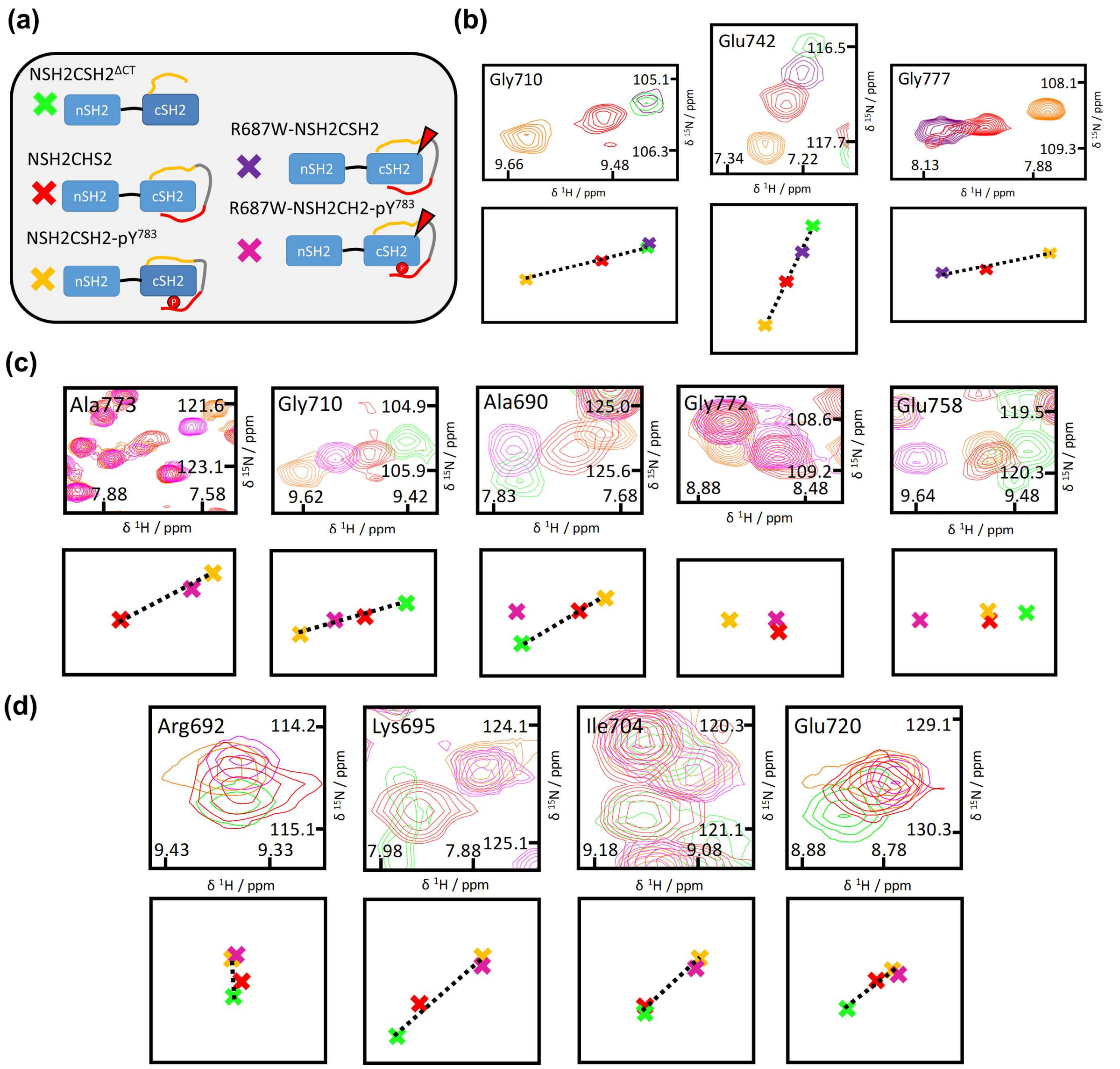

Figure S11: Specific effect of the Arg687Trp mutation on the C-terminal linker dependent pathway (additional examples, linked to *Fig. 6*).

(a) Key for panels (b-d), showing the correspondances between constructs and symbols/colors.

(b) Similar to the situation in R687W-NSH2CSH2-pY^{783}, the cross peaks for many residues in R687W-NSH2CSH2 are shifted toward the 'closed' (C) state with respect to WT constructs. ^{1}H,^{15}N-HSQC spectra were recorded at 950 MHz (R687W-NSH2CSH2) or 700 MHz (all others).

(c) Cross peaks for residues associated mostly with the C-terminal linker-dependent pathway often display large chemical shift perturbations when comparing Arg687Trp constructs with WT constructs. ^{1}H,^{15}N-HSQC spectra were recorded at 600 MHz.

(d) Cross peaks for residues that are mostly associated with the C-terminal linker-independent pathwa, are not or hardly shifted in Arg687Trp constructs with respect to the WT protein. ^{1}H,^{15}N-HSQC spectra were recorded at 600 MHz.

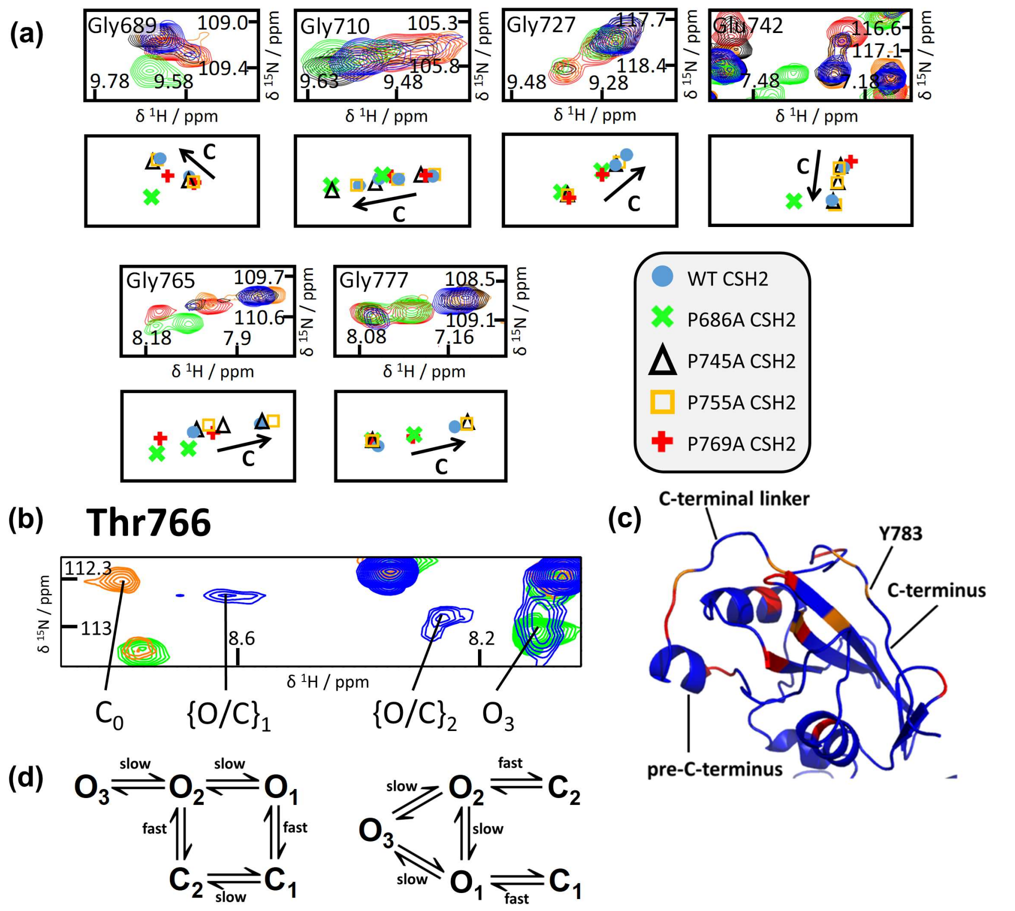

Figure S12: Multistate fast and slow exchange in CSH2 and NSH2CSH2 constructs. (a) Chemical shift analysis comparing $^{1}H,^{15}N$-HSQC spectra (600 MHz) for various CSH2 constructs without or with Pro→Ala mutations (see color key). Pro→Ala mutationsdramatically shift the exchange behavior of multiple residues. The spatially close residues Pro686Ala and Pro769Ala substitutions often tilt the fast dynamic equilibrium towards the 'open' state. The direction of the vector towards the 'closed' state is indicated by the arrow. (b) The CSH2 $^{1}H,^{15}N$-HSQC spectrum (blue) for residue Thr766 shows three cross peaks. One of them is at the same position as that detected for NSH2CSH2$^{\Delta CT}$ (green); in the corresponding O_3 state, the pre-C-terminus is locally 'open'. The 'closed' state is not present for CSH2; the corresponding NSH2CSH2-pY^{783} cross peak (orange) is shown for reference. The other two CSH2 cross peaks represent states which are exchanging rapidly between 'closed' and 'open' forms $\{C \rightleftharpoons O\}_2$; $\{C \rightleftharpoons O\}_3$. The resulting overall kinetic scheme is $\{O \rightleftharpoons^{*} C\}_1 \rightleftharpoons^{\dagger} \{O \rightleftharpoons^{*} C\}_2 \rightleftharpoons^{\dagger} O_3$, where * and † denote fast and slow exchange, respectively (for specific examples, see panel (d)). (c) Presence of slow exchange (cross peak doubling) in CSH2 (projected on the cSH2 domain model derived from the 4FBN crystal structure): red, slow exchange confirmed; orange, slow exchange likely. (d) Kinetic schemes exemplifying specific variations of the general scheme found for residue Thr766, $\{O \rightleftharpoons^{*} C\}_1 \rightleftharpoons^{\dagger} \{O \rightleftharpoons^{*} C\}_2 \rightleftharpoons^{\dagger} O_3$, where * and † denote fast and slow exchange, respectively (see also panel (b)).

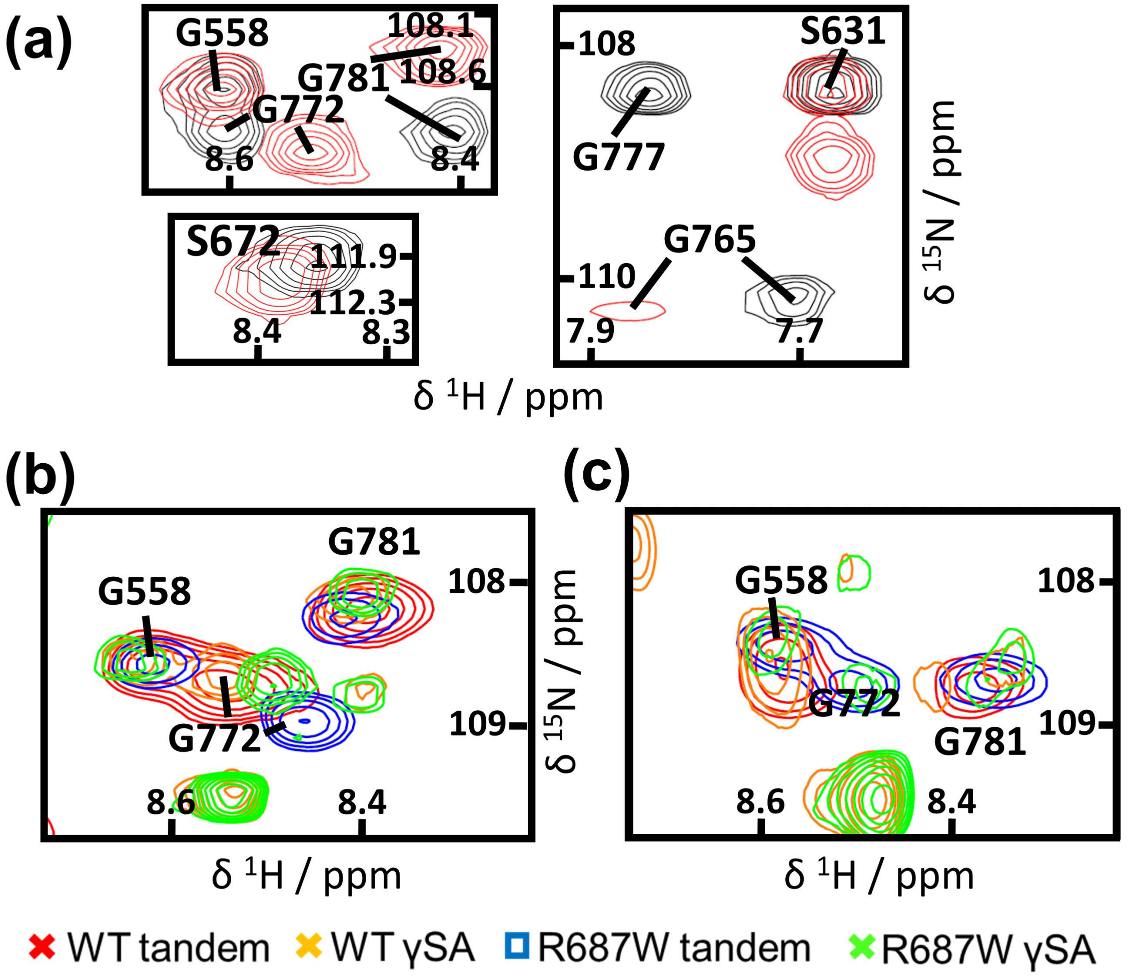

Figure S13. The NMR observations made in context of tandem-nSH2cSH2 constructs are reflected in ^{1}H,^{15}N-NMR spectra for the γ-specific array (γSA) (see also *Fig. 8*). (a) ^{1}H,^{15}N-SOFAST-HMQC spectra of NSH2CSH2 (red) and NSH2CSH2-pY^{783} (black). γSA spectra recorded at > 30°C are generally of higher quality, but for comparability with γSA-pY783, which is unstable at higher temperatures, both spectra were recorded at 25°C. Only cross peaks which were assigned for both γSA and γSA-pY783, or that are of particular relevance for this study, are highlighted here. A more complete backbone resonance assignment for nonphosphorylated γSA can be found elsewhere (10). (b-c) ^{1}H,^{15}N-SOFAST-HMQC spectra of residue Gly772/Gly781 in nonphosphorylated (b) and phosphorylated (c) tandem or γSA constructs. Symbols depict the cross peak centroids (color key depicted below). The 'closed' state is less populated in nonphosphorylated and Arg687Trp mutant constructs than in phosphorylated constructs and WT construct, respectively. Spectra were recorded at 34°C (panel a) or 25°C (panel b) and at 900 MHz (γSA-pY^{783}) or 700 MHz (all others).

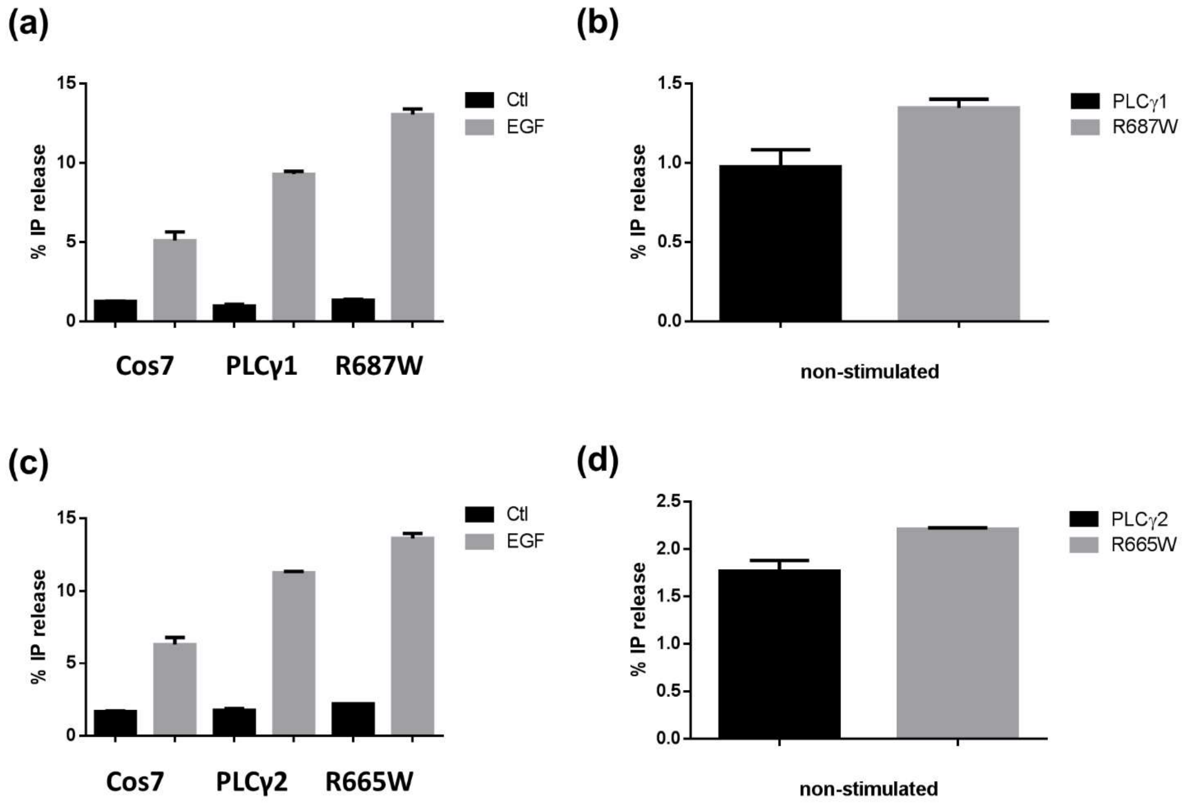

Figure S14: Activity assays. PLCγ containing Arg687Trp (PLCγ1) or Arg665Trp (PLCγ2) mutations expressed in COS-7 cells show increased activity with respect to wild type (WT) PLCγ, Panels (a)-(b) compare PLCγ1 activity. Panels (c)-(d) compare PLCγ2 activity. Panels (a) and (c): Basal activity (Ctl) and activity upon stimulation with EGF for COS-7 cells expressing different constructs are shown. The Arg687Trp mutant displays higher activity than WT upon activation by EGF; changes of basal activity are very moderate. Panels (b) and (d): only the small changes in the basal activity are shown.

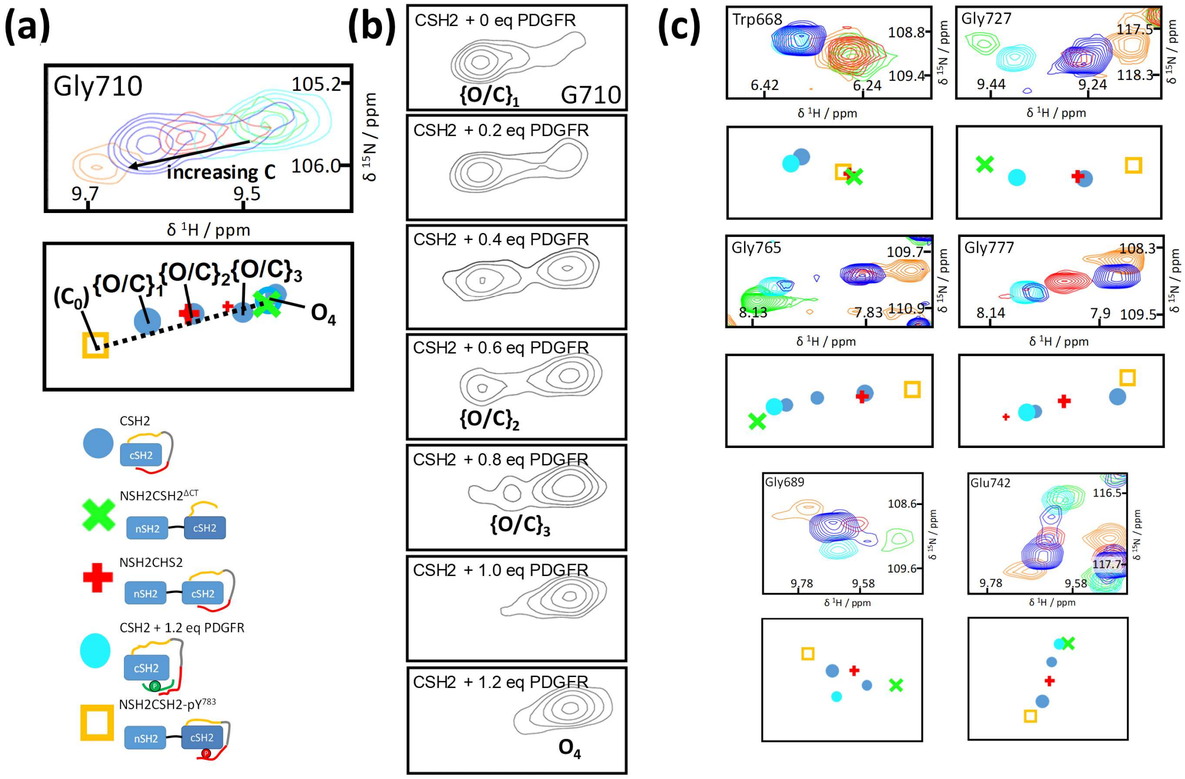

Figure S15: Titration of PDGFR phosphopeptide into CSH2. (a) ^{1}H,^{15}N-HSQC NMR spectra for the titration of PDGFR phosphopeptide into CSH2, focused on residue Gly710. CSH2 cross peaks observed for residue Gly710 coincide with the peak positions for some other constructs (the color scheme is similar to that employed in *Fig. 3*, extended to include the complex of CSH2 with the PDGFR peptide). The cross peak labeled 'C'$_0$ indicates the NSH2CSH2-pY^{783} 'closed' reference state that is not observed in CSH2. Four CSH2 states in slow exchange are identified: $\{O/C\}_1$, $\{O/C\}_2$, $\{O/C\}_3$ and O_4. (b) The titration with PDGFR peptide confirms kinetic connectivity between the states. (c) Additional examples supporting panels (a) and (b).
