# Supplementary material for: Dynamic Allostery in PLCγ1 and Its Modulation by a Cancer Mutation Revealed by MD Simulation and NMR
Source: Biophys J. 2018 Jul 3;115(1):31–45. doi: 10.1016/j.bpj.2018.05.031 (PMC6035297; doi:10.1016/j.bpj.2018.05.031)
Supplement: Document S2. Article plus Supporting Material [file mmc3.pdf]

Biophysical Journal
Article

Biophysical Society

Dynamic Allostery in PLCγ1 and Its Modulation by a Cancer Mutation Revealed by MD Simulation and NMR

Hans Koss,[1,2] Tom D. Bunney,[1] Diego Esposito,[2] Marta Martins,[1] Matilda Katan,[1] and Paul C. Driscoll[2,*]
[1]Institute of Structural and Molecular Biology, Division of Biosciences, University College London, London, United Kingdom and [2]The Francis Crick Institute, London, United Kingdom

ABSTRACT Phosphatidylinositol phospholipase Cγ (PLCγ) is an intracellular membrane-associated second-messenger signaling protein activated by tyrosine kinases such as fibroblast growth factor receptor 1. PLCγ contains the regulatory γ-specific array (γSA) comprising a tandem Src homology 2 (SH2) pair, an SH3 domain, and a split pleckstrin homology domain. Binding of an activated growth factor receptor to γSA leads to Tyr783 phosphorylation and consequent PLCγ activation. Several disease-relevant mutations in γSA have been identified; all lead to elevated phospholipase activity. In this work, we describe an allosteric mechanism that connects the Tyr783 phosphorylation site to the nSH2-cSH2 junction and involves dynamic interactions between the cSH2-SH3 linker and cSH2. Molecular dynamics simulations of the tandem SH2 protein suggest that Tyr783 phosphorylation is communicated to the nSH2-cSH2 junction by modulating cSH2 binding to sections of the cSH2-SH3 linker. NMR chemical shift perturbation analyses for designed tandem SH2 constructs reveal combined fast and slow dynamic processes that can be attributed to allosteric communication involving these regions of the protein, establishing an example in which complex *N*-site exchange can be directly inferred from ^{1}H,^{15}N-HSQC spectra. Furthermore, in tandem SH2 and γSA constructs, molecular dynamics and NMR results show that the Arg687Trp mutant in PLCγ1 (equivalent to the cancer mutation Arg665Trp in PLCγ2) perturbs the dynamic allosteric pathway. This combined experimental and computational study reveals a rare example of multistate kinetics involved in a dynamic allosteric process that is modulated in the context of a disease-relevant mutation. The allosteric influences and the weakened binding of the cSH2-SH3 linker to cSH2 should be taken into account in any more holistic investigation of PLCγ regulation.

INTRODUCTION

Phosphatidylinositol phospholipases (PLCs) are a family of enzymes that hydrolyze substrate membrane PI(4,5)P_2 phosphoinositides to yield the important second messengers diacylglycerol and inositol(1,4,5)P_3 (1,2). In mammals, the ubiquitously expressed PLCγ1 isoform is activated by growth factor receptor tyrosine kinases (RTKs) and the T cell receptor (3,4). The closely related PLCγ2 protein is stimulated by B cell and Fc receptors and mainly found in hematopoietic cells. The expression patterns of PLCγ1 and PLCγ2 are also reflected by the pathologies linked to various PLCγ1 and PLCγ2 mutations that activate PLCγ catalytic activity. PLCγ proteins differ from other PLC subfamily isoforms (PLC β, δ, ε) in that the sequence of the catalytic triose phosphate isomerase barrel domain is interrupted by the regulatory γ-specific array (γSA), consisting of a "split" pleckstrin homology (PH) domain, two Src homology 2 (SH2) domains (N-terminal SH2 (nSH2) and C-terminal SH2 (cSH2)), and an SH3 domain. The polypeptide domain arrangement N-spPH–nSH2–cSH2–SH3–C-spPH, in which the N- and C-terminal portions of the split PH domain come together to form a globular domain (5), results in a pseudo-cyclic structure for the γSA.

Activation of fibroblast growth factor receptor 1 (FGFR1) results in phosphorylation of Tyr766 in the unstructured C-terminal tail of the receptor and leads to recruitment of PLCγ isoforms (6). Full activation of PLCγ1 depends on receptor-dependent phosphorylation of γSA residue Tyr783 and on the presence of a functional cSH2 domain (7–9). We and others have shown that the cSH2 domain is bound to the catalytic domain to block substrate access to the active site in the resting state (10,11). Binding of FGFR1

Submitted August 21, 2017, and accepted for publication May 21, 2018.

*Correspondence: paul.driscoll@crick.ac.uk
Hans Koss's present address is Department of Biochemistry and Molecular Biophysics, Columbia University, New York, New York

Editor: Elizabeth Rhoades.
https://doi.org/10.1016/j.bpj.2018.05.031

© 2018 Biophysical Society.
This is an open access article under the CC BY license (http://creativecommons.org/licenses/by/4.0/).

to PLCγ1 occurs though the γSA nSH2 domain via both canonical and noncanonical protein-protein interactions (6), though more recently, it has been suggested that recruitment and activation of PLCγ1 by a panel of RTKs proceeds via the cSH2 domain alone (12).

Mutations in PLCγs have been detected in a variety of disease conditions through DNA sequence analysis, and these mutations can occur throughout the length of the protein (4). All pathogenic γSA mutations known to date are activating, and all are at sites conserved between PLCγ1 and PLCγ2 (4). For example, pathogenic γSA mutations are either located in split PH (Tyr495Cys, PLCγ2; Ser520Phe, PLCγ1; Leu845Phe, PLCγ2) or in cSH2 (Arg665Trp, PLCγ2; Arg707Gln, PLCγ1; Ser707Tyr, PLCγ2). Differential expression patterns of PLCγ1 and PLCγ2 have a great influence on the resulting pathology. For instance, Ser707Tyr (PLCγ2), which has been linked to autoimmune disease, was found to disrupt the autoinhibitory cSH2 interface with the enzyme core (10,13). Arg707Gln (PLCγ1) has been linked to secondary angiosarcoma and is suspected to disrupt the cSH2 domain structure and thereby release autoinhibition (14). The cSH2 mutation Arg665Trp (PLCγ2) occurs in the context of resistance to the Bruton's tyrosine kinase inhibitor ibrutinib used in chronic lymphocytic leukemia (15). No structural insight about the consequences of the Arg665Trp (PLCγ2)/Arg687Trp (PLCγ1) mutation is currently available.

In the PLCγ resting state, the extent to which the cSH2 domain is "available" for contact with the enzyme core and upstream agonists is unclear. In this context, the binding status of the cSH2 C-terminus, which is equivalent to the cSH2-SH3 linker and harbors the Tyr771, Tyr775, and Tyr783 phosphorylation sites, plays a particular role; recent studies proposed that Tyr783 phosphorylation by ITK kinase and Tyr771 phosphorylation by FGFR2 kinase would occur because of an enhancement of C-terminus availability (12,16). Although tandem SH2 crystal structures suggest that the cSH2 C-terminus might indeed be unbound in the nonphosphorylated state (10), a recent NMR study comparing constructs with and without an unmodified C-terminal extension suggested that the C-terminus is bound even in the nonphosphorylated state (16).

Here, we report the results of an investigation of the tandem nSH2-cSH2 segment of PLCγ1 and the impact of interactions of the globular cSH2 domain with the C-terminus in both non-phospho- and Tyr783-phospho-states, including within the context of the intact γSA. We present an analysis of the tandem SH2 domain protein by molecular dynamics (MD) simulation and heteronuclear NMR spectroscopy. Unexpectedly, the NMR results reveal a complex signature, the examination of which suggests partial dynamic interaction between the C-terminus and the cSH2 domain. In line with the results yielded from MD simulations, we interpret the NMR data to indicate dynamic allosteric communication between the Tyr783 phosphorylation site and the inter-SH2 domain junction, which operates via the C-terminus and residues preceding the C-terminus. Though examples of dynamic allostery have been presented before, here we introduce a case in which the combination of fast- and slow-exchange phenomena results in complex but interpretable NMR crosspeak patterns. We also characterize the effect of the PLCγ1 Arg687Trp mutation—homologous to the cancer therapy resistance Arg665Trp substitution in PLCγ2—on the characteristics of the tandem SH2 domain.

MATERIALS AND METHODS

Materials and methods used during the course of this study are provided in Supporting Materials and Methods, Section 1.

RESULTS

Our principal aim was to probe the impact of Tyr783 phosphorylation on the structural and dynamical properties of both the PLCγ1 nSH2 and cSH2 domains. The following will discuss in detail the structure and dynamics of different PLCγ1 tandem SH2 proteins (Fig. 1). According to the canonical description of SH2 domains, the ordered part of the PLCγ1 nSH2 domain comprises residues 545–662 and the cSH2 domain residues 663–756. The junction between the two SH2 domains ("nSH2-cSH2 junction") is here defined as the residue region 658–668. The region of the polypeptide that encompasses residues to the C-terminus of the cSH2 domain can be described as comprising three segments: the "pre-C-terminus" (residues 757–770), the "C-terminal linker" (residues 771–778), and the "Tyr783-peptide region" (residues 779–790). Together, the C-terminal linker and the Tyr783 peptide region constitute the "C-terminus"; the combination of all three segments is referred to here as the "extended C-terminus."

Three crystal structures (Protein Data Bank (PDB): 3GQI, 4FBN, 4EY0) are available for the tandem nSH2-cSH2 protein. The structures reported by Bunney et al. comprise a "long" unmodified construct (residues 545–790, which include the nSH2, cSH2 and C-terminal regions) and a similar construct that was phosphorylated on the Tyr783 side chain (PDB: 4FBN and 4EY0, respectively) (10). The three-dimensional (3D) structure of 4FBN is illustrated in Fig. 1 *a*. In the structures, electron density for residues 773–782 (4FBN) and 774–781 (4EY0), mainly corresponding to the C-terminal linker region, is missing, suggesting that this segment is dynamic in nature. In 4EY0, electron density corresponding to the phosphorylated Tyr783 peptide region is observed in the typical SH2 peptide-binding groove, indicating binding in *cis*. In the case of 4FBN, similar but much weaker density is observed, suggesting that at least partial binding can occur in the absence of Tyr783 phosphorylation. We sought to explore the effect of Tyr783 phosphorylation on the PLCγ1 tandem SH2

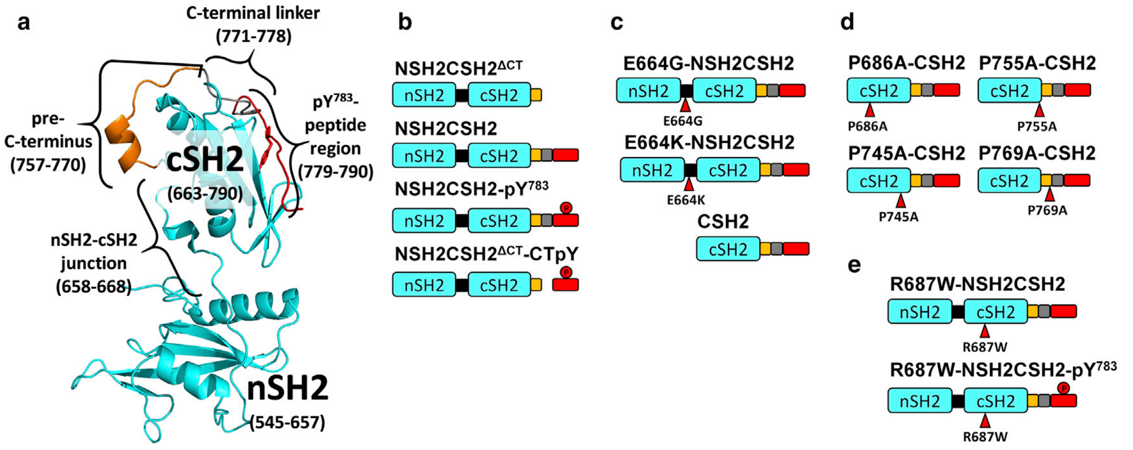

FIGURE 1 Tandem PLCγ1 nSH2-cSH2 constructs and nomenclature. (*a*) Crystal structure (PDB: 4FBN) of the tandem nSH2-cSH2 domains of PLCγ1 is shown. Residues 773–782 in the extended C-terminus, which are missing in the electron density, have been modeled for illustration purposes. (*b–e*) Schematic illustration shows key protein constructs examined in this study, grouped by function: (*b*) wild-type constructs to assess C-terminal binding, (*c*) derivative constructs to probe the role of the nSH2-cSH2 junction on fast-exchange dynamics and to assess slow exchange, (*d*) constructs with single Pro→Ala substitutions to probe slow exchange, and (*e*) disease-relevant Arg687Trp mutant constructs. The structure shown in (*a*) corresponds to the NSH2CSH2 construct shown in (*b*). The color-coding employed is as follows: cyan, SH2 domains; yellow, pre-C-terminus; gray, C-terminal linker; red, pY783-peptide region. In (*b*) and (*e*), the "P" symbol indicates phosphorylation of Tyr783. NSH2CSH2$^{\Delta CT}$-CTpY corresponds to the 1:1 complex between NSH2CSH2$^{\Delta CT}$ and a synthetic phosphopeptide (CTpY) corresponding to residues 779–790 in the NSH2CSH2 protein.

domain protein in solution using MD and heteronuclear NMR spectroscopy.

MD simulations to probe structural changes in the extended C-terminus and the nSH2-cSH2 junction upon Tyr783 phosphorylation

MD simulations were used to generate hypotheses about the structural consequences of Tyr783 phosphorylation and to guide subsequent experiments. We performed six atomistic 100 ns MD simulations of NSH2CSH2 and four atomistic 100 ns simulations of NSH2CSH2-pY^{783} using starting models derived from the crystal structures with PDB: 4FBN and 4EY0, respectively (corresponding to constructs for NMR, vide infra).

We evaluated convergence for local motions in the cSH2 domain by comparing contact and eigenvalue ranges for different MD trajectories using identical eigenvector (principal component) sets from a principal component analysis (PCA). The trajectories were not of sufficient duration to satisfactorily predict the full range of protein motion, interdomain flexibility, and orientation that might be present in solution; special approaches that enhance sampling of the conformational space would be required to accurately address questions regarding interdomain properties of the tandem SH2 constructs. Nevertheless, we identified differences within the cSH2 domain between NSH2CSH2 and NSH2CSH2-pY^{783} trajectories.

PCA in Cartesian space for backbone atoms of the cSH2 domain and the nSH2-cSH2 junction was performed to provide a quantitative analysis of motion represented in the trajectories (Fig. 2 *a*; Fig. S1). Eigenvectors were identified using merged trajectories for each set of simulations. The trajectories for the nonphosphorylated protein converged, as judged by overlap of the range of trajectory projections on the most significant eigenvectors. Analysis of the projections of each trajectory on the PCA eigenvectors revealed a difference between NSH2CSH2-pY^{783} and NSH2CSH2 trajectories in NSH2CSH2 eigenvector 2 (Fig. S2 *b*). Specifically, a high root mean-square fluctuation (RMSF) of the C-terminal linker and the pre-C-terminus in this eigenvector (Fig. 2 *a*) suggests that the dynamics of this region differs between NSH2CSH2 and NSH2CSH2-pY^{783}.

To further explore this finding, we performed contact analyses of the trajectories. We specifically focused on contacts between the extended C-terminus and the nSH2-cSH2 junction on one hand and other regions of the protein on the other hand (Fig. 2 *a*). For these protein regions, we use the secondary structure nomenclature that has been widely employed in previous descriptions of SH2 domains (17). Of particular note to this investigation, the α_A-helix (Arg675-Arg684) forms a rather exposed part of the cSH2 domain; the shorter α_B-helix (Asp634-Gln641) is close in space to the nSH2-cSH2 junction (Glu650-Trp668). The results of the contact analysis are shown in Fig. 2, *c* and *d* and Fig. S2 *a* for the extended C-terminus, and in Fig. S8 *a* for the αB-helix/nSH2-cSH2 junction. Here, we focus on the most significant differences in contact probability between the NSH2CSH2 and NSH2CSH2-pY^{783} trajectories related to the pre-C-terminus and the C-terminus. As expected, the contact between pTyr783 and the cSH2 domain was essentially permanent in the NSH2CSH2-pY^{783} trajectories but less stable in the NSH2CSH2 trajectories (Fig. S1 *b*). The contact between the following interface pairs was

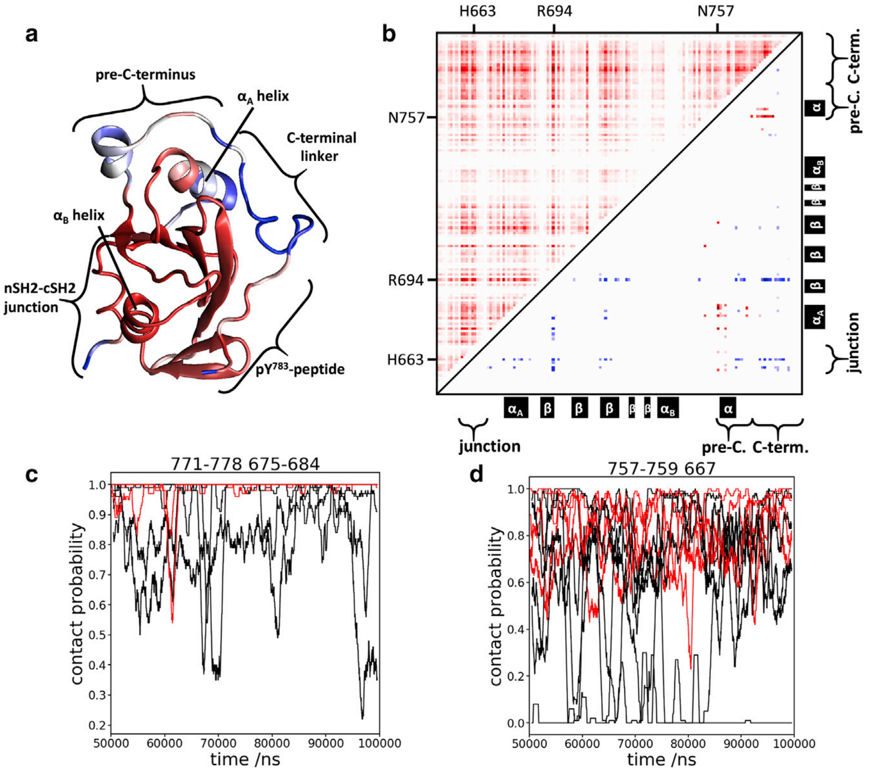

FIGURE 2 Molecular dynamics simulations predict dynamic differences between NSH2CSH2 and NSH2CSH2-pY^{783} for residues in the pre-C-terminus and the C-terminal linker. (*a*) Root mean-square fluctuation (RMSF) of the second eigenvector of the NSH2CSH2 PCA contains information about differences in backbone motions of the cSH2 domain between NSH2CSH2 and NSH2CSH2-pY^{783} (see also Fig. S1). The data suggest that the (pre-)-C-terminus could be relevant for an allosteric connection between Tyr783 and the nSH2-cSH2 junction. RMSF data were projected on a simulation model using color scaling: red (no fluctuation)–white–blue (high fluctuation). The particularly high RMSF for parts of the pre-C-terminus and the C-terminus is evident. (*b*) Mutual information between side-chain dihedral torsion angles for NSH2CSH2 and NSH2CSH2-pY^{783} trajectories is shown. The results for the cSH2 domain are shown in this panel (for the entire construct, see Fig. S2 *b*). Upper left half: residue-wise sum of mutual information between side-chain dihedral angles in NSH2CSH2 is shown; results were averaged but not filtered. Lower right half: mutual information difference between NSH2CSH2 and NSH2CSH2-pY^{783} is shown filtered (*t*-test). Red indicates an increase in mutual information in the phosphorylated protein (marked as described in the main text); blue indicates a decrease in mutual information. Particularly interesting residues as well as secondary structure elements are indicated on the plot abscissae. (*c* and *d*) Contact probabilities are shown between C-terminal linker and α_A-helix (*c*) or between pre-C-terminal residues 757–759 and nSH2-cSH2 junction residue 667, shown for different MD trajectories. Black, NSH2CSH2 ($n = 6$ trajectories); red, NSH2CSH2-pY^{783} ($n = 4$). Any number of contacts (<2.5 Å) at a given time point is counted as a single contact event, yielding a contact probability for a 1 ns sliding average time bin.

more frequent in the phosphorylated constructs: (p)Tyr783 and cSH2 domain core (670–750), C-terminal linker and cSH2 domain α_A-helix, pre-C-terminal region Asn757-Lys759 and cSH2 domain core, and pre-C-terminal region Asn757-Lys759 and nSH2-cSH2 junction residue Glu667 (Figs. 2 and S2 *a*). Thus, the MD simulations suggest that Tyr783 phosphorylation modulates the interactions between the pre-C-terminus and the nSH2-cSH2 junction and leads to stronger interactions of the extended C-terminus with the cSH2 domain.

We used the tool MutInf (18) to predict allosteric pathways from the MD trajectories by analyzing mutual information in the distribution of side-chain dihedral angles (Fig. 2 *b*). Particularly strong mutual information was found in the pre-C-terminus, the C terminus, and cSH2 core, which are likely to be in direct contact with these elements. Importantly, significant differences in mutual information were detected between NSH2CSH2 and NSH2CSH2-pY^{783}. The mutual information between pre-C-terminus residues Asn757 and Glu759 on one hand and residues in the 1) C-terminal linker and 2) cSH2 domain—specifically the α_A-helix—on the other hand is higher in the NSH2CSH2-pY^{783} simulations (Fig. 2 *b*). Conversely, the mutual information between various residues in the C-terminus, cSH2 residue Arg694, and junction residues Thr660 and His663 is lower for the NSH2CSH2 trajectories. There was also a lower level of mutual information between Arg694 and the nSH2-cSH2 junction on one hand and on the other hand several nSH2 residues (Fig. S2 *b*), only some of which are spatially close to the nSH2-cSH2 junction (Asn547-Arg567), whereas others are more distant (Asp615, Gly617, Glu589, Arg645-Glu649). Our data suggest an allosteric connection between the Tyr783 phosphorylation site and the nSH2-cSH2 junction (and to a minor extent, the nSH2 domain), which primarily involves sections of the extended C-terminus and the cSH2 domain residues with which they interact. Together, the MD PCA, contact, and MutInf analysis results suggest the potential presence of dynamic interplay between the extended C-terminus and the cSH2 domain.

Constructs for NMR spectroscopy

For NMR experiments, we prepared a variety of tandem-SH2 proteins; key constructs are illustrated schematically in Fig. 1 *b*. Similar to the proteins in the crystal structures, we prepared unmodified (non-phospho) "NSH2CSH2" (residues 545–790) and Tyr783-phosphorylated "NSH2CSH2-pY^{783}" (residues 545–790, pTyr783). We also prepared a construct comprising solely the cSH2 domain and the

appended C-terminal region ("CSH2"; residues 663–790), a shortened form of the tandem lacking the C-terminal residues 545–770 denoted "NSH2CSH2$^{\Delta CT}$," and a complex of NSH2CSH2$^{\Delta CT}$ with a pTyr783 phosphopeptide (residues 779–790) denoted "NSH2CSH2$^{\Delta CT}$-CTpY"; for the last sample, the CTpY peptide was added to 1.2 equivalents (CTpY binding to cSH2 has been described in a previous study (10)). From titration experiments, we confirmed that CTpY peptide binds sufficiently strongly ($K_D < 15\ \mu M$) to the cSH2 domain to be saturating under the NMR measurement conditions (Fig. S6; Supporting Materials and Methods, Section 2.7). In addition, we prepared single residue Gly and Lys mutants at junction residue Glu664 of NSH2CSH2 ("E664G-NSH2CSH2" and "E664K-NSH2CSH2"), both nonphospho- and phospho-preparations of a mutant Arg687Trp construct ("R687W-NSH2CSH2" and "R687W-NSH2CSH2-pY^{783}"), and single residue Ala mutants of CSH2 at Pro686, Pro745, Pro755, and Pro769.

Backbone crosspeak assignments and chemical shift perturbation analyses

^{1}H,^{15}N-heteronuclear single quantum coherence HSQC spectra of the tandem nSH2-cSH2 proteins constructs were generally well dispersed (Fig. S3). ^{1}H-, ^{15}N-, $^{13}C_{\alpha}$-, ^{13}CO, and $^{13}C_{\beta}$ crosspeak assignments were obtained for NSH2CSH2-pY^{783} and NSH2CSH2 using standard 3D NMR experiments. ^{1}H- and ^{15}N-backbone crosspeak assignments for all other constructs were transferred by inspection of ^{15}N-HSQC experiments. For NSH2CSH2$^{\Delta CT}$ and R687W-NSH2CSH2-pY^{783}, 3D HNCA data sets were recorded to support the assignments. The assignment is most complete for NSH2CSH2-pY^{783} (87%) and least complete for NSH2CSH2$^{\Delta CT}$ (53%). Some crosspeaks for the cSH2 domain, in the nSH2-cSH2 junction, and in the extended C-terminus could not be located, suggesting intermediate exchange broadening. Multiple crosspeaks were observed for several residues in mostly, but not exclusively, CSH2, E664G-NSH2CSH2, and E664K-NSH2CSH2, indicating the presence of slow exchange. Spectra of nonphospho constructs generally displayed weaker intensity than their phospho counterparts. Although pronounced line broadening in the cSH2 domain is often a result of the aforementioned exchange phenomena, the generally lower signal/noise observed for all residues (also in the nSH2 domain) is likely attributable to a propensity to weak self-association. We have characterized this behavior with ^{15}N nuclear relaxation and small angle x-ray scattering data obtained for NSH2CSH2 and NSH2CSH2-pY^{783} (Fig. S4; Supporting Materials and Methods, Section 2.1). Notably, the crosspeak positions and the extent of peak doubling were concentration independent. On the other hand, the presence of weak self-association confounded detailed linewidth analysis and relaxation dispersion experiments; hence, our NMR analyses are predominantly based on chemical shift patterns. In most of the following description, only the major (most intense) crosspeak is considered when peak doubling is present.

The C-terminus is partially bound in NSH2CSH2 constructs

Interactions between an SH2 domain and a cognate phosphopeptide ligand typically exhibit slow-exchange characteristics, which is consistent with relatively high affinity binding and a slow off-rate (19). On this basis, the spectrum of NSH2CSH2$^{\Delta CT}$ was expected to display different chemical shifts compared to either NSH2CSH2-pY^{783} or NSH2CSH2$^{\Delta CT}$-CTpY arising from the occupation of the cSH2 binding site by the C-terminal pTyr783 peptide in the latter cases. In line with this expectation, the NMR spectra of NSH2CSH2$^{\Delta CT}$ and NSH2CSH2-pY^{783} appear quite different (Fig. S5). For the purposes of describing our observations, it is useful to declare the following: trivially, for NSH2CSH2$^{\Delta CT}$, no interaction of the pTyr783 region with the cSH2 domain is present, and the protein is in an unbound or "open" state. On the other hand, the characteristics of the NSH2CSH2-pY^{783} protein, with at least pTyr783 of the C-terminal tail occupying the cSH2 binding site, can be regarded as representing a bound or "closed" state. Importantly, the largest chemical shift perturbations (CSPs) between the "open" and "closed" forms were not confined to the putative binding contact zones in the cSH2 domain and pTyr783 region but were also observed for sites in the pre-C-terminus (distant from the pY783 binding site), the C-terminal linker, and throughout the cSH2 domain.

Superposition of the NMR spectra for all of the other constructs that we have examined shows that the chemical shifts for conserved residues do not always coincide with those of either NSH2CSH2$^{\Delta CT}$ ("open" state) or NSH2CSH2-pY^{783} ("closed" state). The crosspeaks for a given residue lie on or close to the vector that joins the corresponding crosspeaks for the "open" and "closed" states. Importantly, this behavior was detected over a whole series of different constructs, several of which were not prepared with this specific phenomenon in mind but for other purposes, and so, this was an adventitious observation. For example, residues Gly777 and Gly789 from the C-terminus and residues Gly689, Ala690, and Gly710, which are located on the cSH2 protein surface, exhibit this behavior (Fig. 3 *a*). This pattern suggests an underlying fast-exchange phenomenon; importantly, the population of "closed" and "open" states for any given construct and residue can be directly inferred by comparing the corresponding peak position to those for the reference "open" and "closed" states, similar to the procedure employed in chemical shift projection analysis (CHESPA) (20). Given their respective locations in the 3D structure, these two sets of residues could take part in mutual interactions that would accompany entry of the

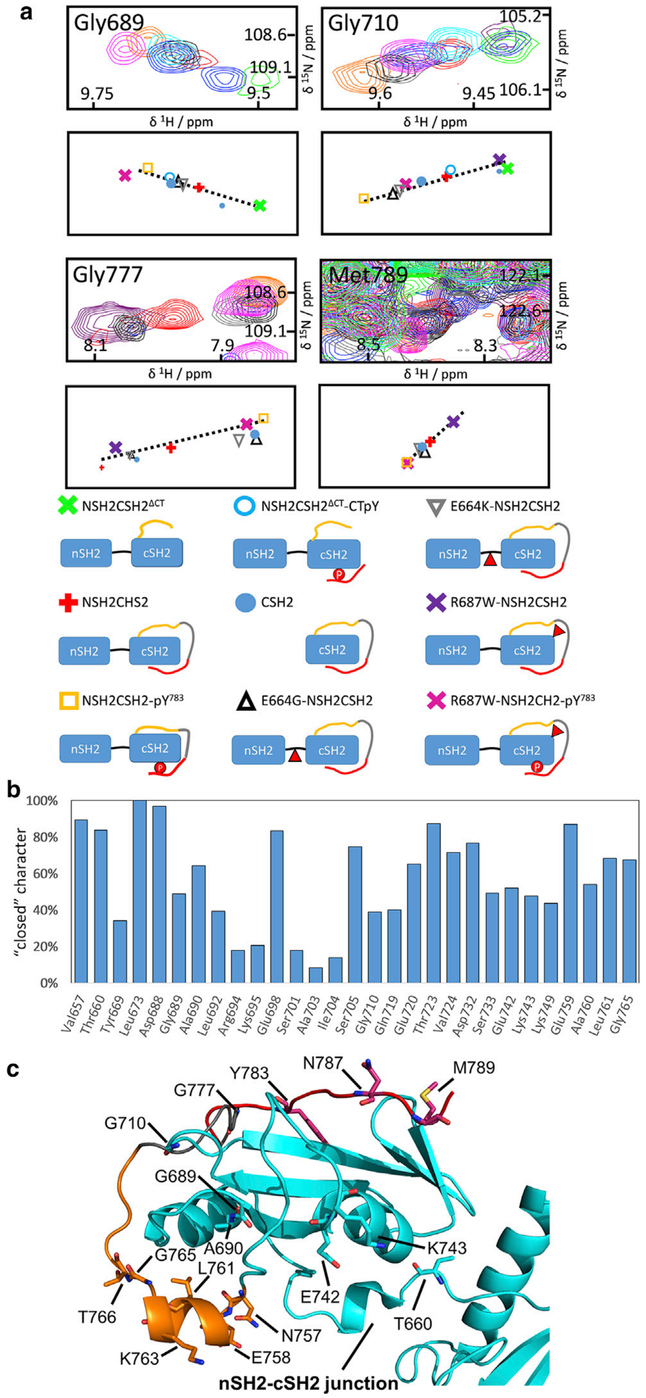

FIGURE 3 NMR spectra demonstrate that the C-terminus is partially bound in NSH2CSH2 constructs. (*a*) Superposition of the ^{1}H,^{15}N-HSQC NMR spectra of the tandem nSH2-cSH2 constructs is shown. Each pair of panels is focused on a particular residue and shows the raw NMR spectra (*upper panel*) with schematic illustrations of the crosspeak centroids (*lower panel*). The contour colors and centroid symbols are plotted according to the protein construct, as indicated in the key below, which uses cartoon representations wherein the NSH2-CSH2 junction, pre-C-terminus, C-terminal linker, and Tyr783 region are shown in black, yellow, gray, and red, respectively; phosphorylation of Tyr783 is indicated by a red circle with a white "P," and sites of single residue substitutions are indicated by a red arrowhead. In each case, the crosspeaks are located on a vector connecting positions corresponding to fast-exchanging "open" (O) and "closed" (C) states (see main text). (*b*) Plot of the degree of "closed" character in the NSH2CSH2 construct is shown, estimated from the position of the corresponding crosspeaks on the O-C vector. (*c*) Model of the cSH2 protein (based on 4FBN) shows the location of residues whose crosspeak chemical shifts demonstrate O ⇌ C fast-exchange.

extreme C-terminal pTyr783 region into the cSH2 binding site. The MD simulations suggest high mobility and multiple transient contacts between the C-terminus and the cSH2 domain. Therefore, the fast-exchange phenomenon suggested by the pattern of chemical shifts can be attributed to a dynamic interaction between the C-terminus and the cSH2 domain; the local conformation of the NSH2CSH2 protein shuttles between at least two states, rationalized by partial binding of the C-terminal region to the cSH2 domain despite the absence of Tyr783 phosphorylation. This interaction is consistent with the occupation by the nonphosphorylated Tyr783 region of the cSH2 binding site in the 4FBN crystal structure of the NSH2CSH2 protein. The fast-exchange NMR characteristic and the weak electron density in the crystal structure suggest that this interaction is substantially weaker than in the case of NSH2CSH2-pY^{783} but, compared to an untethered nonphospho-Tyr783 peptide, might be promoted by the enhanced effective concentration provided by the tethering pre-C-terminus and C-terminal linker.

Multiple NSH2CSH2 crosspeak patterns evidencing fast exchange were identified; the corresponding locations are projected onto a 3D structural representation in Fig. 3 *c*, whereas peak patterns are displayed in Figs. 3 *a*, S7, S11, and S12. Residue-wise examination of the crosspeak positions reveals that the "open" and "closed" state population distribution differs from residue to residue (Fig. 3 *b*), indicating that the dynamic situation in NSH2CSH2 cannot be properly described by a single two-state fast-exchange model applicable to the whole protein construct. Rather, the pattern suggests a heterogeneous dynamic interaction between the cSH2 domain and different parts of the C-terminal tail. For this reason, it is not possible to determine a single K_D or k_{ex} parameter to describe globally the equilibrium between "open" and "closed" forms.

C-terminal linker-dependent dynamic allosteric communication of Tyr783 phosphorylation to the nSH2-cSH2 junction

Similar to the situation with NSH2CSH2, a subset of cSH2 residues of NSH2CSH2$^{\Delta CT}$-CTpY display evidence of heterogeneous "open" ⇌ "closed" dynamics, corresponding to a partially bound pre-C-terminus (Fig. 3 *a*; Fig. S7). However, the crosspeaks for these residues are directly coincident only for the NMR spectra of NSH2CSH2$^{\Delta CT}$ and NSH2CSH2$^{\Delta CT}$-CTpY. This pattern indicates that upon Tyr783 phosphorylation, CSP for the pre-C-terminus

(Glu758, Ala760, Leu761, Gly765, Thr766) toward the "closed" state is observed only when the C-terminal linker (residues 771–778) is present (Fig. 4 *a*); the interaction between the cSH2 domain and the pre-C-terminus is dependent upon an intact tether between the pre-C-terminus and the C-terminal tail.

The peak position for nSH2-cSH2 junction residue Thr660 is perturbed by phosphorylation of Tyr783 both for NSH2CSH2$^{\Delta CT}$-CTpY and to a greater degree for NSH2CSH2-pY^{783} (with respect to NSH2CSH2$^{\Delta CT}$; Fig. 4 *a*). As the separation between Thr660 and the C-terminus is substantial (the distance between Thr660 and Tyr783 Cα atoms is ~23.9 Å), the shift pattern suggests that Tyr783 phosphorylation is communicated to Thr660 in the nSH2-cSH2 junction, at least in part, via the tethering C-terminal linker. In line with this observation, substantial chemical shift differences between NSH2CSH2-pY^{783} and NSH2CSH2$^{\Delta CT}$-CTpY were observed throughout the cSH2 domain and the pre-C-terminus (Fig. S7 *c*; Fig. 5 *b*). One can rationalize these chemical shift patterns on the basis of a dynamic allosteric connection between the Tyr783 binding site and the nSH2-cSH2 junction.

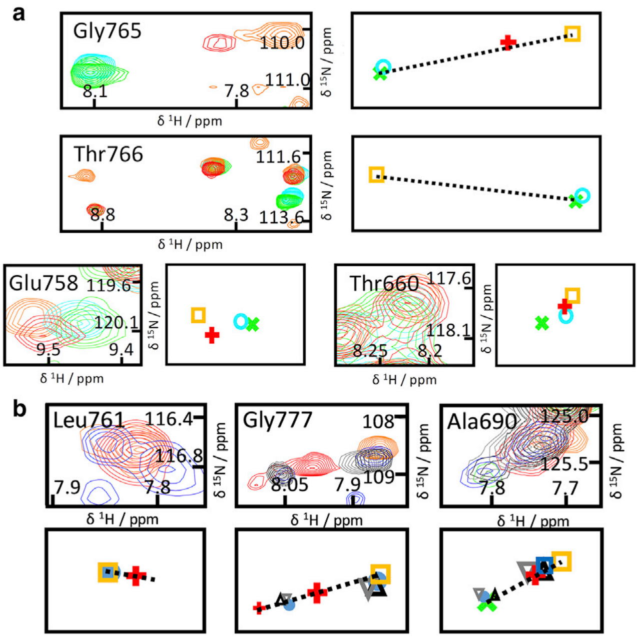

FIGURE 4 NMR evidence for a C-terminal-linker-dependent allosteric pathway connecting the Tyr783 phosphorylation site with the nSH2-cSH2 junction. (*a* and *b*) Superposition of ^{1}H,^{15}N-HSQC spectra is shown next to supporting schematic representations of crosspeak centroids ((*a*) 600 MHz, (*b*) 700 MHz, using the same color scheme as in Fig. 3; further examples are shown in Fig. S7). (*a*) For many extended C-terminus residues, only the crosspeaks for NSH2CSH2$^{\Delta CT}$ and NSH2CSH2$^{\Delta CT}$-CTpY coincide, indicating a C-terminal-linker-dependent effect. Thr660 (nSH2-cSH2 junction) is affected by Tyr783 phosphorylation in part via the bridging C-terminal linker. (*b*) The crosspeaks for the residues shown are shifted toward the "closed" state when the nSH2 domain is absent (CSH2) or when the nSH2-cSH2 junction is mutated, consistent with allosteric communication via the extended C-terminus.

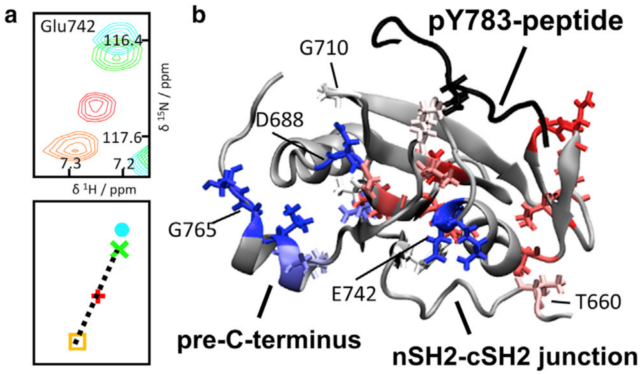

FIGURE 5 C-terminal-linker-dependent and C-terminal-linker-independent contributions to allosteric communication. (*a*) Superposition of ^{1}H,^{15}N-HSQC NMR spectra and corresponding schematic representation of the crosspeak centroids for residue Glu742 are shown. The color scheme is as depicted in Fig. 3. The crosspeaks for NSH2CSH2-pY^{783} and NSH2CSH2$^{\Delta CT}$-CTpY are shifted in opposite directions along the "fast-exchange vector," exposing a differential impact of allosteric influences. (*b*) Depiction of the cSH2 domain illustrates how the Tyr783 status is communicated via two distinct allosteric pathways (the C-terminal linker is not shown; pY783-peptide is in *black*). The communication type is graded by color: C-terminal-linker-independent allosteric pathway in red—via white—to C-terminal-linker-dependent communication in blue. Only residues with data are displayed in stick model form. The communication type was determined by the projection of the NSH2CSH2$^{\Delta CT}$-CTpY peak on the vector connecting the NSH2CSH2-pY^{783} and NSH2CSH2$^{\Delta CT}$ crosspeaks.

To confirm the dynamic allosteric connection between the nSH2-cSH2 junction and the Tyr783-binding site, we assessed mutants that modulate the fast-exchange equilibria state populations for multiple residues in a manner consistent with long-range dynamic allostery. The nSH2-cSH2 junction has the potential to provide a tight linkage between the two SH2 domains. The NMR spectra of the isolated cSH2 domain (CSH2, a construct that is equivalent to deletion of the nSH2 domain from the NSH2CSH2 protein) and nSH2-cSH2-junction mutants E664G-NSH2CSH2 and E664K-NSH2CSH2 provide an opportunity to trace which cSH2 residues are influenced by the presence of the nSH2 domain and/or an intact nSH2-cSH2 junction. Relative to the wild-type tandem domain constructs, these proteins can be characterized as "junction-disrupted." Moreover, along with the wild-type (WT) proteins, the spectra of these mutants are helpful in dissecting the influence of the C-terminus on the nSH2-cSH2 junction. The crosspeaks of residues Leu761, Lys763, Gly765, Glu776, Gly777, and Asn787 in the extended C-terminus for CSH2 as well as for E664G-NSH2CSH2 and E664K-NSH2CSH2 are often not coincident with those for NSH2CSH2 but instead are located on the vectors connecting those for NSH2CSH2 and NSH2CSH2-pY^{783} (Fig. 4 *b*; Fig. S7 *a*), suggesting that the pre-C-terminus and the C-terminus populate the "closed" form more than in NSH2CSH2. Mutations within the nSH2-cSH2 junction, which in effect communicates the presence of the nSH2 domain, or removal of the

nSH2 domain results in a stronger association of the pre-C-terminus and C-terminal linker with the cSH2 domain. As well as these C-terminal residues, Ala690 in the globular part of the cSH2 domain and in spatial proximity of the C-terminus displays a crosspeak in NSH2CSH2 at a position corresponding to dynamic fast exchange between "open" and "closed" states (Fig. 4 *b*), whereas the equivalent crosspeak for the CSH2 construct is shifted toward position in the "closed" NSH2CSH2-pY^{783}. This difference suggests that the attached nSH2 domain influences not only the extended C-terminus but also the cSH2 residues with which the C-terminus interacts. MD simulations and NMR reveal an unstable contact between Asn757-Glu759 and the nSH2-cSH2 junction in NSH2CSH2-pY^{783}, which is further loosened in NSH2CSH2 by virtue of contact between the nSH2-cSH2 junction and the pre-C-terminus (details in Supporting Materials and Methods, Section 2.2).

Overall, the picture is that the extended C-terminus and the nSH2 domain represent countervailing influences on the chemical shifts of cSH2 crosspeaks, consistent with two-way dynamic allosteric communication between the pY783-binding site and the nSH2-cSH2 junction. In energetic terms, the effects must be weak because the "open"-"closed" population balances for different residues vary only within a small range, but this is sufficient to lead to distinct influences on the chemical shifts that are directionally coherent (i.e., consistently toward either the "open" or "closed" states).

Relevance of the dynamic C-terminal-linker-dependent pathway for allosteric communication

The foregoing experimental observations support the concept that binding of the Tyr783 region to the cSH2 domain influences the nSH2-cSH2 junction in a C-terminal-linker-dependent manner, in line with expectations from the MD simulations described above. Therefore, crosspeak positions that differ for particular residues in NSH2CSH2$^{\Delta CT}$ and NSH2CSH2$^{\Delta CT}$-CTpY must indicate a C-terminal-linker-"independent" impact of Tyr783 phosphorylation. As outlined in Supporting Materials and Methods, Section 2.6, and Fig. S7, we identified residues that are remote from the pY783 peptide binding site but influenced by pY783 binding even in the absence of the C-terminal linker, constituting a C-terminal-linker-"independent" pathway. Mapping the impact on both C-terminal-linker-dependent and C-terminal-linker-"independent" pathways on the cSH2 structure (Fig. 5 *b*) suggests that the former pathway is mostly dominant, other than in the pY783 peptide binding site.

Structural and dynamic interactions between the cSH2 α_B-helix and the nSH2-cSH2 junction that were predicted in the MD simulations are supported by the NMR data, as discussed in Supporting Materials and Methods, Section 2.2, and Fig. S8. In this context, Glu742 emerges as a particularly notable residue. In the crystal structure, this residue is located next to the cSH2 domain α_B-helix, adjacent to the nSH2-cSH2 junction (Fig. S8 *c*). Glu742 shows a fast-exchange type peak pattern that indicates that it is part of the dynamic C-terminal-linker-dependent allosteric pathway. In addition, we found evidence that pY783 peptide binding to NSHCSH2$^{\Delta CT}$ also modulates the dynamic equilibrium at this residue. Specifically, C-terminal-linker-dependent and -independent Tyr783 binding have opposing effects on the Glu742 chemical shift (Fig. 5 *a*). A more detailed chemical shift and MD analysis results suggest that the nearby nSH2-cSH2 junction structure is modulated in a different manner in each case (more information can be found in Supporting Materials and Methods, Section 2.5). The relevance of the dynamic C-terminal linker-dependent allosteric pathway in the framework of overall allostery is corroborated by the MD simulations, the dominant influence on sites that are spatially distant from Tyr783, and the specific impact of this pathway on the dynamics of the cSH2 domain and the C-terminus. Importantly, the disease-relevant Arg687Trp substitution has a specific impact on the C-terminal-linker-dependent allosteric pathway, as discussed below.

Arg687Trp substitution modulates the dynamic allosteric pathway

The single site activating mutation Arg665Trp in PLCγ2 is known to occur in the context of ibrutinib resistance in chronic lymphocytic leukemia (4,5,15). Although this was a phenomenon was associated with influence on Rac-induced activation of the enzyme (21), we have found that when the Arg665Trp mutant of PLCγ2, and separately the equivalent Arg687Trp mutant of PLCγ1, is expressed in COS-7 cells, stimulation with epidermal growth factor (EGF) leads to significant loss of autoinhibition of phospholipase activity (Fig. S14). Although the basal level of activity (i.e., without EGF) is only moderately increased compared to WT, both mutants exhibited a higher level of activity under EGF receptor stimulation, suggesting that the mutants are more readily activated than the WT equivalents. For experimental studies, we have found it difficult to obtain ^{15}N-labeled PLCγ2 constructs for NMR studies. However, the PLCγ1 Arg687Trp mutant is tractable.

As a prelude to NMR investigations of the mutant, we performed three MD simulations each 100 ns of R687W-NSHCSH2 and R687W-NSH2CSH2-pY^{783} for comparison with the respective WT trajectories. In the 3D structure, Arg687 is located on the loop connecting cSH2 helix α_A and the first strand of the central β-sheet in close proximity to the pre-C-terminus. The simulations suggest that the Arg687Trp substitution reduces the allosteric communication described above for the WT (Figs. S1, S9, and S10;

Supporting Materials and Methods, Section 2.8). This disruption appears to be linked to a change of the structure or dynamics of the C-terminus.

We prepared Arg687Trp tandem SH2 constructs to experimentally probe the impact on structure, dynamics, and the dynamic allostery. The pre-C-terminus Gly765 crosspeak for the Arg687Trp mutant construct is located well away from the fast-exchange "open"-"closed" vector, indicating CSPs dominated by the local effect of the side-chain substitution over other influences. The overall pattern suggests that Arg687 could be in direct contact with residues in the C-terminus. However, CSPs between NSH2CSH2-pY^{783} and R687W-NSH2CSH2-pY^{783} constructs are not confined to the immediate locale of residue Arg687 (Fig. 6 *a*). The Arg687Trp change likely impacts the nSH2-cSH2 junction itself, as suggested by the large CSPs for Thr660 (Fig. 6 *b*). Significant CSPs were observed for several residues in the putative contact regions between the extended C-terminus and the cSH2 domain, for example Ala690, Gly710, Gly758, Ala770, Leu761, Gly772, Ala773, and Thr766, as well as for the α_B-helix residue Glu742 (Fig. 6 *b*; Fig. S11 *c*). Data for R687W-NSH2CSH2 indicate a similar trend (Fig. S11 *b*). The data reveal that the pre-C-terminus and C-terminal linker populate the "closed" state to a lesser degree in Arg687Trp constructs than in the WT, suggesting that the Arg687Trp substitution modulates the C-terminal-linker-dependent allosteric pathway.

It is noteworthy that there are no major CSPs for residues belonging exclusively or even mostly to the C-terminal-linker-independent pathway upon nSH2 domain removal or upon Arg687Trp mutation; namely, Leu692, Lys695, Ala703, Ile704, Glu720, Val724, Gly727, Asn728, and Ser733 have the same or very similar chemical shifts in the mutant (Fig. S11 *d*). The Arg687Trp mutation selectively perturbs the dynamic C-terminal-linker-dependent pathway and exerts its functional influence either by weakening the association of the C-terminus with the cSH2 domain or by impact upon the nSH2-cSH2 junction.

Structural origin and dynamic complexity of the C-terminal-linker-dependent pathway

Returning to the WT proteins, in addition to the fast-exchange phenomena described above, peak doubling or broadening was also observed in the spectra. Residues for which peak doubling, and even tripling or more, was evident are located in the extended C-terminus or in regions of the cSH2 domain predicted to be in close proximity, for example Gly689, Ala690, Gly710, Gly727, Gly765, Thr766, and Gly777 (Figs. 3 *a*, 4 *b*, and 7; Figs. S12 and S15). Notably, the peak doubling was most readily discerned in the spectrum of CSH2, presumably because of the overall lower number of residues (∼crosspeaks) and narrower linewidths on account of the smaller molecular mass. However, additional minor populations in NSH2CSH2 were revealed upon mutation of nSH2-cSH2 junction residue Glu664. In multiple instances, the resulting peak patterns resemble those found for CSH2 (Figs. 7 *b* and S7 *a*), consistent with the notion that the junction plays a role in relaying the presence of the nSH2 domain to the tandem construct C-terminus.

The peak doubling pattern was observed consistently over several independently prepared samples and therefore cannot be attributed to chemical heterogeneity and must arise because of slow exchange between different states. As a result, the relative intensity of the crosspeaks is defined by the respective state populations. In the cases in which (only) two CSH2 crosspeaks are observed, one peak is frequently located at the position of, or very close to, the corresponding peak for NSH2CSH2$^{\Delta CT}$, whereas the other sits on the vector connecting the crosspeaks for NSH2CSH2$^{\Delta CT}$ and

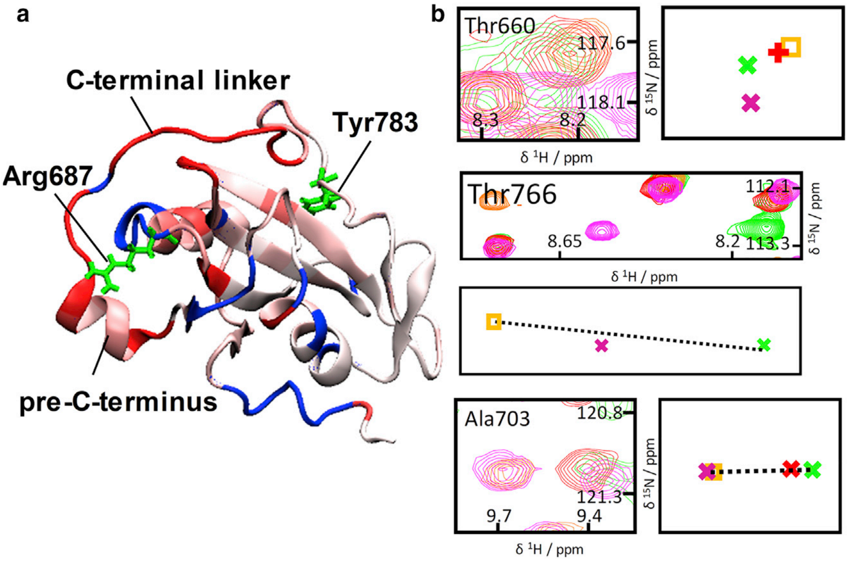

FIGURE 6 NMR data indicating a specific effect of the Arg687Trp mutation on the C-terminal-linker-dependent pathway. (*a*) Depiction of the cSH2 domain is colored to illustrate the degree of chemical shift perturbation for R687W-NSH2CSH2-pY^{783} relative to NSH2CSH2-pY^{783} mutants: white (no perturbation) to red (strong perturbation); blue, insufficient data. (*b*) Superposition of ^{1}H,^{15}N-HSQC spectra recorded at 600 MHz and corresponding schematic representations of crosspeak centroids are shown (*color scheme* as in Fig. 3). The Arg687Trp mutation leads to a perturbation for junction residue Thr660. The crosspeaks for residues such as Ala703 that are associated with the C-terminal-linker-independent allosteric pathway are barely shifted. Larger CSPs are observed for residues exemplified by Thr766 that are associated with the C-terminal-linker-dependent pathway. Additional examples are shown in Fig. S11.

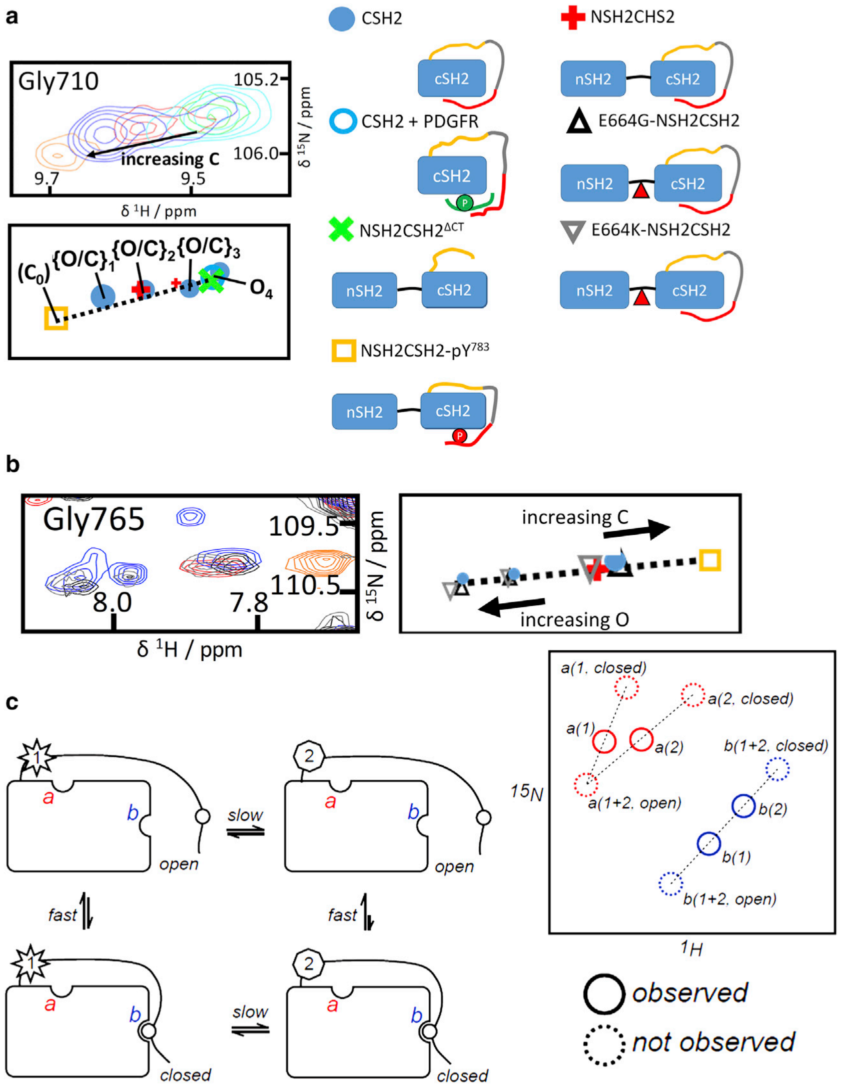

FIGURE 7 For a Figure360 author presentation of Fig. 7 *c*, see the figure legend at https://doi.org/10.1016/j.bpj.2018.05.031#mmc2. Figure360▷

Evidence for multistate fast and slow exchange in CSH2 and NSH2CSH2 constructs. (*a*) CSH2 crosspeaks observed for residue Gly710 coincide with the peak positions of some other constructs (the *color* scheme is similar to that employed in Fig. 3, extended to include the complex of CSH2 with the platelet-derived growth factor receptor (PDGFR) phosphopeptide). The crosspeak label C_0 indicates the NSH2CSH2-pY^{783} bound reference state that is not observed in CSH2. Four CSH2 states in slow exchange are identified: $\{O/C\}_1$, $\{O/C\}_2$, $\{O/C\}_3$, and O (see main text). These states can also be observed over the course of a titration of CSH2 with PDGFR phosphopeptide (titration and further examples are shown in Fig. S15). The titration endpoint (CSH2 fully bound to the PDGFR phosphopeptide) is shown here. (*b*) Superposition of ^{1}H,^{15}N-HSQC spectra (700 MHz) indicates slow exchange for CSH2 and junction mutant constructs, exemplified by residue Gly765; color scheme as in (*a*). (*c*) Consideration of the effects of fast exchange in combination with "local" versus "remote" slow exchange on crosspeak patterns; see Figure360 for an extended animated version of this scheme. Top: in the scheme, the origin of slow exchange between states "1" and "2" (*left* and *right*, respectively) is illustrated conceptually by star and heptagon symbols. Consider now additional fast exchange (between *top* and *bottom* structures), for example corresponding to the association of an otherwise flexible appendage with the protein core. The rate of fast exchange depends on whether the molecule is in state "1" or "2." We can now probe two sites *a* and *b* at which the combined exchange phenomena might be observed; the corresponding schematic spectra for both sites are shown on the right of the bottom panel. For site *a*, the origin of the slow exchange is "local"; for site *b*, it is "remote." Note that in this study, the position of the fully "closed" position is known from experiments with NSH2CSH2-pY^{783}. In the "local" slow case *a*, we would not expect crosspeak(s) to sit on a single vector between "open" and "closed" state coordinates. The observations described in this article correspond to the scenario depicted for site *b*, i.e., the crosspeak patterns that we have analyzed arise from residues apparently "remote" from the source of slow exchange.

NSH2CSH2-pY^{783}. The observed crosspeak pattern is consistent with the concept that, in addition to the major population already discussed, the second population of molecules is also undergoing the "open" ⇌ "closed" (fast) exchange discussed above for NSH2CSH2. However, these additional slowly exchanging states of the C-terminus and pre-C-terminus differentially influence the apparent (local) affinity of a given residue for the cSH2 domain. For the "simpler" case of CSH2 peak-doubling, we can envisage the following: in one slow-exchanging state (state "1"), the effective "open" → "closed" on-rate is $k_{on,1}$, and the "closed" → "open" off-rate is $k_{off,1}$; in the second slow-exchanging state "2," at least one of the effective on-rates and/or off-rates $k_{on,2}$, $k_{off,2}$ is different. Whereas the rate for the "1" ⇌ "2" interconversion is slow, in each of states "1" and "2" (with populations p_1 and p_2), the exchange between "open" and "closed" is fast on the NMR timescale. For residue *i*, we will thus observe two effective binding constants, namely $K^i_1 = p^i_{1,closed}/p^i_{1,open} = k^i_{1,on}/k^i_{1,off}$ and $K^i_2 = p^i_{2,closed}/p^i_{2,open} = k^i_{2,on}/k^i_{2,off}$. In the absence of multiple slow-exchanging states, a single crosspeak would sit at a position along the vector joining the chemical shift coordinates for the "open" and "closed" forms. When the "1" ⇌ "2" exchange is slow, two crosspeaks would be expected, with intensities defined by the state populations p^i_1 and p^i_2. However, when both of the states X="1," "2" are each also in "open"$_X$ ⇌ "closed"$_X$ fast exchange, the crosspeaks are again positioned along the "open"-"closed" vector. The whole scenario can be encapsulated in the schema:

$$\left\{O \overset{*}{\rightleftharpoons} C\right\}_1 \overset{\dagger}{\rightleftharpoons} \left\{O \overset{*}{\rightleftharpoons} C\right\}_2,$$

where O and C denote "open" and "closed," brackets encapsulate the states "1" and "2," and * and † indicate fast and slow exchange, respectively.

Thus, there are two populations of molecules, each demonstrating "open" ⇌ "closed" equilibria but spectroscopically separated by a "remote" slow-exchange phenomenon. "Remote" here is meant in the sense that the difference in molecular properties that gives rise to slow exchange does not influence the chemical shifts underlying the concomitant fast-exchange process but can influence the populations for the fast exchange in each of the two (slowly exchanging) states "1" and "2." The general scenario can be conveniently depicted in cartoon form (Fig. 7 *c*, animated Figure360). Importantly, the pattern of crosspeaks is distinctly different from that expected for a crosspeak that originates from a location close to the site of slow exchange.

Our model for the dynamic origin of the detected crosspeak patterns is bolstered by our observations of crosspeak doubling in a titration of a platelet-derived growth factor receptor *β* (PDGFR*β*)-derived phosphopeptide with CSH2 (Fig. 7 *a*). As outlined in Supporting Materials and Methods, Section 2.9, and Fig. S15, we observe more than two slowly exchanging states over the course of the titration, which can be understood by invoking an extension of the combined fast/slow-exchange scheme:

$$\left\{O \overset{*}{\rightleftharpoons} C\right\}_1 \overset{\dagger}{\rightleftharpoons} \left\{O \overset{*}{\rightleftharpoons} C\right\}_2 \overset{\dagger}{\rightleftharpoons} \left\{O \overset{*}{\rightleftharpoons} C\right\}_3 \overset{\dagger}{\rightleftharpoons} O{:}P,$$

where O:P indicates the protein:peptide complex. Importantly, we observe that over the course of the titration, the populations of the slow-exchanging "1," "2," and "3" states change because of the mass action of the peptide, which effectively captures the "open" state of the protein to form the O:P protein-peptide complex. When the peptide is in molar excess, essentially all of the protein is in the O:P state (see Supporting Materials and Methods, Section 2.9). Our interpretation of peak patterns as the result of a combination of slow and fast-exchange phenomena also rationalizes the peak tripling observed for residue Thr766 in the CSH2 construct (Fig. S12 *b*; Supporting Materials and Methods, Section 2.3).

Slow exchange due to purely conformational events is relatively rare in protein NMR. Given that the most common source of such slow events is the *cis* ⇌ *trans* isomerism of X-Pro peptide bonds (22,23), often in disordered regions of polypeptides, we compared CSPs for WT constructs with variants in which specific proline residues, including two that reside in the C-terminal linker, were substituted with alanine (Fig. S12 *a*; Supporting Materials and Methods, Section 2.4). Although none of the mutations completely quenched the presence of slow exchange, overall our observations strongly suggest that *cis-*/*trans*-peptide bond isomerization in the extended C-terminus leads to the complexity of the spectra of the cSH2-domain-containing proteins.

Tyr783 phosphorylation and the Arg687Trp mutation in the context of the γ-specific array

To test whether the observations made for tandem SH2 proteins also apply in the context of the larger γSA protein, both nonphospho- and phospho-constructs for WT and mutant versions of the γSA were prepared. Many backbone crosspeaks in two-dimensional ^{1}H,^{15}N-HSQC- or HMQC-type spectra were assigned (Fig. 8; Fig. S13), mostly through superposition with NMR spectra for various subconstructs (Supporting Materials and Methods, Section 1.4).

For γSA, the well-resolved crosspeak for C-terminal-linker residue Gly777, which has a relatively narrow line shape, is particularly suited for analysis (Fig. 8). The nonphospho-, and separately the phospho-, protein crosspeaks almost coincide for WT and Arg687Trp tandem SH2 and γSA proteins but, because of their relative positions, indicate weaker association of the cSH2-SH3 linker (which corresponds to the C-terminus in the context of NSH2CSH2) in γSA compared to the respective tandem nSH2-cSH2

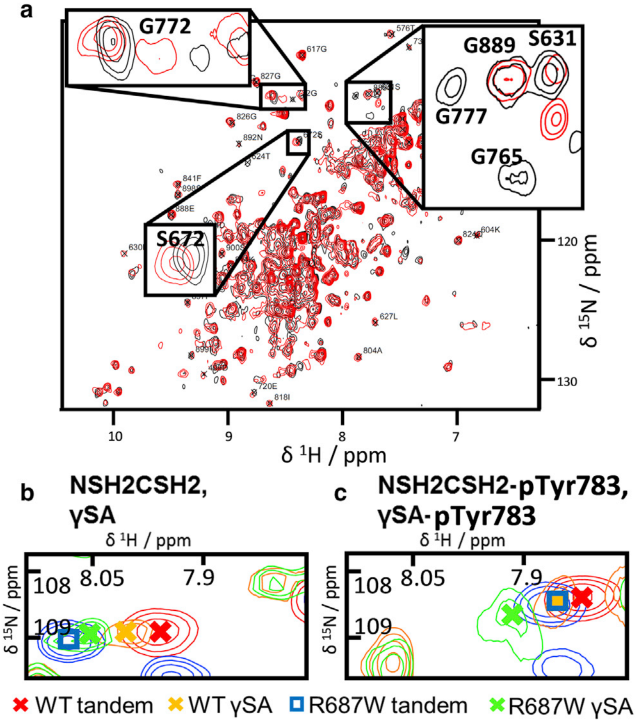

FIGURE 8 The NMR observations made in context of tandem- nSH2-cSH2 constructs are reflected in ^{1}H,^{15}N-NMR spectra for the γ-specific array (γSA). (*a*) ^{1}H,^{15}N-SOFAST-HMQC spectra of γSA (*red*) and γSA-pY783 (*black*) are shown. Spectra were recorded at 950 MHz at 25°C. Further backbone crosspeak assignments for nonphosphorylated γSA can be found elsewhere (10). (*b* and *c*) ^{1}H,^{15}N-SOFAST-HMQC crosspeaks of residue Gly777 in nonphosphorylated (*b*) and phosphorylated (*c*) tandem- or γSA constructs are shown. Symbols depict the crosspeak centroids (*color* key depicted below). The "closed" state is less populated in nonphosphorylated and R687W mutant constructs than in the corresponding phosphorylated and WT constructs. Spectra were recorded at 34°C (*b*) or 25°C (*a* and *c*) and at 700 MHz (*b*) or 950 MHz (*a* and *c*).

proteins. However, the relative (phospho- vs. nonphospho-, WT versus mutant) CSPs within each construct group (γSA and tandem SH2) are essentially identical, indicating that the underlying source of the shift difference is present in both the tandem and γSA contexts. Further, CSP analysis for residues Ser672, Gly765, Gly772, and Gly777 (Fig. 8; Fig. S13) confirms that the "closed" population differences between nonphospho- and phospho-constructs and between WT and Arg687Trp constructs apply in the γSA context.

DISCUSSION

We and others have demonstrated that phosphorylation of Tyr783 in the cSH2-SH3 linker of PLCγ1 has a critical regulatory role in the mechanism of activation of the enzyme (10). Here, MD simulations of PLCγ1 tandem SH2 constructs were analyzed to interrogate the potential impact of phosphorylation on NSH2CSH2 structure and dynamics. PCA suggested significant differences between correlated motions in NSH2CSH2 and NSH2CSH2-pY^{783}. Specifically, differences were detected between the one-dimensional projections of NSH2CSH2 and NSH2CSH2-pY^{783}-tandem trajectories on the eigenvector describing motions of the C-terminus and the pre-C-terminus. Contact probabilities for NSH2CSH2 and NSH2CSH2-pY^{783} differ for the following pairs: extended C-terminus/cSH2, extended C-terminus/nSH2-cSH2 junction, and nSH2-cSH2 junction/cSH2 α_B-helix. Side-chain mutual information analysis, which can be used to detect correlations between allosteric sites (18), suggests the presence of an allosteric connection between the C-terminus and the nSH2-cSH2 junction. These observations spurred the experimental analysis of the set of tandem SH2 protein constructs by heteronuclear NMR, with the aim to validate the predictions.

Naïvely, Tyr783 phosphorylation would be expected to lead to binding of the Tyr783 peptide region to the phosphopeptide binding site on the cSH2 domain. In contrast, NMR data show that Tyr783 phosphorylation leads to changes in structure and dynamics in the cSH2 domain that are more extensive. Importantly, a substantial proportion of these changes depend on the presence of the C-terminal linker. The chemical shift patterns observed for many residues in different constructs can be rationalized by invoking fast averaging between "open" and "closed" states. Significantly, it is not possible to rationalize the fast-exchange kinetics in terms of a single binding equilibrium; rather, the data suggest that different parts of the C-terminus are more strongly associated with the surface of the cSH2 domain than others.

A previous study showed that the presence of the C-terminus modulates the chemical shifts of multiple cSH2 domain crosspeaks; therefore, the C-terminus was thought to be bound to the cSH2 domain even in the absence of Tyr783 phosphorylation (16). However, our observations obtained for a range of WT and mutant phospho- and nonphospho-constructs indicate that the C-terminus is only partially bound in the latter case. That the binding is not complete is relevant in the context of the supposed association of the cSH2 domain with the PLCγ1 enzyme core, leading to autoinhibition in the nonphospho-state (10) because a fully bound C-terminus would impede the molecular interaction of the cSH2 domain with the core. Moreover, it has been suggested that interactions of the cSH2 domain with two FGFR kinase domains in the supramolecular complex arising from FGF ligand-receptor association are part of the activation mechanism for PLCγ1 (12). Whether or not these interactions are involved in PLCγ1 activation, each requires that the cSH2 domain exhibit at least transient exposure of the requisite interaction surface. Because the interactions are expected to take place at the cSH2 pTyr-binding site, there would be a requirement that the cSH2-SH3 linker can be readily displaced from the binding site, which is in agreement with our findings for transient association in both tandem SH2 and γSA proteins.

Tyr783 phosphorylation leads to CSPs for nSH2-cSH2 junction residue Thr660, which is at >20 Å distance, strongly suggesting an allosteric effect. A direct allosteric communication via the C-terminal linker can be postulated when including the "pre-C-terminus" region that is predicted by both crystallographic analysis and MD simulations to come into contact with the nSH2-cSH2 interdomain junction. The chemical shift patterns for many cSH2 residues indicate that Tyr783 phosphorylation affects the relative populations rather than the structures of the pre-existing "open" and "closed" states. Importantly, the fast-exchange "open" $\rightleftharpoons$ "closed" equilibrium is modulated in various mutant constructs; for each construct, we observed a global tendency to shift residues toward either the "open" or "closed" state. A substantial part of the change triggered by Tyr783 phosphorylation can therefore be described as dynamic allostery (24–26).

The dynamic allosteric connection is attributed to the C-terminus and the pre-C-terminus of the tandem domain constructs; in particular, the nature of the allostery depends on the presence of the C-terminal linker. The additional observation that the populations of the local "open" and "closed" states differ for various C-terminal residues implies that the overall number of conformations is large. The high number of degrees of freedom suggests that entropic considerations could be particularly relevant in the allosteric communication of Tyr783 phosphorylation as well as in potential interactions of a binding partner with the C-terminus or the cSH2 domain (26). These intermolecular interactions could be controlled by dynamic interactions of the C-terminus with the cSH2 domain, a situation that would be an example of a "fuzzy" intramolecular complex (27).

Identification of the dynamic, C-terminal-linker-dependent allosteric effect of Tyr783 phosphorylation should

improve the understanding of the effect of mutations in the cSH2 domain. We have studied the dynamic impact of the activating, disease-relevant Arg687Trp mutation by MD simulations and NMR. We find that within the context of the tandem nSH2-cSH2 and γSA proteins, the Arg687Trp change shifts the extended C-terminus toward the "open" state and modulates the dynamic C-terminal-linker-dependent allosteric pathway. Distinct hypotheses emerge for how these dynamic and structural changes lead to the activating effect of the Arg687Trp substitution. Perturbation of the nSH2-cSH2 junction structure or stability could lead to an alteration in the relative orientation of or flexibility between the nSH2 and cSH2 domains; alternatively, the weakened interaction between the cSH2 domain and the extended C-terminus, which corresponds to the tether between the cSH2 with SH3 domains in the γSA, might be key to the mutant behavior. The consequences of Arg687Trp could include changes in interactions of the γSA with RTKs, with the enzyme core, or, in the case of Arg665Trp in PLCγ2, with Rac2.

Herein, we have presented a system in which dynamic allosteric communication is directly observable by monitoring ^{1}H,^{15}N-HSQC peak shifts, representing population shifts between fast-exchanging locally bound and unbound states. Our approach is related to the chemical shift projection analysis procedure (28) to reveal allosteric networks, which is distinct from chemical shift covariance analysis in that we focus mostly on population shifts of fast-exchanging residues, which helps avoid false positives (20). Our work also reveals the presence of a complex set of slow-exchange phenomena that combines with fast exchange and thus leads to unusual NMR crosspeak patterns, with two or more crosspeaks representing two fast-exchanging but differentially populated states for a single residue. In particular, we rationalize the spectral characteristics in terms of "remote" slow-exchange equilibria that determine the population of "locally" fast-exchanging "open" ⇌ "closed" populations (Fig. 7). The system is conceptually similar to the population shuffling of rotamer conformations that was recently described for protein G and ubiquitin (29), though it remains to be investigated whether the underlying mechanistic processes in PLCγ are mutually independent. A schematic that aims to capture the different exchange processes at play in this system is presented in Fig. 9. We emphasize that the apparent complexity of the tandem nSH2-cSH2 system described herein emerged only by examination of a relatively large number of protein variants. It is possible that other unrelated systems could also encompass such multitimescale dynamic allostery that may go undetected in the absence of appropriate variant constructs. We note that the PLCγ1 system described here should lend itself to quantitative exploration of the *N*-state kinetics that underlies dynamic allosteric communication using state-of-the-art methods in NMR relaxation experiments over a wide range of timescales (30,31).

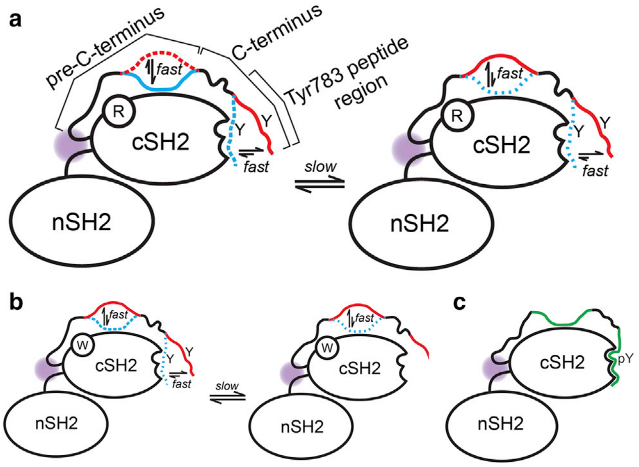

FIGURE 9 Scheme illustrating the interpretation of the combination of slow- and fast-exchange phenomena present in various tandem SH2 constructs. (*a*) NSH2CSH2, (*b*) R687W-NSH2CSH2, and (*c*) NSH2CSH2-pY^{783} are shown. The regional variation of kinetic equilibria throughout the extended C-terminus is indicated. Red and blue colors refer to parts of the extended C-terminus in fast-exchanging "open" and "closed" states, respectively. The relative populations of the different chain segments in the extended C-terminus are symbolized by colored solid (high), dotted (medium), or sparsely dotted (low) chain segments. (*a*) For NSH2CSH2, the pattern of crosspeaks suggests underlying fast-exchange processes attributable to fleeting contacts of the cSH2 domain with different segments of the extended C-terminus; additional peak doubling indicates slow exchange between states, potentially associated with *cis-/trans*-peptide bond isomerization. (*b*) For R687W-NSH2CSH2, a similar mixture of slow and fast exchange pertains, though the affinity of the cSH2 domain for the extended C-terminus appears weaker. (*c*) For NSH2CSH2-pY^{783}, the strong interaction between the cSH2 pTyr783 binding site and the extreme pY783 peptide region at the C-terminus cooperates with the binding of other regions of the C-terminus and the pre-C-terminus (all depicted in *green color*) to the "body" of the cSH2 domain. In each case, long-range effects involving the interactions of the extended C-terminus were evident in the NMR characteristics of crosspeaks for residues located at the nSH2-cSH2 junction, indicated by purple shading.

CONCLUSIONS

This work has highlighted a hitherto unappreciated and complex pattern of allosteric influences within the receptor adaptor domains of PLCγ1 that are likely relevant for experiments aimed at elucidating its regulation in both space and time at any greater length scale (e.g., with full-length proteins in a whole cell context). MD simulations suggested structural connectivity between occupation of the canonical cSH2 phosphopeptide binding site and the nSH2-cSH2 interdomain junction. As part of the experimental validation of the key MD predictions, we uncovered a surprisingly complex set of NMR characteristics for the tandem SH2 domain protein. The MD simulations also provided an invaluable context within which to effectively design and assess tandem SH2 domain variants to decipher the emergent NMR characteristics of allostery in the cSH2 domain. We have shown that the C-terminus is partially bound in the nonphosphorylated state and that presence of the nSH2 domain changes the dynamic properties of the cSH2

domain. The comparison of multiple protein variants revealed evidence for allosteric connectivity that importantly involves the polypeptide tether between the globular part of the cSH2 domain and the Tyr783 phosphorylation site. The allosteric connection is dynamic in nature and involves the presence of multiple combined fast- and slow-exchange processes. We have demonstrated that the disease-relevant mutation Arg687Trp weakens the dynamic allosteric connection (Fig. 9), provoking specific hypotheses about the mechanistic basis for the effect of this substitution; it remains to be determined which particular section of the dynamic allosteric arc spanning the long extended C-terminus and the nSH2-cSH2 junction is functionally relevant in context of holo-PLCγ1. Importantly, the modulated binding of the extended C-terminus in Arg687Trp mutants was also detected in the context of the larger γSA fragment of PLCγ1, indicating that the impact of the dynamic modulation extends beyond tandem SH2 constructs and should be taken into account in any examination of the upstream regulation of PLCγ proteins by RTKs and small G-proteins.

SUPPORTING MATERIAL

Supporting Materials and Methods, Supporting Results, fifteen figures, and two data files are available at http://www.biophysj.org/biophysj/supplemental/S0006-3495(18)30670-2.

AUTHOR CONTRIBUTIONS

H.K., M.K., and P.C.D. designed the research. H.K. performed and analyzed the MD simulations. H.K. and T.D.B. performed the mutagenesis experiments. H.K. and T.D.B. expressed and purified proteins. H.K. and D.E. performed the NMR experiments. H.K. analyzed the NMR data. M.M. conducted the enzyme activity measurements. H.K. and P.C.D. wrote the article.

ACKNOWLEDGMENTS

We thank Willa Yim for assisting with the expression and purification of FGFR1 kinase protein and helpful discussions. We thank the staff of the Medical Research Council Biomedical NMR Centre for assistance and Arthur G. Palmer for a critical reading of the article. Small-angle x-ray scattering data for Section 4 were recorded at the Deutsche Elektronen-Synchrotron facility using synchrotron radiation from the DORIS III electron-positron storage ring, and we thank Dr. Dmitri Svergun and Melissa Gräwert for discussion and support.

Financial support for this work was from the Wellcome Trust (PhD studentship 096617/Z/11/Z to H.K.) and the Medical Research Council.

SUPPORTING CITATIONS

References (32–52) appear in the Supporting Material.

REFERENCES

1. Bunney, T. D., and M. Katan. 2011. PLC regulation: emerging pictures for molecular mechanisms. *Trends Biochem. Sci.* 36:88–96.
2. Suh, P. G., J. I. Park, …, S. H. Ryu. 2008. Multiple roles of phosphoinositide-specific phospholipase C isozymes. *BMB Rep.* 41:415–434.
3. Rebecchi, M. J., and S. N. Pentyala. 2000. Structure, function, and control of phosphoinositide-specific phospholipase C. *Physiol. Rev.* 80:1291–1335.
4. Koss, H., T. D. Bunney, …, M. Katan. 2014. Dysfunction of phospholipase Cγ in immune disorders and cancer. *Trends Biochem. Sci.* 39:603–611.
5. Walliser, C., M. Retlich, …, T. D. Bunney. 2008. rac regulates its effector phospholipase Cgamma2 through interaction with a split pleckstrin homology domain. *J. Biol. Chem.* 283:30351–30362.
6. Bae, J. H., E. D. Lew, …, J. Schlessinger. 2009. The selectivity of receptor tyrosine kinase signaling is controlled by a secondary SH2 domain binding site. *Cell.* 138:514–524.
7. Poulin, B., F. Sekiya, and S. G. Rhee. 2005. Intramolecular interaction between phosphorylated tyrosine-783 and the C-terminal Src homology 2 domain activates phospholipase C-gamma1. *Proc. Natl. Acad. Sci. USA.* 102:4276–4281.
8. Gresset, A., S. N. Hicks, …, J. Sondek. 2010. Mechanism of phosphorylation-induced activation of phospholipase C-gamma isozymes. *J. Biol. Chem.* 285:35836–35847.
9. DeBell, K., L. Graham, …, B. Rellahan. 2007. Intramolecular regulation of phospholipase C-gamma1 by its C-terminal Src homology 2 domain. *Mol. Cell. Biol.* 27:854–863.
10. Bunney, T. D., D. Esposito, …, M. Katan. 2012. Structural and functional integration of the PLCγ interaction domains critical for regulatory mechanisms and signaling deregulation. *Structure.* 20:2062–2075.
11. Hajicek, N., T. H. Charpentier, …, J. Sondek. 2013. Autoinhibition and phosphorylation-induced activation of phospholipase C-γ isozymes. *Biochemistry.* 52:4810–4819.
12. Huang, Z., W. M. Marsiglia, …, M. Mohammadi. 2016. Two FGF receptor kinase molecules act in concert to recruit and transphosphorylate phospholipase Cγ. *Mol. Cell.* 61:98–110.
13. Zhou, Q., G. S. Lee, …, I. Aksentijevich. 2012. A hypermorphic missense mutation in PLCG2, encoding phospholipase Cγ2, causes a dominantly inherited autoinflammatory disease with immunodeficiency. *Am. J. Hum. Genet.* 91:713–720.
14. Behjati, S., P. S. Tarpey, …, P. J. Campbell. 2014. Recurrent PTPRB and PLCG1 mutations in angiosarcoma. *Nat. Genet.* 46:376–379.
15. Woyach, J. A., R. R. Furman, …, J. C. Byrd. 2014. Resistance mechanisms for the Bruton's tyrosine kinase inhibitor ibrutinib. *N. Engl. J. Med.* 370:2286–2294.
16. Devkota, S., R. E. Joseph, …, A. H. Andreotti. 2015. Scaffold protein SLP-76 primes PLCγ1 for activation by ITK-mediated phosphorylation. *J. Mol. Biol.* 427:2734–2747.
17. Eck, M. J., S. Pluskey, …, S. E. Shoelson. 1996. Spatial constraints on the recognition of phosphoproteins by the tandem SH2 domains of the phosphatase SH-PTP2. *Nature.* 379:277–280.
18. McClendon, C. L., G. Friedland, …, M. P. Jacobson. 2009. Quantifying correlations between allosteric sites in thermodynamic ensembles. *J. Chem. Theory Comput.* 5:2486–2502.
19. Siegal, G., B. Davis, …, P. C. Driscoll. 1998. Solution structure of the C-terminal SH2 domain of the p85 alpha regulatory subunit of phosphoinositide 3-kinase. *J. Mol. Biol.* 276:461–478.
20. Boulton, S., M. Akimoto, …, G. Melacini. 2014. A tool set to map allosteric networks through the NMR chemical shift covariance analysis. *Sci. Rep.* 4:7306.
21. Walliser, C., E. Hermkes, …, P. Gierschik. 2016. The phospholipase Cγ2 mutants R665W and L845F identified in ibrutinib-resistant chronic lymphocytic leukemia patients are hypersensitive to the Rho GTPase Rac2 protein. *J. Biol. Chem.* 291:22136–22148.
22. Alderson, T. R., J. H. Lee, …, A. Bax. 2018. Propensity for cis-proline formation in unfolded proteins. *ChemBioChem.* 19:37–42.
23. Wüthrich, K. 1976. NMR in Biological Research: Peptides and Proteins. North Holland Publishing Company, Amsterdam, The Netherlands.

24. Tzeng, S. R., and C. G. Kalodimos. 2011. Protein dynamics and allostery: an NMR view. *Curr. Opin. Struct. Biol.* 21:62–67.
25. Cooper, A., and D. T. Dryden. 1984. Allostery without conformational change. A plausible model. *Eur. Biophys. J.* 11:103–109.
26. Wand, A. J. 2001. Dynamic activation of protein function: a view emerging from NMR spectroscopy. *Nat. Struct. Biol.* 8:926–931.
27. Fuxreiter, M., and P. Tompa. 2012. Fuzzy complexes: a more stochastic view of protein function. *Adv. Exp. Med. Biol.* 725:1–14.
28. Selvaratnam, R., B. VanSchouwen, …, G. Melacini. 2012. The projection analysis of NMR chemical shifts reveals extended EPAC autoinhibition determinants. *Biophys. J.* 102:630–639.
29. Smith, C. A., D. Ban, …, D. Lee. 2015. Population shuffling of protein conformations. *Angew. Chem. Int.Engl*. 54:207–210.
30. Palmer, A. G., III, C. D. Kroenke, and J. P. Loria. 2001. Nuclear magnetic resonance methods for quantifying microsecond-to-millisecond motions in biological macromolecules. *Methods Enzymol.* 339:204–238.
31. Koss, H., M. Rance, and A. G. Palmer, III. 2017. General expressions for $R_{1\rho}$ relaxation for N-site chemical exchange and the special case of linear chains. *J. Magn. Reson.* 274:36–45.
32. Murray, V., Y. Huang, …, Q. Li. 2012. A novel bacterial expression method with optimized parameters for very high yield production of triple-labeled proteins. *Methods Mol. Biol.* 831:1–18.
33. Hess, B., C. Kutzner, …, E. Lindahl. 2008. GROMACS 4: algorithms for highly efficient, load-balanced, and scalable molecular simulation. *J. Chem. Theory Comput.* 4:435–447.
34. Humphrey, W., A. Dalke, and K. Schulten. 1996. VMD: visual molecular dynamics. *J. Mol. Graph.* 14:33–38, 27–38.
35. Schwieters, C. D., J. J. Kuszewski, …, G. M. Clore. 2003. The Xplor-NIH NMR molecular structure determination package. *J. Magn. Reson.* 160:65–73.
36. Lindorff-Larsen, K., S. Piana, …, D. E. Shaw. 2010. Improved side-chain torsion potentials for the Amber ff99SB protein force field. *Proteins.* 78:1950–1958.
37. Homeyer, N., A. H. Horn, …, H. Sticht. 2006. AMBER force-field parameters for phosphorylated amino acids in different protonation states: phosphoserine, phosphothreonine, phosphotyrosine, and phosphohistidine. *J. Mol. Model.* 12:281–289.
38. Mobley, D. L., J. D. Chodera, and K. A. Dill. 2006. On the use of orientational restraints and symmetry corrections in alchemical free energy calculations. *J. Chem. Phys.* 125:084902.
39. Schulte-Herbrüggen, T., and O. W. Sorensen. 2000. Clean TROSY: compensation for relaxation-induced artifacts. *J. Magn. Reson.* 144:123–128.
40. Eletsky, A., A. Kienhöfer, and K. Pervushin. 2001. TROSY NMR with partially deuterated proteins. *J. Biomol. NMR.* 20:177–180.
41. Hyberts, S. G., H. Arthanari, and G. Wagner. 2012. Applications of non-uniform sampling and processing. *Top. Curr. Chem.* 316:125–148.
42. Kazimierczuk, K., and V. Y. Orekhov. 2011. Accelerated NMR spectroscopy by using compressed sensing. *Angew. Chem. Int. Ed. Engl.* 50:5556–5559.
43. Kay, L. E., D. A. Torchia, and A. Bax. 1989. Backbone dynamics of proteins as studied by 15N inverse detected heteronuclear NMR spectroscopy: application to staphylococcal nuclease. *Biochemistry.* 28:8972–8979.
44. Delaglio, F., S. Grzesiek, …, A. Bax. 1995. NMRPipe: a multidimensional spectral processing system based on UNIX pipes. *J. Biomol. NMR.* 6:277–293.
45. Orekhov, V. Y., and V. A. Jaravine. 2011. Analysis of non-uniformly sampled spectra with multi-dimensional decomposition. *Prog. Nucl. Magn. Reson. Spectrosc.* 59:271–292.
46. Vranken, W. F., W. Boucher, …, E. D. Laue. 2005. The CCPN data model for NMR spectroscopy: development of a software pipeline. *Proteins.* 59:687–696.
47. Williamson, M. P. 2013. Using chemical shift perturbation to characterise ligand binding. *Prog. Nucl. Magn. Reson. Spectrosc.* 73:1–16.
48. Everett, K. L., T. D. Bunney, …, M. Katan. 2009. Characterization of phospholipase C gamma enzymes with gain-of-function mutations. *J. Biol. Chem.* 284:23083–23093.
49. Svergun, D. I., and M. H. Koch. 2002. Advances in structure analysis using small-angle scattering in solution. *Curr. Opin. Struct. Biol.* 12:654–660.
50. Bernadó, P., E. Mylonas, …, D. I. Svergun. 2007. Structural characterization of flexible proteins using small-angle X-ray scattering. *J. Am. Chem. Soc.* 129:5656–5664.
51. Rossi, P., G. V. Swapna, …, G. T. Montelione. 2010. A microscale protein NMR sample screening pipeline. *J. Biomol. NMR.* 46:11–22.
52. Pascal, S. M., A. U. Singer, …, J. D. Forman-Kay. 1994. Nuclear magnetic resonance structure of an SH2 domain of phospholipase C-gamma 1 complexed with a high affinity binding peptide. *Cell.* 77:461–472.

Biophysical Journal, Volume *115*

Supplemental Information

Dynamic Allostery in PLCγ1 and Its Modulation by a Cancer Mutation Revealed by MD Simulation and NMR

Hans Koss, Tom D. Bunney, Diego Esposito, Marta Martins, Matilda Katan, and Paul C. Driscoll

S1. Materials and Methods

S1.1 Cloning, mutagenesis, protein expression and purification

PLCγ1 constructs used in this work are all human and generally based on the NCBI sequence NP_002651.2. All PLCγ1 constructs were expressed from pOPINS (Oxford Protein Production Facility) plasmids using *E. coli* strain C41 (DE3) (Lucigen). For a definition of construct boundaries, see first paragraph in the results section and *Fig. 1b*. Protein constructs include an N-terminal 6xHis-SUMO tag. For improved comparability with NSH2CSH2-pY^{783}, NSH2CSH2 "WT" constructs carry Tyr771,775Phe mutations, which do not change any relevant peak positions in ^{1}H,^{15}N-HSQC spectra. The plasmids for NSH2CSH2 (545-790), NSH2CSH2$^{\Delta CT}$ (545-770), γSA WT (488-933), γSA Tyr771Phe (488-933) and all cSH2 constructs (663-790) were part of the lab inventory and created in context of a previous study (Bunney et al 2012).

Mutagenesis reactions were performed using the Thermo Scientific Pfu DNA Polymerase kit to create constructs with Arg687Trp or/and Tyr771,775Phe mutations. Per 25 µl reaction, 62.5 ng of both forward and reverse oligonucleotides were added; Tyr771,775Phe mutants were created in a one-step mutagenesis reaction.

Oligonucleotides for mutagenesis reactions were ordered from Invitrogen. Forward sequences are:
R687W (PLCγ1): CTAATGCGCGTCCCTTGGGATGGGGCCTTCCTGG
R665W (PLCγ2): GATGAGGATTCCCTGGGACGGGGCCTTCC
E664G (PLCγ1): CAGACCAACGCCCACGGGAGCAAAGAGTGG
E664K (PLCγ1): CAGACCAACGCCCACAAGAGCAAAGAGTGG
Y771,775F (PLCγ1): GACTTCGGGGCCCTGTTTGAGGGACGCAACCC).

For sequencing of PLCγ constructs, a T7 forward primer, a set of internal forward primers and a SUMO-tag primer were used. For the construction of other plasmids refer to Bunney et al. (10).

The following procedure was used to produce ^{15}N or ^{13}C,^{15}N-labelled protein. Liquid cultures were generally shaken at 200 rpm and baffled flasks were used for expression. Expression yields were improved by taking into account suggestions from Murray et al. (32). The pOPINS vector harboring the construct was transformed into E. coli strain C41 (DE3). Eight colonies were picked and incubated to 37 °C for 9 hours in a 2 l flask with 500 ml 2xYT/Kana, then pelleted and resuspended in a 2l flask with 500 ml minimal medium (90 mM Na_2HPO_4, 22 mM KH_2PO_4, 8.5 mM NaCl, 7.5 mM ^{15}N-$(NH_4)_2SO_4$ 56 mM D-Glucose, 0.5 x Trace Metal Mix, 50 µg/ml Kanamycin, 20 mg/l Thiamin, 20 mg/l Biotin, 2 mM $MgCl_2$, pH 7.4). Glucose concentration was set to 11 mM for ^{13}C-labelled proteins (substituting regular glucose with D-Glucose-$^{13}C_6$). The flask was kept at 37 °C for one hour and then cooled to 20 °C for one hour. Protein expression was induced with 100 µM IPTG and expressed at 20 °C for 10 hours. Bacteria were pelleted and kept frozen at -80 °C. For expression in 100% D_2O, the

culture was pre-grown in a small volume of 100% D_2O prior to expression; expression time was 20 hours.

Frozen pellets from a 500 ml – 2000 ml culture were used in the purification procedure; larger cultures up to 6 l were split accordingly, and pooled after His tag cleavage. All steps were performed at 4 °C. 15 ml (1000 ml culture: 30 ml) lysis buffer (25 mM TrisCl, pH 8.0, 250 mM NaCl, 40 mM imidazole, 10 mM benzamidine, 1 mM $MgCl_2$, 10 μM $CaCl_2$, lysozyme) was added to frozen pellets in large-diameter centrifugation vessels (500 ml or 1000 ml culture). The vessels were placed on a shaker (200 rpm) for 30 minutes to 1 hour. 3.5 ml (1000 ml culture: 7.5 ml) 10% (v/v) Triton X-100 (needs about one hour to dissolve) and 75 μl (1000 ml culture: 150 μl) Dnase I (bovine pancrease) from glycerated solution were added. The vessels were shaken for another hour and then centrifuged for one hour (Beckmann Coulter Avanti K-20XP, JS-25.50 rotor, 12000 rpm). The supernatant was used for further purification using the AKTA Explorer or Purifier system (GE Healthcare). A HisTrap (GE Healthcare, individual columns for distinct proteins) column and His buffers A (25 mM TrisCl, pH 8.0, 500 mM NaCl, 40 mM imidazole, 1 mM TCEP) and B (25 mM TrisCl, pH=8.0, 500 mM NaCl, 500 mM imidazole, 1 mM TCEP) were used for the first purification step (10 column volumes of His buffer A, then 5 column volumes linear gradient to His buffer B). The His-SUMO tag of collected sample was then cleaved by adding 100 μl of 5 mg/ml Ulp1 protease and dialyzing (MW 10 kDa tubing) against His Chelating Buffer (25 mM TrisCl, pH=8.0, 250 mM NaCl, 20 mM imidazole, 1 mM TCEP) overnight (at least 14 hours). For the second purification step a HisTrap Chelating column (GE Healthcare, the same column for all proteins) was used (same conditions as for the first purification step, but use filtered Chelating buffer instead of His buffer A). The collected sample was dialyzed (MW 10 kDa tubing) against Low Salt buffer for at least 4 hours (25 mM TrisCl, pH 8.0, 1 mM TCEP). A HiTrap Q column (GE Healthcare) and Q buffers A (25 mM TrisCl, pH=8.0, 20 mM NaCl, 1 mM TCEP) and B (25 mM TrisCl, pH 8.0, 1M NaCl, 1 mM TCEP) were used for the third purification step (first Q buffer A, then linear gradient to 50% Q buffer B over 25 column volumes). Elution from a Superdex 75 26/60 column was performed prior to the final spin concentration step. In a protocol variation without gel filtration, the buffer was exchanged by spin-concentrating (Vivascience; tandem SH2: 10 kDa filters; γSA: 30 kDa filters) and diluting thrice with 20 ml NMR buffer (25 mM Na_2HPO_4/NaH_2PO_4, pH=6.5, 50 mM NaCl, 5 mM DTT, 1 mM EDTA; in the first two runs concentrated 500 μl; the last run to 200-300 μl, if possible). The sample was snap-frozen and stored at -80 °C.

E664G-NSH2CSH2, E664K-NSH2CSH2 and cSH2 mutant constructs were expressed according to the similar, previously published procedure (10).

S1.2 Tyr783 Phosphorylation of proteins

FGFR1 kinase (464-775) with mutations only leaving a single, functionally relevant phosphorylation site was expressed and purified according to the procedure published previously (10). ^{15}N- or ^{13}C,^{15}N-labelled NSH2CH2 or γSA (as always with Tyr771Phe and Tyr775Phe mutations) was used for phosphorylation. 200 µl Strepactin Macroprep beads (IBA) were washed three times with phosphorylation buffer A (25 mM TrisCl, 150 mM NaCl, 1 mM TCEP, pH = 8.0) and incubated for 5 minutes with 0.4 mg FGFR1 kinase. The identical volume phosphorylation buffer B (25 mM TrisCl, 150 mM NaCl, 1 mM TCEP, 20 mM ATP, 50 mM MgCl2, pH 8.0) was added to obtain an ATP concentration of 10 mM; the solution was kept at room temperature for 10 minutes. 10 mg tandem SH2 was then added. The sample was left for 48 hours in the cold (4 °C). After centrifugation at low speed to precipitate the beads, the supernatant was applied to a gel filtration procedure equivalent to the procedure described in the purification protocol (see above).

S1.3 Molecular dynamics simulations

All molecular dynamics simulations were performed in Gromacs v. 4.5.4 (33) and resulting trajectories processed either with the same package or with VMD (34).

NSH2CSH2 (PDB ID: 4FBN) and NSH2CSH2-pY^{783} (PDB: 4EY0) crystal structures were taken as starting structures after modelling missing residues and performing an energy minimization with Xplor-NIH v. 2.3.8 (35); the Arg687Trp-mutated constructs were also generated with Xplor-NIH v. 2.3.8.

The AMBER ff99SB-ILDN force field (36) was used for all simulations. A phosphotyrosine residue topology was not available in GROMACS, therefore an AMBER parameter set for phosphotyrosine residues (37) was integrated into GROMACS using a conversion script (38). The phosphotyrosine residue side chain was assumed to be in a nonprotonated state.

The protein was set to be in a dodecahedral box; the box dimensions were 2 nm plus the maximum diameter of the protein. TIP3P was chosen as a water model, and the NaCl concentration was set to 150 mM. The following runs were performed for preparation: 1) L-BFGS energy minimization, timestep 0.001 ps, 5 ps duration, protein atom positions restrained; 2) L-BFGS energy minimization, timestep 0.001 ps, 10 ps duration, protein atom positions not restrained; 3) equilibration run 1 - leap frog integrator, timestep: 0.0005 ps, simulation duration: 100 ps, protein atom positions restrained. Coulomb interactions: Particle-Mesh Ewald; short range: 0.95 nm. Van-der-Waals-type: cut-off (0.95 nm). NPT ensemble; velocity-rescale thermostat: t_τ = 0.1 ps, reference temperature = 300 K; Berendsen barostat, isotropic, separate protein / non-protein coupling, t_P = 1.5 ps, compressibility = 4.5 10^{-5} bar^{-1}, reference pressure = 1.0 bar; 4) equilibration run 2: equal to equilibration run 1, but no protein atom position restrains, timestep: 0.002 ps; 5) equilibration run 3: leap frog integrator,

timestep: 0.002 ps, simulation duration: 100 ps, protein atom positions not restrained. Coulomb interactions: Particle-Mesh Ewald; short range: 1.0 nm. Van-der-Waals-type: cut-off (1.0 nm). NVT ensemble; Berendsen thermostat: t_τ = 1.0 ps, reference temperature = 298 K; 6) The production run was performed with the same parameters as the last equilibration run, but longer (up to 100 ns). Our local cluster was used (per calculation 2x8 cores, with LAM-MPI, efficiency: ~ 8.5 ns simulation / (day * 16 cores * ~ 50,000 atoms).

Contact events between two regions (any number of residues) were identified using the GROMACS g_mindist function (cutoff 2.5 Å), a bash script for this is provided in *Suppl. Inf. 3.1*. At a given frame, exactly one contact event involving two regions can occur: Any number of contacts (< 2.5 Å) at a given time point is counted as a single contact event. The contact events are added and normalized to the maximum number of contact events to give a contact probability, using a sliding average window of 1 ns.

For principle component analysis, the GROMACS 4.5.4 g_anaeig tool was used. In order to determine whether a certain eigenvector of one structure (obtained from the merged trajectory) can be used to describe the motions in a given trajectory of the same or another structure, the eigenvalue range of this given trajectory, projected on the eigenvector in question, can be estimated. The reference eigenvalue range is defined by the projection of the original merged trajectory used to generate the eigenvector in question. The projection operation was performed for any trajectory on any of the eigenvectors extracted from the three to six merged trajectories of the four different structures (three NSH2CSH2, four NSH2CSH2-pY^{783}, three R687W-NSH2CSH2-pY^{783}, three R687W-NSH2CSH2). Average values and standard deviations for the eigenvalue minimum and maximum for each structure projected on each eigenvector were determined using all non-merged trajectories. In order to compare 1D projections of trajectory sets on eigenvectors, a custom-made script (provided in *Suppl. Inf. 3.2*) was used.

Mutual information analysis: Six (NSH2CSH2) or three (NSH2CSH2-pY^{783}, R687W-NSH2CSH2-pY^{783}, R687W-NSH2CSH2) 100 ns trajectories were used for analysis. From each of these trajectories, six 10 ns bins starting at 40 ns were extracted, yielding 36 (NSH2CSH2) or 18 (others) 10 ns bins. The tool Mutinf (18) typically uses a combination of several 10 ns blocks for analysis. We performed 10 Mutinf runs using 5 randomly selected blocks (options –n 5 and –o 5), generating resampled datasets. The required side chain angle files were obtained from the trajectories using the Gromacs tool g_chi, with the following options: -maxchi 6 -phi -psi -all. The Mutinf scripts were compiled and run on a dedicated, custom-modified Linux Ubuntu machine, following the provided instructions. Residue Y783/pY783 was excluded from analysis. Mutinf running options were: -g gcc -a “yes” -o 5 -w 30 -o 0 -n 5. The mutual information sums for each residue, without Wilcoxon test, were collected to then have 10 mutual information data points for each residue. The

results are shown unfiltered in this paper. To establish the relevance of a difference in mutual information between two constructs (for example NSH2CSH2 vs. NSH2CSH2-pY^{783}), a two-sided t-test (unequal population variances, Welch's t-test) was performed, using the SEM obtained from 10 data points, obtained from Mutinf runs which were based on resampled data. Residue-wise mutual information differences between two constructs are only shown if this test returns that they are not equal with $p > 0.95$. The python data processing and visualization script, starting from Mutinf results, is provided in *Suppl. Inf. 3.3*.

S1.4 Nuclear magnetic resonance spectroscopy: Backbone resonance assignment and ^{1}H,^{15}N-heteronuclear correlation experiments

Bruker Avance III (600, 700, 800 and 950 MHz) NMR spectrometers equipped with cryogenically cooled triple resonance probes with a z-axis pulse field gradient coil were used for all NMR experiments. Spectra were recorded at 25 °C in NMR buffer (25 mM Na_2HPO_4/NaH_2PO_4, pH 6.5, 50 mM NaCl, 5 mM DTT, 1 mM EDTA and 9% D_2O), unless stated otherwise. Shigemi NMR tubes were used to maximize protein concentration for most experiments, because sample availability rather than solubility is a limiting factor. Protein concentrations were usually 100 - 250 μM; results in this paper are concentration-independent.

A standard set of 3D backbone resonance assignment spectra (HNCA, HNCOCA, HNCACB, CACBCONH, HNCO and HNCACO) using Bruker library pulse sequences (with Watergate water suppression) were recorded for ^{15}N,^{13}C-labelled NSH2CSH2 and NSH2CSH2-pY^{783} samples; HNCA spectra were also recorded for NSH2CSH2$^{\Delta CT}$ and R687W-NSH2CSH2-pY^{783}. The respective ^{1}H,^{15}N-TROSY spectra using echo-antiecho and gradient water suppression were recorded for ^{2}H,^{15}N,^{13}C-labelled NSH2CSH2 (39, 40). Non-uniform sampling (NUS) was used to obtain a high-resolution HNCA spectrum for ^{2}H,^{15}N,^{13}C-nonphospho- and ^{15}N,^{13}C-NSH2CH2-pY^{783} (41, 42). The NUS fraction 17%, and the transverse relaxation time estimate for NUS was set to 8 ms.

^{1}H,^{15}N-HSQC-type experiments for γSA constructs displayed many more cross peaks when being recorded at 34 °C rather than at 25 °C. γSA-pY783 constructs were stable at 25 °C only for a relatively limited duration. Superposition of the recorded and assigned ^{1}H,^{15}N-HSQC NMR spectra for the isolated SH3, spPH, and NSH2CSH2 and NSH2CSH2-pY^{783} proteins (for spPH assignments see (5)), in combination with a 3D HNCA spectrum recorded for a Tyr771Phe-γSA construct allowed for transfer of some resonances. In some cases, the assignments were confirmed using a 3D HNCA spectrum recorded for a Tyr771Phe-γSA construct; only this particular γSA construct was available at a sufficiently high concentration and stability to record a 3D spectrum.

Bruker pulse sequences hsqcfpf3gpphwg (version 12/01/11) or sfhmqcf3gpph (version 13/02/28) were used to record ^{1}H,^{15}N-HSQC spectra and ^{1}H,^{15}N-SOFAST-HMQC spectra, respectively. For

NSH2CSH2$^{\Delta CT}$-CTpY samples, the pTyr783-peptide NPGFpYVEANPMP (PLCγ 779-790) was added in excess prior to performing the experiment (10). The PDGFRβ peptide TSNQEpYLDLSM was obtained from Cambridge Peptides Ltd; for more information, see Bunney et al. (10).
Backbone resonance assignments were submitted to the BioMagResBank, accession number 27496.

S1.5 NMR data processing and analysis of fast exchange

NMR raw data were preprocessed with NMRPipe and NMRDraw (44). Non-uniform sampling data were analyzed using the iterative re-weighted least squares reconstruction algorithm (IRLS) (42) implemented in the MddNMR 2.1 (45) suite. NMR spectra were analyzed with CcpNmr Analysis (46). Chemical shift perturbation was calculated from ^{1}H and ^{15}N chemical shift differences by adjusting the ^{15}N shift differences using 0.14 as a scaling factor for ^{15}N shift changes (47).
For analysis of relaxation data, CcpNmr Analysis was used to pick cross peaks and fit peak heights to exponential decays to get the relaxation time constants T_1 and T_2. CcpNmr returns a fitting error that was used as the error for R_1 and R_2. The error was obtained from the fitting procedure.

S1.6 Cell culture, transfection, and fractionation.

COS-7 and HEK293 cells were maintained at 37°C in a humidified atmosphere of 95% air and 5% CO2 in Dulbecco's modified Eagle's medium (DMEM) (Invitrogen) supplemented with 10% (v/v) fetal bovine serum (Invitrogen) and 2.5 mM glutamine. Prior to transfection, cells were seeded into 6-well plates at a density of 2.5×10^5 cells/well and grown for 16 h in 2 ml/well of the same medium. For transfection of COS-7, 1.0 µg of PLCγ DNA was mixed with 1 µl PlusReagent and 7 µl Lipofectamine (Invitrogen) and the mixture added to the cells in 0.8 ml DMEM without serum. The cells were incubated for 3.5 h at 37°C, 5% CO_2 before the transfection mixture was removed and replaced with DMEM containing serum.

S1.7 Analysis of inositol phosphate formation in intact COS-7 cells.

This analysis was performed essentially as described previously (5, 48). Briefly, 24 h posttransfection, cells were labeled with 1.5 µCi/ml myo-[2-^{3}H]inositol. After a further 24 h, the cells were incubated in 1.2 ml inositol-free DMEM, without serum, containing 20 mM LiCl with or without stimulation with 100 ng/ml epidermal growth factor (EGF; Calbiochem) for 1 h. The cells were lysed by the addition of 1.2 ml 4.5% perchloric acid, and supernatants and pellets were separated. Inositol phosphates were collected using AG1-X8 200-400 columns (Bio-Rad). The levels of inositol phosphates were quantified by liquid scintillation counting using Ultima-Flo scintillation fluid (PerkinElmer). The PLC activity analyzed only by this standard measurement is given as "PLC activity

(cpm).” Data shown are the means ± SDs of triplicate samples and are representative of three or more independent experiments.

S1.8 Relaxation experiments

Relaxation data (^{15}N-R_1, ^{15}N-R_2) for NSH2CSH2 and NSH2CSH2-pY^{783} were collected at 600 MHz similar to a procedure described elsewhere (43) and based on Bruker pulse sequences hsqct2etf3gpsi3d.2 (version 04/01/05) and hsqct1etf3gpsi3d.2 (04/01/05). Recovery delays for ^{15}N-R_2 relaxation experiments were set to 8, 16, 24, 40, 56, 72, 96 and 120 ms or 8, 16 and 40 ms (for the NSH2CSH2 sample at 30 µM). Recovery delays for ^{15}N-R_1 relaxation experiments were set to 10, 100, 200, 300, 500, 700, 800 and 1200 ms. The delay time recording schedule was randomized with data recorded an in interleaved manner.

S1.9 Peptide titration experiments for K_D determination

Peptide titration experiments were performed at constant volume by titrating peptide into protein solution and recording either ^{15}N-HSQC or ^{15}N-SOFAST experiments. Due to peak broadening, overlap or complex exchange phenomena, not many peaks were eligible for quantitative analysis. The peptide binding occurs in the slow exchange regime (with minimal broadening in some cases), which is why peak volumes rather than positions were used for quantiative analysis.

The following pairs of titration pairs were examined to determine K_Ds: CSH2 (-770) (conc. 432 µM) and pY783 peptide (peptide/protein ratio 0 – 2.0; residues analyzed: Glu679, GlyG689, Ser701, Glu720, Ser739); NSH2CSH2 (conc. 200 µM) and pY783 peptide (peptide/protein ratio 0 – 1.25; residues analyzed: His670, Ala703, Cys715, Glu742); CSH2 (-790) (conc. 117 µM) and PDGFR peptide (peptide/protein ratio 0 – 1.2; residues analyzed: Glu679, Gly689, Gly710, Cys715, Gly727, Glu742).

The dissociation constant for binding of a single ligand to a protein is defined as

$$K_D = \frac{([L_0]-[PL])([P_0]-[PL])}{[PL]} = \frac{([L_0]-f[P_0])([P_0]-f[P_0])}{f[P_0]},$$

with $[P_0]$: total concentration of protein; $[L_0]$: total concentration of ligand; [PL]: protein-ligand complex concentration; $f=[PL]/[P_0]$.

This can be rewritten

$$K_D[P_0]f = [L_0][P_0] - f[L_0][P_0] + f^2[P_0]^2 - f[P_0]^2$$

$$f = \left(\frac{[P_0]+[L_0]+K_D}{2[P_0]}\right) + \sqrt{\left(\frac{[P_0]+[L_0]+K_D}{2[P_0]}\right)^2 - \frac{[L_0]}{[P_0]}}$$

Sufficiently separated peaks (see above which residues) in the titration spectra for the bound and the unbound form were selected for fitting. The fraction *f*, representing the ratio of complex to total protein concentration, were obtained from total peak volumes. For $f < 0.5$, *f* was obtained from the intensity of the peaks corresponding to the unbound state. A simultaneous fit to the above equation was performed with a custom Python (2.7) script using the Numpy 1.11.3, Matplotlib 2.0.0 and the Scipy. 0.18.1 libraries (the iPython notebook is provided in *Suppl. Inf. 3.4*). For sampling statistics, 6-8 subsets of data (2-3 data points per peptide concentration) were used for the fitting procedure (jackknife resampling).

S1.10 Small Angle X-Ray Scattering (SAXS)

Small angle X-ray scattering (SAXS) data were recorded at the Deutsche Elektronen-Synchrotron (DESY) facility using synchrotron radiation from the DORIS III electron-positron storage ring. SAXS data have been recorded for NSH2CHS2 and NSH2CSH2-pY783. Note that the construct used for NSH2CSH2 analysis did not contain the Tyr771,775Phe mutations as in other parts in the paper (however, 2D NMR spectra for these two minimally different constructs are almost identical). Both constructs were measured in SAXS buffer (25 mM Tris-Cl, 150 mM NaCl, 10% (v/v) glycerol, 5 mM TCEP, pH 8.0) at the following concentrations: NSHCSH2 - 5.83, 4.02, 2.24 mg/ml; NSH2CHS2-pY^{783}: 5.82, 4.05, 2.16 mg/ml. Scattering data were recorded with a Pilatus detector and an EMBL X33 camera.

The initial SAXS data analysis for calculating the radius of gyration and maximum diameter was performed with PRIMUS (49). Most processing steps were performed using a variety of tools of the ATSAS program package (49, 50), including basic SAXS data processing. Fitting of an ensemble of structures (rigid bodies with flexible linkers) to a SAXS data was performed using the tool EOM (ensemble optimization method).

S2. Supporting Results

S2.1 Relaxation experiments and SAXS experiments showing aggregation

The ^{15}N R_2/R_1 ratios for NSH2CSH2-pY^{783} and NSH2CSH2 (both ≈28.5 kDa) were measured residue-wise. The expected average R_2/R_1 ratio for a spherical protein of 28.5 kDa is estimated as a value around 23.1 (at 600 MHz, estimated from standards measured by Rossi et al.) (51). The measured

average ^{15}N R_2/R_1 ratio at 600 MHz for NSH2CSH2-pY^{783} is 26 at 150 µM, which is close to the value expected for this protein. The average ^{15}N R_2/R_1 ratio at 600 MHz for NSH2CSH2 is 34. Comparing ^{15}N R_2 values of NSH2CSH2 with those of NSH2CSH2-pY^{783} also reveals that NSH2CSH2 has rather high ^{15}N R_2 values, as it is expected for high ^{15}N R_2/R_1 ratios. Dilution of NSH2CSH2 (from 150 µM to 30 µM) returns ^{15}N R_2 values that are much smaller than those recorded for NSH2CSH2 at a higher concentration (150 µM). The reason for the large ^{15}N R_2/R_1 ratios in undiluted samples is probably a propensity of NSH2CH2 to aggregate. Importantly, this aggregation did not lead to any change in chemical shifts.

Small angle X-ray scattering (SAXS) experiments were performed in order to test for aggregation propensity of NSH2CHS2 and NSH2CSH2-pY^{783}. The shape of the SAXS curves, general parameters and distance distribution function match broadly to what would be expected for these two-domain proteins (*Fig. S4*). However, the gyration radius and volume is higher for NSH2CSH2. The large distances for NSH2CSH2 which are found in the distance distribution cannot be explained by the presence of a flexible nSH2-cSH2 junction and a flexible C-terminus alone; we tested this by generating structural ensembles using the Ensemble Optimization Method (50) in which rigid-body structures (individual SH2 domains) are connected by flexible linkers. In addition, we find a concentration dependency of R_g (*Fig. S4a*), revealing that the SAXS data are affected by partial aggregation of this construct.

S2.2 Crystal structure and chemical shift patterns revealing the potential role of interactions between Asn757 and the nSH2-cSH2 junction

According to the crystal structures, the side chain NH_2 group of residue Asn757 at the beginning of the pre-C-terminus could form a hydrogen bond to the backbone carbonyl oxygen of nSH2-cSH2 junction residue Glu667 (distances for Asn-Nδ - Glu-O: 4FBN – 3.4 Å; 4EY0 – 3.9 Å; 3GQI – 3.9 Å), which would offer a potential allosteric connection from Y783 to the nSH2-cSH2 junction that depends on the presence of the C-terminal linker (*Fig. S8b*).

MD simulations predicted an unstable contact between Asn757-Glu759 and the nSH2-cSH2 junction in NSH2CSH2-pY^{783}. The major cross peak for Asn757 in the CSH2 spectrum is located on an *extension* of the vector connecting the corresponding cross peak positions in the spectra of NSH2CSH2 and NSH2CSH2-pY^{783} (*Fig. S7b*), i.e. not *between* these two positions. How might this pattern be rationalized within the context of the exchange phenomenon invoked above? One means is as follows. For NSH2CSH2-pY^{783} it might be expected that for most residues p^i(closed) ~ 1. Within the model of chemical exchange described above the Asn757 peak position for CSH2 suggests $p^{N757}{}_{closed} > 1$, which is clearly not reasonable. Rather, the cross peak position in NSH2CSH2-pY^{783} may reflect balance

between 'closed' and (a - naively - unexpected non-zero population of) 'open' states specifically at Asn757 (different to the situation at Tyr783), and that p^{N757}_{closed}(NSH2CSH2-pY^{783}) < p^{N757}_{closed}(CSH2). In this scenario the presence of the nSH2 domain in the NSH2CSH2-pY^{783} protein has the effect of 'loosening' the interaction between the cSH2 domain and the pre-C-terminus, if we assume that this is the structural equivalent of a "more open" conformation. This scenario is equivalent to inferring that even in the case of NSH2CSH2-pY^{783} the pre-C-terminus is not fully bound; this has been predicted by MD simulations (*Fig. 2d*). It can be inferred that residue Asn757 in NSH2CSH2-pY^{783} is not always in the 'closed' state, possibly due to the nSH2 domain weakening the interaction between the cSH2 domain and the pre-C-terminus via contacts between the nSH2-cSH2 junction and the pre-C-terminus.

Taking the likely structural contact between the nSH2-cSH2 junction and the Asn757 domain into account, it appears likely that the nSH2 exercises the loosening influence on the pre-C-terminus / cSH2 interaction via the nSH2-cSH2 junction.

S2.3 Combined slow and fast exchange kinetics can explain peak tripling for residue Thr766

Residue Thr766 in the pre-C-terminus provides another example of a residue exhibiting both slow and fast exchange (*Fig. S12b)*. In this case, three cross peaks are detected, one of which is at the same position as that detected for NSH2CSH2$^{\Delta CT}$. Of the other two, one appears significantly broadened. It is a challenge to unambiguously decipher the underlying mechanism that gives rise to this pattern. However, the following argument can be posited. The first cross peak can be thought of a corresponding to a state O_3 wherein the C-terminal tail is locally 'open'. The second cross peak represents a state which is exchanging rapidly between 'closed' and 'open' forms $\{C \rightleftharpoons O\}_2$ with an equilibrium partitioning given by $K^i_2 = p^i_{2,C}/p^i_{2,O}$. The third cross peak represents another state which exchanges $\{C \rightleftharpoons O\}_1$ at a different rate and is partitioned according to $K^i_1 = p^i_{1,C}/p^i_{1,O}$. The *local* structures O_1, O_2 and O_3 and likewise the *local* structures C_1 and C_2 can reasonably assumed to be identical because the cross peaks for O_3, $\{O/C\}_2$ and $\{O/C\}_1$ are all located on the same vector connecting the cross peaks for $O_1/O_2/O_3$ and C_1/C_2. However, what gives rise to the presence of multiple cross peaks is a slow conformational process that is *remote* but nevertheless 'sensed' at Thr766 by virtue of a shift in the extent of 'closed' character at that position in the different states. A general kinetic scheme for this exchange situation would read $\{O \rightleftharpoons^* C\}_1 \rightleftharpoons^\dagger \{O \rightleftharpoons^* C\}_2 \rightleftharpoons^\dagger O_3$, where * and † denote fast and slow exchange equilibria, respectively. Sample schemes illustrating possible more specific underlying kinetic schemes are illustrated in *Fig. S12d*.

S2.4 Proline *cis-trans* peptide bond isomerization could be the source of slow exchange

One can speculate that in the context of the NSH2CSH2 protein, the source(s) of slow processes could be *cis-trans* peptide bond isomerization in the C-terminal region at Glu768-Pro769, Asn779-Pro780, Asn787-Pro7888 and/or Met789-Pro790. In line with wide experience of unstructured peptides, such isomerization would be on a slow timescale, and is highly likely in the relatively unconstrained Tyr783-unbound state of the C-terminal region. It is entirely plausible that the *cis-/trans-* state of one or more of these peptide bonds could influence the effective affinity of the Tyr783 region for the cSH2 domain, and thereby give rise to the observed NMR characteristics. We have performed initial experiments comparing the WT CSH2, Pro686Ala, Pro745Ala, Pro755Ala and Pro769Ala mutant constructs (Fig. S*12a*). Chemical shift analysis suggests that these mutations can dramatically shift the exchange behavior of multiple residues. For several residues, the Pro686Ala and Pro769Ala substitutions tilt the dynamic equilibrium towards the 'open' state; note that Pro686 and Pro759 are spatially close to each other, and that Pro686 neighbors the site of the disease-relevant residue Arg687. Comparing the cross peak patterns for WT and Pro745Ala proteins suggests that the 'open' population(s) might often be lower in the latter case. Overall these observations are strongly suggestive that *cis-trans* peptide bond isomerization in the extended C-terminus is contributing to the complexity in the spectra of the cSH2 domain-containing proteins. Moreover, that complexity serves to reinforce the conclusion that the various residues within the C-terminal linker are in dynamic binding equilibria with the cSH2 domain surface.

S2.5 Structural interactions between the cSH2 α_B helix and the nSH2-cSH2 junction

The structural and dynamic interactions between the cSH2 α_B helix (including surrounding residues) and the nSH2-cSH2 junction are of interest because of the impact of allosteric communication triggered by Tyr783 phosphorylation. In order to predict the effect of Tyr783 phosphorylation on the nSH2-cSH2 junction, the potential for interaction between the junction and nearby residues, specifically those in cSH2 helix α_B, were identified from MD simulation trajectories (*Fig. S8a*). Transient interactions between nSH2-cSH2 junction residue Trp668 and α_B residue Ile738, as well as between junction residues Thr660-His663 and α_B residue Ser733, are much more pronounced in the NSH2CSH2 trajectories than for NSH2CSH2-pY^{783}, suggesting that Tyr783 phosphorylation leads to a reduction of some contacts between the nSH2-cSH2 junction and the α_B helix; other contact analysis focusing on the α_B helix (with surrounding residues, Ser732-Glu742) and on nSH2-cSH2 junction

sections (Thr660-His663 or Glu664-Lys666) show a slightly stronger interaction in the phosphorylated state.

In agreement with the MD simulation, Ile738 chemical shifts are sensitive to structural changes in the nSH2-cSH2 junction (*Fig. S8d*). Similar perturbations were observed for neighboring residue Leu737 (*Fig. S8d*). A particularly large perturbation in the CSH2 spectrum indicates the potential for a direct contact between Ile738 and the nSH2-cSH2 junction that is disrupted upon removal of the nSH2 domain (*Fig. S8b*).

Chemical shift perturbation experiments also reveal that a large impact of Tyr783 phosphorylation on the cross peak position is evident for residue Glu742; a plot of the peak position for various constructs indicates that they can be interpreted within a framework of fast exchange (*Fig 5a*). Inspection of the cross peaks for NSH2CSH2, NSH2CSH2$^{\Delta CT}$, NSH2CSH2-pY^{783}, E664G-NSH2CSH2, E664K-NSH2CSH2 and CSH2 reveals an equivalence with the peak patterns observed in context of the dynamic allosteric C-linker dependent pathway. However, comparison of NSH2CSH2-pY^{783} and NSH2CSH2$^{\Delta CT}$-CTpY peak positions relative to NSH2CSH2$^{\Delta CT}$ indicate that the presence and absence of the C-terminal linker have opposing effects for Glu742 in the two phospho-constructs. First, it is indicated that even without C-terminal linker presence, pY^{783} phosphorylation can be communicated to this residue. Secondly, the assumption that NSH2CSH2$^{\Delta CT}$ is wholly 'open' ($p_C = 0$) does not hold because the NSH2CSH2$^{\Delta CT}$-CTpY cross peak is not located between the corresponding NSH2CSH2$^{\Delta CT}$ and NSH2CSH2-pY^{783} peaks but rather on an extension of a vector pointing from NSH2CSH2-pY^{783} to NSH2CSH2$^{\Delta CT}$. Thus, it is not certain to what extent the two states underlying the Glu742 peak positions can be identified as strictly 'open' or 'closed'. C-terminal linker-dependent and -*in*dependent binding have an opposing effect on the Glu742 chemical shift, suggesting that the nearby nSH2-cSH2 junction structure might be modulated in a different manner in each case.

S2.6 C-terminal linker-*in*dependent allostery

In agreement with the location of the putative pY783 peptide binding site, which has been described elsewhere (10, 11), large CSPs are observed between NSH2CSH2$^{\Delta CT}$-CTpY and NSH2CSH2$^{\Delta CT}$ for multiple β-sheet residues: Peak positions for β-sheet residues Arg694, Lys695, Ala703, Ile704, Ser705 and Glu720 are essentially identical for NSH2CSH2-pY^{783} and NSH2CSH2$^{\Delta CT}$-CTpY (*Fig. S7d*), indicating that these residues are mostly affected by C-terminal linker-*in*dependent effects of pY783 peptide binding. The nSH2-cSH2 junction and α_B helix (with surrounding residues) are not part of the putative pY783 peptide binding site. However, cross peak position differences between NSH2CSH2$^{\Delta CT}$ and NSH2CSH2$^{\Delta CT}$-CTpY observed for junction residue Thr660 and residue Ala732

(close to the α_B helix, *Fig 4a*, *Fig. S8c*) indeed reveal a significant C-terminal linker-*in*dependent allosteric connection to the nSH2-cSH2 junction.

S2.7 Peptide titration experiments for K_D determination

For the peptide titration method and data processing see *Suppl. Mat. S1.9*, All peptide titrations reveal strong binding of the peptides to the cSH2 domain. The K_D for PDGFRβ peptide is estimated to be ≤ 1.1 μM. Due to peak broadening, overlap and exchange phenomena, a precise estimation of the K_D of binding pY^{783} peptide to the cSH2 domain is challenging. The K_D for the system NSH2CSH2 / pY^{783} is 3.1±1.1 μM and for $CSH2^{\Delta CT}$ / pY^{783} 14.6±1.1 μM. Titration plots, with all data points and overall fit, are shown in *Fig. S4*. At much higher pY^{783} peptide/protein ratio (> 1.5), secondary peptide binding to the nSH2 domain occurs. From these results, and from qualitative inspection of $NSH2CSH2^{\Delta CT}$/pY^{783} peptide (1.2 equivalents) spectra, we can confirm that in all NMR experiments of this paper, PDGFR and pY^{783} peptide is fully bound to cSH2 and in slow exchange.

S2.8 Molecular dynamics simulations of Arg687Trp-mutant constructs

Three 100 ns simulations for each R687W-NSH2CSH2 and R687W-NSH2CSH2-pY783 were performed for comparison with WT constructs. The predictive value of these simulations is limited as they were not started from crystal structure, but rather from Arg687Trp *in silico* mutated constructs.

Principal component analysis was extended to Arg687Trp constructs. The projections of each trajectory on the PCA eigenvectors revealed a difference between R687W-NSH2CSH2 and NSH2CSH2 trajectories in NSH2CSH2 eigenvector 2 (*Fig. S1a*); the same eigenvector has been found to be relevant to describe differences between NSH2CSH2 and NSH2CSH2-pY783. In addition, R687W-NSH2-CSH2 eigenvector 3 and R687W-NSH2CSH2-pY783 eigenvector 2 reveal differences between Arg687Trp and WT construct (*Fig. S1c-d*). A high root-mean-square fluctuation (RMSF) of the C-terminal linker and the pre-C-terminus in these eigenvectors *(Fig. S10b-c)* suggests that the dynamics of these regions differs between Arg687Trp and WT.

Comparison of mutual information analysis results for R687W-NSH2CHS2 trajectories with NSH2CSH2 trajectory results (*Fig. S10a*) reveal that the Arg687Trp mutation leads to a general reduction of mutual information between C-terminal residues and a great number of cSH2 residues, including the nSH2-cSH2 junction, and some residues in the nSH2 domain that are spatially close to the nSH2-cSH2 junction. These data suggest that the Arg687Trp disrupts the allosteric network which we have found in the WT construct.

The results of contact probability analysis for R687W-NSH2CSH2 and NSH2CSH2 molecular dynamics trajectories are ambiguous for contacts between pre-C-terminal and C-terminal residues and the cSH2 domain: contact events between C-terminal linker and the α_A helix are more likely in R687W-NSH2CSH2, while many other contacts are less probable (*Fig. S9a*); however, additional simulation material for R687W-NSH2CSH2would be required to yield more precise predictions. Comparing R687W-NSH2CH2 with R687W-NSH2CSH2-pY^{783} suggests that allosteric communication might be disrupted in the mutant: most contact probability changes for contacts with the nSH2-cSH2 junction (related to Tyr783 phosphorylation) that were observed in the respective WT constructs could be observed in the presence of the Arg687Trp substitution (*Fig. S9c*), with the exception of a higher probability of contacts between (a) Tyr783 and cSH2 and (b) Thr660-His663 and Asp732-Glu742 (α_B helix and surrounding residues; *Fig. S9d*). For additional information about contacts between the α_B helix and the nSH2-cSH2 junction, see also *Suppl. Inf. 2.5*.

In summary, MD simulations suggest that the Arg687Trp mutation reduces allosteric communication across the cSH2 domain. This disruption of allostery appears to be linked in a change of the structure or dynamics of the C-terminus.

S2.9 PDGFRβ peptide titration to explore combined, pre-formed slow/fast exchange equulibria in CSH2

The PDGFRβ peptide was employed previously in context of a study of PLCγ1 and is known to bind to CSH2 (10, 52). We found strong binding ($K_D \leq 1.1$ μM) in a titration experiment (*Suppl. Inf. 2.7, Fig. S6*). In the titration, the PDGFRβ peptide shifts the slow exchanging populations of residues sensitive to the attached C-terminal linker, towards the 'most open' state exemplified by Gly710 in *Fig. S15a* and Gly689, Gly727, Glu742, Gly765 and Gly777 in *Fig. S15c*). Remarkably, in this experiment more than two slowly exchanging states are evident. The Gly710 site in the cSH2 domain αA-β1 loop is remote from the PDGFRβ peptide binding site. Specifically, for this residue we find that the population of a partially 'closed' state $\{O \rightleftharpoons C\}_1$ falls upon addition of the phosphopeptide (*Fig. 15b*). As more peptide is added, the cross peak intensity is then successively distributed between a number of resonances that are located on the vector connecting $\{O/C\}_2$, $\{O/C\}_3$ and the complex between the cSH2 domain and the PDGFRβ peptide, here denoted O:P. Namely, the observed titration behaviour can be understood by invoking an extension of the combined fast/slow exchange scheme:

$(\{O \rightleftharpoons^{*} C\}_1 \rightleftharpoons^{\dagger} \{O \rightleftharpoons^{*} C\}_2 \rightleftharpoons^{\dagger} \{O \rightleftharpoons^{*} C\}_3 \rightleftharpoons^{\dagger} O{:}P$

Close inspection of the CSH2 peak pattern suggests that the set of $\{O/C\}_i$ states pre-exist in WT CSH2; the population shifts occur due to peptide association at the (remote) binding site, leading to local

changes of the dynamic equilibrium between states where the C-terminal linker is associated, or not, with the surface of the cSH2 domain.

S3 Bash and Python Scripts

S3.1 MD data processing - contact event detection in Gromacs trajectories using bash scripts and g_mindist

main bash script for contact analysis

```
#!/bin/bash
for x in $(echo $1) #need $1.gro and $1.xtc in this folder.
do
printf "" > "$x"summat.dat
printf "" > "$x"freqmat.dat
y=$x
for z in $(echo "650-668 670-750 675-684 650-659 660-668 660-663 664-666 667 668 680-690 687");
do
printf "$x"'\n'"$y"'\n'"$z"'\nq\n757-790\n757-770\n771-778\n779-790\n757-759\n760-766\n767-770\n783\n732-742\n733\n738\nq\n' | ./mindistcalc.sh
sleep 0.01
echo "header" > "$1"res.csv; cat "$1"_n.csv | awk '{printf "xx "}{for (i=1; i<=NF/2; i++){printf "%i ", $(2*i)}}{printf "\n"}' >> "$1"res.csv
cp "$x"res.csv ./"$x"_"$z"res.csv
echo $(cat "$x"res.csv | sed "2,$ ! d" | awk '{for (i=2; i<=NF; i++) {if ($i>0) printf "%s ", 1; else printf "%s ", 0} {printf "\n"}}' | awk 'BEGIN{for (i=1; i<=NF; i++) {x[i]=0;z=0}}{z+=1;for (i=1; i<=NF; i++) {x[i]+=$i}}END{for (i=1;i<=NF;i++){printf "%i ", 1000000*x[i]/(z)}}') >> "$x"summat.dat
echo $(cat "$x"res.csv | sed "2,$ ! d" | awk '{for (i=2; i<=NF; i++) {if ($i>0) printf "%s ", 1; else printf "%s ", 0} {printf "\n"}}' | awk 'BEGIN{for (i=1; i<=NF; i++) {x[i]=0; y[i]=$i; z[i]=0}}{z+=1;for (i=1; i<=NF; i++) {if (NR==1) {y[i]=$i}else {if (y[i]==0 && $i ==1) {x[i]+=1};if (y[i]==1 && $i ==0) {x[i]+=1};y[i]=$i}}}END{for (i=1;i<=NF;i++){printf "%i ", 1000000*x[i]/(2*z)}}') >> "$x"freqmat.dat
sleep 0.01
done
done
```

bash script - mindistcalc.sh, called by the main bash script for contact analysis

```
#!/bin/bash
echo -e "Please type name of trajectory to process (no xtc at the end)"
read trjfile
echo -e "Please type name of mass file to take into account (no gro at the end)"
read massfile
declare -a index1list
declare -a index2list
count=0
while [ "$index1" != "q" ]
do
echo -e "Please put (mext) index1 variable (format i.e. 545 or 545-559), press q when finished"
read index1
index1list[(($count))]="$index1"
count=$(( $count + 1 ))
done
count2=0
while [ "$index2" != "q" ]
do
echo -e "Please put (mext) index2 variable, press q when finished"
read index2
index2list[(($count2))]="$index2"
count2=$(( $count2 + 1 ))
done
count3=0
firstline="time "
space=" "
while [ $count3 != $(($(($count)) - 1)) ]
do
count4=0
while [ $count4 != $(($(($count2)) - 1)) ]
do
rm tempndx.ndx
printf "r${index1list[$(($count3))]}\nr${index2list[$(($count4))]}\n\nq'" | make_ndx -f "$massfile".gro -o tempndx.ndx
printf "10\n11\n" | g_mindist -f "$trjfile".xtc -group -s "$massfile".gro -od tempd.xvg -or tempr.xvg -on tempn.xvg -xvg none -n tempndx.ndx -d 0.25
echo "$firstline""$space"r${index1list[$(($count3))]}_r${index2list[$(($count4))]}
firstline="$firstline""$space"r${index1list[$(($count3))]}_r${index2list[$(($count4))]}
        if [ $count3 == 0 ] && [ $count4 == 0 ]
        then
```

```
        mv tempr.xvg "$massfile"_r.csv
        mv tempd.xvg "$massfile"_d.csv
        mv tempn.xvg "$massfile"_n.csv
        else
        paste -d \  "$massfile"_r.csv tempr.xvg > "$massfile"t_r.csv
        paste -d \  "$massfile"_d.csv tempd.xvg > "$massfile"t_d.csv
        paste -d \  "$massfile"_n.csv tempn.xvg > "$massfile"t_n.csv
         rm temp*
        mv "$massfile"t_r.csv "$massfile"_r.csv
        mv "$massfile"t_d.csv "$massfile"_d.csv
        mv "$massfile"t_n.csv "$massfile"_n.csv
        fi
count4=$(($count4 + 1))
done
count3=$(($count3 + 1))
done
rm temp*
rm \#*
echo $firstline
echo $firstline > header.txt
```

S3.2 MD data processing – principal component analysis with post-processing (bash script)

```
#!/bin/bash
datasets='nph ph 687nph 687ph' #list of trajectory sets. Needs to have relevant .gro and .xtc files in the ../(dataset)/
path.
stres=658 #residue from which the PCA starts. set to 545 for the entire tandem construct.

###### preparation and fusion of trajectories. #######
mkdir fusetraj
cd fusetraj
for a in $datasets
do
n=0
#in this section, it is defined how trajectories will be merged for PCA. For example, in this example, the 'nph'
construct
#includes 6 trajectories of 50000 us length (because only 50-100 ns will be used).
if [[ $a == 'nph' ]]
then
trajst='10\n50010\n100010\n150010\n200010\n250010\n'
trajlst='tx_1.xtc tx_2.xtc tx_3.xtc tx_4.xtc tx_5.xtc tx_6.xtc'
elif [[ $a == 'ph' ]]
then
trajst='10\n50010\n100010\n150010\n'
trajlst='tx_1.xtc tx_2.xtc tx_3.xtc tx_4.xtc'
else
trajst='10\n50010\n100010\n'
trajlst='tx_1.xtc tx_2.xtc tx_3.xtc'
fi

##this aligns trajectories, ensures that they are whole, extracts backbone atoms of the desired segment, and fuses
trajectories.
for b in $(ls ../../"$a"/*.gro | sed "s/.gro//g")
do
echo $b
n=$((n+1))
cp "$b".gro ./"$a"_"$n".gro
printf '4&r'"$stres""-790\nq\n" | make_ndx -f "$a"_"$n".gro -o "$a"_"$n".ndx
printf "10\n" | trjconv -n "$a"_"$n".ndx -s "$a"_"$n".gro -f "$a"_"$n".gro -o "$a"_"$n"_cSH2.gro
printf "r670-750\nq\n" | make_ndx -f "$a"_"$n"_cSH2.gro -o "$a"_"$n"_cSH2.ndx
printf "1\n0\n" | trjconv -s "$a"_"$n"_cSH2.gro -f "$a"_"$n"_cSH2.gro -o "$a"_"$n"_cSH2b.gro -box 200 200 200 -center
echo $b
printf "10\n" | trjconv -n "$a"_"$n".ndx -s "$a"_"$n"_cSH2.gro -f $b.xtc -b 50010 -o ta_"$n".xtc

printf "10\n1\n" | trjconv -s "$a"_"$n"_cSH2.gro -f ta_"$n".xtc -fit progressive -o tb_"$n".xtc -n "$a"_"$n"_cSH2.ndx
printf "1\n" | trjconv -s "$a"_"$n"_cSH2.gro -f tb_"$n".xtc -o tc_"$n".xtc -b 50010 -e 100000 -n "$a"_"$n"_cSH2.ndx
printf "1\n0\n" | trjconv -s "$a"_"$n"_cSH2.gro -f tc_"$n".xtc -o tx_"$n".xtc -box 200 200 200 -center
done

printf $trajst | trjcat -f $trajlst -o tx_comb.xtc -cat -settime
printf "1\n" | trjconv -s "$a"_"$n"_cSH2b.gro -f tx_comb.xtc -pbc nojump -o ty_comb.xtc
printf "1\n1\n" | trjconv -s "$a"_"$n"_cSH2b.gro -f ty_comb.xtc -fit progressive -o "$a"_cSH2.xtc
cp "$a"_"$n"_cSH2b.gro ./"$a"_cSH2.gro
echo 'done'
done
cd ..


############ This is the actual principal component analysis, based on the fused trajectories generated
beforehand.#####
mkdir pca_docsh2
cd pca_docsh2

for x in $datasets
do
mkdir $x
cp ../fusetraj/"$x"_cSH2.xtc ./$x/"$x"_cSH2.xtc
cp ../fusetraj/"$x"_cSH2.gro ./$x/"$x"_cSH2.gro
done
cwd=$(pwd)
```

```
for x in $datasets
do
cd $x
printf "1\n1\n" | g_covar -s "$x"_cSH2.gro -f "$x"_cSH2.xtc -av aver_"$x".pdb -o eigenval_"$x".xvg -l covar_"$x".log
-v eigenvec_"$x".trr
cd $cwd
done

cwd=$(pwd)

############## All trejectories are now projected on all eigenvectors, in two rounds:
#first, projections on "self" (for example nph on nph), then all others.
for initrun in $(seq 0 1 1)
do
for z in $datasets
do
if [[ $z == 'nph' ]]
then
maxnum=25001
elif [[ $z == 'ph' ]]
then
maxnum=15001
else
maxnum=10000
fi
echo 'maxnum' $maxnum
if [[ $initrun -eq 0 ]]
then
mkdir pca_actual_$z
fi
cd pca_actual_$z
cp ../$z/"$z"_cSH2.xtc jointtrj_$z.xtc
for x in $datasets;
do
if [[ ($initrun -eq 0 && $z == $x) || ($initrun -eq 1 && $z != $x) ]]
then
echo 'select ' $initrun $z $x
printf "" > proj_analysis_"$z"_"$x".txt
cp ../$x/aver_"$x".pdb aver1_$x.pdb
cp ../$x/eigenval_"$x".xvg eigenval_$x.xvg
cp ../$x/eigenvec_"$x".trr eigenvec_$x.trr
cp ../$x/covar_"$x".log covar_$x.log


for y in $(seq 1 1 10)
do
#do the projections on the joint trajectories
printf "1\n1\n" | g_anaeig -s aver1_"$x".pdb -f jointtrj_"$z".xtc -eig eigenval_"$x".xvg -v eigenvec_"$x".trr -extr
extreme_"$x"_$y.pdb -first $y -last $y
printf "0\n0\n" | g_anaeig -s aver1_$x.pdb -f jointtrj_$z.xtc -eig eigenval_$x.xvg -v eigenvec_$x.trr -proj
proj_"$x"_$y.xvg -split -first $y -last $y

#the results are split to obtain a result for each trajectory. For each trajectory, minimum, maximum and average
projection value are determined. For these three results, an average and standard deviation is calculated and saved
in a results table.
cp proj_"$x"_$y.xvg tempdata.tmp
printf "" > dataout.tmp
grep -v @ tempdata.tmp | grep -v \& | tac > dataout.tmp
for q in $(seq 1 5000 $maxnum); do p=$(($q+5000)); sed "$q"','"$p"" ! d" dataout.tmp | awk
'BEGIN{a=0;b=100000000;c=0;d=0}{a+=1;d+=$2;if($2<b){b=$2};if($2>c){c=$2}}END{printf "%.4f %4f %4f\n", b, c, d/a}';
done > test.log
cp test.log test_"$x"_"$z".log
echo $z $x $y $(awk -v e=$(awk 'BEGIN{a=0;b=0;c=0;d=0}{a+=1;b+=$1}END{printf "%.6f\n", b/a}' test.log) -v f=$(awk
'BEGIN{a=0;b=0;c=0;d=0}{a+=1;b+=$2}END{printf "%.6f\n", b/a}' test.log) -v g=$(awk
'BEGIN{a=0;b=0;c=0;d=0}{a+=1;b+=$3}END{printf "%.6f\n", b/a}' test.log) 'BEGIN{a=0;b=0;c=0;d0}{a+=1;b+=($1-
e)**2;c+=($2-f)**2;d+=($3-g)**2}END{printf "%.5f %.5f %.5f %.5f %.5f %.5f\n", e, sqrt(b/a),f,sqrt(c/a),g,sqrt(d/a)}'
test.log) >> proj_analysis_"$z"_"$x".txt

done

fi
done

cd $cwd
done
done

############# collection of data and output preparation ########
cp -rp ../cSH2full ./
mkdir colldat
cd colldat
for t in $datasets
do
s='../cSH2full/'"$t"'_cSH2_x'
r='../'"$t"'/'"$t"
printf "r""$stres""-790\nq\n" | make_ndx -f "$s".gro -o "$r"_CSH2x.ndx
printf "10\n" | trjconv -f "$s".gro -s "$s".gro -n "$r"_CSH2x.ndx -o "$r"_CSH2x.gro
printf "1\n" | trjconv -f "$r"_CSH2x.gro -s "$r"_CSH2x.gro -o "$r"_CSH2x.pdb

for v in $(seq 1 1 10);
do
```

```
## the extreme structures for each eigenvector have been calculated. For better illustration, the RMSD (revealing the regions which are most different between the extreme structures) is
#determined. A structure with sidechains (for illustrative purposes) is used to write the RMSD into the B factor column.
printf "1\n" | g_rmsf -s "$r"_cSH2.gro -f ../pca_actual_$t/extreme_"$t"_"$v".pdb -oq ../$t/temp.pdb -res; sed "s/ 1.00/  1.00 /g" ../$t/temp.pdb > "$r"_cSH2_ex_"$v".pdb
for a in $(seq $stres 1 790);
do
grep ^"ATOM" "$r"_CSH2x.pdb | grep '   '"$a"'   ' | sed 's/1.00  0.00/1.00  '"$(grep ^"ATOM" "$r"_cSH2_ex_"$v".pdb | grep '   '"$a"'   ' | sed "1 ! d" | awk '{printf "%.2f\n", $10}')"'/g'
done > "$t"_ex_"$v".pdb
done
done
cwd=$(pwd)
for initrun in $(seq 0 1 1)
do
for y in $datasets
do
mkdir $y
for x in $datasets
do
if [[ ($initrun -eq 0 && $y == $x) || ($initrun -eq 1 && $x != $y) ]]
then
cp ../pca_actual_"$x"/proj_analysis_"$x"_"$y".txt ./$y/

cd ./$y
c=$y
d=$x
## This routine filters the projection analysis results to reveal where projections are "significantly" different. Significance not in the statistical sense.
if [[ $x != $y ]]
then
a=proj_analysis_"$c"_"$c".txt; b=proj_analysis_"$d"_"$c".txt; for x in $(seq 1 1 $(cat $a | wc | awk '{print $1}')); do h=$(sed "$x ! d" $a | awk '{print $4}'); i=$(sed "$x ! d" $a | awk '{print $5}'); j=$(sed "$x ! d" $a | awk '{print $6}'); k=$(sed "$x ! d" $a | awk '{print $7}'); l=$(sed "$x ! d" $a | awk '{print $8}'); m=$(sed "$x ! d" $a | awk '{print $9}'); cat $b | sed "$x ! d" | awk -v h=$h -v i=$i -v j=$j -v k=$k -v l=$l -v m=$m '{n=0; o=0; p=0; q=0; r=0; s=0; if ($4+$5<h-i){n=$4; o=$5}; if ($4-$5>h+i){n=$4; o=$5}; if ($6+$7<j-k){p=$6; q=$7}; if ($6-$7>j+k){p=$6; q=$7}; if ($8+$9<l-m){r=$8; s=$9}; if ($8-$9>l+m){r=$8; s=$9}; printf "%i %.5f %.5f %.5f %.5f %.5f %.5f\n", $3, n, o,p,q,r,s}'; done > TRAJ_"$d"_on_EVset_"$c"_RES"$d"; b=proj_analysis_"$c"_"$c".txt; a=proj_analysis_"$d"_"$c".txt; for x in $(seq 1 1 $(cat $a | wc | awk '{print $1}')); do h=$(sed "$x ! d" $a | awk '{print $4}'); i=$(sed "$x ! d" $a | awk '{print $5}'); j=$(sed "$x ! d" $a | awk '{print $6}'); k=$(sed "$x ! d" $a | awk '{print $7}'); l=$(sed "$x ! d" $a | awk '{print $8}'); m=$(sed "$x ! d" $a | awk '{print $9}'); cat $b | sed "$x ! d" | awk -v h=$h -v i=$i -v j=$j -v k=$k -v l=$l -v m=$m '{n=0; o=0; p=0; q=0; r=0; s=0; if ($4+$5<h-i){n=$4; o=$5}; if ($4-$5>h+i){n=$4; o=$5}; if ($6+$7<j-k){p=$6; q=$7}; if ($6-$7>j+k){p=$6; q=$7}; if ($8+$9<l-m){r=$8; s=$9}; if ($8-$9>l+m){r=$8; s=$9}; printf "%i %.5f %.5f %.5f %.5f %.5f %.5f\n", $3, n, o,p,q,r,s}'; done > TRAJ_"$d"_on_EVset_"$c"_RES"$c"
fi
rm \#*
rm temp*
rm \#*
cd $cwd
rm \#*
fi
done
cp ./"$y"_ex* ./$y/
done
done
```

S3.3 MD data processing – MutInf post-processing (Python 2.7 script)

```
import csv
import numpy as np
import matplotlib as mpl
import matplotlib.pyplot as plt
import os
from IPython.core.interactiveshell import InteractiveShell
from IPython.core.display import display,HTML
InteractiveShell.ast_node_interactivity = "all"
from scipy import stats
from os import listdir

def getdatalist(path2):
    global fnames,path
    path="/media/hanskoss/data/PLCg1_PhD/NIMR_evacuate/research3/hkoss/md_tandem/new_reanalysis/mutinf/proc/"+path2
    fnames=os.listdir( path )

def prepselection(selection,mina,maxa,minb,maxb):
    for a in selection:
        global abc
        global datlist
        global listx
        with open(path+'/'+fnames[a],'rb') as csvfile:
            datax = csv.reader(csvfile, delimiter=' ')
            i=0
            val = []
            for row in datax:
                val.append(row)
                i+=1
        valnp=np.array(val)
        abc=np.shape(valnp)[0]
        listx=[]
        header=valnp[0][0:abc-1]
        for x in np.arange(1,abc,1):
            listx.append(valnp[x][1:abc])
        datlist=np.array(listx).astype('float64')
        if abc - 1 == maxresxx-minresxx+2:
            newdat=np.zeros([np.shape(datlist)[0]-1,np.shape(datlist)[1]-1])
            delres=783
            newdat[0:delres-minresxx,0:delres-minresxx]=datlist[0:delres-minresxx,0:delres-minresxx]
            newdat[delres-minresxx:maxresxx-minresxx+1,delres-minresxx:maxresxx-minresxx+1]=datlist[delres+1-
minresxx:maxresxx-minresxx+2,delres+1-minresxx:maxresxx-minresxx+2]
            newdat[0:delres-minresxx,delres-minresxx:maxresxx-minresxx+1]=datlist[0:delres-minresxx,delres+1-
minresxx:maxresxx-minresxx+2]
            newdat[delres-minresxx:maxresxx-minresxx+1,0:delres-minresxx]=datlist[delres+1-minresxx:maxresxx-
minresxx+2,0:delres-minresxx]
            datlist=newdat
            abc=np.shape(valnp)[0]-1
        mpl.rcParams['figure.figsize'] = (10,10)

def filterdiag(t,omitdiag):
        u=np.zeros(np.shape(t))
        for x in np.arange(0,np.shape(t)[0]):
            for y in np.arange(np.shape(t)[0]):
                if not x-omitdiag < y or not y < x+omitdiag:
                    u[x,y]=t[x,y]
        return u

def filterhalfdiag(t,whichhalf): #1 upper half
        u=np.zeros(np.shape(t))
        for x in np.arange(0,np.shape(t)[0]):
            for y in np.arange(0,np.shape(t)[0]):
                if whichhalf == 1:
                    if y<=x:
                        u[x,y]=t[x,y]
                else:
                    if y>=x:
                        u[x,y]=t[x,y]
        return u

def plotstuff(mina,maxa,minb,maxb,filenam):
    if abc == maxresxx-minresxx+2:
        datlist0=filterdiag(datlist,5)[::-1]
        b3=mina-minresxx
        b4=maxa-mina+1+b3
        b2=maxresxx-(minb-minresxx)-minresxx+1
        b1=(maxresxx-maxb)+minresxx-minresxx
        a=datlist0[b1:b2,b3:b4]
        totmax=np.max(np.abs(a))
        print "maximum abs value of this plot is " + str(totmax)
        plt.rcParams['figure.facecolor']='white'
        totmax=5.12 #this sets a fixed max/min value for the plot.Comment out when needed.
        plt.imshow(a,   cmap='seismic',   clim=(-totmax,totmax),interpolation='none',extent=[mina-0.5,maxa+0.5,minb-
0.5,maxb+0.5])
        #seismicbwr
        plt.colorbar()
        plt.savefig('/home/hanskoss/mutinfres/'+filenam)
        plt.show()
```

```
def getregionsums(whatever,r1,r2,r3,r4,filtersize):
    xq=filterdiag(whatever,filtersize)[r1-minresxx:r2-minresxx+1,r3-minresxx:r4-minresxx+1]
    #print xq
    x=np.arange(r1,r2+1,1)
    y=np.sum(xq,1)
    return [list(x),list(y)]

def plotsome(whatever,r1,r2,r3,r4,filtersize):

    [x,y]=getregionsums(whatever,r1,r2,r3,r4,filtersize)
    plt.plot(x,y)
    plt.show()
    stuff=[x,y]
    match=np.array(stuff)[0,list(np.argsort(stuff,1)[1])]*(np.array(stuff)[1,list(np.argsort(stuff,1)[1])]>0)
    matchlist=[]
    for x in match[match > 0]: matchlist.append(int(x))
    print np.array(matchlist)
    print np.sort(matchlist)

def preparedata(setname,mina,maxa,minb,maxb,maxcol):
    getdatalist(setname)
    alldata=[]
    for x in np.arange(1,maxcol,1):
        prepselection([x],mina,maxa,minb,maxb)
        alldata.append(datlist)
    return [np.average(alldata,axis=0), np.std(alldata,axis=0)]

def ttestdo(datasetaver1,datasetstd1,statn1,datasetaver2,datasetstd2,statn2,cutoffstat):

[statval,pval]=stats.ttest_ind_from_stats(datasetaver1,datasetstd1*np.sqrt(statn1),statn1,datasetaver2,datasetstd2*
np.sqrt(statn2),statn2,equal_var=False)
    return np.where(~np.isnan(pval),pval,1)<cutoffstat
print 'finished loading definitions'
print 'loading data and calculating...'
minresxx=545; maxresxx=789
mina=minresxx; maxa=maxresxx; minb=minresxx; maxb=maxresxx
[phdat,phdats]=preparedata("phrndcoll",mina,maxa,minb,maxb,10)
[nphdat,nphdats]=preparedata("nphrndcoll",mina,maxa,minb,maxb,10)
[nph687dat,nph687dats]=preparedata("nph687rndcoll",mina,maxa,minb,maxb,10)
[ph687dat,ph687dats]=preparedata("ph687rndcoll",mina,maxa,minb,maxb,10)
nphupdownstat=ttestdo(nphdat,nphdats,10,phdat,phdats,10,0.05)
nph687updownstat=ttestdo(nphdat,nphdats,10,nph687dat,nph687dats,10,0.05)
ph687updownstat=ttestdo(phdat,phdats,10,ph687dat,ph687dats,10,0.05)
datlist=filterhalfdiag(nphdat,1)+filterhalfdiag((phdat-nphdat)*nphupdownstat,0)

print 'producing figures...'
plotstuff(mina,maxa,minb,maxb,'nph_nphud_t.png')
datlist=filterhalfdiag((ph687dat-phdat)*ph687updownstat,0)+filterhalfdiag((nph687dat-nphdat)*nph687updownstat,1)
plotstuff(mina,maxa,minb,maxb,'ph687nphud_687phud_t.png')
datlist=filterhalfdiag(ph687dat,0)+filterhalfdiag(nph687dat,1)
plotstuff(mina,maxa,minb,maxb,'ph687nph_687ph_t.png')
datlist=filterhalfdiag(phdat,0)+filterhalfdiag(nphdat,1)
plotstuff(mina,maxa,minb,maxb,'nph_ph_t.png')
datlist=filterhalfdiag(nphdat,1)+filterhalfdiag((phdat-nphdat)*nphupdownstat,0)
plotstuff(mina,maxa,minb,maxb,'nph_nphud_c.png')
datlist=filterhalfdiag((ph687dat-phdat)*ph687updownstat,0)+filterhalfdiag((nph687dat-nphdat)*nph687updownstat,1)
plotstuff(mina,maxa,minb,maxb,'ph687nphud_687phud_c.png')
datlist=filterhalfdiag(ph687dat,0)+filterhalfdiag(nph687dat,1)
plotstuff(mina,maxa,minb,maxb,'ph687nph_687ph_c.png')
datlist=filterhalfdiag(phdat,0)+filterhalfdiag(nphdat,1)
plotstuff(mina,maxa,minb,maxb,'nph_ph_c.png')
print 'finished'
```

S3.4 K_D determination – NMR data post-processing (Python 2.7 script)

```
import csv
import numpy as np
from IPython.core.interactiveshell import InteractiveShell
InteractiveShell.ast_node_interactivity = "all"
from IPython.core.display import display,HTML
import matplotlib as mpl
import matplotlib.pyplot as plt
import os
from os import listdir
from scipy import optimize

#p1 path. can contain files, no folders, which are in alphabetical order matching the concentrations; for example,
#use 0.0.csv, 0.2.csv instead of 0.csv, 0.2.csv. First and last file have to have peaks in identical order.
#other files can't contain extra peaks.
#
fitfunc = lambda p, xb: ((cp+xb*cp+p[0])/(2*cp))-np.sqrt(((cp+xb*cp+p[0])/(2*cp))**2-xb)
conccutoff=0.5  ## cutoff (peptide concentration) at which the fraction of
p0 = [0.07] ## kD guess
selectset=1
if selectset == 1:
```

```
    p1="/media/hanskoss/nethadat/nmrtrans/nmrrec/tombunney/cSH2_special/hPLCg1_cSH2-pY_150710/"
    conclist=[0.0,0.2,0.4,1.0,1.6,1.8,2.0]
    cp=0.432 #1 #432 #protein concentration
    jacknlistset=[[0,1,2],[1,3,4],[1,2,4],[0,3,4],[1,2,3],[0,2,3]] #jackknife sets / sets selected for fitting
    errfunc = lambda p, x1, y1, x2, y2,x3,y3: np.r_[fitfunc(p,x1)-y1,fitfunc(p,x2)-y2,fitfunc(p,x3)-y3]
    finalplot=[0,1,2,3,4]
    titleset='cSH2 (-770) and pY783'
    filetitle='/home/hanskoss/peptidetrans/cSH2_770_pY783.png'
elif selectset == 2:
    p1="/media/hanskoss/nethadat/nmrtrans/nmrrec/tombunney/tandem_special/hPLCg1_nSH2cSH2_pY_111110/"
    conclist=[0.0,0.2,0.4,0.7,1.0,1.25]
    cp=0.2
    jacknlistset=[[0,1],[1,2],[0,3],[1,3],[2,3],[0,2]]
    errfunc = lambda p, x1, y1, x2, y2: np.r_[fitfunc(p,x1)-y1,fitfunc(p,x2)-y2]
    finalplot=[0,1,2,3]
    titleset='tandem-SH2 (-790) and pY783'
    filetitle='/home/hanskoss/peptidetrans/tandem_790_pY783.png'
elif selectset ==3:
    cp=0.117
    p1="/media/hanskoss/nethadat/nmrtrans/nmrrec/tombunney/cSH2_special/hPLCg1_cSH2_PDGFR_030112/"
    conclist=[0.0,0.2,0.4,0.6,0.8,1.0,1.2]
    jacknlistset=[[1,2,4],[2,3,4],[0,4,5],[0,2,4],[0,3,5],[2,4,5],[1,3,5],[0,1,3]]
    errfunc = lambda p, x1, y1, x2, y2,x3,y3: np.r_[fitfunc(p,x1)-y1,fitfunc(p,x2)-y2,fitfunc(p,x3)-y3]
    finalplot=[0,1,2,3,4,5]
    titleset='cSH2 (-790) and PDGFR'
    filetitle='/home/hanskoss/peptidetrans/cSH2_790_PDGFR.png'


maxdat=np.shape(conclist)[0]-1 #some useful abbreviation
flatten = lambda l: [item for sublist in l for item in sublist] #flattens lists

#reads and lists path contents, could be improved (folder structure and file naming has to be perfect)
def getdatalist(path2):
    global fnames,path
    path=p1+path2
    fnames=os.listdir( path )

#open CSV file and read in data
def prepselection(selection):
    for a in selection:
        colselect=[0,2,3,6,7,8]
        global abc
        global datlist
        global listx
        with open(path+'/'+fnames[a],'rb') as csvfile:
            datax = csv.reader(csvfile, delimiter=' ')
            i=0
            val = []
            for row in datax:
                val.append(row)
                i+=1
        valnp=np.array(val)
        np.shape(np.array(valnp))
        abc=np.shape(valnp)[0]
        listx=[]
        header=valnp[0]
        for x in np.arange(1,abc,1):
            listx.append(valnp[x][colselect])
        datlist=np.array(listx).astype('float64')
        return datlist, header[colselect], fnames[a]

#expands certain elements to a list of repeating elements for shape and accessibility reasons
def expfillist(num):
    Posfnx=[]; statex=[]
    for a in np.arange(0,np.shape(datcoll[num]['Position_F1'])[0]):
        Posfnx.append(datcoll[num]['file'])
        statex.append(num)
    return np.array(Posfnx), np.array(statex)

#useful to find all elements equal to a in list lst
def find(lst, a):
    return [i for i, x in enumerate(lst) if x==a]

#start here
getdatalist('data')

#after reding in data, create dictionary with peak list and other information.
datcoll=[]
for x in np.arange(np.shape(fnames)[0]):
    datcoll.append({})
    datcoll[x]['data']=prepselection([x])[0]
    datcoll[x]['header']=prepselection([x])[1]
    datcoll[x]['file']=prepselection([x])[2]
    datcoll[x]['conc']=conclist[x]
    for y in np.arange(np.shape(datcoll[x]['header'])[0]):
        datcoll[x][datcoll[x]['header'][y]]=datcoll[x]['data'][:,y]

#Creation of a reference peak dictionary to easily access information for each reference peak.
#reference peaks are peaks at the lowest and the highest concentration (have to be alphabetically
#at the beginning and the end of the folder.)
PosF1ref=datcoll[0]['Position_F1']
```

```
Posfn=expfillist(0)[0]; Posstat=expfillist(0)[1]
PosF1ref=np.append(PosF1ref,datcoll[maxdat]['Position_F1'])
PosF2ref=datcoll[0]['Position_F2']
PosF2ref=np.append(PosF2ref,datcoll[maxdat]['Position_F2'])
Posvol=datcoll[0]['Volume']
Posvol=np.append(Posvol,datcoll[maxdat]['Volume'])
Posfn=np.append(Posfn,expfillist(maxdat)[0])
Posstat=np.append(Posstat,expfillist(maxdat)[1])
Posres=datcoll[0]['Number']
Posres=np.append(Posres,datcoll[maxdat]['Number'])
Posref={}
Posref['shifts']=np.transpose(np.array([PosF1ref,PosF2ref]))
Posref['files']=Posfn;Posref['residue']=Posres;Posref['state']=Posstat;Posref['volume']=Posvol

# This goes through all peak lists and finds the reference peak which is closest to the peak in question.
for a in np.arange(np.shape(datcoll)[0]):
    datcoll[a]['refpeak']=[]
    for b in np.arange(np.shape(datcoll[a]['Position_F1'])[0]):
        datcoll[a]['refpeak'].append(np.argmin((datcoll[a]['Position_F1'][b]-
Posref['shifts'][:,0])**2+(datcoll[a]['Position_F2'][b]-Posref['shifts'][:,1])**2))

# This routine goes through the reference peak lists and scans all other peak lists for matching peaks (the
# matches have been determined in the previous routine. It fills a "matched" dictionary for each reference peak
# so that all relevant peaks for a certain concentration, residue and state are collected.
Posref['matched']=[]
for a in np.arange(np.shape(Posref['volume'])[0]):
    Posref['matched'].append([])
    Posref['matched'][a]={}
    matchingx=[]; volx=[]; shiftx=[]; filex=[];concx=[]
    for b in np.arange(np.shape(datcoll)[0]):
        for c in np.arange(np.shape(datcoll[b]['Position_F1'])[0]):
            if a == datcoll[b]['refpeak'][c]:
                matchingx.append([b,c])
                shiftx.append([datcoll[b]['Position_F1'][c],datcoll[b]['Position_F2'][c]])
                volx.append(datcoll[b]['Volume'][c])
                filex.append(datcoll[b]['file'])
                concx.append(datcoll[b]['conc'])
    Posref['matched'][a]['shifts']=shiftx
    Posref['matched'][a]['volume']=volx
    Posref['matched'][a]['position']=matchingx
    Posref['matched'][a]['file']=filex
    Posref['matched'][a]['conc']=concx

# The following routine checks whether there are peak volume data for a given residue at a given
# concentration for the bound and the unbound state. The fraction of the bound state for each residue and
# concentration is then determined. This can in principle be modified to merge information from the bound
# and the unbound peaks.
collectx=[];collecty=[]
for a in list(np.unique(Posref['residue'])):
    x=[];y=[]
    for b in conclist:
        state0pos=list(set(find(Posref['residue'], int(a))).intersection(find(Posref['state'], 0)))[0]
        state1pos=list(set(find(Posref['residue'], int(a))).intersection(find(Posref['state'], maxdat)))[0]
        vol0=Posref['volume'][state0pos]
        vol3=Posref['volume'][state1pos]
        try:
            st0pos2=find(Posref['matched'][state0pos]['conc'],b)[0]
            vol1=Posref['matched'][state0pos]['volume'][st0pos2]
            if b < conccutoff:
                x.append(b);
                y.append(1-vol1/vol0)
        except:
            pass
        try:
            st1pos2=find(Posref['matched'][state1pos]['conc'],b)[0]
            vol2=Posref['matched'][state1pos]['volume'][st1pos2]
            if b >= conccutoff:
                x.append(b);
                y.append(vol2/vol3)
        except:
            pass
    collectx.append(np.array(x));collecty.append(np.array(y))

#jackknife fits and plots
plt.rcParams.update({'font.size': 14})
p1collect=[]
for jacknlist in jacknlistset:
    guru3=[]
    for x in jacknlist:
        guru3.append(collectx[x]); guru3.append(collecty[x])
    guru4=tuple(guru3)
    p1,success = optimize.leastsq(errfunc, p0, args=guru4) #args=(Tx, tX, Ty, tY)
    p1collect.append(p1)
    num_points = 100
    x_dat = np.linspace(0, 2, num_points)
    y_dat = fitfunc(p1,x_dat)
    plt.clf()
    colorlist=['k','r','g','b','cyan','m','y']
    cnt=0
    for bx in jacknlist: #np.arange(np.shape(np.unique(Posref['residue']))[0]):
        a=plt.figure(2)
```

```
a=plt.plot(list(collectx[bx]),list(collecty[bx]),marker='x',linestyle='None',markersize=10,c=colorlist[cnt])
        a=plt.xlabel('peptide/protein ratio')
        a=plt.ylabel('bound fraction')
        cnt+=1
    a=plt.plot(x_dat,y_dat,marker='None',linestyle='-',color='k')
    a=plt.show()
print 'kD is ' + str(1000*np.average(p1collect))+' uM +- ' + str(1000*np.std(p1collect))

cnt=0
for bx in finalplot: #np.arange(np.shape(np.unique(Posref['residue']))[0]):
    a=plt.figure(2)
    a=plt.plot(list(collectx[bx]),list(collecty[bx]),marker='x',linestyle='None',markersize=10,c=colorlist[cnt])
    a=plt.xlabel('peptide/protein ratio')
    a=plt.ylabel('bound fraction')
    cnt+=1
num_points = 100
x_dat = np.linspace(0, 2, num_points)
y_dat = fitfunc([np.average(p1collect)],x_dat)
a=plt.title(titleset)
a=plt.plot(x_dat,y_dat,marker='None',linestyle='-',color='k')
a=plt.savefig(filetitle)
a=plt.show()
```

Supporting Figures

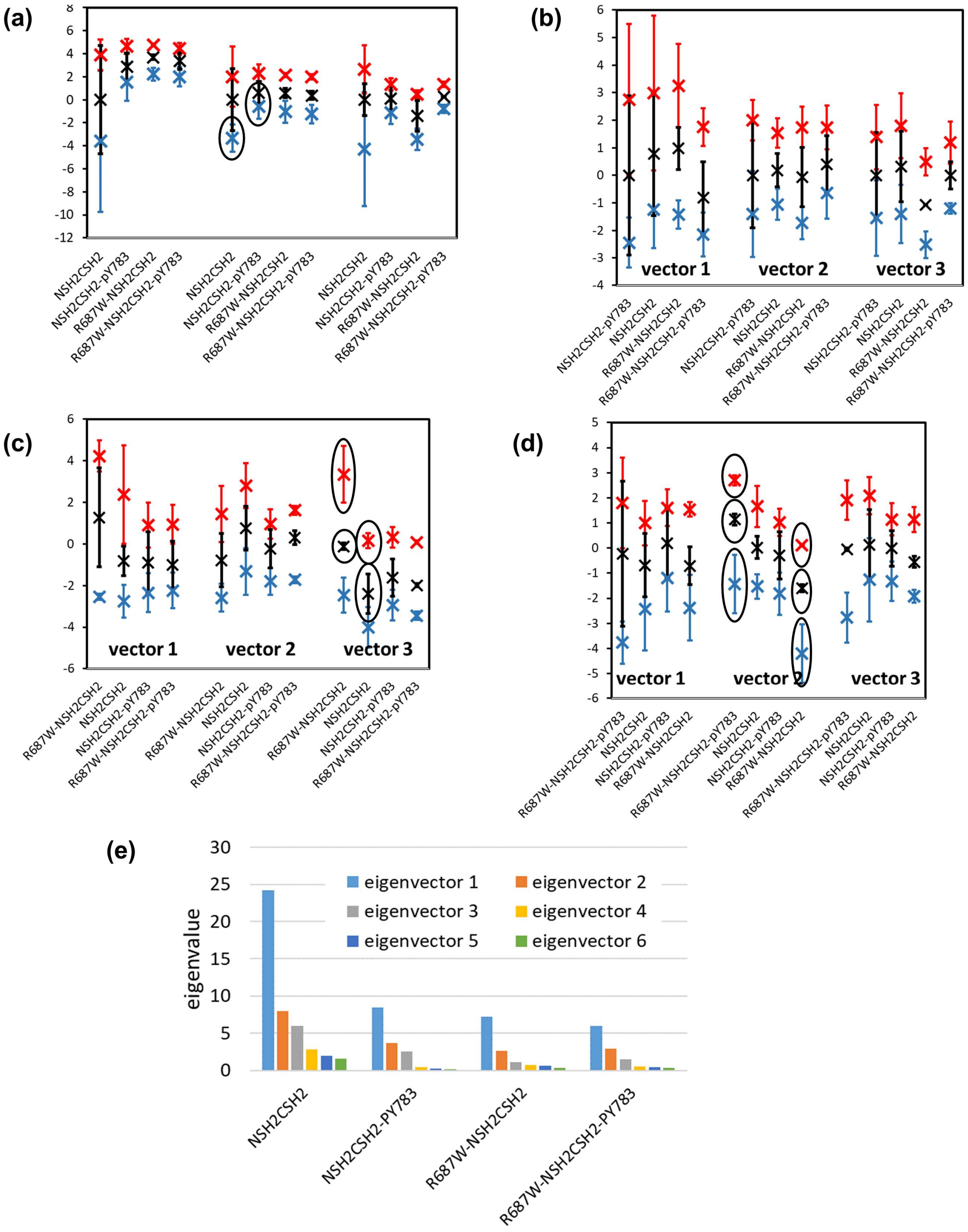

Figure S1. Differences between NSH2CSH2, NSH2CSH2-pY^{783}, R687W-NSH2CSH2 and R687W-NSH2CSH2-pY^{783} molecular dynamics trajectories revealed by principal component analysis.

a-d: Projections of trajectories on PCA eigenvectors reveal differences between trajectories, represented by the eigenvectors. Eigenvectors were generated from pooled trajectories (50-100 ns) for each of the four constructs: (a) NSH2CSH2, (b) NSH2CSH2-pY^{783}, (c) R687W-NSH2CSH2 and (d) R687W-NSH2CSH2-pY^{783}. The projection of any trajectory on any eigenvector covers a certain range

(which varies for each trajectory). Rather than this range, the upper and lower limit of this range as well as the average, along with the errors for these limits, are shown for some of the projections. The upper and lower range limits and the average were calculated by projecting the different individual trajectories of a construct on the eigenvector in question (giving average and standard deviation for both upper and lower limit).

Example for the PCA of NSH2CSH2 - eigenvector 2 (panel a): The 1D projection of the NSH2CSH2-pY^{783} trajectories on this eigenvector is different from the NSH2CSH2 projection - the lower limit of the projection range (circled) does not overlap with the projection of NSH2CSH2. This analysis reveals that NSH2CSH2 and NSH2CSH2-pY^{783} can be expected to differ for eigenvector 2 of the NSH2CSH2 PCA. Statistical significance is not implied, but based on this analysis the (mostly C-terminal and pre-C-terminal) motion represented by this eigenvector (*Fig. 2a*) is realized to different extents between these two constructs. Other relevant projections to describe differences between WT and ARG687TRP constructs are marked in panels (c) and (d).

(e) The magnitude of the first six eigenvalues obtained from the PCA for each of the four MD trajectories. Example: the first three eigenvalues correspond to the three eigenvectors on which various trajectories are projected in panel (a). NSH2CSH2 eigenvector 2 obtained from pooled NSH2CSH2 data describes a motion which differs between NSH2CSH2 and NSH2CSH2-pY^{783}. The eigenvalue corresponding to this eigenvector appears relatively large.

(a)

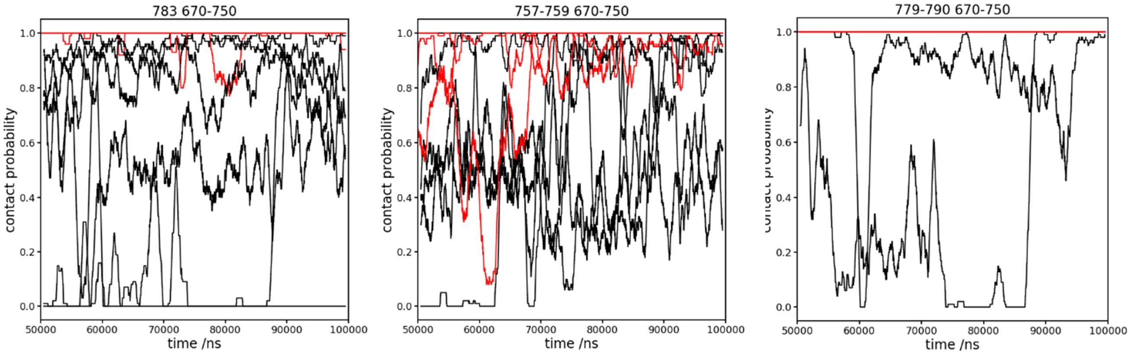

(b)

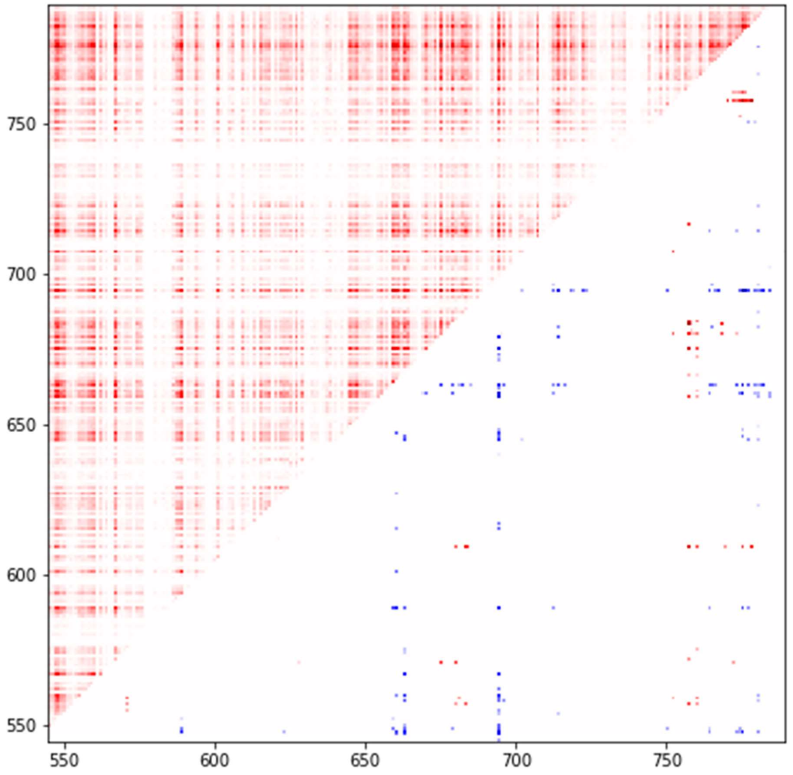

Figure S2. Molecular dynamics simulations predict dynamic differences between NSH2CSH2 and NSH2CSH2-pY^{783} for residues in the pre-C-terminus and the C-terminal linker, supplemental contact analysis and mutual information data. (a) Probability of contact between C-terminal linker residues and the cSH2 domain, shown for different MD trajectories. Color key (numbers of available trajectories in brackets): black – NSH2CSH2 (n = 6 trajectories); red – NSH2CSH2-pY^{783} (n = 4). Any number of contacts (< 2.5 Å) at a given time point is counted as a single contact event, yielding a contact probability for any 1 ns sliding average time bin. (b) Mutual information between side chain dihedrals of NSH2CSH2 and NSH2CSH2-pY783 trajectories. See *Fig. 2b* for details and labels. The matrix is shown here for the entire construct, including the nSH2 domain.

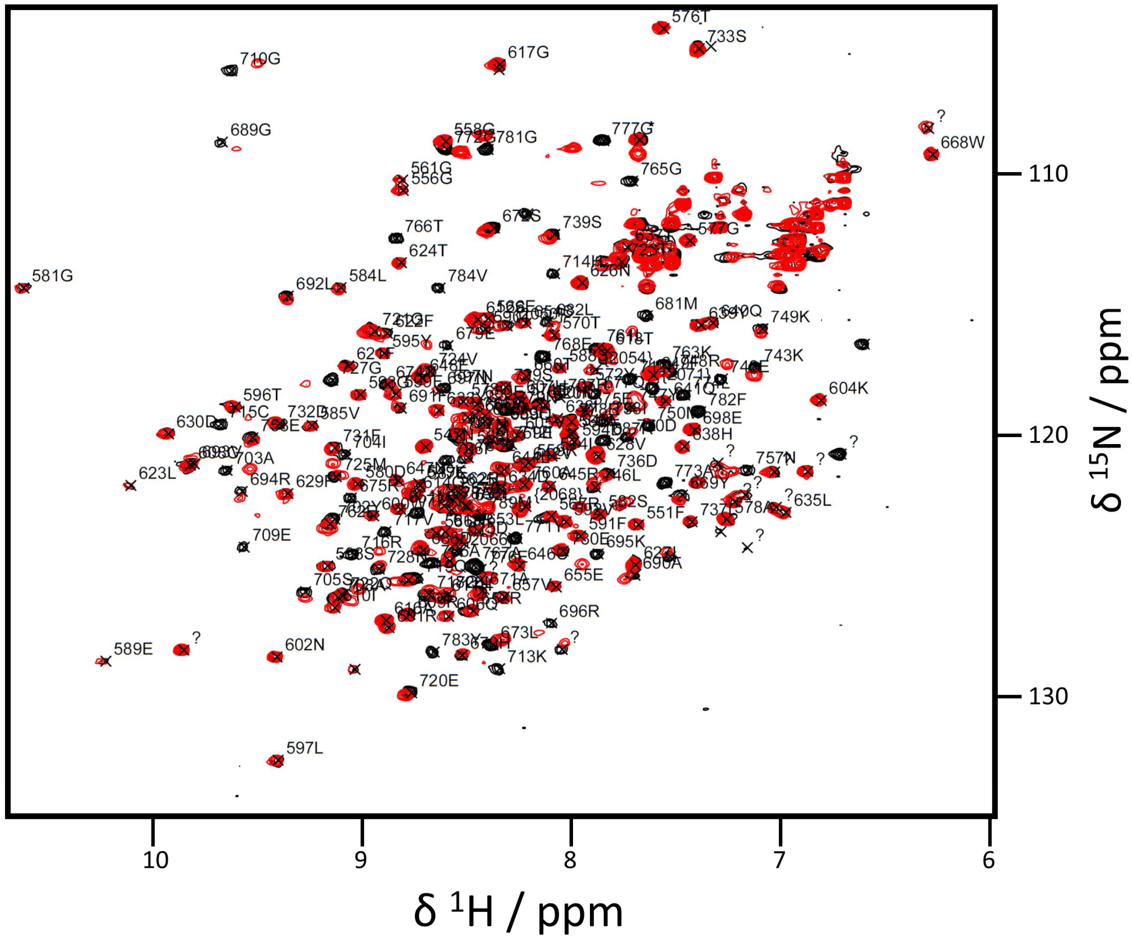

Figure S3: $^{1}H,^{15}N$-HSQC spectra of NSH2CSH2 (red) and NSH2CSH2-pY^{783} (black). Spectra were recorded at 700 MHz. For more extensive backbone assignment information refer to the relevant BMRB depositions (BMRB entry 27496).

(a)

construct	MW / kDa	conc / mg ml^{-1}	deleted points	R_g / nm	I_0	D_{max} / nm	Vol_{excl} / nm^3	back-calc. MW / kDa
NSH2CSH2-pY^{783}	28.5	5.8	32	2.43±0.20	21.83±0.05	8	38	21
	28.5	4.1	28	2.38±0.10	21.18±0.05	8	39	21
	28.5	2.2	34	2.27±0.07	20.03±0.08	7	40	20
NSH2CSH2	28.5	5.8	26	2.76±0.29	26.57±0.06	9	42	26
	28.5	4.0	31	2.62±0.18	24.43±0.07	9	40	24
	28.5	2.2	28	2.48±0.29	22.05±0.09	8	39	22

(b)

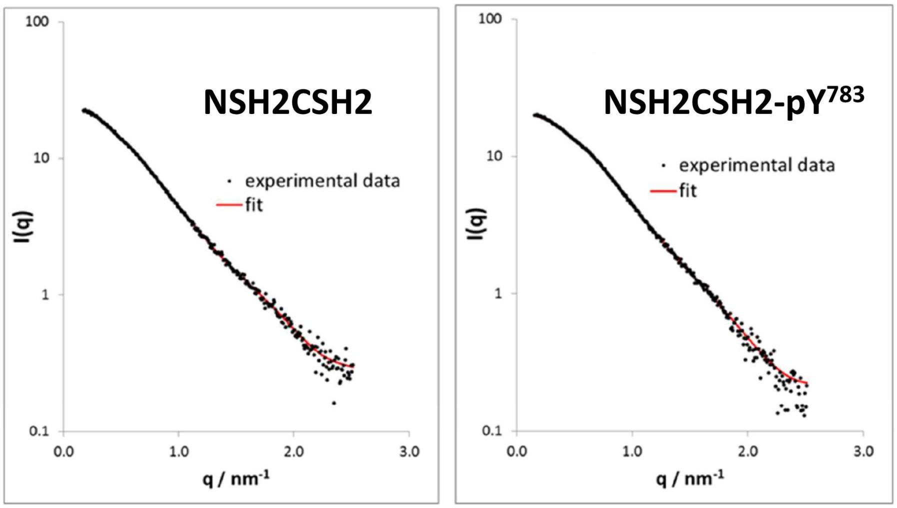

(c)

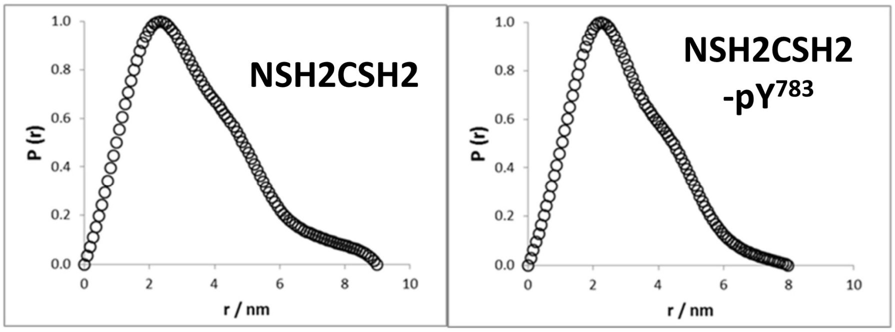

Figure S4: SAXS data recorded for NSH2CSH2 and NSH2CSH2-pY^{783} reveal an aggregation propensity for NSH2CSH2. (a) General parameters extracted from the SAXS data. The molecular weight was back-calculated from I_0 for the construct and I_0 for a reference sample of bovine serum albumin. The gyration radius and volume of NSH2CSH2-pY^{783} is generally lower and more stable upon concentration reduction than the respective values for NSH2CSH2. This might indicate a weak propensity to aggregate for NSH2CSH2. (b) Experimental data and reciprocal fits of the distance distribution functions. (c) Distance distribution functions. The shapes of these curves match to a two-domain protein of the given size. However, the presence of some larger distances, especially for NSH2CSH2, suggests that some aggregation is present.

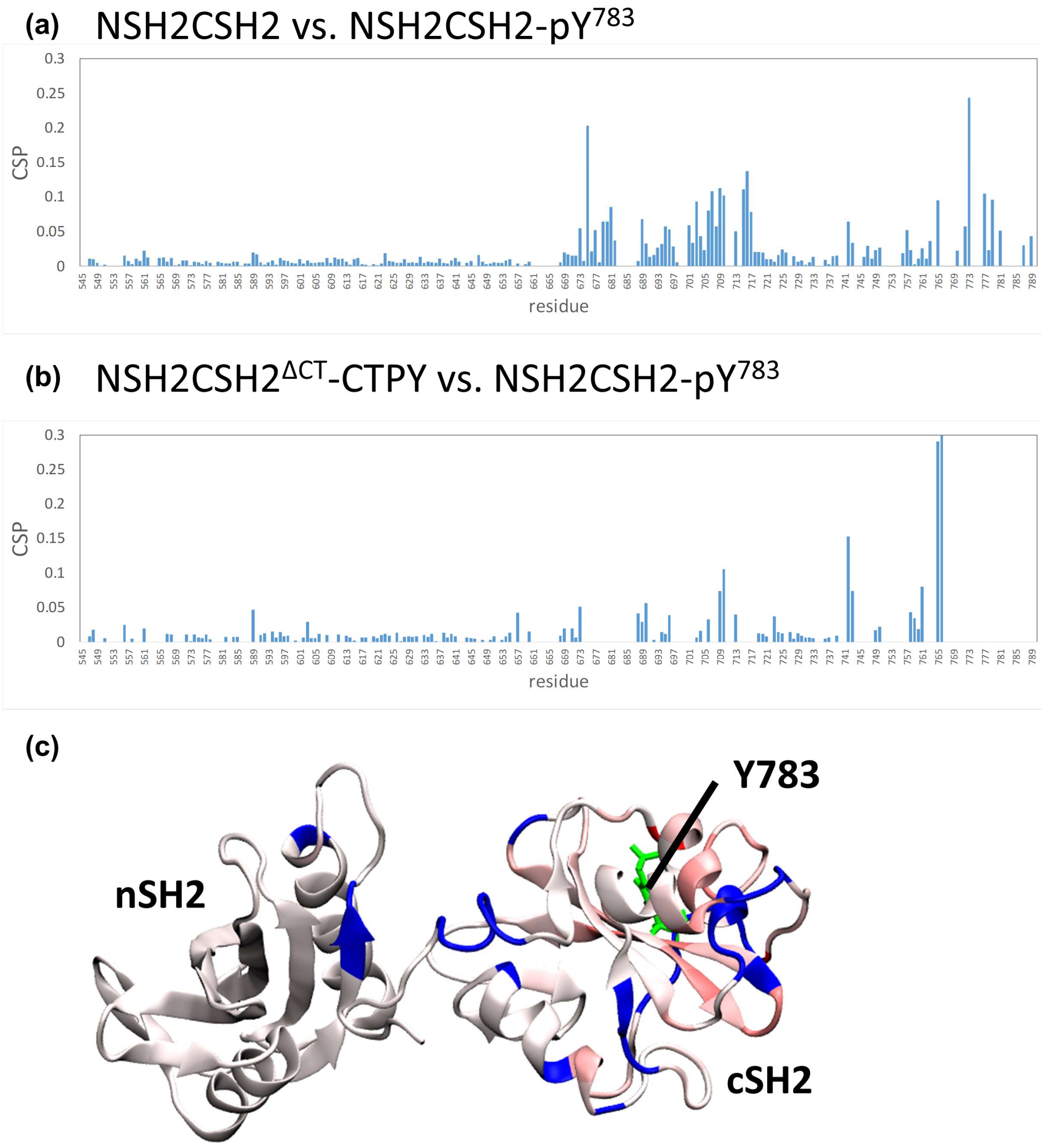

Figure S5: Chemical shift perturbation (CSP) analysis for tandem SH2 constructs in ^{1}H,^{15}N-HSQC spectra (recorded at 700 MHz). (a) and (c): CSPs between NSH2CSH2 and NSH2CSH2-pY^{783} contructs. (b): CSPs between NSH2CSH2$^{\Delta CT}$-CTpY and NSH2CSH2-pY^{783} constructs. (c): Illustration of the CSPs between NSH2CSH2 and NSH2CSH2-pY^{783}, projected on the structural model based on the tandem domain crystal structure 4FBN. Color scheme: white (small CSP) to red (large CSP); blue: no data. The atoms of Tyr783 are highlighted in green.

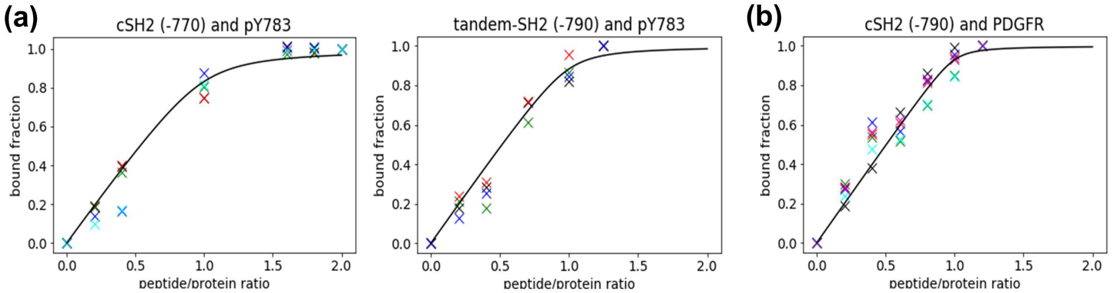

Figure S6: Peptide / protein titrations for K_D determination. The black line was plotted based on the average K_D determined from several jackknife-sampled fits. Experimental data were obtained from the peak volume ratios (bound fraction) for different residues, each plotted here in a different color. For each jackknife-sampled fit, data sets from 2-3 residues were taken for each K_D determination.

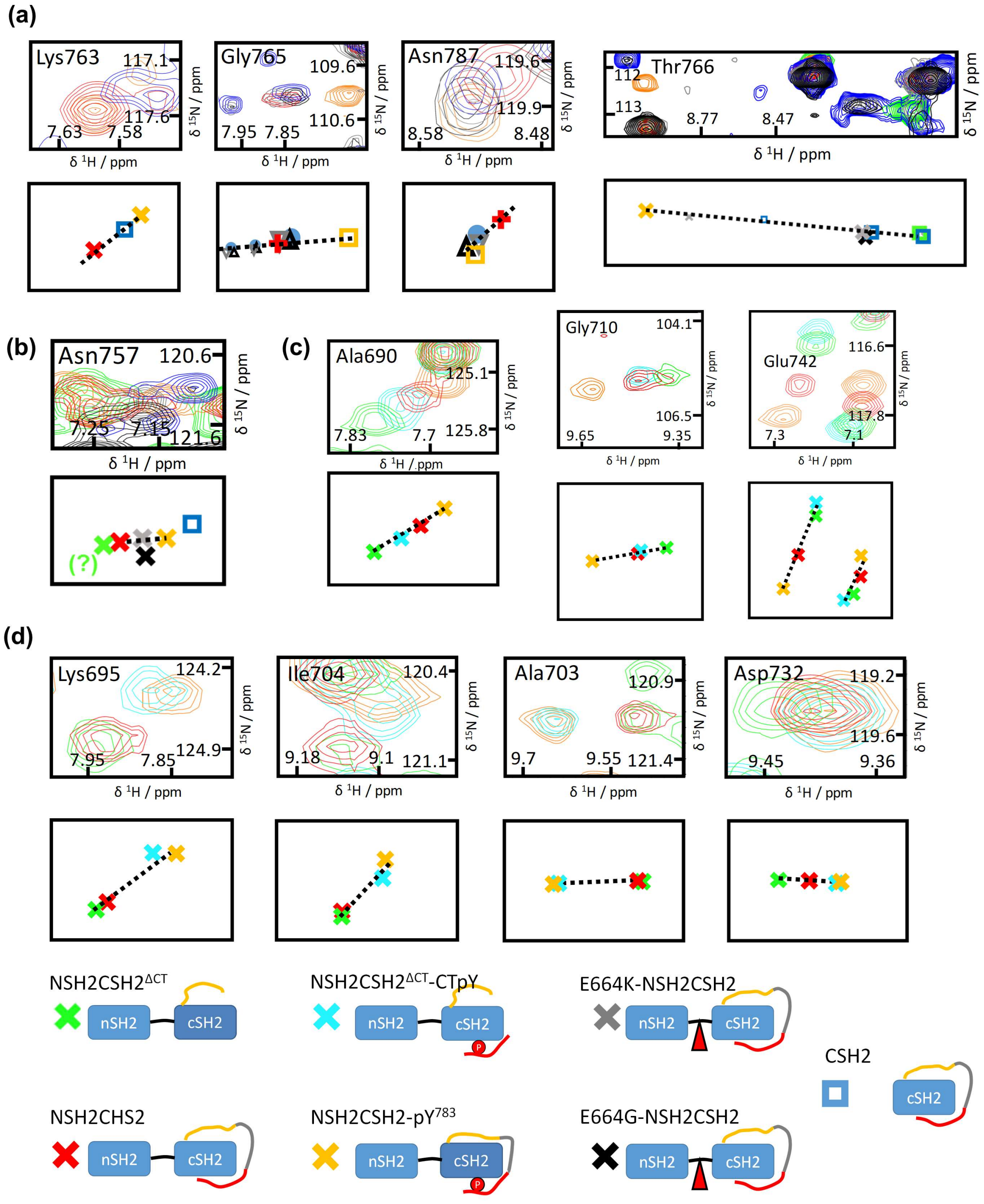

Figure S7: Allosteric pathways connecting the Tyr783 phosphorylation site with the nSH2-cSH2 junction; see also Fig. 4. ^{1}H,^{15}N-HSQC spectra are shown next to supporting symbolic representations of the spectra (panels a and b - 700 MHz; panels c and d - 600 MHz; legend at bottom of figure).
(a) The peaks are shifted towards the ‘closed’ state when the nSH2 domain is absent (CSH2) or when nSH2-cSH2 junction mutations are present, confirming dynamic allosteric communication via the extended C-terminus (see also *Fig. 4b*).

(b) The cross peak for Asn757 in the CSH2 spectrum is located on an *extension* of the vector connecting the corresponding cross peak positions in the spectra of NSH2CSH2 and NSH2CSH2-pY^{783}. It can be inferred that residue Asn757 in NSH2CSH2-pY^{783} is not fully 'closed' in CSH2. The exact position of the corresponding NSH2CSH2$^{\Delta CT}$ cross peak is uncertain.
(c) For some residues, the cross peaks for NSH2CSH2-pY^{783} and NSH2CSH2$^{\Delta CT}$-CTpY do not coincide, indicating a C-terminal linker-dependent effect (see also *Fig. 4a*).
(d) For some residues, especially those located in the β-sheet, the peaks for NSH2CSH2-pY^{783} and NSH2CSH2$^{\Delta CT}$-CTpY coincide, indicating a C-terminal linker-*in*dependent effect.

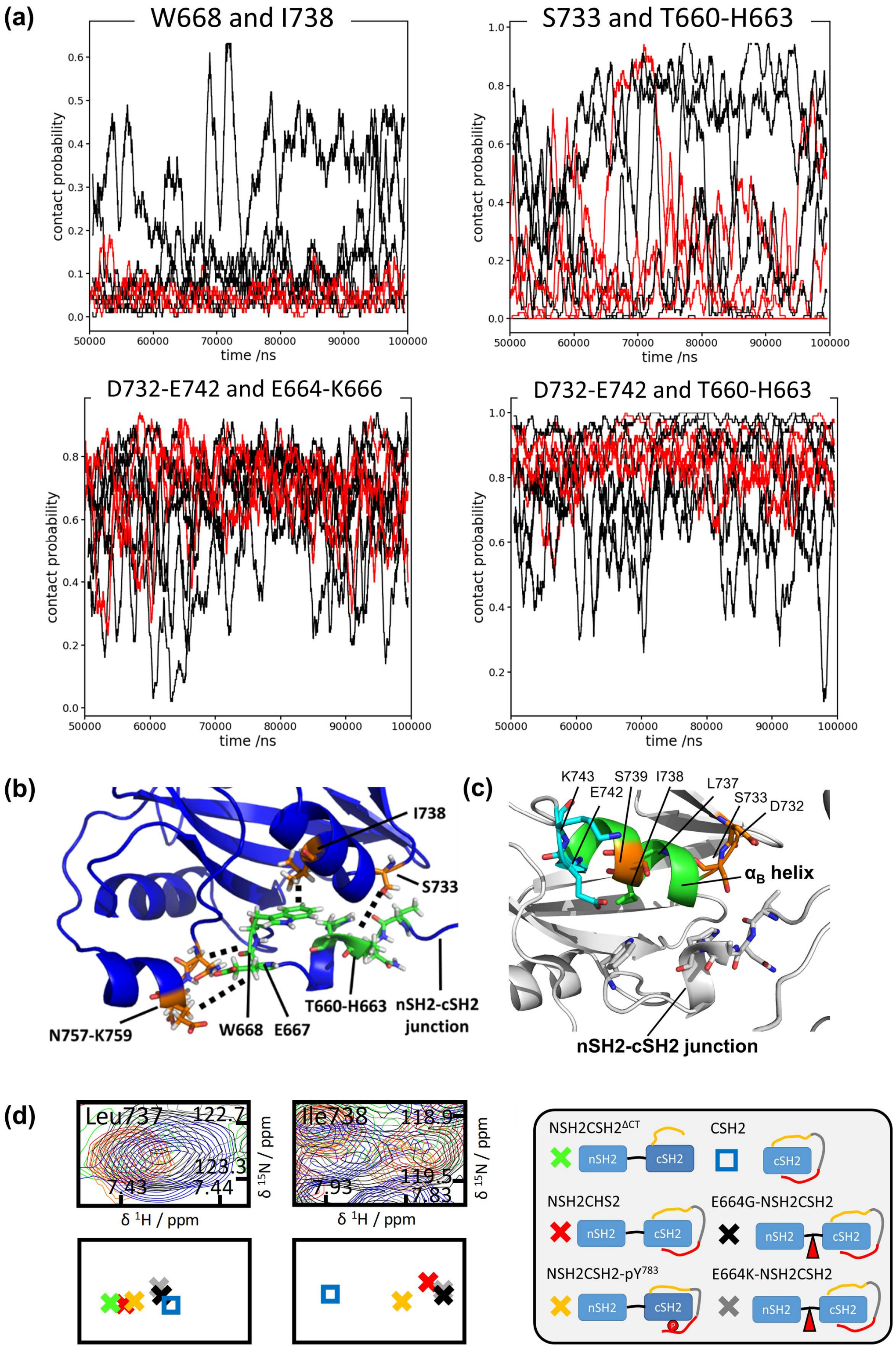

Figure S8: Contacts between the nSH2-cSH2 junction and the α_B helix and the extended C-terminus. (a) Contact probabilities between α_B helix and nSH2-cSH2 junction, shown for different MD trajectories. Color key (numbers of available trajectories in brackets): black, NSH2CSH2 (6); red, NSH2CSH2-pY^{783} (4). Any number of contacts (< 2.5 Å) at a given time point is counted as a single contact event, yielding the contact probability over a 1 ns sliding average time bin.

(b) This cSH2 model (based on the 4FBN structure) shows residues and contacts that were found to be relevant for the interaction between the nSH2-cSH2 junction with the pre-C-terminus and the α_B helix.

(c) Depiction of part of the cSH2 domain structure illustrating the location of α_B residues discussed in the main text: cyan, residues whose chemical shifts are perturbed mostly by the C-terminal linker-dependent allosteric pathway; orange, residues whose peaks are perturbed mostly by the C-terminal linker-independent pathway.

(d) Impact of the presence or modification of the nSH2-cSH2 junction on α_B helix residues Leu737 and Ile738. ^{1}H,^{15}N-HSQC spectra are shown next to supporting symbolic representations of the spectra (700 MHz, color key shown on the right).

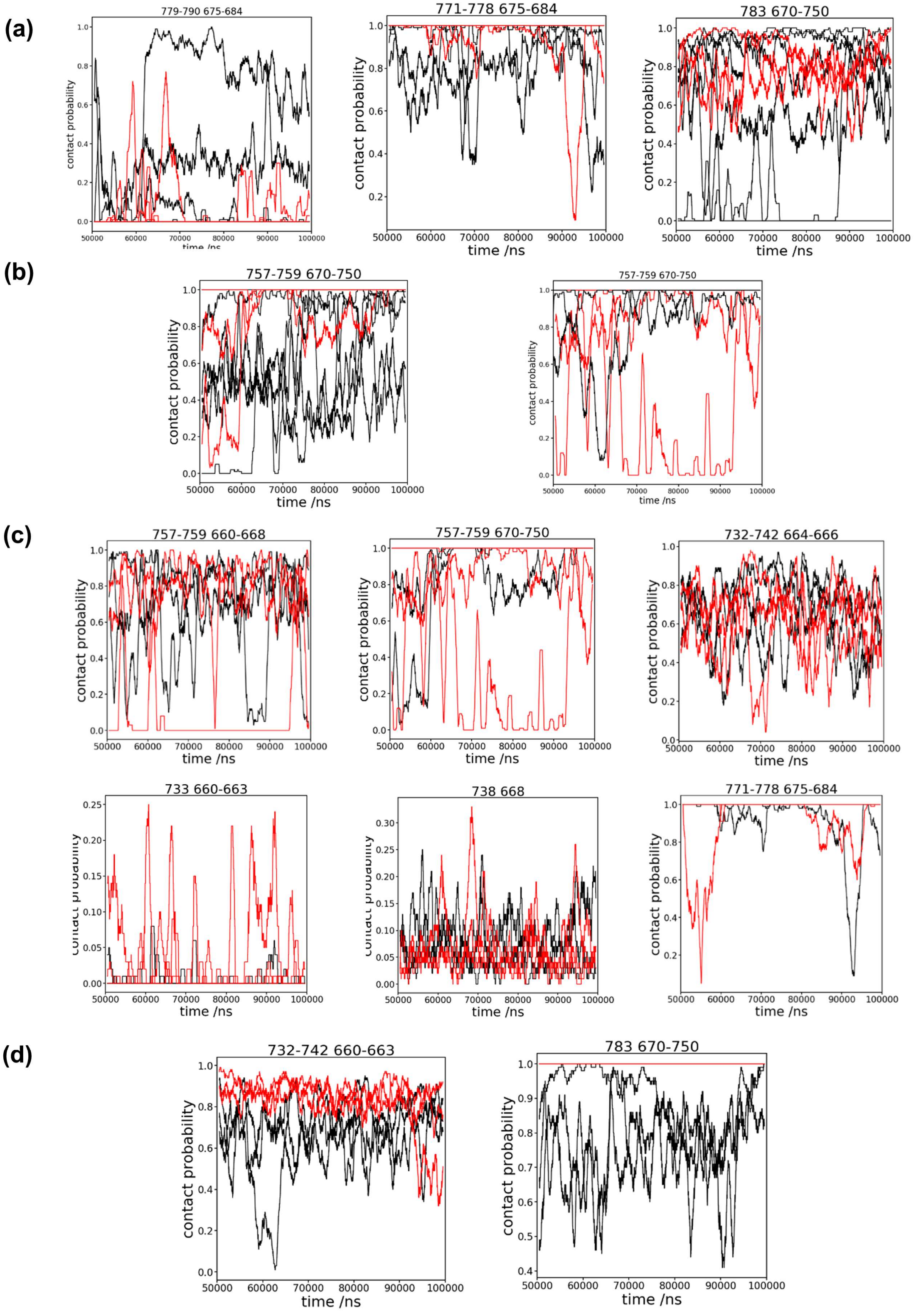

Figure S9: MD contact analyses for Arg687Trp mutant constructs. Contact probabilities are shown for different MD trajectories. The number of region pairs which have clearly different contact probabilities between nonphosphorylated and phosphorylated Arg687Trp states is much lower than for the WT. Any number of contacts (< 2.5 Å) at a given time point is counted as a single contact event, yielding a contact probability over a 1 ns sliding average time bin. Labels at the top of the figure

indicate the specific contact regions that were evaluated. (a) Comparison of NSH2CSH2 (black) with R687W-NSH2CSH2 (red) constructs, contacts between C-terminal regions and cSH2 domain regions. (b) Contacts between pre-C-terminus and cSH2 domain. left: Comparison of NSH2CSH2 (black) with R687W-NSH2CHS2 (red); right: comparison of NSH2CHS2-pY^{783} (black) with R687W-NSH2CSH2-pY^{783} (red). (c) Comparison of R687W-NSH2CSH2 (black) with R687W-NSH2CSH2-pY^{783} (red): differencesdo not appear to be significant based on inspection of the trajectories; much clearer differences were shown for the respective WT contact pairs as shown in other figures. (d) Comparison of R687W-NSH2CSH2 (black) with R687W-NSH2CSH2-pY^{783} (red); differences appear to be significant based on inspection of the trajectories.

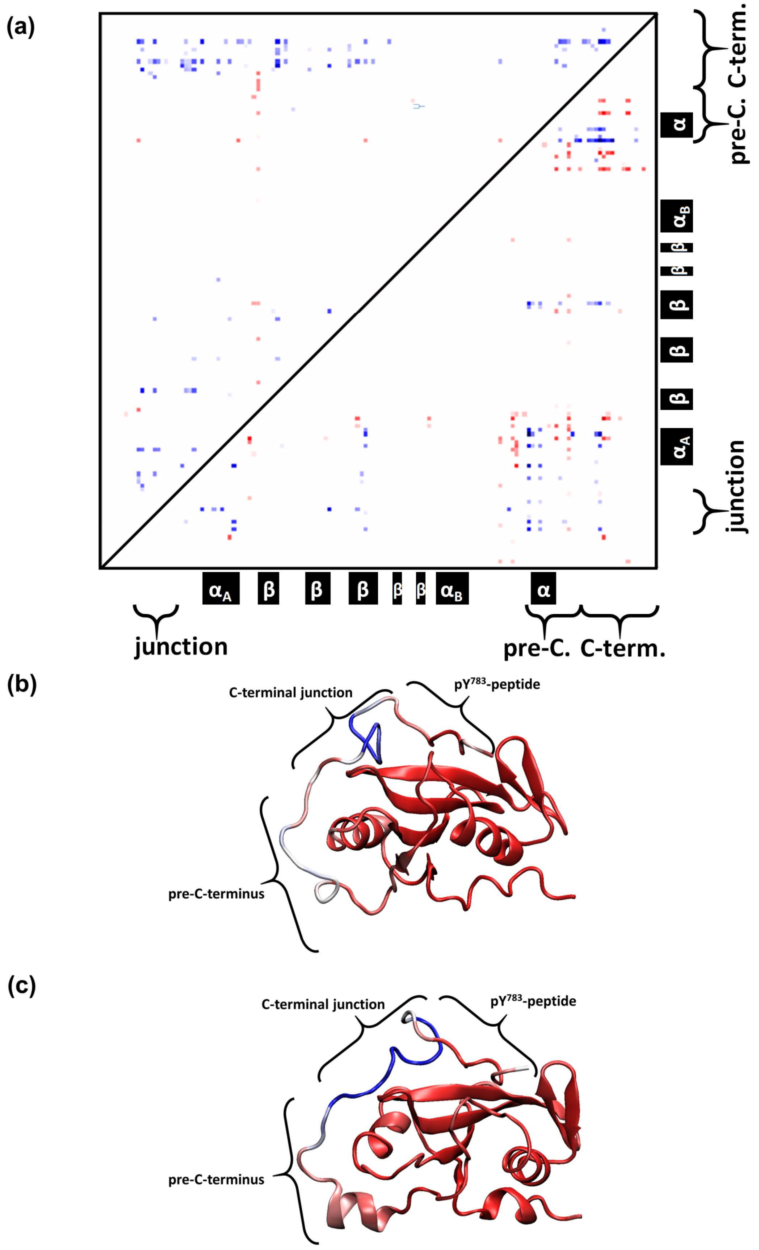

Figure S10: Molecular dynamics simulations predict dynamic differences between WT and Arg687Trp constructs for residues in the pre-C-terminus and the C-terminal linker. (a) Mutual side chain dihedral information, differences between WT and Arg687Trp mutants. The filtered results (t-test) for the cSH2 domain are shown in this panel. Upper left half: mutual information difference between NSH2CSH2 and R687W-NSH2CSH2. Lower right half: mutual information difference between NSH2CSH2-pY^{783} and R687W-NSH2CSH2-pY^{783}. Red indicates an increase in mutual information in the mutated construct; blue, decrease in mutual information. (b-c) RMS fluctuation of Arg687Trp eigenvectors: (b) R687W-NSH2CSH2-pY^{783} eigenvector 2; (c) R687W-NSH2CSH2 eigenvector 3 containing information about differences in backbone motions in the cSH2 domain between Arg687Trp and WT proteins (see also *Fig. 2b* and *Fig. S1*).

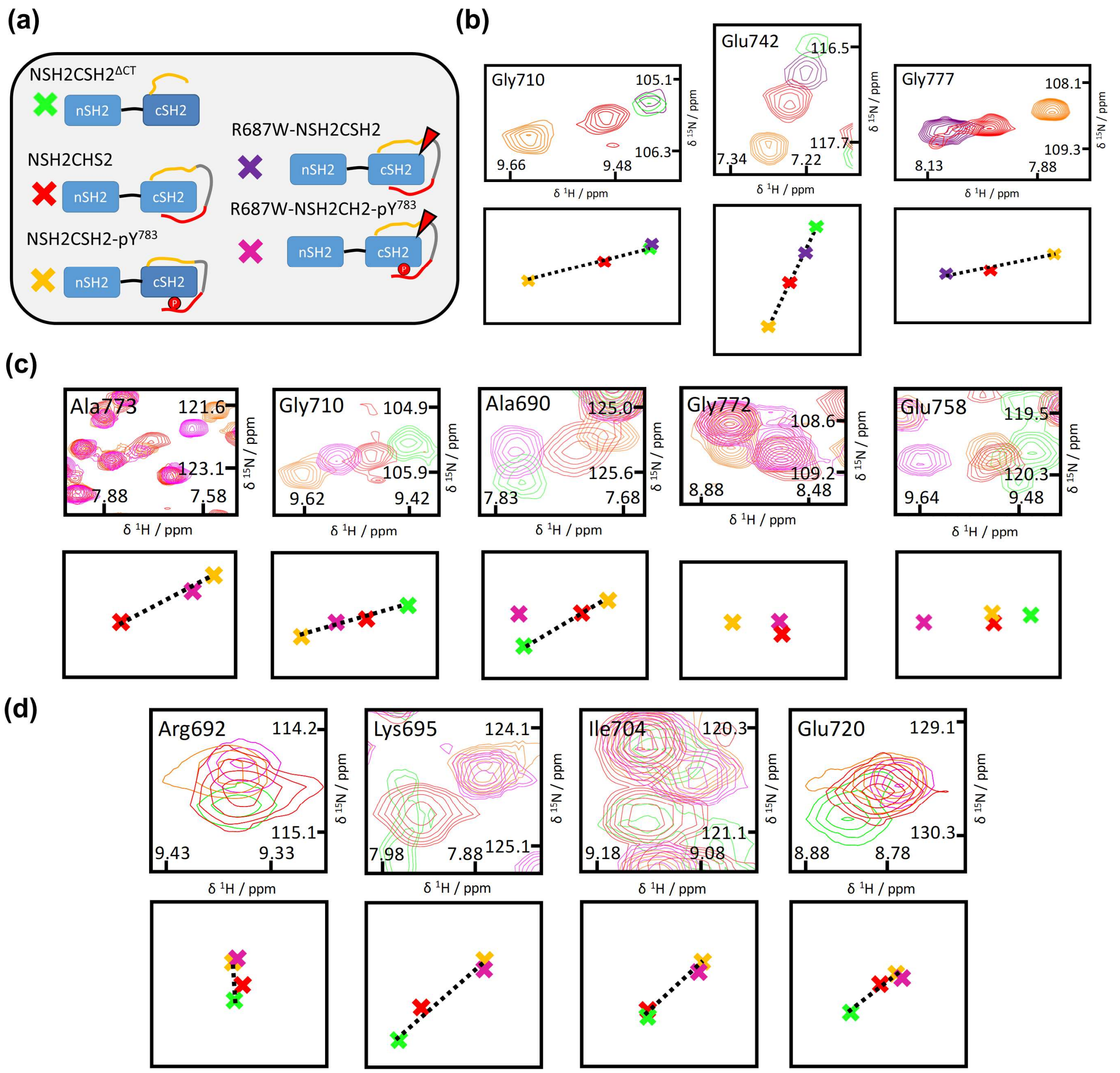

Figure S11: Specific effect of the Arg687Trp mutation on the C-terminal linker dependent pathway (additional examples, linked to *Fig. 6*).

(a) Key for panels (b-d), showing the correspondances between constructs and symbols/colors.

(b) Similar to the situation in R687W-NSH2CSH2-pY^{783}, the cross peaks for many residues in R687W-NSH2CSH2 are shifted toward the 'closed' (C) state with respect to WT constructs. ^{1}H,^{15}N-HSQC spectra were recorded at 950 MHz (R687W-NSH2CSH2) or 700 MHz (all others).

(c) Cross peaks for residues associated mostly with the C-terminal linker-dependent pathway often display large chemical shift perturbations when comparing Arg687Trp constructs with WT constructs. ^{1}H,^{15}N-HSQC spectra were recorded at 600 MHz.

(d) Cross peaks for residues that are mostly associated with the C-terminal linker-independent pathwa, are not or hardly shifted in Arg687Trp constructs with respect to the WT protein. ^{1}H,^{15}N-HSQC spectra were recorded at 600 MHz.

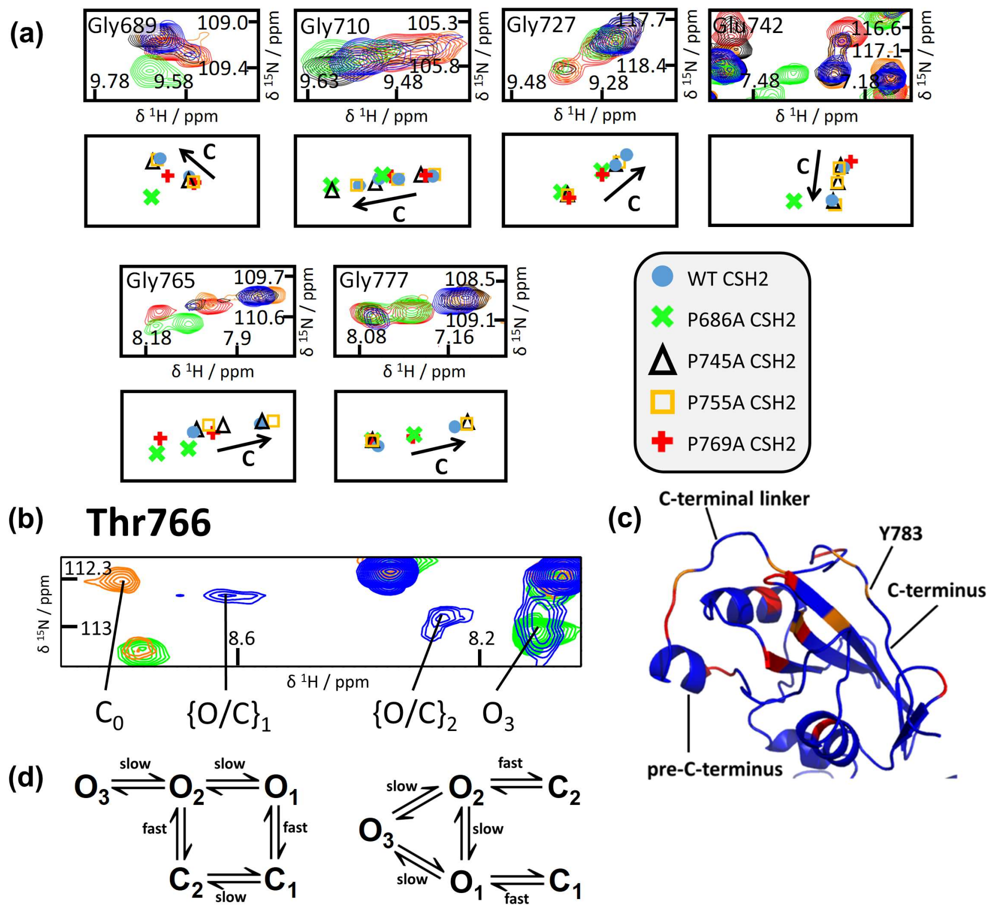

Figure S12: Multistate fast and slow exchange in CSH2 and NSH2CSH2 constructs. (a) Chemical shift analysis comparing $^{1}H,^{15}N$-HSQC spectra (600 MHz) for various CSH2 constructs without or with Pro→Ala mutations (see color key). Pro→Ala mutationsdramatically shift the exchange behavior of multiple residues. The spatially close residues Pro686Ala and Pro769Ala substitutions often tilt the fast dynamic equilibrium towards the 'open' state. The direction of the vector towards the 'closed' state is indicated by the arrow. (b) The CSH2 $^{1}H,^{15}N$-HSQC spectrum (blue) for residue Thr766 shows three cross peaks. One of them is at the same position as that detected for NSH2CSH2$^{\Delta CT}$ (green); in the corresponding O_3 state, the pre-C-terminus is locally 'open'. The 'closed' state is not present for CSH2; the corresponding NSH2CSH2-pY^{783} cross peak (orange) is shown for reference. The other two CSH2 cross peaks represent states which are exchanging rapidly between 'closed' and 'open' forms $\{C \rightleftharpoons O\}_2$; $\{C \rightleftharpoons O\}_3$. The resulting overall kinetic scheme is $\{O \rightleftharpoons^{*} C\}_1 \rightleftharpoons^{\dagger} \{O \rightleftharpoons^{*} C\}_2 \rightleftharpoons^{\dagger} O_3$, where * and † denote fast and slow exchange, respectively (for specific examples, see panel (d)). (c) Presence of slow exchange (cross peak doubling) in CSH2 (projected on the cSH2 domain model derived from the 4FBN crystal structure): red, slow exchange confirmed; orange, slow exchange likely. (d) Kinetic schemes exemplifying specific variations of the general scheme found for residue Thr766, $\{O \rightleftharpoons^{*} C\}_1 \rightleftharpoons^{\dagger} \{O \rightleftharpoons^{*} C\}_2 \rightleftharpoons^{\dagger} O_3$, where * and † denote fast and slow exchange, respectively (see also panel (b)).

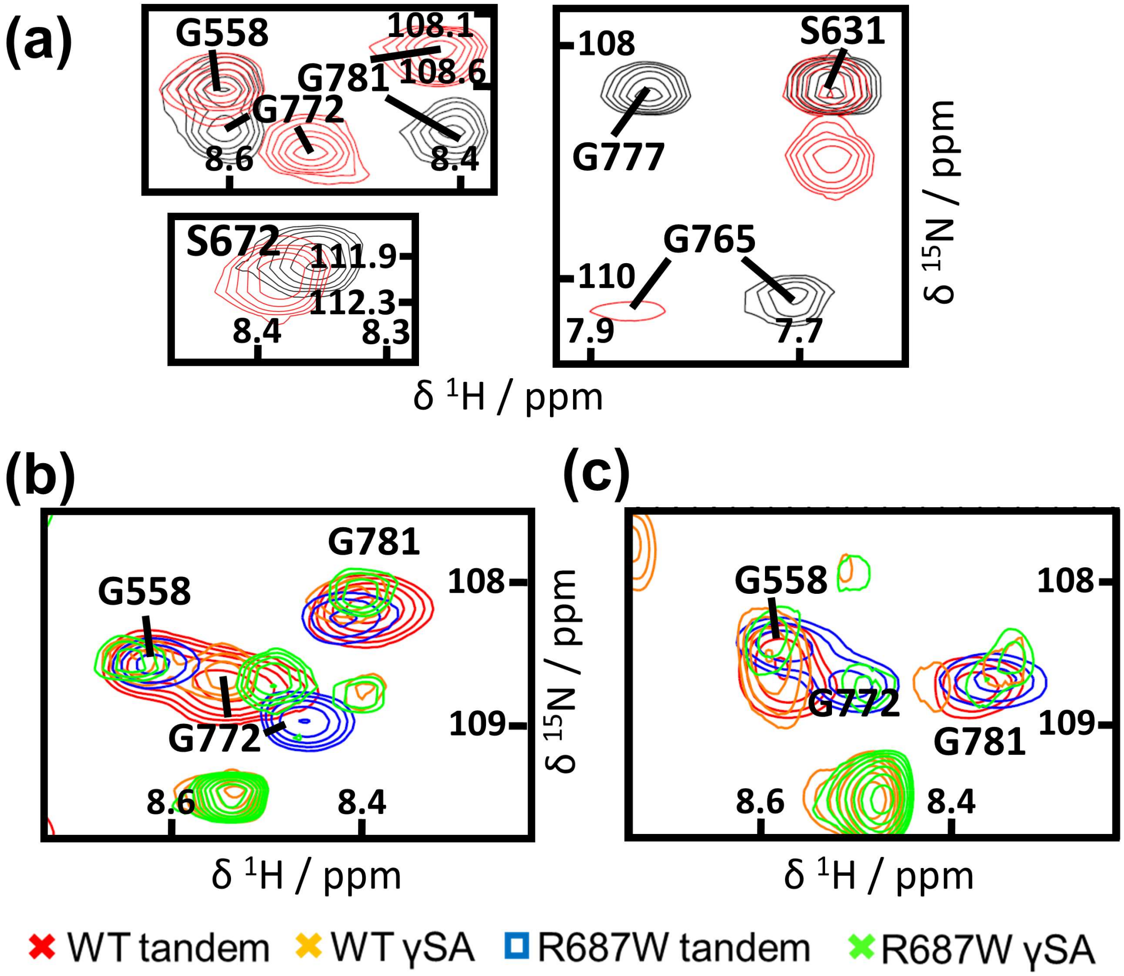

Figure S13. The NMR observations made in context of tandem-nSH2cSH2 constructs are reflected in ^{1}H,^{15}N-NMR spectra for the γ-specific array (γSA) (see also *Fig. 8*). (a) ^{1}H,^{15}N-SOFAST-HMQC spectra of NSH2CSH2 (red) and NSH2CSH2-pY^{783} (black). γSA spectra recorded at > 30°C are generally of higher quality, but for comparability with γSA-pY783, which is unstable at higher temperatures, both spectra were recorded at 25°C. Only cross peaks which were assigned for both γSA and γSA-pY783, or that are of particular relevance for this study, are highlighted here. A more complete backbone resonance assignment for nonphosphorylated γSA can be found elsewhere (10). (b-c) ^{1}H,^{15}N-SOFAST-HMQC spectra of residue Gly772/Gly781 in nonphosphorylated (b) and phosphorylated (c) tandem or γSA constructs. Symbols depict the cross peak centroids (color key depicted below). The 'closed' state is less populated in nonphosphorylated and Arg687Trp mutant constructs than in phosphorylated constructs and WT construct, respectively. Spectra were recorded at 34°C (panel a) or 25°C (panel b) and at 900 MHz (γSA-pY^{783}) or 700 MHz (all others).

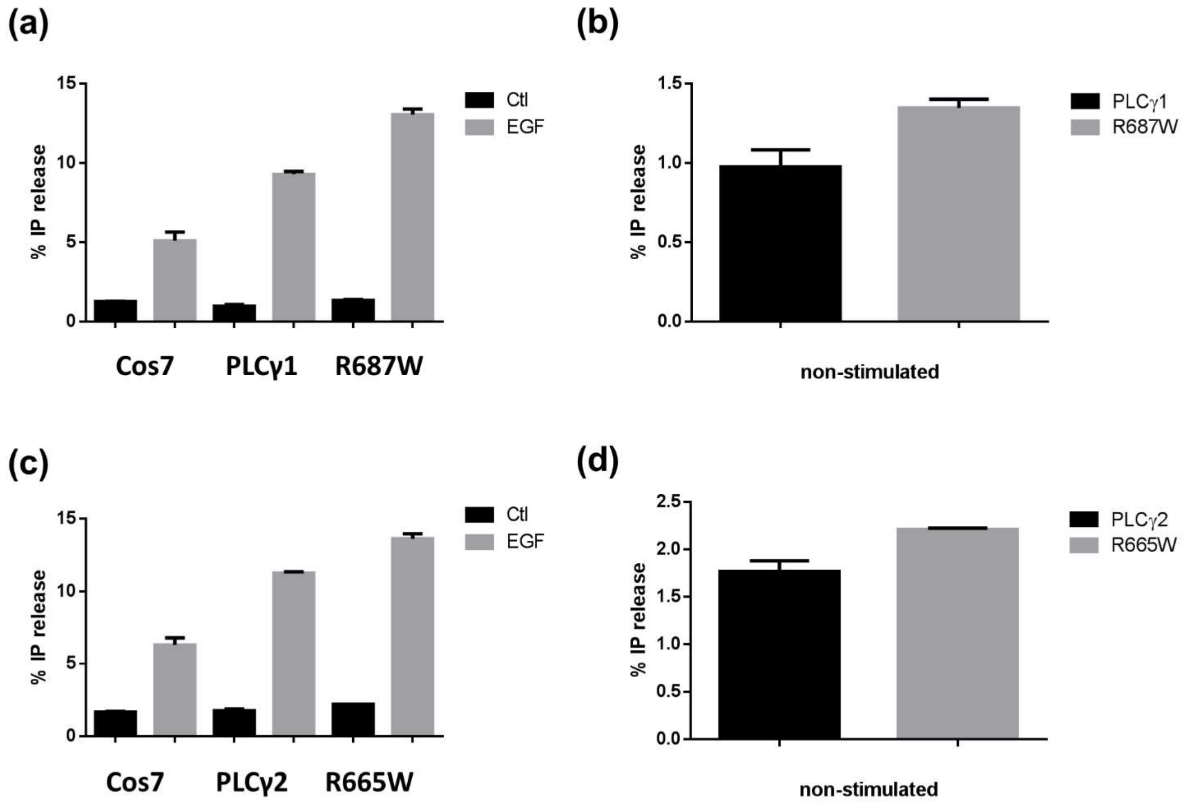

Figure S14: Activity assays. PLCγ containing Arg687Trp (PLCγ1) or Arg665Trp (PLCγ2) mutations expressed in COS-7 cells show increased activity with respect to wild type (WT) PLCγ, Panels (a)-(b) compare PLCγ1 activity. Panels (c)-(d) compare PLCγ2 activity. Panels (a) and (c): Basal activity (Ctl) and activity upon stimulation with EGF for COS-7 cells expressing different constructs are shown. The Arg687Trp mutant displays higher activity than WT upon activation by EGF; changes of basal activity are very moderate. Panels (b) and (d): only the small changes in the basal activity are shown.

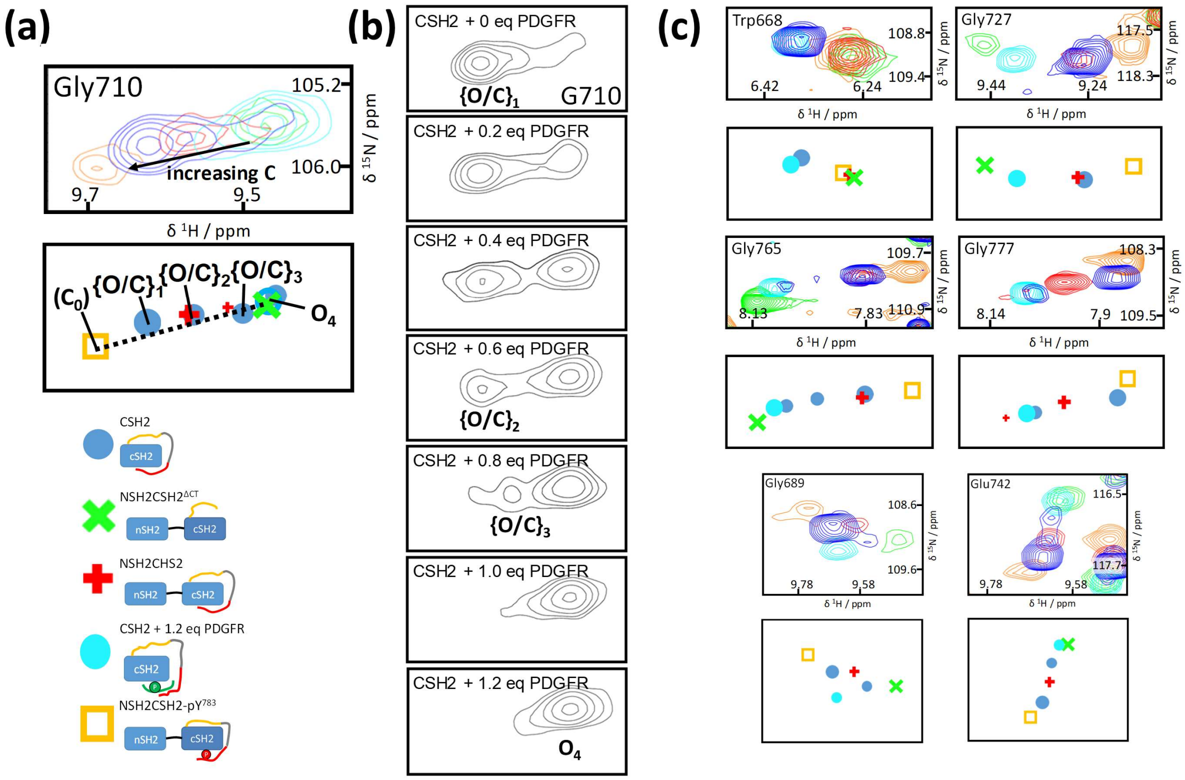

Figure S15: Titration of PDGFR phosphopeptide into CSH2. (a) ^{1}H,^{15}N-HSQC NMR spectra for the titration of PDGFR phosphopeptide into CSH2, focused on residue Gly710. CSH2 cross peaks observed for residue Gly710 coincide with the peak positions for some other constructs (the color scheme is similar to that employed in *Fig. 3*, extended to include the complex of CSH2 with the PDGFR peptide). The cross peak labeled 'C'$_0$ indicates the NSH2CSH2-pY^{783} 'closed' reference state that is not observed in CSH2. Four CSH2 states in slow exchange are identified: {O/C}$_1$, {O/C}$_2$, {O/C}$_3$ and O$_4$. (b) The titration with PDGFR peptide confirms kinetic connectivity between the states. (c) Additional examples supporting panels (a) and (b).
